# Potential Therapeutic Targets for Oral Cancer: ADM, TP53, EGFR, LYN, CTLA4, SKIL, CTGF, CD70

**DOI:** 10.1371/journal.pone.0102610

**Published:** 2014-07-16

**Authors:** Saurabh Bundela, Anjana Sharma, Prakash S. Bisen

**Affiliations:** 1 Defence Research Development Establishment, Defence Research Development Organization, Ministry of Defence, Govt. of India, Gwalior, Madhya Pradesh, India; 2 Department of Postgraduate Studies & Research in Biological Sciences, Rani Durgavati University, Jabalpur, Madhya Pradesh, India; 3 School of Studies in Biotechnology, Jiwaji University, Gwalior, Madhya Pradesh, India; National Institute of Genomic Medicine, Mexico

## Abstract

In India, oral cancer has consistently ranked among top three causes of cancer-related deaths, and it has emerged as a top cause for the cancer-related deaths among men. Lack of effective therapeutic options is one of the main challenges in clinical management of oral cancer patients. We interrogated large pool of samples from oral cancer gene expression studies to identify potential therapeutic targets that are involved in multiple cancer hallmark events. Therapeutic strategies directed towards such targets can be expected to effectively control cancer cells. Datasets from different gene expression studies were integrated by removing batch-effects and was used for downstream analyses, including differential expression analysis. Dependency network analysis was done to identify genes that undergo marked topological changes in oral cancer samples when compared with control samples. Causal reasoning analysis was carried out to identify significant hypotheses, which can explain gene expression profiles observed in oral cancer samples. Text-mining based approach was used to detect cancer hallmarks associated with genes significantly expressed in oral cancer. In all, 2365 genes were detected to be differentially expressed genes, which includes some of the highly differentially expressed genes like matrix metalloproteinases (MMP-1/3/10/13), chemokine (C-X-C motif) ligands (IL8, CXCL-10/-11), PTHLH, SERPINE1, NELL2, S100A7A, MAL, CRNN, TGM3, CLCA4, keratins (KRT-3/4/13/76/78), SERPINB11 and serine peptidase inhibitors (SPINK-5/7). XIST, TCEAL2, NRAS and FGFR2 are some of the important genes detected by dependency and causal network analysis. Literature mining analysis annotated 1014 genes, out of which 841 genes were statistically significantly annotated. The integration of output of various analyses, resulted in the list of potential therapeutic targets for oral cancer, which included targets such as ADM, TP53, EGFR, LYN, CTLA4, SKIL, CTGF and CD70.

## Introduction

About 7.6 million cancer deaths were estimated in 2008 worldwide, out of which 0.64 million people died from cancer in India [1]. Oral cancer has emerged as one of the top three causes of cancer-related deaths in South Asian countries like India, Bangladesh, and Sri Lanka [1]. According to the latest cancer statistics reported from India, oral cancer is the top-most cause of cancer related deaths in men, and it contributes about 23% of deaths caused by all cancer types in men [2]. India has become an epicenter of oral cancer-related mortalities, and according to a rough estimate more than half of the worldwide oral cancer mortalities are from India [1]–[3]. Oral cancer is currently managed through surgery, radiation and chemotherapy. Cetuximab is the only approved targeted therapy available for oral cancer, which targets epidermal growth factor receptor (EGFR) involved in cell growth. Targeted therapies have shown their usefulness in managing various cancers, mostly because of its ability to reduce toxicities by several folds when compared with chemotherapeutic drugs. The acquisition of resistance to targeted cancer therapies due to an emergence of various genetic and/or non-genetic mechanisms, have seriously undermined their clinical application [4]–[6]. The challenge of emergence of drug resistance in cancer cells can be addressed by - (a) targeting multiple targets by combination therapy, (b) designing a drug against molecular target(s) which are involved in diverse pathways critically linked with survival, growth and proliferation of cancer cells, or by the combination of (a) and (b).

**Figure 1:**
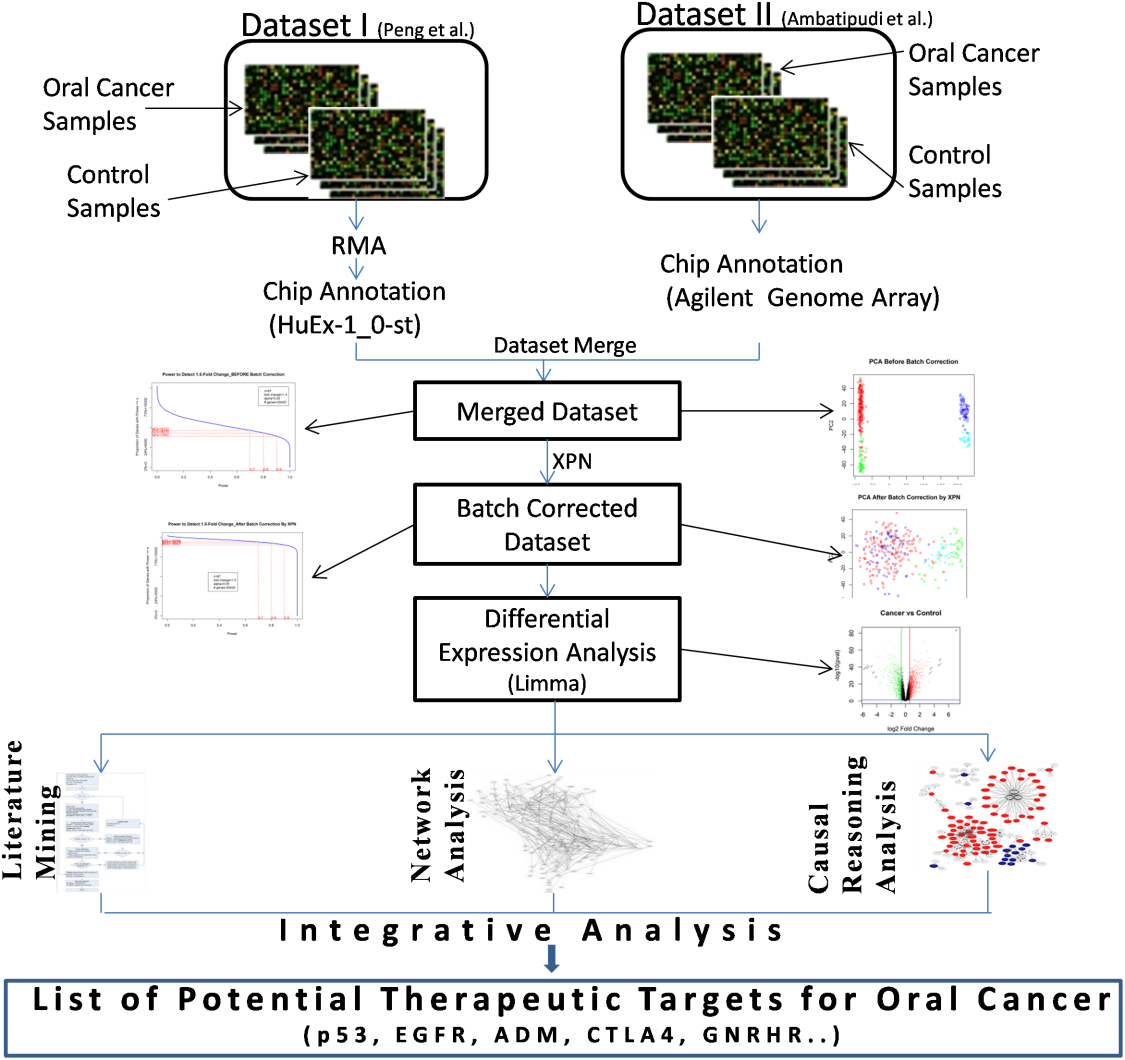
Process flow of identification of therapeutic targets for oral cancer.

The current study, attempts to identify potential therapeutic targets for oral cancer that are associated with multiple cancer hallmarks, which can facilitate rational discovery of effective therapies for oral cancer. We have used microarray datasets available from NCBI-GEO database, to study transcriptional profiles specifically altered in oral cancer. We have integrated dataset from two studies with similar experimental design (i.e. oral cancer vs. control) to derive meaningful results from underlying dataset with improved statistical power. The direct integration of dataset from different studies is challenging due to existence of myriad sources of non-biological variations, often referred as ‘batch-effects’. Such probe-level integration of dataset from two different studies is possible by removing batch-effects by cross-platform normalization [7]. Different analytical methods have been integrated to enable logical selection of the most promising therapeutic targets for oral cancer (Fig. 1). We have used gene dependency network analysis to understand topological properties under cancer and control condition, the genes with marked topological differences could be regarded as therapeutic target genes [8]. Causal reasoning analysis was used for identification of potential genes, which can explain differential gene expression changes in oral cancer. The development of cancer is a multistep process enabled by occurrence of key hallmark events like sustaining proliferative signaling, evading growth suppressors, resisting apoptotic cell death, enabling replicative immortality, inducing angiogenesis, activating invasion, metastasis and inflammation [9]. Novel literature mining method has been used to associate these cancer hallmarks to genes of our interest. In the present study, the diversity of cancer hallmarks associated with a gene, along with impressive topological profile in dependency- and/or causal-network, qualifies a gene to be a potential drug target for oral cancer.

Large-scale integration of datasets from oral cancer gene expression studies had been attempted in the past with an objective to mine transcriptional signatures linked with neoplastic transformation [10] or survival [11]. Recently, it has been used to identify frequent somatic drivers for oral carcinogenesis [12]. The task of identifying potential therapeutic targets by integrative analysis, has been attempted for the first time in the current study. With a surge in deaths caused by oral cancer especially in Indian subcontinent region, there is an urgent need to expedite our efforts to find novel therapies for oral cancer. The current study, present a logical framework to find potential therapeutic targets that are associated with multiple cancer hallmarks, and targeting them is thus expected to be a perfect answer to challenges associated with acquired drug-resistance to targeted therapies.

## Materials and Methods

### Data source

The gene expression data of oral cancer patients and normal persons (control samples), reported in two different studies [13], [14] were used in the current work (Table 1).

**Table 1:** Dataset Details.

DataSet	No. of CancerSamples	No. of ControlSamples	Platform	NCBI-GEOAccession	Study Reference
DS-1	57	22	Affymetrix HumanExon 1.0 ST Array – GeneVersion (HuEx-1_0-st)	GSE25099	Peng et al. [14]
DS-2	27	5	Agilent-014850 Whole HumanGenome Microarray 4×44KG4112F (Probe Name version)	GSE23558	Ambatipudi et al. [13]

### Direct Data Integration

The gene expression data generated by different experiments cannot be combined directly for downstream analysis, even after processing with similar normalization method, because of the inherent non-biological experimental variations or “batch-effects”. The direct integration of data is possible after processing datasets with appropriate normalization method followed by chip annotation and the post processing operations required for removal of the batch-effects with the help of batch correction methods.

#### Normalization

The raw data or CEL files used in the gene expression profiling study by Peng et al. [14] were downloaded from the NCBI gene expression data repository (NCBI-GEO), and the probe level summaries were obtained by Robust Multichip Analysis (RMA) algorithm [15] implemented in Affymetrix Expression Console software (version 1.3). The RMA algorithm fits a robust linear model at the probe level to minimize the effect of probe-specific affinity differences. The normalized dataset, deposited in NCBI-GEO by Ambatipudi et al. [13], was downloaded and used in the current study. The details of normalization procedures used for this dataset can be found in related publication [13].

#### Chip Annotation

The Netaffyx annotation file HuEx-1_0-st-v2.na33.1.hg19.transcript.csv was downloaded from http://www.affymetrix.com/, and used as a primary source of annotation for HuEx-1_0-st array dataset. Custom parser was written in perl to extract most relevant columns like Probeset ID, Representative Public ID, Entrez GeneID from these annotation files. The annotation file for Agilent-014850 Whole Human Genome Microarray 4x44K G4112F (Probe Name version) was downloaded from the corresponding platform file (GPL6480) available from the NCBI-GEO. Custom parser was written in perl to extract Entrez GeneID and Gene Symbol mapped against corresponding probe IDs.

The chip annotation was further enhanced with the help of gene2accession file downloaded from the NCBI ftp site (ftp://ftp.ncbi.nlm.nih.gov/gene/DATA). The gene2accession file helped us in finding missing Entrez GeneIDs for the probes based on other available information like rna/genomic nucleotide accession id which is a common field between annotation file and gene2accession. We could annotate 30,932 probes in Agilent-014850 Whole Human Genome Microarray 4x44K G4112F (Probe Name version) and 38,349 probes in HuEx-1_0-st (transcript version) with the corresponding Entrez GeneIDs. Probes without annotation were not considered for downstream analytical processes.

#### Dealing with many-to-many relationship between Probes and Genes

There is not always one to one correspondence between microarray probes and associated genes, which creates ambiguity while analyzing results of downstream statistical and/or functional analysis. Two types of specific cases arise because of the many-to-many relationships between probes and genes, viz. (a) one probe is mapped to more than one GeneID (e.g. Probe1-> BIRC5, BIRC3), due to a non-specific nature of the probe, and (b) more than one probe can map to same GeneID, often referred as “sibling” probes (e.g. Probe1-> BIRC5, Probe2-> BIRC5), which usually occurs due to clustering nature of secondary databases (UniGene, RefSeq) or due to duplicate spotted probes.

Considering only probes with one-to-one relationship would be the simplest analytical approach; however, it would mean losing information. Ramasamy et al. [16] recommended replacing probes mapped to multiple genes with new record for each GeneID. We have written custom perl script for “expanding” the probes with multiple genes to deal with non-specific probes, which maps to more than one gene. This creates new record for each GeneID.

The information spread across sibling probes was consolidated with the help of a robust statistic, the Tukey’s biweight [17]. The median related Tukey’s biweight is a robust statistic, which is known to have excellent behaviour in the presence or absence of outliers, because of these attributes, it was implemented in MAS5.0 algorithm used for probe level summarization [18]. Custom scripts were written in perl and R to deal with sibling probes, and the R method ‘tbrm()’ available with dplR package was used to compute Tukey’s biweight robust mean. Groups of sibling probes were identified, and these records were replaced by single representative record in which expression values spread across sibling probes were replaced by Tukey’s biweight robust mean; this process was repeated for every sibling probe group.

After resolving many-to-many relationship between probes and genes, 19,593 and 23,407 probes/genes were retained in Agilent-014850 Whole Genome and HuEx-1_0-st arrays, respectively. Both datasets were further merged based on common field, i.e. Entrez GeneID. The merged dataset consisted of 18,927 probes/genes, 84 cancer samples and 27 control samples. This merged dataset was used for the subsequent batch correction process.

#### Batch Correction

We used two analytical methods, i.e. ComBat [19] and XPN [20] to deal with non-biological variations or batch-effects. These methods were reported to outperform other cross-platform normalization techniques [21], [22].

The R implementation of ComBat (www.bu.edu/jlab/wp-assets/ComBat/) was used for removing batch-effects from the two datasets. Similarly normalized datasets were processed by XPN method, implemented in CONOR package [22] available with the CRAN package repository (cran.r-project.org/web/packages/). The normalized and batch corrected data will allow probe/gene level integration of data from two studies, thus facilitate a generation of the robust hypotheses on data with improved statistical power.

#### Assessment of Quality of Batch Correction

The batch corrected dataset was assessed for attributes like distribution of sample types and change in experimental power. This was done for choosing among ComBat and XPN, as a batch correction method which suits best for our dataset. R implementation of Principal Component Analysis - PCA (i.e. prcomp() method) was used for the assessment of distribution of cancer and control samples between two dataset used in the current study [13], [14]. The R statistical package ssize() was used for estimation of experimental power [23].

### Differential expression analysis

The normalized and batch corrected dataset was used for further analysis. The differential expression analysis was performed using LIMMA package (version 3.14.4) with least-squares regression and empirical Bayes moderated t-statistics [24], [25]. The design matrix was constructed to represent the layout of the cancer and control samples in the data-matrix. The difference in expression levels of samples in two conditions was studied by setting contrast ‘cancer-control’. P-values were adjusted for multiple comparisons using the Benjamini Hochberg false discovery rate correction or ‘fdr’ [26]. Genes with the adjusted p-value less than or equal to 0.05 and the fold change threshold of 1.5 were considered as differentially expressed, in the current study.

### Network Analysis

The R statistical package ‘GeneNet’ (version 1.2.7) [27] was used to infer large-scale gene association networks among differentially expressed genes obtained in our study. The association networks inferred by GeneNet are graphical Gaussian models (GGMs), which represent multivariate dependencies in bio-molecular networks by partial correlation. This method produces a graph in which each node represents a gene, and the edges represent direct dependencies between connecting nodes/genes. This method also computes statistical significance value (p-value) along with fdr corrected/adjusted q-value for the edges in GGM network, which provides a mechanism to extract only significant edges in the network. Dependency network was generated for each condition independently. The threshold of q-value less than or equal to 0.05, was used to filter out non-significant edges in the final network. Custom perl scripts were written to extract connectivity or degree statistics of networks for cancer and control samples.

### Causal Reasoning

Causal reasoning attempts to explain the putative biological causes of the observed gene expression changes based on directed causal relationships. Causal relationships can be represented as ‘causal graphs’, which consist of nodes (gene/biological process), and directed edges depicting the relationship between connecting nodes. Biological regulation can also be represented in such causal graphs in the form of signed edges, with the sign indicating whether a change in the causal variable affects the second variable positively or negatively.

In the current study, we have applied causal reasoning method proposed by Chindelevitch et al. [28], to retrieve the list of statistically significant upstream hypotheses, which explains observed gene expression changes in our study dataset. This method identifies putative upstream hypothesis based on a set of causal relationships represented as a causal graph, and ranks such a hypothesis by computing their cumulative score based on nature of prediction (correct = +1, incorrect = −1, ambiguous = 0) made by hypothesis in the causal graph. This method also computes statistical significance of each score and output’s hypotheses that are statistically significant.

The R-code of causal reasoning method [28] requires three inputs viz. (i) Causal Network Entities: a tab-delimited file consisting of information about entities of causal network, in our study it consisted of the list of genes, which are part of causal network, (ii) Differentially Expressed Genelist: a tab-delimited file consisting of two columns (i.e. gene name and direction of regulation, which is 1 or −1 for up- or down-regulation), (iii) Causal Network Relationships: a tab-delimited file consisting of constituting entities (i.e. source gene to target gene) and type of relationship between entities (type: “increase” or “decrease” describes the causal effect of source on target). The output files produced by this method are: (i) HypothesisTable.xls (see Text S4): a tab-delimited file, each row of which is a hypothesis (i.e. an entity in the graph with a direction of + or − and a number of downstream steps that are taken to predict transcripts) and column consists of score, the name and number of correct, incorrect, and not explained transcripts as well as p-values and Bonferroni corrected p-value [29], [30] as a conservative estimate of significance under multiple testing correction (ii) XGMML files: causal sub-graphs of significant hypothesis detected by the method are generated in xgmml format.

#### Causal Graph Creation

We have used causal relationship embedded in KEGG pathways [31] as a source of generating the causal graph in the current study. KEGG API was leveraged as a framework for parsing entities and relationships from kgml file of a pathway. KEGG pathways for human were considered for collecting information required to construct the causal network. The kgml file contains entity list (gene/compound etc.) and relationship information (activation/inhibition/expression etc.). We have considered ‘activation’ and ‘inhibition’ along with entities involved in such a relationship for constructing the causal graph. The final causal graph generated from KEGG pathways consisted of 11,586 causal relationships.

#### Post processing of XGMML files and generation of consolidated Causal Network

The xgmml files generated by causal reasoning analysis were parsed by custom perl script to extract critical information about upstream hypothesis and to create a consolidated causal network. The hypotheses and the predicted relationships were further subjected to screen to remove hypotheses not supported by our data and also to remove falsely predicted causal relationships, which can be identified as ‘I(+/−)’ in Text S5. The correctly predicted relationships can be identified as ‘C(+/−)’ in Text S5. The hypotheses which were not differentially expressed were checked for its expression level (i.e. up/down-regulation) depicted in causal graph and then compared with its corresponding expression level in our dataset. Any hypothesis with contradicting direction in expression profile (i.e. up-regulated in the causal graph and down-regulated in expression dataset, or vice-versa) was not considered for further analysis. Thus, the correctly predicted hypotheses will include only those hypotheses which can be corroborated by integrated expression dataset used in the current study (i.e. hypothesis depicted as over-expressed in causal network, should also show over-expression in expression dataset, or vice-versa).

The correctly predicted relationships and hypotheses were considered while creating the consolidated causal network. Connectivity information along with nature of relationship (increases/decreases) between hypothesis and downstream genes were saved in ‘Causal_Net.rel’ (see Text S6). Connectivity statistics were also computed for all edges in final causal network and saved in ‘Causal_Net.degree’ (see Text S7).

### Literature Mining

Differentially expressed genes were considered for functional analysis based on information available in published articles archived in NCBI PubMed database. The NCBI eUtils, in particular, Esearch and Efetch, were used along with Perl LWP module, for mining NCBI PubMed database [32]. The scope of literature search with gene symbol of differentially expressed genes was expanded by using gene synonym table, queries incorporating synonyms along with other search terms were then sent to PubMed using the Esearch utility, followed by retrieval of relevant records by Efetch utility.

**Figure 2:**
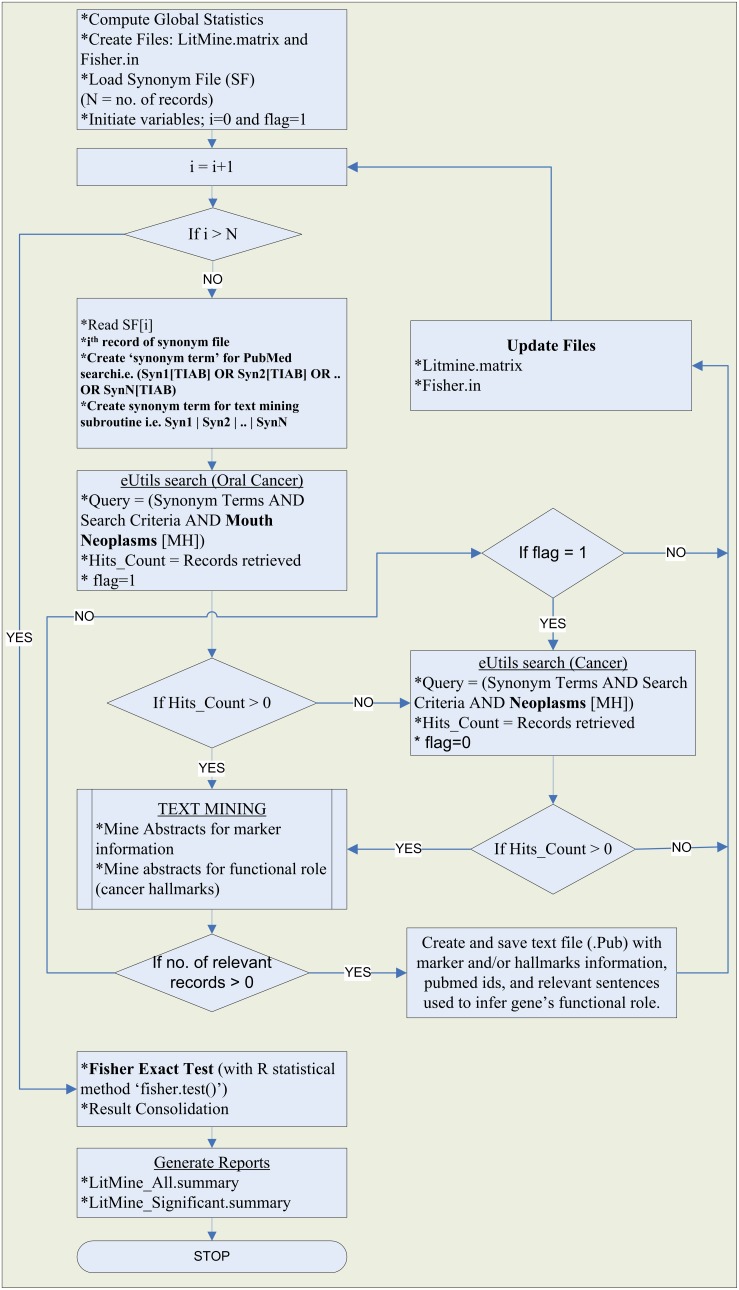
Literature Mining Process Flow.

The method uses text-mining rules defined in algorithm, to classify differentially expressed genes according to the marker type (therapeutic/diagnostic/prognostic) and relevant cancer hallmarks (apoptosis/cell-proliferation/angiogenesis/metastasis/inflammation) reported for the concerned gene in articles published in NCBI-PubMed. The algorithm computes statistical significance of search statistics and consolidates literature mining results as report files. The algorithmic flow of literature mining method used in the current study is depicted in Fig. 2.

Perl script was written for functional annotation of input gene-list, based on the text mining of relevant articles retrieved with the help of NCBI eUtils. The literature mining algorithm implemented in current study consists of following major components:

1. Creation of gene-synonym table.
2. Query formation.
3. Text-mining.
4. Significance analysis of the text-mining result.

#### Gene synonym table

The tab-delimited ‘gene_info’ file was downloaded from NCBI ftp site and was used to create gene synonym table. The entries for human were extracted from the gene_info file with the help of organism code for human (Taxonomy id: 9606), and these entries were used to create an intermediate file, which was further used to create gene synonym table. The columns of the intermediate file which were used to generate alternative names for the genes are: (i) ‘gene synonyms’, (ii) ‘descriptive name’, and (iii) ‘other names’.

The resulting gene synonym table was saved as a tab-delimited file with two columns viz. gene symbol and synonyms. An entry in the gene synonym table was in following format:

MMP1 CLG#fibroblast collagenase#interstitial collagenase#matrix metalloprotease 1#matrix metalloproteinase 1.

#### Query formation

The search queries were optimized by using appropriate search tags [33], for retrieving relevant articles from PubMed. This optimization was necessary because PubMed does not support phrase searches. While searching for phrase consisting of multiple words, PubMed search would return articles having all words in the phrase spread across different places in abstract. This default behavior of PubMed can be controlled by using search tags. The search tag ‘[TIAB]’ (Title/Abstract) was used after the gene terms and biological concepts like apoptosis or angiogenesis, which were used for querying PubMed database. Further, the search tag ‘[MH]’ (MeSH Terms) was applied for restricting context of search specific to oral cancer by using MeSH term “mouth neoplasms[MH]” and have used the query term “neoplasms[MH]” for searching articles related to any cancer type.

The queries used by our method can be broadly divided into two categories viz.

- **Global Queries**: These queries were used to extract search global statistics for computing statistical significance of literature mining results. The global statistics required for Fisher Exact test includes the total number of articles related with oral cancer/cancer, and number of articles related to the functional concept (like apoptosis, metastasis, angiogenesis etc.) as well as oral cancer/cancer.

E.g. (cell death[TIAB] OR apoptosis[TIAB] OR apoptotic[TIAB] OR anti-apoptosis[TIAB] OR anti-apoptotic[TIAB]) AND mouth neoplasms[MH].

- **Gene specific Queries**: Gene symbols from the differentially expressed gene-list were translated into corresponding synonyms with the help of gene synonym table. Gene specific queries incorporating synonyms, keywords for concepts and cancer-type (mouth neoplasms or neoplasms) were sent to PubMed using Esearch utility, followed by retrieval of relevant records using the Efetch utility. No restriction was set for the number of articles retrieved per query, since our objective was to assign annotation based on consensus among published articles. Since oral cancer is the focus of this study, the initial attempt of our method was to query among articles related to oral cancer, and then to consider articles related to any cancer-types only in condition of failure to retrieve any information with specific context to oral cancer. This was done to improve annotation rate of the input gene-list.

E.g. ((MMP1[TIAB] OR CLG[TIAB] OR fibroblast collagenase[TIAB] OR interstitial collagenase[TIAB] OR matrix metalloprotease 1[TIAB] OR matrix metalloproteinase 1[TIAB]) AND (((therapeutic[TIAB] OR therapy[TIAB] OR diagnostic[TIAB] OR diagnosis[TIAB] OR prognostic[TIAB] OR prognosis[TIAB] OR inflammatory[TIAB]) AND (target[TIAB] OR molecule[TIAB] OR marker[TIAB])) OR (cell[TIAB] AND (proliferation[TIAB] OR proliferative[TIAB] OR death[TIAB] OR growth[TIAB] OR immortalization[TIAB] OR migration[TIAB])) OR (apoptosis[TIAB] OR apoptotic[TIAB] OR anti-apoptosis[TIAB] OR anti-apoptotic[TIAB] OR angiogenesis[TIAB] OR metastasis[TIAB] OR metastatic[TIAB] OR inflammation[TIAB] OR invasion[TIAB] OR (immune[TIAB] AND (modulation[TIAB] OR resistance[TIAB] OR destruction[TIAB]))))) AND mouth neoplasms[MH].

#### Text Mining

The relevant articles were retrieved in PubMed ‘XML’ format, which makes information extraction more precise due to presence of content enclosed within xml tag pairs. Review articles were not considered for text mining, because it may lead to extraction of redundant information, which is already captured by mining of the original research articles referred in those review articles. The abstract section of articles was considered for text mining. In an article, the gene name can be used as an acronym for a concept unrelated to gene and thus can become a source of false-positive [34], [35]. Our method attempts to resolve ambiguity caused by an acronym by searching for expanded form of the acronym in the content preceding an acronym and then comparing it with synonyms of the acronym retrieved from gene synonym table. The abstract is excluded from the analysis, if no match is found in the synonym list.

The abstract section of any article is a gist of the article, which contains concise information about background, results and conclusions of the work mentioned in the articles. A lot of variations can be seen in the structure of abstract section of research articles. Some articles have separate subsections for background, results, and conclusions, whereas other articles would have all these information written under abstract section without any sub-sectioning. The content of ‘conclusions’ subsection of articles can be considered as the most informative and less ambiguous for functional annotation tasks like ours. The content used for text mining in our method was extracted from the ‘conclusions’ subsection of articles with well-defined subsections in abstract section. For other articles without sub-sectioned abstract, our method extracts this information from the last 25% portion of the abstract section with an assumption based on general observation that conclusions invariably appear towards the end of abstract and make up about a quarter of the entire content in the abstract section.

Perl regular expression was used to detect the presence of keywords related with marker-types and/or cancer hallmarks in the content that is extracted from abstract section of the article. The keyword containing extracted content was divided into units of single sentence. The parsing of such a single sentence when compared to the parsing of entire paragraph as a single unit has been reported to yield higher effectiveness for text-mining based information extraction [36]. The perl module “Lingua::EN::Sentence” was used for sentence boundary detection, it splits input textual content into sentences for downstream analysis. Sentences containing both expanded gene synonyms and keywords related with marker-type and/or cancer hallmarks were used to assign annotation to the gene. Case insensitive regular expression matching was performed to detect sentences containing keywords of interest and gene synonyms. The keywords used for functional annotating genes in the current study can be broadly classified under following two categories:

- **Marker related keywords:**
  - **Therapeutic marker**: a gene was considered as the therapeutic marker if the gene/synonym containing sentence have one or more items from the related keyword-list [therapeutic or therapy].
  - **Prognostic marker**: a gene was considered as the prognostic marker if the gene/synonym containing sentences have one or more items from the related keyword-list [prognostic or prognosis].
  - **Diagnostic marker**: a gene was considered as the diagnostic marker if the gene/synonym containing sentences have one or more items from the related keyword-list [diagnostic or diagnosis or predictive or tumor marker].
- **Cancer hallmark related keywords:**
  - **Apoptosis**: a gene was considered to be associated with apoptosis if the gene/synonym containing sentences have one or more items from the related keyword-list [cell death or apoptosis or anti-apoptosis or anti-apoptotic].
  - **Angiogenesis**: a gene was considered to be associated with angiogenesis if the gene/synonym containing sentences have one or more items from the related keyword-list [angiogenesis or angiogenic].
  - **Cell proliferation**: a gene was considered to be associated with cell proliferation if the gene/synonym containing sentences have one or more items from the related keyword-list [cell growth or proliferation or proliferative].
  - **Metastasis**: a gene was considered to be associated with metastasis if the gene/synonym containing sentences have one or more items from the related keyword-list [cell migration or cell motility or invasion or metastasis or metastases or metastatic]. Although invasion and metastasis characteristically differ in the strict sense, however, they were grouped together in current study for interpretational simplicity, and also because both are associated with worse prognosis and poor survival.
  - **Inflammation**: a gene was considered to be associated with inflammation if the gene/synonym containing sentences have one or more items from the related keyword-list [inflammation or inflammatory].

For an instance, a sentence with the co-occurrence of ‘matrix metalloproteinase 1′ (synonym of the gene ‘MMP1’) and ‘metastatic’, will assign metastatic function to MMP1.

The text mining results of successfully annotated genes by the current method were saved as <gene_symbol>_pub.txt files for validation and future reference. Search statistics were saved in file named ‘LitMine.matrix’ (see Text S9) and ‘Fisher.in’, which contains information in a matrix format where rows represent genes, and columns represent various statistics like number of PubMed articles assigning apoptosis as a cancer hallmark functionally related with particular gene.

#### Significance Analysis (Fisher’s Exact Test)

The R implementation of Fisher’s exact test [37] was used for estimating statistical significance of the literature mining results. The search statistics were retrieved from ‘Fisher.in’ file and were used as an input for Fisher’s exact test computation by ‘fisher.test()’ method. For every gene in the gene-list, list of p-values corresponding to various biological concepts mined from literature (markers and cancer hallmarks) were generated and saved in ‘Fisher.out’ file. The p-value of less than equal to 0.05 for a concept was considered to be significantly associated with the concerned gene.

For e.g.: Among research articles published for oral cancer (53,049 articles in PubMed), 5190 are related with apoptosis. Our text mining process detected 27 relevant articles published for BIRC5/survivin in context with oral cancer, and among them 20 articles supported apoptotic role for BIRC5. The 2×2 contingency matrix was generated using PubMed search statistics for BIRC5 as illustrated in Table 2, and was used to compute p-value by Fisher’s exact test. Fisher’s exact p-value computed for the data in Table 2 is 2.991e-15, which is less than 0.05, implying that the apoptosis is significantly associated with BIRC5 and this association is not just due to a random chance.

**Table 2:** A 2×2 contingency table is built on search statistics for BIRC5.

	Total no. of articlesfor Oral Cancer	Total no. of articles for BIRC5AND oral cancer
Not involved inApoptosis	47,859	7
Involved inApoptosis	5,190	20

### Integrative Analysis

Results obtained in analytical processes like network analysis, causal network analysis and literature mining were integrated. In order to detect the most potential therapeutic target, we hypothesized that it should be involved in more than one cancer hallmark (apoptosis, angiogenesis, metastasis, cell-proliferation, inflammation) and therefore, targeting it would essentially control cancer cell due to its network statistics and ability to deal with diverse pathways involved in different cancer hallmarks.

The genes which are significantly associated with at least one out of five cancer hallmarks were selected. The difference in connectivity in the dependency network between cancer and control condition (denoted by ‘Diff’) was computed, to get estimate for the topological changes in a gene under two conditions. Genes with ‘Diff’ value greater than the average of ‘Diff’ values across selected genes were identified as topologically evolved (*TE*) genes. Genes which are either topologically evolved (*TE*) or are part of causal network were selected for further processing. The selected genes were further filtered based on the number of cancer hallmarks associated with them. Genes which were associated with at least two cancer hallmarks were considered as potential therapeutic targets for oral cancer. The target information of these potential therapeutic targets was further enriched by mining TTD-Therapeutic Target Database [38].

## Results and Discussion

**Figure 3:**
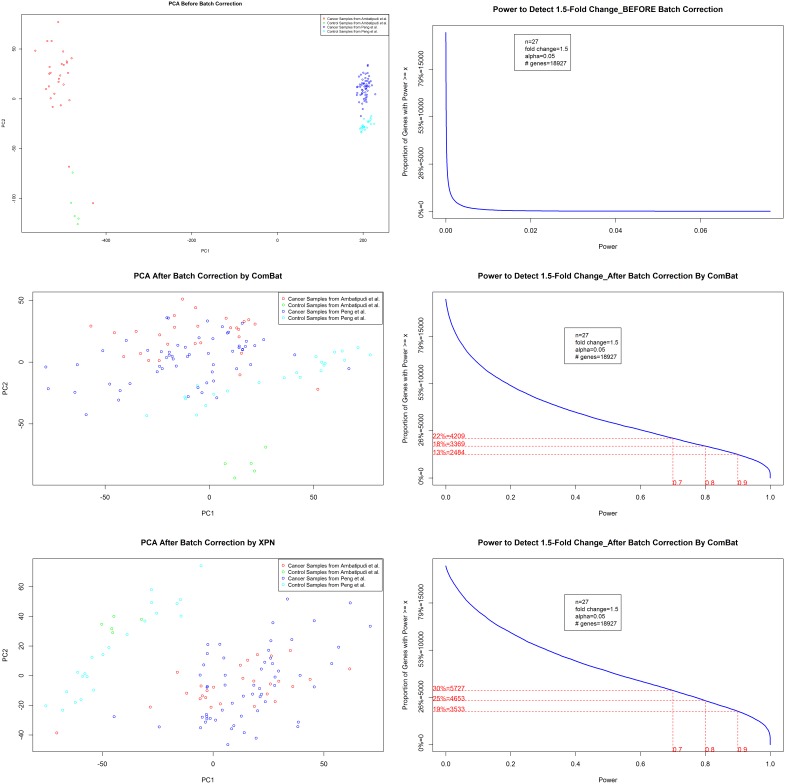
Data Attributes Before and After Batch-Correction. Samples are depicted as colored dots in PCA plots, “red” and “green” colored dots represents cancer and control samples, respectively, from Ambatipudi et al., 2012, whereas “blue” and “cyan” colored dots represents cancer and control samples, respectively, from Peng et al., 2011. The plots (a) and (b) are PCA and Power distribution plot for dataset before batch correction. The plots (c) and (b) are PCA and Power distribution plot for dataset after batch correction by ComBat. The plots (a) and (b) are PCA and Power distribution plot for dataset after batch correction by XPN.

The PCA and power distribution analysis of normalized data before and after batch correction (Fig. 3) clearly suggested XPN to perform better than ComBat for removing batch effects in dataset integrated from the two different studies. The dataset before batch correction occupies two distinct regions of PCA plot with respect to the originating study (Fig. 3(a)), which points to the existence of batch effects in dataset, with similar experimental design (Oral Cancer vs. Control). Both methods could remove inter-study heterogeneity among samples from cancer patients; however, XPN performed better than ComBat with respect to removing inter-study heterogeneity among samples from control or normal persons (Fig. 3). Our analysis showed significant improvement of statistical power in integrated dataset after batch correction by XPN and Combat (Fig. 3). We have selected normalized, and batch corrected data by XPN method for the downstream analysis, because of its ability to better resolve inter-study variability and improved statistical power.

**Figure 4:**
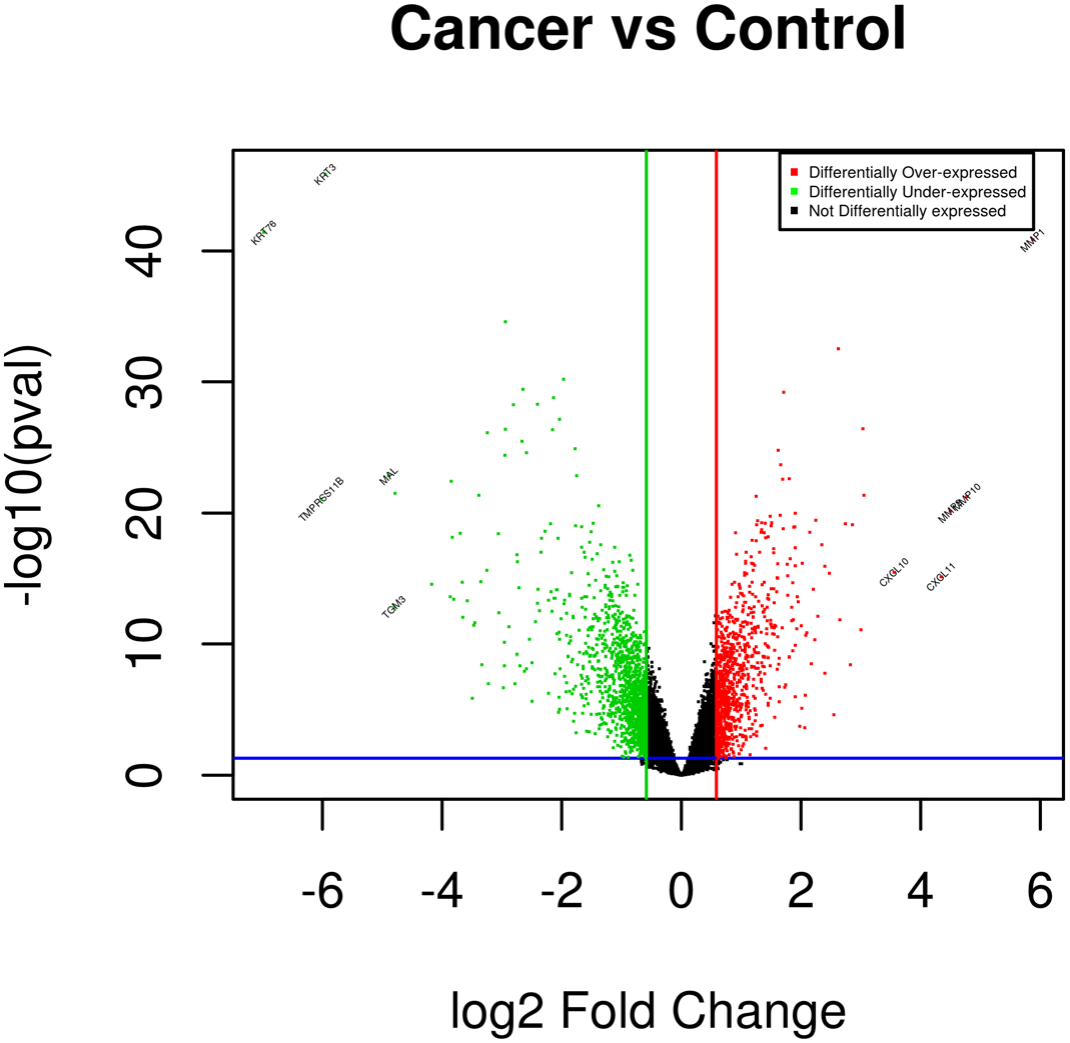
Volcano Plot. Significantly *overexpressed* genes are represented as ‘red’ dots and significant *underexpressed* genes are represented as ‘green’ dots in volcano plot. The names of some of the highly under- and over-expressed genes can be seen at left and right side respectively, of the volcano plot.

The batch-corrected dataset by XPN method consisted of 18,927 genes, which were used as an input for differential expression analysis by limma. Our analysis detected 2,365 genes to be differentially expressed at a fold change threshold of 1.5 and fdr corrected p-value threshold of 0.05. Differentially expressed genes consist of 938 overexpressed genes, which include some of the highly overexpressed genes like matrix metalloproteinases (MMP-1/3/10/13), chemokine (C-X-C motif) ligands (IL8, CXCL-10/11), PTHLH, NELL2, S100A7A, SERPINE1. Analysis detected 1,427 genes to be under-expressed, which include some of highly under-expressed gene like MAL, cornulin (CRNN), TGM3, CLCA4, keratins (KRT-3/4/13/76/78), SERPINB11, serine peptidase inhibitors (SPINK-5/7). Differential expression in our dataset is represented as a volcano plot (Fig. 4). For the complete list of the differentially expressed genes in our study, the file – ‘DE_genes.txt’ (see Text S1) can be referred, which is available as online supplementary material.

The result of differential expression analysis on the integrated dataset was compared with the differentially expressed gene-list reported in the studies related with the datasets used in the current study. Peng et al. reported 24 genes to be differentially expressed between tumors and normal tissue, at the fdr corrected p value threshold of 0.05 [14]. At the same level of significance threshold (corrected p value < = 0.05) our study detected 22 out of these 24 genes to be differentially expressed. We checked the details of two genes (DEPDC6 and NDUFB9) which our study was not able to reproduce, and found that these two genes were excluded from the integrated data matrix because of differences in microarray platform used in previous studies (the genes which were common in arrays used in previous studies [13], [14] were included in the integrated data matrix, for details see Section ‘*Direct Data Integration*’). Ambatipudi et al. reported 315 genes to be differentially expressed between tumors and normal tissues [13]. The integrated dataset generated in our study had 303 genes out of these 315 genes, and the remaining 12 genes were excluded because of aforementioned platform difference between the studies ([13], [14]). The differentially expressed gene-list obtained in the current study has 262 out of these 303 genes (∼85% overlap), which included key genes like SPP1, CA9, HOXC9, TNFRSF12A, LY6K, INHBA, FST, MFAP5, DHRS2, MAL, GPX3, LY6K, SERPINE1, GBP5, MMP10, MMP3, PTHLH, KRT4, ALOX12, EPHX2, and PTGD highlighted by Ambatipudi et al. [13]. It was observed that, the genes with consistent expression profile among source datasets ([13], [14]) were identified as differentially expressed genes in the current study. The detailed result of this comparison can be found in the file – ‘Comparison_with_previous_studies.xlsx’ (see Text S2), which is available as online supplementary material.

The differentially expressed genes were used to generate dependency network under two conditions, viz. cancer and control. Dependency network generated for cancer condition had 1,94,950 significant edges, which were 6.97% of possible edges, whereas dependency network under control condition resulted in 1,875 significant edges which was 0.07% of possible edges. The resultant dependency networks for cancer and control were compared to identify genes, which undergo marked changes at a connectivity level in the network. Such genes have a potential to be used as therapeutic and/or diagnostic markers. Some of the genes with marked difference in connectivity under two conditions are TCEAL2, TGIF1, XIST and CBX7. For the detailed list of network connectivity differences in genes under cancer and control condition, ‘Connections.txt’ (see Text S3) can be referred, which is available as online supplementary material.

**Figure 5:**
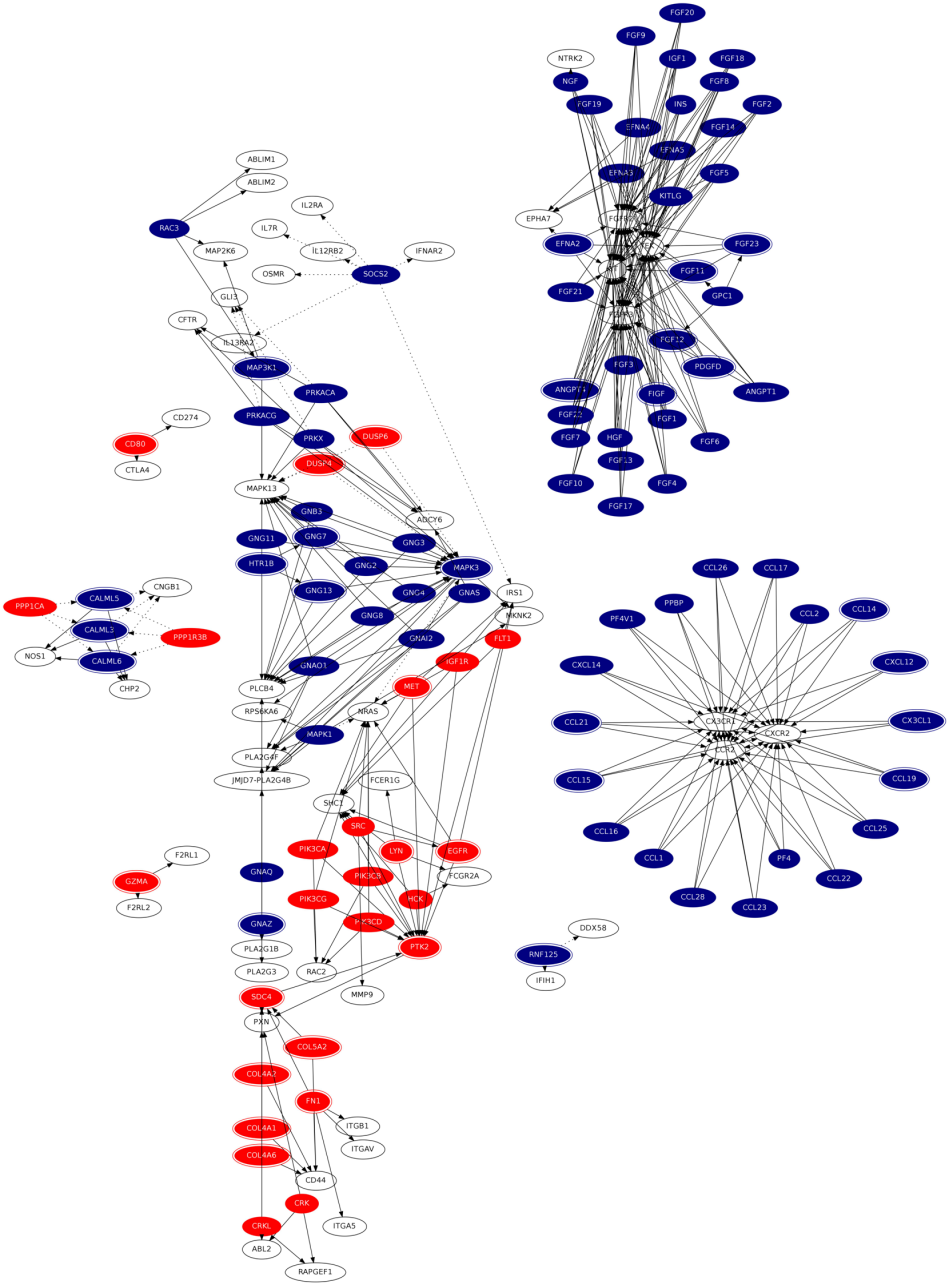
The Consolidated Causal Network. The genes are depicted as *nodes* of causal network. The hypotheses genes are distinctly colored as ‘red’ or ‘blue’ representing their over- or under-expression respectively, observed in study dataset. Relationships are depicted as *edge* or arrow in causal network. The solid arrow represents ‘activation’ relationship between connected nodes, whereas dashed arrow represents ‘inhibition’ relationship between the connected nodes. The node which has been identified as hypothesis gene, and also downstream gene for some other hypothesis, has been marked with an extra peripheral surrounding.

The differentially expressed genes were used as an input for causal reasoning analysis with an objective to retrieve potential upstream hypotheses explaining transcriptional changes involved in development of oral cancer. Our analysis detected 176 significant hypotheses, explaining 804 causal relationships from the causal graph constructed. The detailed list of hypotheses and downstream predicted genes can be found in Text S4 (output file of causal reasoning analysis) and ‘Causal_Net.summary’ (see Text S5) (generated by consolidating causal network files produced for each significant hypothesis detected by analysis), available as online supplementary material. The consolidated causal network (Fig. 5) was constructed after filtering out incorrectly predicted relationships and hypotheses. The consolidated causal network consisted of 106 hypotheses and 372 causal relationships correctly predicted by the method. Some of the highly connected genes in the resulting causal network are from chemokine signaling pathway (CX3CR1, CXCR2, CCR2, PTK2, NRAS), PI3K-Akt signaling pathway (FGFR2, KIT, FGFR3, TEK) and other pathways known to be associated with various cancers. The synopsis of the consolidated causal network along with its connectivity statistics can be found in Text S6 and Text S7, respectively, available as online supplementary material.

**Figure 6:**
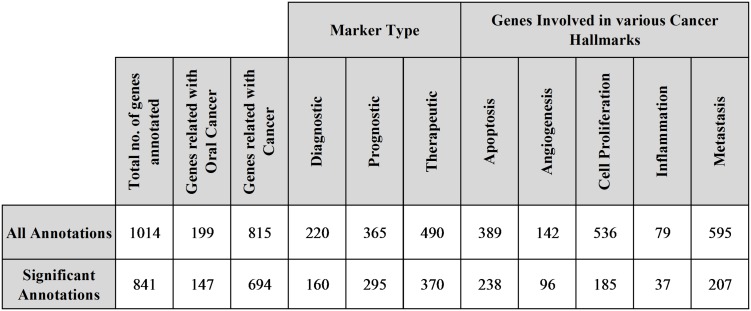
Literature Mining Result Statistics.

The functional annotation of differentially expressed genes was done by novel literature mining based approach. Our method successfully annotated 1,014 genes, out of which 841 genes were detected to be statistically significantly annotated (Fig. 6). The key findings from text mining analysis of successfully annotated genes were recorded for further reference and manual validation in the corresponding <gene_symbol>_pub.txt files; these files are available in ‘Gene_pubs.zip’ (see Text S8), along with other results files like Text S9, ‘LitMine_All.summary’ (see Text S10) and ‘LitMine_Significant.summary’ (see Text S11), which are available as online supplementary material.

**Figure 7:**
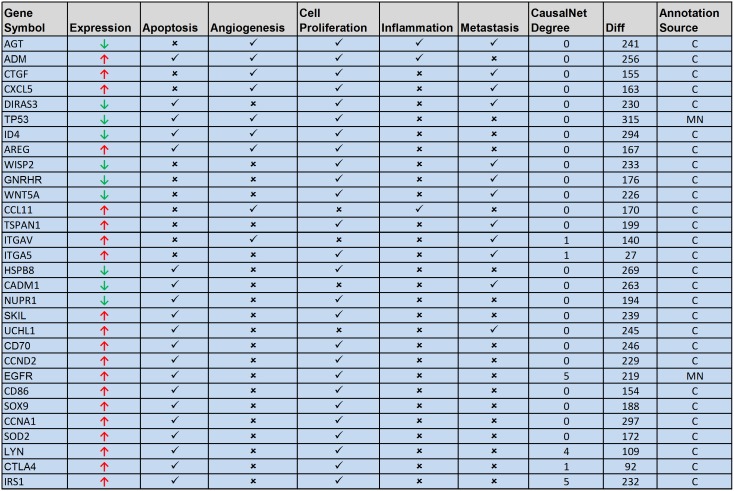
List of potential therapeutic targets for oral cancer. The right sign ‘✓’ represents significant publication evidence to support association of concerned target gene with a cancer hallmark mentioned in a concerned column, and ‘
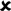
’ represents absence of such association between gene and cancer hallmark. The ‘
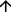
’ sign represents significant overexpression of the gene, and ‘
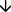
’ represents significant under-expression of the gene, observed in oral cancer in study dataset. ‘CausalNet Degree’ is the no. of causally connected genes to the particular target gene. ‘Diff’ is difference in the no. of connections in dependency network, under cancer and control condition for the concerned target gene. ‘MN’ means that annotations for the concerned target gene was inferred from articles related with mouth neoplasm or oral cancer, whereas ‘C’ means that annotations are not specific to oral cancer and were inferred using generic term ‘neoplasms’ or cancer.

Out of all significantly annotated genes, we found 554 genes to be associated with at-least one of the five cancer hallmarks considered in the current study. These genes were further subjected to filtering based on network statistics of dependency and causal network. Out of 554 genes, we identified 86 genes meeting various filtering criteria. We manually validated literature mining results (*_pub.txt files) of these 86 genes, to deal with issues related with ambiguous annotations. After thorough manual validation, we identified 30 genes, which can be targeted for therapeutic intervention in oral cancer (Fig. 7). After analyzing each of these therapeutic targets based on various criteria like number of associated cancer hallmarks, network connectivity statistics, supporting published literatures we identified 8 most promising therapeutic targets for oral cancer which are adrenomedullin (ADM), TP53, CTGF, EGFR, CTLA4, LYN, SKI-like oncogene (SKIL) and CD70. The list of therapeutic targets along with associated analysis data can be found in ‘OC_Targets.xls’ (see Text S12) available as online supplementary material.

ADM has been identified as a highly connected gene in the dependency network with marked difference under cancer and control condition. Literature mining analysis has identified it to be significantly associated with four out of the five cancer hallmarks considered in the current study. ADM is a research target for various cancers [38], and its significant differential expression in our study dataset suggests it to be one of the most potential therapeutic targets for oral cancer. TP53 is a potent tumor suppressor gene which is known to be under-expressed in various malignancies, including oral cancer [3]. TP53 was detected in our study to be significantly under expressed gene, and was found to be involved in key hallmark events like apoptosis, angiogenesis and cell proliferation. It was detected to be well connected gene with marked topological difference in the dependency network under cancer and control condition. The ability to regulate cancer via multiple pathways makes TP53 as one of the potential therapeutic targets for oral cancer. Literature mining analysis and mining of TTD [38] has identified TP53 as a therapeutic marker for various cancers including those of oral cavity [3]. Connectivity tissue growth factor (CTGF) was identified as a therapeutic target by literature mining analysis and was detected to be significantly involved in key hallmark events like angiogenesis and cell proliferation. CTGF shows marked topological difference in the dependency network under cancer and control condition making it one of the potential therapeutic targets for oral cancer. Epidermal growth factor receptor (EGFR) which is incidentally a successful molecular target for oral cancer [38], has been also detected as a potential therapeutic target in the current study. EGFR was identified as well connected gene in dependency and causal network (Fig. 5), and was detected as a significant hypothesis by causal reasoning analysis. CTLA4 was another potential therapeutic target identified in the current study. Literature mining analysis significantly associated it with apoptosis and cell-proliferation. CTLA4 has been reported to regulate key genes involved in carcinogenesis like STAT1, NFATC2, c-Fos, c-Myc, and/or Bcl-2 [39]. Literature mining analysis and mining of TTD have identified CTLA4 as a therapeutic marker for various cancers. CD70 was identified as a potential anti-body based therapeutic target. Literature mining analysis associated it with the key hallmark events like apoptosis and cell-proliferation. CD70 was detected to be topologically evolved gene by dependency network analysis, which has a significant number of connections in cancer condition, but does not have any connection in control condition. CD70 is a clinical trial target for various cancers [38]. LYN was identified in dependency network analysis as a topologically evolved gene, which has a significant number of connections in cancer condition, but does not have any connection in control condition. Literature mining analysis has associated it with apoptosis and cell-proliferation. It is also well connected in causal network, and was identified as one of the significant hypotheses. LYN has been reported in various studies to be an attractive therapeutic target for various cancers, including oral cancer [40]. SKIL has been identified in our analysis as highly connected gene in the dependency network with marked topological difference under cancer and control condition. Literature mining analysis associated it with apoptosis, cell-proliferation and metastasis. SKIL was reported to be a novel therapeutic target for ovarian cancer [41].

The analytical approach presented in the current study shows the power of direct integration of dataset generated by different studies to derive statistically significant results. The novel literature mining approach presented in the current study can be used for functional annotation of a gene-list produced by high-throughput studies related with cancer. The literature mining based functional classification comprehensively reviews published data, and has an advantage over traditional functional classification methods based on pathways or gene-sets, which does not represent the current state of art information since they are generally not updated quite often.

The current study has identified potential target genes for oral cancer. Some of the most potential therapeutic targets identified by our integrated analysis are adrenomedullin (ADM), TP53, CTGF, EGFR, CTLA4, LYN, SKI-like oncogene (SKIL) and CD70. The data presented here can also be used for identifying targets, which are specific to a particular cancer hallmark. The data presented could facilitate development of effective targeted therapies for oral cancer.

## Supporting Information

Text S1 **List of differentially expressed genes.** File contains following columns: (i) “Entrez GeneID”→Entrez GeneID of differentially expressed gene; (ii) “Symbol”→NCBI Gene Symbol of differentially expressed gene; (iii) “logFC”→Log Fold Change computed by limma; (iv) “P.Value”→p_value computed by limma; (v) “adj.P.Val”→fdr corrected p_value; (vi) “KEGG Pathways”→List of KEGG pathways mapped to the differentially expressed gene.
(TXT)

Text S2 **Comparison of the current study with the previous studies.** The datasets of two related studies - ‘Ambatipudi et al.’ and ‘Peng et al.’ were used for generating batch-corrected integrated dataset. The file contains comparison result of findings of current study with these two studies. File contains of two sheets Ambatipudi et al.’ and ‘Peng et al.’, in which differential expression reported in these studies is compared with findings of the current study.
(XLSX)

Text S3 **Connectivity information of genes computed by dependency network analysis.** File contains following columns: (i) “Symbol”→NCBI Gene Symbol; (ii) “Cancer_connections”→Total no. of significant connections in cancer condition; (iii) “Control_connections”→Total no. of significant connections in control condition; (iv) “Connectivity_diff”→ Difference in no. of connections between cancer and control condition; (v) “logFC”→Log Fold Change computed by limma; (vi) “adj.P.Val”→fdr corrected p_value for the gene computed by limma.
(TXT)

Text S4 **List of significant hypotheses identified by Causal Reasoning analysis.** Each row is a hypothesis (i.e. an entity in the graph with a direction of + or − and a number of downstream steps that are taken to predict transcripts). Each column is a “score”. This includes the name, number of correct, incorrect, and not explained transcripts as well as the correctness score and p-values defined in the manuscript.
(XLS)

Text S5 **Detailed list of hypotheses with corresponding downstream causally related genes.** File contains following columns: (i) Causal Hypothesis/Gene Name→Name of the gene which has been identified as significant hypothesis by causal reasoning analysis; (ii) Regulation→Relationship between hypothesis gene and downstream gene, ‘+’ represents activation and ‘−’ represents inhibition; (iii) Downstream Gene(s)→Downstream gene(s) whose differential expression is predicted by hypothesis gene; (iv) Prediction→‘C’ represents correctly predicted relationship between hypothesis and downstream gene, whereas ‘I’ represents otherwise. The succeeding +/− sign represents up−/down-regulation resp., of the downstream gene(s); (v) Source→The name of Kegg pathway(s) used to infer causal relationship between hypothesis & related downstream gene(s).
(DOCX)

Text S6 **Processed list of hypotheses and downstream genes.** File contains following columns: (i) Causal Hypothesis/Gene Name→Name of the gene which has been identified as significant hypothesis by causal reasoning analysis; (ii) Diff_Exp→The hypothesis genes which are differentially expressed in our analysis are marked as ‘DE’ and non-differentially expressed hypothesis genes are marked as ‘N’; (iii) Regulation→Relationship between hypothesis gene and downstream gene, ‘+’ represents activation and ‘−’ represents inhibition; (iv) Downstream Gene(s) →Downstream gene(s) whose differential expression is predicted by hypothesis gene; (v) Prediction→‘C’ represents correctly predicted relationship between hypothesis and downstream gene, whereas ‘I’ represents otherwise. The succeeding +/− sign represents up−/down-regulation resp., of the downstream gene(s); (vi) Source→The name of Kegg pathway(s) used to infer causal relationship between hypothesis & related downstream gene(s).
(DOCX)

Text S7 **Connectivity information of genes in Causal Network.** File contains connectivity information of gene(s) in causal network generated on the basis of causal relationships mentioned in Text S5. It contains following columns: (i) Symbol→NCBI gene symbol of a constituting node or gene of the causal network; (ii) Connectivity→Total no. of the neighboring directly connected genes based on causal relationship.
(DOCX)

Text S8 **Text mining results of successfully annotated genes.** This is a compressed file, which consist of the text mining results of successfully annotated genes by our method (available as <genesymbol> _pub.txt files specific to a particular gene). *_pub.txt file contains details about relevant articles used for annotation of concerned gene. It contains following columns: (i) PubmedID→Pubmed ID of the article; (ii) Marker→Binary flag where ‘0’ implies that article does not mention that concerned gene can be used as a marker, and ‘1’ implies that article supports the inference that concerned gene can be used as a marker; (iii) MarkerType→Comma delimited field, which represent marker type(s) mentioned for the gene in the article; (iv) CancerHallmark→Binary flag where ‘0’ implies that article does not mention that concerned gene is associated with cancer hallmarks, ‘1’ implies that article supports the inference that concerned gene is associated with cancer hallmarks; (v) HallmarkType→Comma delimited field, which represent cancer hallmarks(s) mentioned for the gene in the article; (vi) RelevantSentence→List of relevant sentence(s) from the article used for inferring marker type(s)/cancer hallmark(s).
(ZIP)

Text S9 **Statistics related with mining of PubMed articles.** File contains search statistics in following main columns: (i) GeneSymbol→NCBI gene symbol; (ii) DE Information (columns:LogFC.Adjusted_pvalue) →Differential expression data for the concerned gene; (iii) CancerType→Source of annotation for the concerned gene, ‘MN’ means that annotations for this gene was inferred from articles related with mouth neoplasm or oral cancer, whereas ‘C’ means that annotations are not specific to oral cancer and were inferred using generic term ‘neoplasms’ or cancer; (iv) TotalHits→Total no. of articles in PubMed satisfying the search criteria; (v) QualifiedHits→No. of articles which were considered to be relevant by text mining logic mentioned in the paper; (vi) Marker (columns:Therapeutic.Diagnostic) →Total no. of articles used to infer that a gene can be used as a particular marker type(s) (therapeutic/prognostic/diagnostic); (vii) Cancer Hallmark (columns:Angiogenesis.Inflammation)→Total no. of articles used to infer that a gene is associated with a particular cancer hallmark(s).
(DOCX)

Text S10 **Detailed report of all genes annotated by literature mining method.**
(DOCX)

Text S11 **Detailed report of significant annotations by literature mining method.**
(DOCX)

Text S12 **List of potential therapeutic targets for oral cancer.** Contains two sheets ‘TargetList’ and ‘IntegrativeAnalysis’. ‘TargetList’ contains list of therapeutic targets for oral cancer found to be most potential. This sheet contains following columns: (a) GeneName→NCBI Gene Symbol; (b) Annotation Source→Source of annotation for the concerned gene, ‘MN’ means that annotations for this gene was inferred from articles related with mouth neoplasm or oral cancer, whereas ‘C’ means that annotations are not specific to oral cancer and were inferred using generic term ‘neoplasms’ or cancer; (c) Connections_cancer→Total no. of significant connections in cancer condition detected by dependency network analysis; (d) Connections_control→Total no. of significant connections in control condition detected by dependency network analysis; (e) Diff→Difference between connections under cancer and control condition (i.e. Diff = Connections_cancer−Connections_control); (f) logFC→Log fold change value obtained from limma/differential expression analysis; (g) Adjusted p_value→Adjusted p_value obtained from limma/differential expression analysis; (h) Apoptosis→Right mark indicates that gene is associated with apoptosis, and cross mark indicates otherwise; (i) Angiogenesis→Right mark indicates that gene is associated with angiogenesis, and cross mark indicates otherwise; (j) CellProliferation→Right mark indicates that gene is associated with cell proliferation, and cross mark indicates otherwise; (k) Inflammation→Right mark indicates that gene is associated with inflammation, and cross mark indicates otherwise; (l) Metastasis→Right mark indicates that gene is associated with metastasis, and cross mark indicates otherwise; (m) TherapeuticTarget→Right mark indicates that gene is reported to be therapeutic target, and cross mark indicates otherwise; (n) CausalHypothesis→Right mark indicates that gene has been identified as significant hypothesis by causal reasoning, and cross mark indicates otherwise; (o) CausalNetGene→Right mark indicates that gene has been identified as downstream gene by causal reasoning, and cross mark indicates otherwise; (p) CausalNetDegree→Total no. of the neighboring directly connected genes based on causal relationship; (q) CausalPathway(s) →The name of Kegg pathway(s) used to infer causal relationships in which gene is involved; (r) TTD-TargetType→Classification of target inferred from TTD database; (s) TTD-TargetDiseases→List of disease(s) in which gene plays role of therapeutic target (inferred from TTD). ‘IntegrativeAnalysis’ contains initial list of candidate therapeutic target genes along with attributes used for identifying potential therapeutic genes.
(XLS)
