# Supplementary material for: Potential Therapeutic Targets for Oral Cancer: ADM, TP53, EGFR, LYN, CTLA4, SKIL, CTGF, CD70
Source: PLoS One. 2014 Jul 16;9(7):e102610. doi: 10.1371/journal.pone.0102610 (PMC4110113; doi:10.1371/journal.pone.0102610)
Supplement: Text S5 — Detailed list of hypotheses with corresponding downstream causally related genes. File contains following columns: (i) Causal Hypothesis/Gene Name→Name of the gene which has been identified as significant hypothesis by causal reasoning analysis; (ii) Regulation→Relationship between hypothesis gene and downstream gene, ‘+’ represents activation and ‘−’ represents inhibition; (iii) Downstream Gene(s)→Downstream gene(s) whose differential expression is predicted by hypothesis gene; (iv) Prediction→‘C’ represents correctly predicted relationship between hypothesis and downstream gene, whereas ‘I’ represents otherwise. The succeeding +/− sign represents up−/down-regulation resp., of the downstream gene(s); (v) Source→The name of Kegg pathway(s) used to infer causal relationship between hypothesis & related downstream gene(s). (DOCX) [file pone.0102610.s005.docx]

Causal Hypothesis/Gene Name Regulation Downstream Gene(s) Prediction Source

ANGPT1 + FGFR2 C- PI3K-Akt signaling pathway

ANGPT1 + MET I+ PI3K-Akt signaling pathway

ANGPT1 + EGFR I+ PI3K-Akt signaling pathway

ANGPT1 + TEK C- PI3K-Akt signaling pathway

ANGPT1 + KIT C- PI3K-Akt signaling pathway

ANGPT1 + FGFR3 C- PI3K-Akt signaling pathway

ANGPT4 + FGFR3 C- PI3K-Akt signaling pathway

ANGPT4 + KIT C- PI3K-Akt signaling pathway

ANGPT4 + TEK C- PI3K-Akt signaling pathway

ANGPT4 + MET I+ PI3K-Akt signaling pathway

ANGPT4 + EGFR I+ PI3K-Akt signaling pathway

ANGPT4 + FGFR2 C- PI3K-Akt signaling pathway

CALML3 - CNGB1 C+ Phototransduction

CALML3 + NRAS I+ Long-term potentiation

CALML3 + NOS1 C- Salivary secretion

CALML3 + CHP2 C- Long-term potentiation,Oocyte meiosis

CALML3 + PYGB I+ Insulin signaling pathway

CALML5 + NRAS I+ Long-term potentiation

CALML5 - CNGB1 C+ Phototransduction

CALML5 + NOS1 C- Salivary secretion

CALML5 + CHP2 C- Long-term potentiation,Oocyte meiosis

CALML5 + PYGB I+ Insulin signaling pathway

CALML6 + NOS1 C- Salivary secretion

CALML6 + NRAS I+ Long-term potentiation

CALML6 + CHP2 C- Long-term potentiation,Oocyte meiosis

CALML6 - CNGB1 C+ Phototransduction

CALML6 + PYGB I+ Insulin signaling pathway

CCL11 + CCR2 C- Chemokine signaling pathway

CCL11 + CXCR2 C- Chemokine signaling pathway

CCL11 + CX3CR1 C- Chemokine signaling pathway

CCL1 + CCR2 C- Chemokine signaling pathway

CCL1 + CX3CR1 C- Chemokine signaling pathway

CCL1 + CXCR2 C- Chemokine signaling pathway

CCL13 + CXCR2 C- Chemokine signaling pathway

CCL13 + CCR2 C- Cytokine-cytokine receptor interaction,Chemokine signaling pathway

CCL13 + CX3CR1 C- Chemokine signaling pathway

CCL14 + CX3CR1 C- Chemokine signaling pathway

CCL14 + CXCR2 C- Chemokine signaling pathway

CCL14 + CCR2 C- Chemokine signaling pathway

CCL15 + CXCR2 C- Chemokine signaling pathway

CCL15 + CX3CR1 C- Chemokine signaling pathway

CCL15 + CCR2 C- Chemokine signaling pathway

CCL16 + CCR2 C- Chemokine signaling pathway

CCL16 + CX3CR1 C- Chemokine signaling pathway

CCL16 + CXCR2 C- Chemokine signaling pathway

CCL17 + CXCR2 C- Chemokine signaling pathway

CCL17 + CX3CR1 C- Chemokine signaling pathway

CCL17 + CCR2 C- Chemokine signaling pathway

CCL18 + CX3CR1 C- Chemokine signaling pathway

CCL18 + CXCR2 C- Chemokine signaling pathway

CCL18 + CCR2 C- Chemokine signaling pathway

CCL19 + CCR2 C- Chemokine signaling pathway

CCL19 + CX3CR1 C- Chemokine signaling pathway

CCL19 + CXCR2 C- Chemokine signaling pathway

CCL20 + CX3CR1 C- Chemokine signaling pathway

CCL20 + CCR2 C- Chemokine signaling pathway

CCL20 + CXCR2 C- Chemokine signaling pathway

CCL21 + CXCR2 C- Chemokine signaling pathway

CCL21 + CX3CR1 C- Chemokine signaling pathway

CCL21 + CCR2 C- Chemokine signaling pathway

CCL2 + CX3CR1 C- Chemokine signaling pathway

CCL2 + CCR2 C- Cytokine-cytokine receptor interaction,Chemokine signaling pathway

CCL2 + CXCR2 C- Chemokine signaling pathway

CCL22 + CXCR2 C- Chemokine signaling pathway

CCL22 + CX3CR1 C- Chemokine signaling pathway

CCL22 + CCR2 C- Chemokine signaling pathway

CCL23 + CXCR2 C- Chemokine signaling pathway

CCL23 + CCR2 C- Chemokine signaling pathway

CCL23 + CX3CR1 C- Chemokine signaling pathway

CCL24 + CXCR2 C- Chemokine signaling pathway

CCL24 + CCR2 C- Chemokine signaling pathway

CCL24 + CX3CR1 C- Chemokine signaling pathway

CCL25 + CX3CR1 C- Chemokine signaling pathway

CCL25 + CCR2 C- Chemokine signaling pathway

CCL25 + CXCR2 C- Chemokine signaling pathway

CCL26 + CXCR2 C- Chemokine signaling pathway

CCL26 + CX3CR1 C- Chemokine signaling pathway

CCL26 + CCR2 C- Chemokine signaling pathway

CCL27 + CCR2 C- Chemokine signaling pathway

CCL27 + CX3CR1 C- Chemokine signaling pathway

CCL27 + CXCR2 C- Chemokine signaling pathway

CCL28 + CX3CR1 C- Chemokine signaling pathway

CCL28 + CCR2 C- Chemokine signaling pathway

CCL28 + CXCR2 C- Chemokine signaling pathway

CCL3 + CXCR2 C- Chemokine signaling pathway

CCL3 + CX3CR1 C- Chemokine signaling pathway

CCL3 + CCR2 C- Chemokine signaling pathway

CCL3L1 + CXCR2 C- Chemokine signaling pathway

CCL3L1 + CCR2 C- Chemokine signaling pathway

CCL3L1 + CX3CR1 C- Chemokine signaling pathway

CCL3L3 + CCR2 C- Chemokine signaling pathway

CCL3L3 + CX3CR1 C- Chemokine signaling pathway

CCL3L3 + CXCR2 C- Chemokine signaling pathway

CCL4 + CXCR2 C- Chemokine signaling pathway

CCL4 + CX3CR1 C- Chemokine signaling pathway

CCL4 + CCR2 C- Chemokine signaling pathway

CCL4L1 + CXCR2 C- Chemokine signaling pathway

CCL4L1 + CCR2 C- Chemokine signaling pathway

CCL4L1 + CX3CR1 C- Chemokine signaling pathway

CCL4L2 + CX3CR1 C- Chemokine signaling pathway

CCL4L2 + CXCR2 C- Chemokine signaling pathway

CCL4L2 + CCR2 C- Chemokine signaling pathway

CCL5 + CCR2 C- Chemokine signaling pathway

CCL5 + CXCR2 C- Chemokine signaling pathway

CCL5 + CX3CR1 C- Chemokine signaling pathway

CCL7 + CCR2 C- Cytokine-cytokine receptor interaction,Chemokine signaling pathway

CCL7 + CXCR2 C- Chemokine signaling pathway

CCL7 + CX3CR1 C- Chemokine signaling pathway

CCL8 + CXCR2 C- Chemokine signaling pathway

CCL8 + CX3CR1 C- Chemokine signaling pathway

CCL8 + CCR2 C- Chemokine signaling pathway

CD80 + CD274 C+ Cell adhesion molecules (CAMs)

CD80 + CTLA4 C+ Cell adhesion molecules (CAMs)

COL4A1 + SDC4 C+ ECM-receptor interaction

COL4A1 + CD44 C+ ECM-receptor interaction

COL4A2 + CD44 C+ ECM-receptor interaction

COL4A2 + SDC4 C+ ECM-receptor interaction

COL4A6 + CD44 C+ ECM-receptor interaction

COL4A6 + SDC4 C+ ECM-receptor interaction

COL5A2 + SDC4 C+ ECM-receptor interaction

COL5A2 + CD44 C+ ECM-receptor interaction

CRK + ABL2 C+ ErbB signaling pathway

CRK + PXN C+ Focal adhesion

CRK + RAPGEF1 C+ Renal cell carcinoma,Focal adhesion

CRKL + PXN C+ Focal adhesion

CRKL + ABL2 C+ ErbB signaling pathway

CRKL + RAPGEF1 C+ Renal cell carcinoma,Focal adhesion

CSF1 + EGFR I+ PI3K-Akt signaling pathway

CSF1 + FGFR3 C- PI3K-Akt signaling pathway

CSF1 + KIT C- PI3K-Akt signaling pathway

CSF1 + TEK C- PI3K-Akt signaling pathway

CSF1 + FGFR2 C- PI3K-Akt signaling pathway

CSF1 + MET I+ PI3K-Akt signaling pathway

CX3CL1 + CCR2 C- Chemokine signaling pathway

CX3CL1 + CX3CR1 C- Cytokine-cytokine receptor interaction,Chemokine signaling pathway

CX3CL1 + CXCR2 C- Chemokine signaling pathway

CXCL10 + CXCR2 C- Chemokine signaling pathway

CXCL10 + CCR2 C- Chemokine signaling pathway

CXCL10 + CX3CR1 C- Chemokine signaling pathway

CXCL11 + CCR2 C- Chemokine signaling pathway

CXCL11 + CXCR2 C- Chemokine signaling pathway

CXCL11 + CX3CR1 C- Chemokine signaling pathway

CXCL1 + CX3CR1 C- Chemokine signaling pathway

CXCL1 + CCR2 C- Chemokine signaling pathway

CXCL1 + CXCR2 C- Cytokine-cytokine receptor interaction,Chemokine signaling pathway

CXCL12 + CXCR2 C- Chemokine signaling pathway

CXCL12 + CX3CR1 C- Chemokine signaling pathway

CXCL12 + CCR2 C- Chemokine signaling pathway

CXCL13 + CX3CR1 C- Chemokine signaling pathway

CXCL13 + CXCR2 C- Chemokine signaling pathway

CXCL13 + CCR2 C- Chemokine signaling pathway

CXCL14 + CXCR2 C- Chemokine signaling pathway

CXCL14 + CCR2 C- Chemokine signaling pathway

CXCL14 + CX3CR1 C- Chemokine signaling pathway

CXCL16 + CX3CR1 C- Chemokine signaling pathway

CXCL16 + CXCR2 C- Chemokine signaling pathway

CXCL16 + CCR2 C- Chemokine signaling pathway

CXCL2 + CXCR2 C- Cytokine-cytokine receptor interaction,Chemokine signaling pathway

CXCL2 + CX3CR1 C- Chemokine signaling pathway

CXCL2 + CCR2 C- Chemokine signaling pathway

CXCL3 + CXCR2 C- Cytokine-cytokine receptor interaction,Chemokine signaling pathway

CXCL3 + CCR2 C- Chemokine signaling pathway

CXCL3 + CX3CR1 C- Chemokine signaling pathway

CXCL5 + CCR2 C- Chemokine signaling pathway

CXCL5 + CXCR2 C- Cytokine-cytokine receptor interaction,Chemokine signaling pathway

CXCL5 + CX3CR1 C- Chemokine signaling pathway

CXCL6 + CXCR2 C- Cytokine-cytokine receptor interaction,Chemokine signaling pathway

CXCL6 + CCR2 C- Chemokine signaling pathway

CXCL6 + CX3CR1 C- Chemokine signaling pathway

CXCL9 + CXCR2 C- Chemokine signaling pathway

CXCL9 + CCR2 C- Chemokine signaling pathway

CXCL9 + CX3CR1 C- Chemokine signaling pathway

DUSP4 - MAPK3 C- MAPK signaling pathway

DUSP4 - MAPK13 C- MAPK signaling pathway

DUSP6 - MAPK3 C- MAPK signaling pathway

DUSP6 - MAPK13 C- MAPK signaling pathway

EFNA1 + KIT C- PI3K-Akt signaling pathway

EFNA1 + FGFR3 C- PI3K-Akt signaling pathway

EFNA1 + MET I+ PI3K-Akt signaling pathway

EFNA1 + EGFR I+ PI3K-Akt signaling pathway

EFNA1 + FGFR2 C- PI3K-Akt signaling pathway

EFNA1 + TEK C- PI3K-Akt signaling pathway

EFNA1 + EPHA7 C- Axon guidance

EFNA2 + EGFR I+ PI3K-Akt signaling pathway

EFNA2 + FGFR3 C- PI3K-Akt signaling pathway

EFNA2 + TEK C- PI3K-Akt signaling pathway

EFNA2 + MET I+ PI3K-Akt signaling pathway

EFNA2 + KIT C- PI3K-Akt signaling pathway

EFNA2 + FGFR2 C- PI3K-Akt signaling pathway

EFNA2 + EPHA7 C- Axon guidance

EFNA3 + FGFR3 C- PI3K-Akt signaling pathway

EFNA3 + KIT C- PI3K-Akt signaling pathway

EFNA3 + TEK C- PI3K-Akt signaling pathway

EFNA3 + EGFR I+ PI3K-Akt signaling pathway

EFNA3 + MET I+ PI3K-Akt signaling pathway

EFNA3 + FGFR2 C- PI3K-Akt signaling pathway

EFNA3 + EPHA7 C- Axon guidance

EFNA4 + FGFR2 C- PI3K-Akt signaling pathway

EFNA4 + KIT C- PI3K-Akt signaling pathway

EFNA4 + MET I+ PI3K-Akt signaling pathway

EFNA4 + EPHA7 C- Axon guidance

EFNA4 + FGFR3 C- PI3K-Akt signaling pathway

EFNA4 + TEK C- PI3K-Akt signaling pathway

EFNA4 + EGFR I+ PI3K-Akt signaling pathway

EFNA5 + EGFR I+ PI3K-Akt signaling pathway

EFNA5 + FGFR2 C- PI3K-Akt signaling pathway

EFNA5 + MET I+ PI3K-Akt signaling pathway

EFNA5 + TEK C- PI3K-Akt signaling pathway

EFNA5 + KIT C- PI3K-Akt signaling pathway

EFNA5 + EPHA7 C- Axon guidance

EFNA5 + FGFR3 C- PI3K-Akt signaling pathway

EGF + MET I+ Melanoma,PI3K-Akt signaling pathway,Focal adhesion

EGF + FGFR3 C- PI3K-Akt signaling pathway

EGF + TEK C- PI3K-Akt signaling pathway

EGF + EGFR I+ Gap junction,Non-small cell lung cancer,ErbB signaling pathway,MAPK signaling pathway,Melanoma,Endometrial cancer,Bladder cancer,Cytokine-cytokine receptor interaction,Glioma,Glioma,Prostate cancer,PI3K-Akt signaling pathway,Focal adhesion,HIF-1 signaling pathway,Pathways in cancer

EGF + FGFR2 C- Prostate cancer,PI3K-Akt signaling pathway

EGF + KIT C- PI3K-Akt signaling pathway

EGFR + MAPK3 I- Proteoglycans in cancer,Proteoglycans in cancer

EGFR + PTK2 C+ Focal adhesion

EGFR + IRS1 C+ PI3K-Akt signaling pathway

EGFR + NRAS C+ Melanoma

EGFR + SHC1 C+ ErbB signaling pathway,ErbB signaling pathway,Estrogen signaling pathway,Glioma,Glioma

FGF10 + KIT C- PI3K-Akt signaling pathway

FGF10 + FGFR2 C- MAPK signaling pathway,PI3K-Akt signaling pathway,Pathways in cancer

FGF10 + EGFR I+ Melanoma,PI3K-Akt signaling pathway

FGF10 + MET I+ Melanoma,PI3K-Akt signaling pathway

FGF10 + FGFR3 C- MAPK signaling pathway,PI3K-Akt signaling pathway,Pathways in cancer

FGF10 + TEK C- PI3K-Akt signaling pathway

FGF11 + TEK C- PI3K-Akt signaling pathway

FGF11 + FGFR2 C- MAPK signaling pathway,PI3K-Akt signaling pathway,Pathways in cancer

FGF11 + KIT C- PI3K-Akt signaling pathway

FGF11 + EGFR I+ Melanoma,PI3K-Akt signaling pathway

FGF11 + FGFR3 C- MAPK signaling pathway,PI3K-Akt signaling pathway,Pathways in cancer

FGF11 + MET I+ Melanoma,PI3K-Akt signaling pathway

FGF1 + MET I+ Melanoma,PI3K-Akt signaling pathway

FGF1 + FGFR2 C- MAPK signaling pathway,PI3K-Akt signaling pathway,Pathways in cancer

FGF1 + EGFR I+ Melanoma,PI3K-Akt signaling pathway

FGF1 + TEK C- PI3K-Akt signaling pathway

FGF1 + FGFR3 C- MAPK signaling pathway,PI3K-Akt signaling pathway,Pathways in cancer

FGF1 + KIT C- PI3K-Akt signaling pathway

FGF12 + FGFR2 C- MAPK signaling pathway,PI3K-Akt signaling pathway,Pathways in cancer

FGF12 + FGFR3 C- MAPK signaling pathway,PI3K-Akt signaling pathway,Pathways in cancer

FGF12 + KIT C- PI3K-Akt signaling pathway

FGF12 + TEK C- PI3K-Akt signaling pathway

FGF12 + EGFR I+ Melanoma,PI3K-Akt signaling pathway

FGF12 + MET I+ Melanoma,PI3K-Akt signaling pathway

FGF13 + MET I+ Melanoma,PI3K-Akt signaling pathway

FGF13 + KIT C- PI3K-Akt signaling pathway

FGF13 + TEK C- PI3K-Akt signaling pathway

FGF13 + EGFR I+ Melanoma,PI3K-Akt signaling pathway

FGF13 + FGFR3 C- MAPK signaling pathway,PI3K-Akt signaling pathway,Pathways in cancer

FGF13 + FGFR2 C- MAPK signaling pathway,PI3K-Akt signaling pathway,Pathways in cancer

FGF14 + KIT C- PI3K-Akt signaling pathway

FGF14 + EGFR I+ Melanoma,PI3K-Akt signaling pathway

FGF14 + FGFR2 C- MAPK signaling pathway,PI3K-Akt signaling pathway,Pathways in cancer

FGF14 + MET I+ Melanoma,PI3K-Akt signaling pathway

FGF14 + TEK C- PI3K-Akt signaling pathway

FGF14 + FGFR3 C- MAPK signaling pathway,PI3K-Akt signaling pathway,Pathways in cancer

FGF16 + KIT C- PI3K-Akt signaling pathway

FGF16 + MET I+ Melanoma,PI3K-Akt signaling pathway

FGF16 + FGFR2 C- MAPK signaling pathway,PI3K-Akt signaling pathway,Pathways in cancer

FGF16 + EGFR I+ Melanoma,PI3K-Akt signaling pathway

FGF16 + FGFR3 C- MAPK signaling pathway,PI3K-Akt signaling pathway,Pathways in cancer

FGF16 + TEK C- PI3K-Akt signaling pathway

FGF17 + FGFR2 C- MAPK signaling pathway,PI3K-Akt signaling pathway,Pathways in cancer

FGF17 + EGFR I+ Melanoma,PI3K-Akt signaling pathway

FGF17 + TEK C- PI3K-Akt signaling pathway

FGF17 + MET I+ Melanoma,PI3K-Akt signaling pathway

FGF17 + KIT C- PI3K-Akt signaling pathway

FGF17 + FGFR3 C- MAPK signaling pathway,PI3K-Akt signaling pathway,Pathways in cancer

FGF18 + FGFR3 C- MAPK signaling pathway,PI3K-Akt signaling pathway,Pathways in cancer

FGF18 + KIT C- PI3K-Akt signaling pathway

FGF18 + EGFR I+ Melanoma,PI3K-Akt signaling pathway

FGF18 + FGFR2 C- MAPK signaling pathway,PI3K-Akt signaling pathway,Pathways in cancer

FGF18 + TEK C- PI3K-Akt signaling pathway

FGF18 + MET I+ Melanoma,PI3K-Akt signaling pathway

FGF19 + FGFR2 C- MAPK signaling pathway,PI3K-Akt signaling pathway,Pathways in cancer

FGF19 + KIT C- PI3K-Akt signaling pathway

FGF19 + FGFR3 C- MAPK signaling pathway,PI3K-Akt signaling pathway,Pathways in cancer

FGF19 + MET I+ Melanoma,PI3K-Akt signaling pathway

FGF19 + TEK C- PI3K-Akt signaling pathway

FGF19 + EGFR I+ Melanoma,PI3K-Akt signaling pathway

FGF20 + FGFR2 C- MAPK signaling pathway,PI3K-Akt signaling pathway,Pathways in cancer

FGF20 + KIT C- PI3K-Akt signaling pathway

FGF20 + FGFR3 C- MAPK signaling pathway,PI3K-Akt signaling pathway,Pathways in cancer

FGF20 + EGFR I+ Melanoma,PI3K-Akt signaling pathway

FGF20 + MET I+ Melanoma,PI3K-Akt signaling pathway

FGF20 + TEK C- PI3K-Akt signaling pathway

FGF21 + FGFR3 C- MAPK signaling pathway,PI3K-Akt signaling pathway,Pathways in cancer

FGF21 + TEK C- PI3K-Akt signaling pathway

FGF21 + KIT C- PI3K-Akt signaling pathway

FGF21 + EGFR I+ Melanoma,PI3K-Akt signaling pathway

FGF21 + FGFR2 C- MAPK signaling pathway,PI3K-Akt signaling pathway,Pathways in cancer

FGF21 + MET I+ Melanoma,PI3K-Akt signaling pathway

FGF2 + KIT C- PI3K-Akt signaling pathway

FGF2 + FGFR3 C- MAPK signaling pathway,PI3K-Akt signaling pathway,Pathways in cancer

FGF2 + TEK C- PI3K-Akt signaling pathway

FGF2 + MET I+ Melanoma,PI3K-Akt signaling pathway

FGF2 + FGFR2 C- MAPK signaling pathway,PI3K-Akt signaling pathway,Pathways in cancer

FGF2 + EGFR I+ Melanoma,PI3K-Akt signaling pathway

FGF22 + KIT C- PI3K-Akt signaling pathway

FGF22 + FGFR3 C- MAPK signaling pathway,PI3K-Akt signaling pathway,Pathways in cancer

FGF22 + TEK C- PI3K-Akt signaling pathway

FGF22 + EGFR I+ Melanoma,PI3K-Akt signaling pathway

FGF22 + MET I+ Melanoma,PI3K-Akt signaling pathway

FGF22 + FGFR2 C- MAPK signaling pathway,PI3K-Akt signaling pathway,Pathways in cancer

FGF23 + FGFR2 C- MAPK signaling pathway,PI3K-Akt signaling pathway,Pathways in cancer

FGF23 + TEK C- PI3K-Akt signaling pathway

FGF23 + FGFR3 C- MAPK signaling pathway,PI3K-Akt signaling pathway,Pathways in cancer

FGF23 + KIT C- PI3K-Akt signaling pathway

FGF23 + MET I+ Melanoma,PI3K-Akt signaling pathway

FGF23 + EGFR I+ Melanoma,PI3K-Akt signaling pathway

FGF3 + FGFR3 C- MAPK signaling pathway,PI3K-Akt signaling pathway,Pathways in cancer

FGF3 + MET I+ Melanoma,PI3K-Akt signaling pathway

FGF3 + EGFR I+ Melanoma,PI3K-Akt signaling pathway

FGF3 + FGFR2 C- MAPK signaling pathway,PI3K-Akt signaling pathway,Pathways in cancer

FGF3 + TEK C- PI3K-Akt signaling pathway

FGF3 + KIT C- PI3K-Akt signaling pathway

FGF4 + FGFR2 C- MAPK signaling pathway,PI3K-Akt signaling pathway,Pathways in cancer

FGF4 + FGFR3 C- MAPK signaling pathway,PI3K-Akt signaling pathway,Pathways in cancer

FGF4 + TEK C- PI3K-Akt signaling pathway

FGF4 + EGFR I+ Melanoma,PI3K-Akt signaling pathway

FGF4 + MET I+ Melanoma,PI3K-Akt signaling pathway

FGF4 + KIT C- PI3K-Akt signaling pathway

FGF5 + TEK C- PI3K-Akt signaling pathway

FGF5 + FGFR2 C- MAPK signaling pathway,PI3K-Akt signaling pathway,Pathways in cancer

FGF5 + KIT C- PI3K-Akt signaling pathway

FGF5 + EGFR I+ Melanoma,PI3K-Akt signaling pathway

FGF5 + MET I+ Melanoma,PI3K-Akt signaling pathway

FGF5 + FGFR3 C- MAPK signaling pathway,PI3K-Akt signaling pathway,Pathways in cancer

FGF6 + FGFR3 C- MAPK signaling pathway,PI3K-Akt signaling pathway,Pathways in cancer

FGF6 + KIT C- PI3K-Akt signaling pathway

FGF6 + MET I+ Melanoma,PI3K-Akt signaling pathway

FGF6 + TEK C- PI3K-Akt signaling pathway

FGF6 + EGFR I+ Melanoma,PI3K-Akt signaling pathway

FGF6 + FGFR2 C- MAPK signaling pathway,PI3K-Akt signaling pathway,Pathways in cancer

FGF7 + TEK C- PI3K-Akt signaling pathway

FGF7 + KIT C- PI3K-Akt signaling pathway

FGF7 + FGFR3 C- MAPK signaling pathway,PI3K-Akt signaling pathway,Pathways in cancer

FGF7 + MET I+ Melanoma,PI3K-Akt signaling pathway

FGF7 + FGFR2 C- MAPK signaling pathway,PI3K-Akt signaling pathway,Pathways in cancer

FGF7 + EGFR I+ Melanoma,PI3K-Akt signaling pathway

FGF8 + FGFR2 C- MAPK signaling pathway,PI3K-Akt signaling pathway,Pathways in cancer

FGF8 + FGFR3 C- MAPK signaling pathway,PI3K-Akt signaling pathway,Pathways in cancer

FGF8 + TEK C- PI3K-Akt signaling pathway

FGF8 + MET I+ Melanoma,PI3K-Akt signaling pathway

FGF8 + KIT C- PI3K-Akt signaling pathway

FGF8 + EGFR I+ Melanoma,PI3K-Akt signaling pathway

FGF9 + TEK C- PI3K-Akt signaling pathway

FGF9 + MET I+ Melanoma,PI3K-Akt signaling pathway

FGF9 + KIT C- PI3K-Akt signaling pathway

FGF9 + FGFR3 C- MAPK signaling pathway,PI3K-Akt signaling pathway,Pathways in cancer

FGF9 + FGFR2 C- MAPK signaling pathway,PI3K-Akt signaling pathway,Pathways in cancer

FGF9 + EGFR I+ Melanoma,PI3K-Akt signaling pathway

FIGF + FGFR2 C- PI3K-Akt signaling pathway

FIGF + FGFR3 C- PI3K-Akt signaling pathway

FIGF + EGFR I+ PI3K-Akt signaling pathway,Focal adhesion

FIGF + KIT C- PI3K-Akt signaling pathway

FIGF + MET I+ PI3K-Akt signaling pathway,Focal adhesion

FIGF + TEK C- PI3K-Akt signaling pathway

FLT1 + SHC1 C+ Focal adhesion

FLT1 + IRS1 C+ PI3K-Akt signaling pathway

FLT1 + PTK2 C+ Focal adhesion

FLT4 + IRS1 C+ PI3K-Akt signaling pathway

FLT4 + PTK2 C+ Focal adhesion

FLT4 + SHC1 C+ Focal adhesion

FN1 + ITGAV C+ Proteoglycans in cancer

FN1 + ITGA5 C+ Proteoglycans in cancer,Proteoglycans in cancer

FN1 + SDC4 C+ Proteoglycans in cancer,ECM-receptor interaction

FN1 + CD44 C+ ECM-receptor interaction

FN1 + ITGB1 C+ Proteoglycans in cancer,Proteoglycans in cancer

GNA11 + JMJD7-PLA2G4B C- Vascular smooth muscle contraction

GNA11 + PLA2G4F C- Vascular smooth muscle contraction

GNA11 + PLA2G3 C- Vascular smooth muscle contraction

GNA11 + PLCB4 C- GnRH signaling pathway,Gap junction,Cholinergic synapse,Insulin secretion,Long-term depression,Long-term depression,Vascular smooth muscle contraction,Calcium signaling pathway

GNA11 + PLA2G1B C- Vascular smooth muscle contraction

GNAI1 + PLA2G4F C- Long-term depression,Long-term depression

GNAI1 + MAPK13 C- Retrograde endocannabinoid signaling,Retrograde endocannabinoid signaling

GNAI1 + JMJD7-PLA2G4B C- Long-term depression,Long-term depression

GNAI1 + NRAS I+ Gap junction

GNAI1 - ADCY6 I- Gap junction,Retrograde endocannabinoid signaling,Retrograde endocannabinoid signaling,GABAergic synapse,GABAergic synapse,Chemokine signaling pathway,Progesterone-mediated oocyte maturation,Cholinergic synapse,Gastric acid secretion,Glutamatergic synapse,Glutamatergic synapse

GNAI1 + MAPK3 C- Retrograde endocannabinoid signaling,Retrograde endocannabinoid signaling

GNAI1 + PLCB4 C- Serotonergic synapse,Dopaminergic synapse,Melanogenesis

GNAI1 + LYN I+ Chemokine signaling pathway

GNAI2 + PLCB4 C- Serotonergic synapse,Dopaminergic synapse,Melanogenesis

GNAI2 + MAPK3 C- Retrograde endocannabinoid signaling,Retrograde endocannabinoid signaling

GNAI2 + MAPK13 C- Retrograde endocannabinoid signaling,Retrograde endocannabinoid signaling

GNAI2 + JMJD7-PLA2G4B C- Long-term depression,Long-term depression

GNAI2 + LYN I+ Chemokine signaling pathway

GNAI2 + NRAS I+ Gap junction

GNAI2 - ADCY6 I- Gap junction,Retrograde endocannabinoid signaling,Retrograde endocannabinoid signaling,GABAergic synapse,GABAergic synapse,Chemokine signaling pathway,Progesterone-mediated oocyte maturation,Cholinergic synapse,Gastric acid secretion,Glutamatergic synapse,Glutamatergic synapse

GNAI2 + PLA2G4F C- Long-term depression,Long-term depression

GNAI3 - ADCY6 I- Gap junction,Retrograde endocannabinoid signaling,Retrograde endocannabinoid signaling,GABAergic synapse,GABAergic synapse,Chemokine signaling pathway,Progesterone-mediated oocyte maturation,Cholinergic synapse,Gastric acid secretion,Glutamatergic synapse,Glutamatergic synapse

GNAI3 + MAPK13 C- Retrograde endocannabinoid signaling,Retrograde endocannabinoid signaling

GNAI3 + PLCB4 C- Serotonergic synapse,Dopaminergic synapse,Melanogenesis

GNAI3 + PLA2G4F C- Long-term depression,Long-term depression

GNAI3 + JMJD7-PLA2G4B C- Long-term depression,Long-term depression

GNAI3 + NRAS I+ Gap junction

GNAI3 + LYN I+ Chemokine signaling pathway

GNAI3 + MAPK3 C- Retrograde endocannabinoid signaling,Retrograde endocannabinoid signaling

GNAO1 + PLCB4 C- Serotonergic synapse,Dopaminergic synapse,Melanogenesis

GNAO1 + MAPK13 C- Retrograde endocannabinoid signaling,Retrograde endocannabinoid signaling

GNAO1 - ADCY6 I- Retrograde endocannabinoid signaling,Retrograde endocannabinoid signaling,GABAergic synapse,GABAergic synapse,Cholinergic synapse,Glutamatergic synapse,Glutamatergic synapse

GNAO1 + PLA2G4F C- Long-term depression,Long-term depression

GNAO1 + MAPK3 C- Retrograde endocannabinoid signaling,Retrograde endocannabinoid signaling

GNAO1 + JMJD7-PLA2G4B C- Long-term depression,Long-term depression

GNAQ + PLCB4 C- GnRH signaling pathway,Gap junction,Retrograde endocannabinoid signaling,Estrogen signaling pathway,Long-term potentiation,Serotonergic synapse,Serotonergic synapse,Cholinergic synapse,Salivary secretion,Insulin secretion,Pancreatic secretion,Long-term depression,Long-term depression,Gastric acid secretion,Dopaminergic synapse,Vascular smooth muscle contraction,Glutamatergic synapse,Calcium signaling pathway

GNAQ + PLA2G1B C- Vascular smooth muscle contraction

GNAQ + PLA2G3 C- Vascular smooth muscle contraction

GNAQ + JMJD7-PLA2G4B C- Serotonergic synapse,Vascular smooth muscle contraction

GNAQ + PLA2G4F C- Serotonergic synapse,Vascular smooth muscle contraction

GNAS + ADCY6 C- GnRH signaling pathway,Bile secretion,Ovarian steroidogenesis,Ovarian steroidogenesis,Gap junction,Vasopressin-regulated water reabsorption,Estrogen signaling pathway,Salivary secretion,Insulin secretion,Pancreatic secretion,Gastric acid secretion,Vascular smooth muscle contraction,Glutamatergic synapse,Melanogenesis

GNAS + PLA2G4F C- Ovarian steroidogenesis,Long-term depression,Long-term depression

GNAS + JMJD7-PLA2G4B C- Ovarian steroidogenesis,Long-term depression,Long-term depression

GNAZ + JMJD7-PLA2G4B C- Long-term depression,Long-term depression

GNAZ + PLA2G4F C- Long-term depression,Long-term depression

GNB1 - ADCY6 A- Retrograde endocannabinoid signaling,Retrograde endocannabinoid signaling,GABAergic synapse,GABAergic synapse,Cholinergic synapse,Glutamatergic synapse

GNB1 + PDE6G C- Phototransduction

GNB1 + MAPK13 C- Retrograde endocannabinoid signaling,Retrograde endocannabinoid signaling

GNB1 + MAPK3 C- Retrograde endocannabinoid signaling,Retrograde endocannabinoid signaling

GNB1 + PDE6A C- Phototransduction

GNB1 + PLCB4 C- Serotonergic synapse,Chemokine signaling pathway,Dopaminergic synapse

GNB2 + MAPK3 C- Retrograde endocannabinoid signaling,Retrograde endocannabinoid signaling

GNB2 + MAPK13 C- Retrograde endocannabinoid signaling,Retrograde endocannabinoid signaling

GNB2 - ADCY6 I- Retrograde endocannabinoid signaling,Retrograde endocannabinoid signaling,GABAergic synapse,GABAergic synapse,Cholinergic synapse,Glutamatergic synapse

GNB2 + PLCB4 C- Serotonergic synapse,Chemokine signaling pathway,Dopaminergic synapse

GNB3 + MAPK3 C- Retrograde endocannabinoid signaling,Retrograde endocannabinoid signaling

GNB3 - ADCY6 A- Retrograde endocannabinoid signaling,Retrograde endocannabinoid signaling,GABAergic synapse,GABAergic synapse,Cholinergic synapse,Glutamatergic synapse

GNB3 + MAPK13 C- Retrograde endocannabinoid signaling,Retrograde endocannabinoid signaling

GNB3 + PLCB4 C- Serotonergic synapse,Chemokine signaling pathway,Dopaminergic synapse

GNB4 + MAPK3 C- Retrograde endocannabinoid signaling,Retrograde endocannabinoid signaling

GNB4 + PLCB4 C- Serotonergic synapse,Chemokine signaling pathway,Dopaminergic synapse

GNB4 + MAPK13 C- Retrograde endocannabinoid signaling,Retrograde endocannabinoid signaling

GNB4 - ADCY6 I- Retrograde endocannabinoid signaling,Retrograde endocannabinoid signaling,GABAergic synapse,GABAergic synapse,Cholinergic synapse,Glutamatergic synapse

GNB5 + MAPK13 C- Retrograde endocannabinoid signaling,Retrograde endocannabinoid signaling

GNB5 - ADCY6 I- Retrograde endocannabinoid signaling,Retrograde endocannabinoid signaling,GABAergic synapse,GABAergic synapse,Cholinergic synapse,Glutamatergic synapse

GNB5 + PLCB4 C- Serotonergic synapse,Chemokine signaling pathway,Dopaminergic synapse

GNB5 + MAPK3 C- Retrograde endocannabinoid signaling,Retrograde endocannabinoid signaling

GNG10 + PLCB4 C- Serotonergic synapse,Chemokine signaling pathway,Dopaminergic synapse

GNG10 + MAPK13 C- Retrograde endocannabinoid signaling,Retrograde endocannabinoid signaling

GNG10 - ADCY6 I- Retrograde endocannabinoid signaling,Retrograde endocannabinoid signaling,GABAergic synapse,GABAergic synapse,Cholinergic synapse,Glutamatergic synapse

GNG10 + MAPK3 C- Retrograde endocannabinoid signaling,Retrograde endocannabinoid signaling

GNG11 - ADCY6 I- Retrograde endocannabinoid signaling,Retrograde endocannabinoid signaling,GABAergic synapse,GABAergic synapse,Cholinergic synapse,Glutamatergic synapse

GNG11 + MAPK3 C- Retrograde endocannabinoid signaling,Retrograde endocannabinoid signaling

GNG11 + PLCB4 C- Serotonergic synapse,Chemokine signaling pathway,Dopaminergic synapse

GNG11 + MAPK13 C- Retrograde endocannabinoid signaling,Retrograde endocannabinoid signaling

GNG13 + MAPK13 C- Retrograde endocannabinoid signaling,Retrograde endocannabinoid signaling

GNG13 + MAPK3 C- Retrograde endocannabinoid signaling,Retrograde endocannabinoid signaling

GNG13 - ADCY6 A- Retrograde endocannabinoid signaling,Retrograde endocannabinoid signaling,GABAergic synapse,GABAergic synapse,Cholinergic synapse,Glutamatergic synapse

GNG13 + PLCB4 C- Serotonergic synapse,Chemokine signaling pathway,Dopaminergic synapse

GNG2 + MAPK13 C- Retrograde endocannabinoid signaling,Retrograde endocannabinoid signaling

GNG2 - ADCY6 I- Retrograde endocannabinoid signaling,Retrograde endocannabinoid signaling,GABAergic synapse,GABAergic synapse,Cholinergic synapse,Glutamatergic synapse

GNG2 + PLCB4 C- Serotonergic synapse,Chemokine signaling pathway,Dopaminergic synapse

GNG2 + MAPK3 C- Retrograde endocannabinoid signaling,Retrograde endocannabinoid signaling

GNG3 + MAPK3 C- Retrograde endocannabinoid signaling,Retrograde endocannabinoid signaling

GNG3 + MAPK13 C- Retrograde endocannabinoid signaling,Retrograde endocannabinoid signaling

GNG3 + PLCB4 C- Serotonergic synapse,Chemokine signaling pathway,Dopaminergic synapse

GNG3 - ADCY6 A- Retrograde endocannabinoid signaling,Retrograde endocannabinoid signaling,GABAergic synapse,GABAergic synapse,Cholinergic synapse,Glutamatergic synapse

GNG4 - ADCY6 I- Retrograde endocannabinoid signaling,Retrograde endocannabinoid signaling,GABAergic synapse,GABAergic synapse,Cholinergic synapse,Glutamatergic synapse

GNG4 + PLCB4 C- Serotonergic synapse,Chemokine signaling pathway,Dopaminergic synapse

GNG4 + MAPK3 C- Retrograde endocannabinoid signaling,Retrograde endocannabinoid signaling

GNG4 + MAPK13 C- Retrograde endocannabinoid signaling,Retrograde endocannabinoid signaling

GNG5 + PLCB4 C- Serotonergic synapse,Chemokine signaling pathway,Dopaminergic synapse

GNG5 + MAPK13 C- Retrograde endocannabinoid signaling,Retrograde endocannabinoid signaling

GNG5 + MAPK3 C- Retrograde endocannabinoid signaling,Retrograde endocannabinoid signaling

GNG5 - ADCY6 I- Retrograde endocannabinoid signaling,Retrograde endocannabinoid signaling,GABAergic synapse,GABAergic synapse,Cholinergic synapse,Glutamatergic synapse

GNG7 + MAPK13 C- Retrograde endocannabinoid signaling,Retrograde endocannabinoid signaling

GNG7 + MAPK3 C- Retrograde endocannabinoid signaling,Retrograde endocannabinoid signaling

GNG7 + PLCB4 C- Serotonergic synapse,Chemokine signaling pathway,Dopaminergic synapse

GNG7 - ADCY6 I- Retrograde endocannabinoid signaling,Retrograde endocannabinoid signaling,GABAergic synapse,GABAergic synapse,Cholinergic synapse,Glutamatergic synapse

GNG8 + MAPK13 C- Retrograde endocannabinoid signaling,Retrograde endocannabinoid signaling

GNG8 - ADCY6 I- Retrograde endocannabinoid signaling,Retrograde endocannabinoid signaling,GABAergic synapse,GABAergic synapse,Cholinergic synapse,Glutamatergic synapse

GNG8 + PLCB4 C- Serotonergic synapse,Chemokine signaling pathway,Dopaminergic synapse

GNG8 + MAPK3 C- Retrograde endocannabinoid signaling,Retrograde endocannabinoid signaling

GNGT1 + PDE6G C- Phototransduction

GNGT1 + MAPK3 C- Retrograde endocannabinoid signaling,Retrograde endocannabinoid signaling

GNGT1 - ADCY6 I- Retrograde endocannabinoid signaling,Retrograde endocannabinoid signaling,GABAergic synapse,GABAergic synapse,Cholinergic synapse,Glutamatergic synapse

GNGT1 + PDE6A C- Phototransduction

GNGT1 + MAPK13 C- Retrograde endocannabinoid signaling,Retrograde endocannabinoid signaling

GNGT1 + PLCB4 C- Serotonergic synapse,Chemokine signaling pathway,Dopaminergic synapse

GNGT2 + PLCB4 C- Serotonergic synapse,Chemokine signaling pathway,Dopaminergic synapse

GNGT2 + MAPK13 C- Retrograde endocannabinoid signaling,Retrograde endocannabinoid signaling

GNGT2 - ADCY6 I- Retrograde endocannabinoid signaling,Retrograde endocannabinoid signaling,GABAergic synapse,GABAergic synapse,Cholinergic synapse,Glutamatergic synapse

GNGT2 + MAPK3 C- Retrograde endocannabinoid signaling,Retrograde endocannabinoid signaling

GPC1 + HBEGF I+ Proteoglycans in cancer

GPC1 + FGF23 C- Proteoglycans in cancer

GPC1 + FGF11 C- Proteoglycans in cancer

GPC1 + FGF12 C- Proteoglycans in cancer

GZMA + F2RL1 C+ Neuroactive ligand-receptor interaction

GZMA + F2RL2 C+ Neuroactive ligand-receptor interaction

HCK + PTK2 C+ Chemokine signaling pathway

HCK + GAB2 I- Fc gamma R-mediated phagocytosis

HCK + SHC1 C+ Chemokine signaling pathway

HCK + FCGR2A C+ Fc gamma R-mediated phagocytosis

HGF + FGFR3 C- PI3K-Akt signaling pathway

HGF + KIT C- PI3K-Akt signaling pathway

HGF + EGFR I+ Melanoma,PI3K-Akt signaling pathway,Focal adhesion

HGF + FGFR2 C- PI3K-Akt signaling pathway

HGF + MET I+ Proteoglycans in cancer,Renal cell carcinoma,Melanoma,Cytokine-cytokine receptor interaction,PI3K-Akt signaling pathway,Focal adhesion,Pathways in cancer

HGF + TEK C- PI3K-Akt signaling pathway

HTR1B + GNG7 C- Serotonergic synapse

HTR1B + GNG13 C- Serotonergic synapse

IGF1 + FGFR2 C- Prostate cancer,PI3K-Akt signaling pathway

IGF1 + KIT C- PI3K-Akt signaling pathway

IGF1 + FGFR3 C- PI3K-Akt signaling pathway

IGF1 + TEK C- PI3K-Akt signaling pathway

IGF1 + EGFR I+ Melanoma,Prostate cancer,PI3K-Akt signaling pathway,Focal adhesion,HIF-1 signaling pathway

IGF1 + MET I+ Melanoma,PI3K-Akt signaling pathway,Focal adhesion

IGF1R + IRS1 C+ PI3K-Akt signaling pathway

IGF1R + SHC1 C+ Glioma,Glioma,Focal adhesion

IGF1R + NRAS C+ Melanoma

IGF1R + GNAZ I- Long-term depression

IGF1R + MAPK3 I- Proteoglycans in cancer

IGF1R + PTK2 C+ Focal adhesion

IL8 + CCR2 C- Chemokine signaling pathway

IL8 + CX3CR1 C- Chemokine signaling pathway

IL8 + CXCR2 C- Cytokine-cytokine receptor interaction,Chemokine signaling pathway

INS + FGFR3 C- PI3K-Akt signaling pathway

INS + EGFR I+ Prostate cancer,PI3K-Akt signaling pathway,HIF-1 signaling pathway

INS + KIT C- PI3K-Akt signaling pathway

INS + FGFR2 C- Prostate cancer,PI3K-Akt signaling pathway

INS + MET I+ PI3K-Akt signaling pathway

INS + TEK C- PI3K-Akt signaling pathway

KDR + PXN C+ VEGF signaling pathway

KDR + PTK2 C+ VEGF signaling pathway,Focal adhesion

KDR + IRS1 C+ PI3K-Akt signaling pathway

KDR + SHC1 C+ Focal adhesion

KITLG + MET I+ PI3K-Akt signaling pathway

KITLG + KIT C- Cytokine-cytokine receptor interaction,PI3K-Akt signaling pathway,Pathways in cancer,Melanogenesis

KITLG + FGFR2 C- PI3K-Akt signaling pathway

KITLG + EGFR I+ PI3K-Akt signaling pathway

KITLG + TEK C- PI3K-Akt signaling pathway

KITLG + FGFR3 C- PI3K-Akt signaling pathway

LOC101060267 + CXCR2 C- Chemokine signaling pathway

LOC101060267 + CX3CR1 C- Chemokine signaling pathway

LOC101060267 + CCR2 C- Chemokine signaling pathway

LOC101060271 + CXCR2 C- Chemokine signaling pathway

LOC101060271 + CCR2 C- Chemokine signaling pathway

LOC101060271 + CX3CR1 C- Chemokine signaling pathway

LOC101060278 + CXCR2 C- Chemokine signaling pathway

LOC101060278 + CCR2 C- Chemokine signaling pathway

LOC101060278 + CX3CR1 C- Chemokine signaling pathway

LYN + CD19 I- Epstein-Barr virus infection

LYN + FCER1G C+ Fc epsilon RI signaling pathway

LYN + FCGR2A C+ Fc gamma R-mediated phagocytosis

LYN + GAB2 I- Fc gamma R-mediated phagocytosis

LYN + MS4A2 I- Fc epsilon RI signaling pathway

LYN + SHC1 C+ Chemokine signaling pathway

LYN + PTK2 C+ Chemokine signaling pathway

MAP3K1 + MAPK13 C- RIG-I-like receptor signaling pathway

MAP3K1 + MAP2K6 C- GnRH signaling pathway

MAPK1 + JMJD7-PLA2G4B C- VEGF signaling pathway,Fc epsilon RI signaling pathway,Long-term depression

MAPK1 + MKNK2 C- Insulin signaling pathway,HIF-1 signaling pathway

MAPK1 + ETS1 I+ Renal cell carcinoma,Pathways in cancer

MAPK1 + CALD1 I+ Vascular smooth muscle contraction

MAPK1 + PLA2G4F C- VEGF signaling pathway,Fc epsilon RI signaling pathway,Fc gamma R-mediated phagocytosis,Long-term depression

MAPK1 + JUN I+ Renal cell carcinoma,B cell receptor signaling pathway,T cell receptor signaling pathway,Toll-like receptor signaling pathway,Pathways in cancer

MAPK1 - NRAS C+ Neurotrophin signaling pathway

MAPK1 + RPS6KA6 C- Long-term potentiation,Progesterone-mediated oocyte maturation,Oocyte meiosis,Neurotrophin signaling pathway

MAPK3 + JUN I+ Renal cell carcinoma,B cell receptor signaling pathway,T cell receptor signaling pathway,Toll-like receptor signaling pathway,Pathways in cancer

MAPK3 + PLA2G4F C- VEGF signaling pathway,Fc epsilon RI signaling pathway,Fc gamma R-mediated phagocytosis,Long-term depression

MAPK3 + ETS1 I+ Renal cell carcinoma,Pathways in cancer

MAPK3 + RPS6KA6 C- Long-term potentiation,Progesterone-mediated oocyte maturation,Oocyte meiosis,Neurotrophin signaling pathway

MAPK3 - NRAS C+ Neurotrophin signaling pathway

MAPK3 + MKNK2 C- Insulin signaling pathway,HIF-1 signaling pathway

MAPK3 + CALD1 I+ Vascular smooth muscle contraction

MAPK3 + JMJD7-PLA2G4B C- VEGF signaling pathway,Fc epsilon RI signaling pathway,Long-term depression

MET + PTK2 C+ Focal adhesion

MET + SHC1 C+ Focal adhesion

MET + NRAS C+ Melanoma

MET + IRS1 C+ PI3K-Akt signaling pathway

NGF + FGFR2 C- PI3K-Akt signaling pathway

NGF + TEK C- PI3K-Akt signaling pathway

NGF + MET I+ PI3K-Akt signaling pathway

NGF + KIT C- PI3K-Akt signaling pathway

NGF + NTRK2 C- MAPK signaling pathway

NGF + FGFR3 C- PI3K-Akt signaling pathway

NGF + EGFR I+ PI3K-Akt signaling pathway

PDGFB + MET I+ Melanoma,PI3K-Akt signaling pathway,Focal adhesion

PDGFB + KIT C- PI3K-Akt signaling pathway

PDGFB + FGFR3 C- PI3K-Akt signaling pathway

PDGFB + TEK C- PI3K-Akt signaling pathway

PDGFB + EGFR I+ Gap junction,Melanoma,Prostate cancer,PI3K-Akt signaling pathway,Focal adhesion

PDGFB + FGFR2 C- Prostate cancer,PI3K-Akt signaling pathway

PDGFC + FGFR2 C- Prostate cancer,PI3K-Akt signaling pathway

PDGFC + FGFR3 C- PI3K-Akt signaling pathway

PDGFC + MET I+ Melanoma,PI3K-Akt signaling pathway,Focal adhesion

PDGFC + TEK C- PI3K-Akt signaling pathway

PDGFC + EGFR I+ Gap junction,Melanoma,Prostate cancer,PI3K-Akt signaling pathway,Focal adhesion

PDGFC + KIT C- PI3K-Akt signaling pathway

PDGFD + EGFR I+ Gap junction,Melanoma,Prostate cancer,PI3K-Akt signaling pathway,Focal adhesion

PDGFD + KIT C- PI3K-Akt signaling pathway

PDGFD + TEK C- PI3K-Akt signaling pathway

PDGFD + FGFR3 C- PI3K-Akt signaling pathway

PDGFD + MET I+ Melanoma,PI3K-Akt signaling pathway,Focal adhesion

PDGFD + FGFR2 C- Prostate cancer,PI3K-Akt signaling pathway

PDGFRA + PTK2 C+ Focal adhesion

PDGFRA + IRS1 C+ PI3K-Akt signaling pathway

PDGFRA + SHC1 C+ Glioma,Glioma,Focal adhesion

PDGFRA + NRAS C+ Melanoma

PDGFRB + NRAS C+ Melanoma

PDGFRB + PTK2 C+ Focal adhesion

PDGFRB + IRS1 C+ PI3K-Akt signaling pathway

PDGFRB + SHC1 C+ Glioma,Glioma,Focal adhesion

PF4 + CXCR2 C- Chemokine signaling pathway

PF4 + CCR2 C- Chemokine signaling pathway

PF4 + CX3CR1 C- Chemokine signaling pathway

PF4V1 + CXCR2 C- Chemokine signaling pathway

PF4V1 + CCR2 C- Chemokine signaling pathway

PF4V1 + CX3CR1 C- Chemokine signaling pathway

PGF + FGFR2 C- PI3K-Akt signaling pathway

PGF + KIT C- PI3K-Akt signaling pathway

PGF + EGFR I+ PI3K-Akt signaling pathway,Focal adhesion

PGF + MET I+ PI3K-Akt signaling pathway,Focal adhesion

PGF + FGFR3 C- PI3K-Akt signaling pathway

PGF + TEK C- PI3K-Akt signaling pathway

PIK3CA + RAC2 C+ Fc epsilon RI signaling pathway,Natural killer cell mediated cytotoxicity

PIK3CA + NRAS C+ Chemokine signaling pathway

PIK3CA + PTK2 C+ Chemokine signaling pathway

PIK3CB + NRAS C+ Chemokine signaling pathway

PIK3CB + PTK2 C+ Chemokine signaling pathway

PIK3CB + RAC2 C+ Fc epsilon RI signaling pathway,Natural killer cell mediated cytotoxicity

PIK3CD + RAC2 C+ Fc epsilon RI signaling pathway,Natural killer cell mediated cytotoxicity

PIK3CD + NRAS C+ Chemokine signaling pathway

PIK3CD + PTK2 C+ Chemokine signaling pathway

PIK3CG + RAC2 C+ Fc epsilon RI signaling pathway,Natural killer cell mediated cytotoxicity

PIK3CG + NRAS C+ Chemokine signaling pathway,Cholinergic synapse

PIK3CG + PTK2 C+ Chemokine signaling pathway

PIK3R1 + PTK2 C+ Chemokine signaling pathway

PIK3R1 + RAC2 C+ Fc epsilon RI signaling pathway,Natural killer cell mediated cytotoxicity

PIK3R1 + NRAS C+ Chemokine signaling pathway,Cholinergic synapse

PIK3R2 + PTK2 C+ Chemokine signaling pathway

PIK3R2 + RAC2 C+ Fc epsilon RI signaling pathway,Natural killer cell mediated cytotoxicity

PIK3R2 + NRAS C+ Chemokine signaling pathway,Cholinergic synapse

PIK3R3 + NRAS C+ Chemokine signaling pathway,Cholinergic synapse

PIK3R3 + RAC2 C+ Fc epsilon RI signaling pathway,Natural killer cell mediated cytotoxicity

PIK3R3 + PTK2 C+ Chemokine signaling pathway

PIK3R5 + RAC2 C+ Fc epsilon RI signaling pathway,Natural killer cell mediated cytotoxicity

PIK3R5 + NRAS C+ Chemokine signaling pathway,Cholinergic synapse

PIK3R5 + PTK2 C+ Chemokine signaling pathway

PPBP + CXCR2 C- Cytokine-cytokine receptor interaction,Chemokine signaling pathway

PPBP + CX3CR1 C- Chemokine signaling pathway

PPBP + CCR2 C- Chemokine signaling pathway

PPP1CA - CALML5 C- Insulin signaling pathway

PPP1CA - CALML3 C- Insulin signaling pathway

PPP1CA + GYS2 I- Insulin signaling pathway

PPP1CA - CALML6 C- Insulin signaling pathway

PPP1CB - CALML3 C- Insulin signaling pathway

PPP1CB + GYS2 I- Insulin signaling pathway

PPP1CB - CALML6 C- Insulin signaling pathway

PPP1CB - CALML5 C- Insulin signaling pathway

PPP1CC - CALML3 C- Insulin signaling pathway

PPP1CC - CALML5 C- Insulin signaling pathway

PPP1CC + GYS2 I- Insulin signaling pathway

PPP1CC - CALML6 C- Insulin signaling pathway

PPP1R3A - CALML6 C- Insulin signaling pathway

PPP1R3A - CALML5 C- Insulin signaling pathway

PPP1R3A - CALML3 C- Insulin signaling pathway

PPP1R3A + GYS2 I- Insulin signaling pathway

PPP1R3B + GYS2 I- Insulin signaling pathway

PPP1R3B - CALML3 C- Insulin signaling pathway

PPP1R3B - CALML6 C- Insulin signaling pathway

PPP1R3B - CALML5 C- Insulin signaling pathway

PPP1R3D - CALML5 C- Insulin signaling pathway

PPP1R3D - CALML3 C- Insulin signaling pathway

PPP1R3D - CALML6 C- Insulin signaling pathway

PPP1R3D + GYS2 I- Insulin signaling pathway

PRKACA + ADCY6 C- Cholinergic synapse

PRKACA + CFTR C- Bile secretion

PRKACA - GLI3 C+ Hedgehog signaling pathway

PRKACA - ITPR3 A+ Vascular smooth muscle contraction

PRKACA + MAPK13 C- Dopaminergic synapse

PRKACA - ITPR2 I- Vascular smooth muscle contraction

PRKACA + MAPK3 C- Long-term potentiation,Cholinergic synapse

PRKACA + PTK2 I+ Proteoglycans in cancer

PRKACB + ADCY6 C- Cholinergic synapse

PRKACB - ITPR2 I- Vascular smooth muscle contraction

PRKACB - ITPR3 A+ Vascular smooth muscle contraction

PRKACB + MAPK3 C- Long-term potentiation,Cholinergic synapse

PRKACB + CFTR C- Bile secretion

PRKACB - GLI3 C+ Hedgehog signaling pathway

PRKACB + PTK2 I+ Proteoglycans in cancer

PRKACB + MAPK13 C- Dopaminergic synapse

PRKACG - GLI3 C+ Hedgehog signaling pathway

PRKACG + PTK2 I+ Proteoglycans in cancer

PRKACG + ADCY6 C- Cholinergic synapse

PRKACG + MAPK13 C- Dopaminergic synapse

PRKACG + MAPK3 C- Long-term potentiation,Cholinergic synapse

PRKACG - ITPR3 A+ Vascular smooth muscle contraction

PRKACG - ITPR2 I- Vascular smooth muscle contraction

PRKACG + CFTR C- Bile secretion

PRKCB + NRAS I+ Glioma,Glioma

PRKCB + PLD1 C- Glutamatergic synapse

PRKCB + PTK2 I+ Focal adhesion

PRKCB + SPHK1 I+ VEGF signaling pathway

PRKCB + MAPK3 C- Glutamatergic synapse

PRKCB + PLA2G4F C- Glutamatergic synapse

PRKCB + JMJD7-PLA2G4B C- Glutamatergic synapse

PRKCB + TYR C- Melanogenesis

PRKX - GLI3 C+ Hedgehog signaling pathway

PRKX + CFTR C- Bile secretion

PRKX - ITPR2 I- Vascular smooth muscle contraction

PRKX + PTK2 I+ Proteoglycans in cancer

PRKX + MAPK3 C- Long-term potentiation,Cholinergic synapse

PRKX + ADCY6 C- Cholinergic synapse

PRKX - ITPR3 A+ Vascular smooth muscle contraction

PRKX + MAPK13 C- Dopaminergic synapse

PTK2 + MAPK13 I- Proteoglycans in cancer

PTK2 + PXN C+ Chemokine signaling pathway,Focal adhesion

PTK2 + SHC1 C+ Focal adhesion

PTPN6 - IL12RB2 C+ Jak-STAT signaling pathway

PTPN6 - CNTFR I- Jak-STAT signaling pathway

PTPN6 - PRLR I- Jak-STAT signaling pathway

PTPN6 - IL13RA2 C+ Jak-STAT signaling pathway

PTPN6 - IFNAR2 C+ Jak-STAT signaling pathway

PTPN6 - IL20RB I- Jak-STAT signaling pathway

PTPN6 - IL7R C+ Jak-STAT signaling pathway

PTPN6 - OSMR C+ Jak-STAT signaling pathway

PTPN6 - LIFR I- Jak-STAT signaling pathway

PTPN6 - IL11RA I- Jak-STAT signaling pathway

PTPN6 - LCP2 C+ Natural killer cell mediated cytotoxicity

PTPN6 - VAV2 C+ Natural killer cell mediated cytotoxicity

PTPN6 - IL2RA C+ Jak-STAT signaling pathway

RAC1 + PAK1 I+ Regulation of actin cytoskeleton,Fc gamma R-mediated phagocytosis,Renal cell carcinoma,MAPK signaling pathway,Axon guidance,Axon guidance,Axon guidance,Chemokine signaling pathway,Focal adhesion,Natural killer cell mediated cytotoxicity

RAC1 + PIP5K1B C- Fc gamma R-mediated phagocytosis

RAC1 + ABLIM1 C- Axon guidance

RAC1 + ABLIM2 C- Axon guidance

RAC1 + NCF2 I+ Osteoclast differentiation

RAC1 + MAP2K6 C- Fc epsilon RI signaling pathway

RAC1 + MAP3K1 C- MAPK signaling pathway,Neurotrophin signaling pathway

RAC2 + ABLIM1 C- Axon guidance

RAC2 + MAP2K6 C- Fc epsilon RI signaling pathway

RAC2 + ABLIM2 C- Axon guidance

RAC2 + PIP5K1B C- Fc gamma R-mediated phagocytosis

RAC2 + MAP3K1 C- MAPK signaling pathway

RAC2 + PAK1 I+ Regulation of actin cytoskeleton,Fc gamma R-mediated phagocytosis,MAPK signaling pathway,Axon guidance,Axon guidance,Axon guidance,Chemokine signaling pathway,Focal adhesion,Natural killer cell mediated cytotoxicity

RAC3 + PAK1 I+ Regulation of actin cytoskeleton,MAPK signaling pathway,Axon guidance,Axon guidance,Axon guidance,Focal adhesion,Natural killer cell mediated cytotoxicity

RAC3 + MAP2K6 C- Fc epsilon RI signaling pathway

RAC3 + MAP3K1 C- MAPK signaling pathway

RAC3 + ABLIM2 C- Axon guidance

RAC3 + ABLIM1 C- Axon guidance

RNF125 - DDX58 C+ RIG-I-like receptor signaling pathway

RNF125 - IFIH1 C+ RIG-I-like receptor signaling pathway

SDC4 + PTK2 C+ Proteoglycans in cancer

SDC4 + PXN C+ Proteoglycans in cancer

SOCS1 - IRS1 C+ Type II diabetes mellitus

SOCS1 - IL13RA2 C+ Jak-STAT signaling pathway

SOCS1 - CNTFR I- Jak-STAT signaling pathway

SOCS1 - IL20RB I- Jak-STAT signaling pathway

SOCS1 - OSMR C+ Jak-STAT signaling pathway

SOCS1 - IL11RA I- Jak-STAT signaling pathway

SOCS1 - PRLR I- Jak-STAT signaling pathway

SOCS1 - IL7R C+ Jak-STAT signaling pathway

SOCS1 - LIFR I- Jak-STAT signaling pathway

SOCS1 - IL2RA C+ Jak-STAT signaling pathway

SOCS1 - IFNAR2 C+ Jak-STAT signaling pathway

SOCS1 - IL12RB2 C+ Jak-STAT signaling pathway

SOCS2 - IL20RB I- Jak-STAT signaling pathway

SOCS2 - LIFR I- Jak-STAT signaling pathway

SOCS2 - IL13RA2 C+ Jak-STAT signaling pathway

SOCS2 - IL12RB2 C+ Jak-STAT signaling pathway

SOCS2 - IL2RA C+ Jak-STAT signaling pathway

SOCS2 - IL7R C+ Jak-STAT signaling pathway

SOCS2 - IRS1 C+ Type II diabetes mellitus

SOCS2 - IFNAR2 C+ Jak-STAT signaling pathway

SOCS2 - CNTFR I- Jak-STAT signaling pathway

SOCS2 - IL11RA I- Jak-STAT signaling pathway

SOCS2 - PRLR I- Jak-STAT signaling pathway

SOCS2 - OSMR C+ Jak-STAT signaling pathway

SOCS4 - IRS1 C+ Type II diabetes mellitus

SOCS4 - IL12RB2 C+ Jak-STAT signaling pathway

SOCS4 - IL11RA I- Jak-STAT signaling pathway

SOCS4 - IL20RB I- Jak-STAT signaling pathway

SOCS4 - CNTFR I- Jak-STAT signaling pathway

SOCS4 - PRLR I- Jak-STAT signaling pathway

SOCS4 - IL2RA C+ Jak-STAT signaling pathway

SOCS4 - IL7R C+ Jak-STAT signaling pathway

SOCS4 - OSMR C+ Jak-STAT signaling pathway

SOCS4 - IFNAR2 C+ Jak-STAT signaling pathway

SOCS4 - LIFR I- Jak-STAT signaling pathway

SOCS4 - IL13RA2 C+ Jak-STAT signaling pathway

SRC + MMP9 C+ Estrogen signaling pathway

SRC + EGFR C+ GnRH signaling pathway

SRC + SHC1 C+ Chemokine signaling pathway

SRC + PTK2 C+ Proteoglycans in cancer,ErbB signaling pathway,Chemokine signaling pathway

SRC + NRAS C+ Estrogen signaling pathway

VEGFA + MET I+ PI3K-Akt signaling pathway,Focal adhesion

VEGFA + EGFR I+ PI3K-Akt signaling pathway,Focal adhesion

VEGFA + FGFR2 C- PI3K-Akt signaling pathway

VEGFA + FGFR3 C- PI3K-Akt signaling pathway

VEGFA + TEK C- PI3K-Akt signaling pathway

VEGFA + KIT C- PI3K-Akt signaling pathway

VEGFB + KIT C- PI3K-Akt signaling pathway

VEGFB + FGFR3 C- PI3K-Akt signaling pathway

VEGFB + MET I+ PI3K-Akt signaling pathway,Focal adhesion

VEGFB + TEK C- PI3K-Akt signaling pathway

VEGFB + FGFR2 C- PI3K-Akt signaling pathway

VEGFB + EGFR I+ PI3K-Akt signaling pathway,Focal adhesion

XCL1 + CXCR2 C- Chemokine signaling pathway

XCL1 + CX3CR1 C- Chemokine signaling pathway

XCL1 + CCR2 C- Chemokine signaling pathway

XCL2 + CXCR2 C- Chemokine signaling pathway

XCL2 + CX3CR1 C- Chemokine signaling pathway

XCL2 + CCR2 C- Chemokine signaling pathway
