# Supplementary material for: Potential Therapeutic Targets for Oral Cancer: ADM, TP53, EGFR, LYN, CTLA4, SKIL, CTGF, CD70
Source: PLoS One. 2014 Jul 16;9(7):e102610. doi: 10.1371/journal.pone.0102610 (PMC4110113; doi:10.1371/journal.pone.0102610)
Supplement: Text S6 — Processed list of hypotheses and downstream genes. File contains following columns: (i) Causal Hypothesis/Gene Name→Name of the gene which has been identified as significant hypothesis by causal reasoning analysis; (ii) Diff_Exp→The hypothesis genes which are differentially expressed in our analysis are marked as ‘DE’ and non-differentially expressed hypothesis genes are marked as ‘N’; (iii) Regulation→Relationship between hypothesis gene and downstream gene, ‘+’ represents activation and ‘−’ represents inhibition; (iv) Downstream Gene(s) →Downstream gene(s) whose differential expression is predicted by hypothesis gene; (v) Prediction→‘C’ represents correctly predicted relationship between hypothesis and downstream gene, whereas ‘I’ represents otherwise. The succeeding +/− sign represents up−/down-regulation resp., of the downstream gene(s); (vi) Source→The name of Kegg pathway(s) used to infer causal relationship between hypothesis & related downstream gene(s). (DOCX) [file pone.0102610.s006.docx]

Causal Hypothesis/Gene Name Diff_Exp Regulation Downstream Gene(s) Prediction Source

ANGPT1 N + FGFR2 C- PI3K-Akt signaling pathway

ANGPT1 N + TEK C- PI3K-Akt signaling pathway

ANGPT1 N + KIT C- PI3K-Akt signaling pathway

ANGPT1 N + FGFR3 C- PI3K-Akt signaling pathway

ANGPT4 DE + FGFR3 C- PI3K-Akt signaling pathway

ANGPT4 DE + KIT C- PI3K-Akt signaling pathway

ANGPT4 DE + TEK C- PI3K-Akt signaling pathway

ANGPT4 DE + FGFR2 C- PI3K-Akt signaling pathway

CALML3 DE - CNGB1 C+ Phototransduction

CALML3 DE + NOS1 C- Salivary secretion

CALML3 DE + CHP2 C- Long-term potentiation,Oocyte meiosis

CALML5 DE - CNGB1 C+ Phototransduction

CALML5 DE + NOS1 C- Salivary secretion

CALML5 DE + CHP2 C- Long-term potentiation,Oocyte meiosis

CALML6 DE + NOS1 C- Salivary secretion

CALML6 DE + CHP2 C- Long-term potentiation,Oocyte meiosis

CALML6 DE - CNGB1 C+ Phototransduction

CCL1 N + CCR2 C- Chemokine signaling pathway

CCL1 N + CX3CR1 C- Chemokine signaling pathway

CCL1 N + CXCR2 C- Chemokine signaling pathway

CCL14 DE + CX3CR1 C- Chemokine signaling pathway

CCL14 DE + CXCR2 C- Chemokine signaling pathway

CCL14 DE + CCR2 C- Chemokine signaling pathway

CCL15 DE + CXCR2 C- Chemokine signaling pathway

CCL15 DE + CX3CR1 C- Chemokine signaling pathway

CCL15 DE + CCR2 C- Chemokine signaling pathway

CCL16 N + CCR2 C- Chemokine signaling pathway

CCL16 N + CX3CR1 C- Chemokine signaling pathway

CCL16 N + CXCR2 C- Chemokine signaling pathway

CCL17 N + CXCR2 C- Chemokine signaling pathway

CCL17 N + CX3CR1 C- Chemokine signaling pathway

CCL17 N + CCR2 C- Chemokine signaling pathway

CCL19 DE + CCR2 C- Chemokine signaling pathway

CCL19 DE + CX3CR1 C- Chemokine signaling pathway

CCL19 DE + CXCR2 C- Chemokine signaling pathway

CCL21 DE + CXCR2 C- Chemokine signaling pathway

CCL21 DE + CX3CR1 C- Chemokine signaling pathway

CCL21 DE + CCR2 C- Chemokine signaling pathway

CCL2 N + CX3CR1 C- Chemokine signaling pathway

CCL2 N + CCR2 C- Cytokine-cytokine receptor interaction,Chemokine signaling pathway

CCL2 N + CXCR2 C- Chemokine signaling pathway

CCL22 N + CXCR2 C- Chemokine signaling pathway

CCL22 N + CX3CR1 C- Chemokine signaling pathway

CCL22 N + CCR2 C- Chemokine signaling pathway

CCL23 N + CXCR2 C- Chemokine signaling pathway

CCL23 N + CCR2 C- Chemokine signaling pathway

CCL23 N + CX3CR1 C- Chemokine signaling pathway

CCL25 N + CX3CR1 C- Chemokine signaling pathway

CCL25 N + CCR2 C- Chemokine signaling pathway

CCL25 N + CXCR2 C- Chemokine signaling pathway

CCL26 N + CXCR2 C- Chemokine signaling pathway

CCL26 N + CX3CR1 C- Chemokine signaling pathway

CCL26 N + CCR2 C- Chemokine signaling pathway

CCL28 N + CX3CR1 C- Chemokine signaling pathway

CCL28 N + CCR2 C- Chemokine signaling pathway

CCL28 N + CXCR2 C- Chemokine signaling pathway

CD80 DE + CD274 C+ Cell adhesion molecules (CAMs)

CD80 DE + CTLA4 C+ Cell adhesion molecules (CAMs)

COL4A1 DE + SDC4 C+ ECM-receptor interaction

COL4A1 DE + CD44 C+ ECM-receptor interaction

COL4A2 DE + CD44 C+ ECM-receptor interaction

COL4A2 DE + SDC4 C+ ECM-receptor interaction

COL4A6 DE + CD44 C+ ECM-receptor interaction

COL4A6 DE + SDC4 C+ ECM-receptor interaction

COL5A2 DE + SDC4 C+ ECM-receptor interaction

COL5A2 DE + CD44 C+ ECM-receptor interaction

CRK N + ABL2 C+ ErbB signaling pathway

CRK N + PXN C+ Focal adhesion

CRK N + RAPGEF1 C+ Renal cell carcinoma,Focal adhesion

CRKL N + PXN C+ Focal adhesion

CRKL N + ABL2 C+ ErbB signaling pathway

CRKL N + RAPGEF1 C+ Renal cell carcinoma,Focal adhesion

CX3CL1 DE + CCR2 C- Chemokine signaling pathway

CX3CL1 DE + CX3CR1 C- Cytokine-cytokine receptor interaction,Chemokine signaling pathway

CX3CL1 DE + CXCR2 C- Chemokine signaling pathway

CXCL12 DE + CXCR2 C- Chemokine signaling pathway

CXCL12 DE + CX3CR1 C- Chemokine signaling pathway

CXCL12 DE + CCR2 C- Chemokine signaling pathway

CXCL14 N + CXCR2 C- Chemokine signaling pathway

CXCL14 N + CCR2 C- Chemokine signaling pathway

CXCL14 N + CX3CR1 C- Chemokine signaling pathway

DUSP4 DE - MAPK3 C- MAPK signaling pathway

DUSP4 DE - MAPK13 C- MAPK signaling pathway

DUSP6 DE - MAPK3 C- MAPK signaling pathway

DUSP6 DE - MAPK13 C- MAPK signaling pathway

EFNA2 DE + FGFR3 C- PI3K-Akt signaling pathway

EFNA2 DE + TEK C- PI3K-Akt signaling pathway

EFNA2 DE + KIT C- PI3K-Akt signaling pathway

EFNA2 DE + FGFR2 C- PI3K-Akt signaling pathway

EFNA2 DE + EPHA7 C- Axon guidance

EFNA3 N + FGFR3 C- PI3K-Akt signaling pathway

EFNA3 N + KIT C- PI3K-Akt signaling pathway

EFNA3 N + TEK C- PI3K-Akt signaling pathway

EFNA3 N + FGFR2 C- PI3K-Akt signaling pathway

EFNA3 N + EPHA7 C- Axon guidance

EFNA4 N + FGFR2 C- PI3K-Akt signaling pathway

EFNA4 N + KIT C- PI3K-Akt signaling pathway

EFNA4 N + EPHA7 C- Axon guidance

EFNA4 N + FGFR3 C- PI3K-Akt signaling pathway

EFNA4 N + TEK C- PI3K-Akt signaling pathway

EFNA5 N + FGFR2 C- PI3K-Akt signaling pathway

EFNA5 N + TEK C- PI3K-Akt signaling pathway

EFNA5 N + KIT C- PI3K-Akt signaling pathway

EFNA5 N + EPHA7 C- Axon guidance

EFNA5 N + FGFR3 C- PI3K-Akt signaling pathway

EGFR DE + PTK2 C+ Focal adhesion

EGFR DE + IRS1 C+ PI3K-Akt signaling pathway

EGFR DE + NRAS C+ Melanoma

EGFR DE + SHC1 C+ ErbB signaling pathway,ErbB signaling pathway,Estrogen signaling pathway,Glioma,Glioma

FGF10 N + KIT C- PI3K-Akt signaling pathway

FGF10 N + FGFR2 C- MAPK signaling pathway,PI3K-Akt signaling pathway,Pathways in cancer

FGF10 N + FGFR3 C- MAPK signaling pathway,PI3K-Akt signaling pathway,Pathways in cancer

FGF10 N + TEK C- PI3K-Akt signaling pathway

FGF11 DE + TEK C- PI3K-Akt signaling pathway

FGF11 DE + FGFR2 C- MAPK signaling pathway,PI3K-Akt signaling pathway,Pathways in cancer

FGF11 DE + KIT C- PI3K-Akt signaling pathway

FGF11 DE + FGFR3 C- MAPK signaling pathway,PI3K-Akt signaling pathway,Pathways in cancer

FGF1 N + FGFR2 C- MAPK signaling pathway,PI3K-Akt signaling pathway,Pathways in cancer

FGF1 N + TEK C- PI3K-Akt signaling pathway

FGF1 N + FGFR3 C- MAPK signaling pathway,PI3K-Akt signaling pathway,Pathways in cancer

FGF1 N + KIT C- PI3K-Akt signaling pathway

FGF12 DE + FGFR2 C- MAPK signaling pathway,PI3K-Akt signaling pathway,Pathways in cancer

FGF12 DE + FGFR3 C- MAPK signaling pathway,PI3K-Akt signaling pathway,Pathways in cancer

FGF12 DE + KIT C- PI3K-Akt signaling pathway

FGF12 DE + TEK C- PI3K-Akt signaling pathway

FGF13 N + KIT C- PI3K-Akt signaling pathway

FGF13 N + TEK C- PI3K-Akt signaling pathway

FGF13 N + FGFR3 C- MAPK signaling pathway,PI3K-Akt signaling pathway,Pathways in cancer

FGF13 N + FGFR2 C- MAPK signaling pathway,PI3K-Akt signaling pathway,Pathways in cancer

FGF14 N + KIT C- PI3K-Akt signaling pathway

FGF14 N + FGFR2 C- MAPK signaling pathway,PI3K-Akt signaling pathway,Pathways in cancer

FGF14 N + TEK C- PI3K-Akt signaling pathway

FGF14 N + FGFR3 C- MAPK signaling pathway,PI3K-Akt signaling pathway,Pathways in cancer

FGF17 N + FGFR2 C- MAPK signaling pathway,PI3K-Akt signaling pathway,Pathways in cancer

FGF17 N + TEK C- PI3K-Akt signaling pathway

FGF17 N + KIT C- PI3K-Akt signaling pathway

FGF17 N + FGFR3 C- MAPK signaling pathway,PI3K-Akt signaling pathway,Pathways in cancer

FGF18 N + FGFR3 C- MAPK signaling pathway,PI3K-Akt signaling pathway,Pathways in cancer

FGF18 N + KIT C- PI3K-Akt signaling pathway

FGF18 N + FGFR2 C- MAPK signaling pathway,PI3K-Akt signaling pathway,Pathways in cancer

FGF18 N + TEK C- PI3K-Akt signaling pathway

FGF19 N + FGFR2 C- MAPK signaling pathway,PI3K-Akt signaling pathway,Pathways in cancer

FGF19 N + KIT C- PI3K-Akt signaling pathway

FGF19 N + FGFR3 C- MAPK signaling pathway,PI3K-Akt signaling pathway,Pathways in cancer

FGF19 N + TEK C- PI3K-Akt signaling pathway

FGF20 N + FGFR2 C- MAPK signaling pathway,PI3K-Akt signaling pathway,Pathways in cancer

FGF20 N + KIT C- PI3K-Akt signaling pathway

FGF20 N + FGFR3 C- MAPK signaling pathway,PI3K-Akt signaling pathway,Pathways in cancer

FGF20 N + TEK C- PI3K-Akt signaling pathway

FGF21 N + FGFR3 C- MAPK signaling pathway,PI3K-Akt signaling pathway,Pathways in cancer

FGF21 N + TEK C- PI3K-Akt signaling pathway

FGF21 N + KIT C- PI3K-Akt signaling pathway

FGF21 N + FGFR2 C- MAPK signaling pathway,PI3K-Akt signaling pathway,Pathways in cancer

FGF2 N + KIT C- PI3K-Akt signaling pathway

FGF2 N + FGFR3 C- MAPK signaling pathway,PI3K-Akt signaling pathway,Pathways in cancer

FGF2 N + TEK C- PI3K-Akt signaling pathway

FGF2 N + FGFR2 C- MAPK signaling pathway,PI3K-Akt signaling pathway,Pathways in cancer

FGF22 N + KIT C- PI3K-Akt signaling pathway

FGF22 N + FGFR3 C- MAPK signaling pathway,PI3K-Akt signaling pathway,Pathways in cancer

FGF22 N + TEK C- PI3K-Akt signaling pathway

FGF22 N + FGFR2 C- MAPK signaling pathway,PI3K-Akt signaling pathway,Pathways in cancer

FGF23 DE + FGFR2 C- MAPK signaling pathway,PI3K-Akt signaling pathway,Pathways in cancer

FGF23 DE + TEK C- PI3K-Akt signaling pathway

FGF23 DE + FGFR3 C- MAPK signaling pathway,PI3K-Akt signaling pathway,Pathways in cancer

FGF23 DE + KIT C- PI3K-Akt signaling pathway

FGF3 N + FGFR3 C- MAPK signaling pathway,PI3K-Akt signaling pathway,Pathways in cancer

FGF3 N + FGFR2 C- MAPK signaling pathway,PI3K-Akt signaling pathway,Pathways in cancer

FGF3 N + TEK C- PI3K-Akt signaling pathway

FGF3 N + KIT C- PI3K-Akt signaling pathway

FGF4 N + FGFR2 C- MAPK signaling pathway,PI3K-Akt signaling pathway,Pathways in cancer

FGF4 N + FGFR3 C- MAPK signaling pathway,PI3K-Akt signaling pathway,Pathways in cancer

FGF4 N + TEK C- PI3K-Akt signaling pathway

FGF4 N + KIT C- PI3K-Akt signaling pathway

FGF5 N + TEK C- PI3K-Akt signaling pathway

FGF5 N + FGFR2 C- MAPK signaling pathway,PI3K-Akt signaling pathway,Pathways in cancer

FGF5 N + KIT C- PI3K-Akt signaling pathway

FGF5 N + FGFR3 C- MAPK signaling pathway,PI3K-Akt signaling pathway,Pathways in cancer

FGF6 N + FGFR3 C- MAPK signaling pathway,PI3K-Akt signaling pathway,Pathways in cancer

FGF6 N + KIT C- PI3K-Akt signaling pathway

FGF6 N + TEK C- PI3K-Akt signaling pathway

FGF6 N + FGFR2 C- MAPK signaling pathway,PI3K-Akt signaling pathway,Pathways in cancer

FGF7 N + TEK C- PI3K-Akt signaling pathway

FGF7 N + KIT C- PI3K-Akt signaling pathway

FGF7 N + FGFR3 C- MAPK signaling pathway,PI3K-Akt signaling pathway,Pathways in cancer

FGF7 N + FGFR2 C- MAPK signaling pathway,PI3K-Akt signaling pathway,Pathways in cancer

FGF8 N + FGFR2 C- MAPK signaling pathway,PI3K-Akt signaling pathway,Pathways in cancer

FGF8 N + FGFR3 C- MAPK signaling pathway,PI3K-Akt signaling pathway,Pathways in cancer

FGF8 N + TEK C- PI3K-Akt signaling pathway

FGF8 N + KIT C- PI3K-Akt signaling pathway

FGF9 N + TEK C- PI3K-Akt signaling pathway

FGF9 N + KIT C- PI3K-Akt signaling pathway

FGF9 N + FGFR3 C- MAPK signaling pathway,PI3K-Akt signaling pathway,Pathways in cancer

FGF9 N + FGFR2 C- MAPK signaling pathway,PI3K-Akt signaling pathway,Pathways in cancer

FIGF DE + FGFR2 C- PI3K-Akt signaling pathway

FIGF DE + FGFR3 C- PI3K-Akt signaling pathway

FIGF DE + KIT C- PI3K-Akt signaling pathway

FIGF DE + TEK C- PI3K-Akt signaling pathway

FLT1 N + SHC1 C+ Focal adhesion

FLT1 N + IRS1 C+ PI3K-Akt signaling pathway

FLT1 N + PTK2 C+ Focal adhesion

FN1 DE + ITGAV C+ Proteoglycans in cancer

FN1 DE + ITGA5 C+ Proteoglycans in cancer,Proteoglycans in cancer

FN1 DE + SDC4 C+ Proteoglycans in cancer,ECM-receptor interaction

FN1 DE + CD44 C+ ECM-receptor interaction

FN1 DE + ITGB1 C+ Proteoglycans in cancer,Proteoglycans in cancer

GNAI2 N + PLCB4 C- Serotonergic synapse,Dopaminergic synapse,Melanogenesis

GNAI2 N + MAPK3 C- Retrograde endocannabinoid signaling,Retrograde endocannabinoid signaling

GNAI2 N + MAPK13 C- Retrograde endocannabinoid signaling,Retrograde endocannabinoid signaling

GNAI2 N + JMJD7-PLA2G4B C- Long-term depression,Long-term depression

GNAI2 N + PLA2G4F C- Long-term depression,Long-term depression

GNAO1 N + PLCB4 C- Serotonergic synapse,Dopaminergic synapse,Melanogenesis

GNAO1 N + MAPK13 C- Retrograde endocannabinoid signaling,Retrograde endocannabinoid signaling

GNAO1 N + PLA2G4F C- Long-term depression,Long-term depression

GNAO1 N + MAPK3 C- Retrograde endocannabinoid signaling,Retrograde endocannabinoid signaling

GNAO1 N + JMJD7-PLA2G4B C- Long-term depression,Long-term depression

GNAQ N + PLCB4 C- GnRH signaling pathway,Gap junction,Retrograde endocannabinoid signaling,Estrogen signaling pathway,Long-term potentiation,Serotonergic synapse,Serotonergic synapse,Cholinergic synapse,Salivary secretion,Insulin secretion,Pancreatic secretion,Long-term depression,Long-term depression,Gastric acid secretion,Dopaminergic synapse,Vascular smooth muscle contraction,Glutamatergic synapse,Calcium signaling pathway

GNAQ N + PLA2G1B C- Vascular smooth muscle contraction

GNAQ N + PLA2G3 C- Vascular smooth muscle contraction

GNAQ N + JMJD7-PLA2G4B C- Serotonergic synapse,Vascular smooth muscle contraction

GNAQ N + PLA2G4F C- Serotonergic synapse,Vascular smooth muscle contraction

GNAS N + ADCY6 C- GnRH signaling pathway,Bile secretion,Ovarian steroidogenesis,Ovarian steroidogenesis,Gap junction,Vasopressin-regulated water reabsorption,Estrogen signaling pathway,Salivary secretion,Insulin secretion,Pancreatic secretion,Gastric acid secretion,Vascular smooth muscle contraction,Glutamatergic synapse,Melanogenesis

GNAS N + PLA2G4F C- Ovarian steroidogenesis,Long-term depression,Long-term depression

GNAS N + JMJD7-PLA2G4B C- Ovarian steroidogenesis,Long-term depression,Long-term depression

GNAZ DE + JMJD7-PLA2G4B C- Long-term depression,Long-term depression

GNAZ DE + PLA2G4F C- Long-term depression,Long-term depression

GNB3 N + MAPK3 C- Retrograde endocannabinoid signaling,Retrograde endocannabinoid signaling

GNB3 N + MAPK13 C- Retrograde endocannabinoid signaling,Retrograde endocannabinoid signaling

GNB3 N + PLCB4 C- Serotonergic synapse,Chemokine signaling pathway,Dopaminergic synapse

GNG11 N + MAPK3 C- Retrograde endocannabinoid signaling,Retrograde endocannabinoid signaling

GNG11 N + PLCB4 C- Serotonergic synapse,Chemokine signaling pathway,Dopaminergic synapse

GNG11 N + MAPK13 C- Retrograde endocannabinoid signaling,Retrograde endocannabinoid signaling

GNG13 DE + MAPK13 C- Retrograde endocannabinoid signaling,Retrograde endocannabinoid signaling

GNG13 DE + MAPK3 C- Retrograde endocannabinoid signaling,Retrograde endocannabinoid signaling

GNG13 DE + PLCB4 C- Serotonergic synapse,Chemokine signaling pathway,Dopaminergic synapse

GNG2 N + MAPK13 C- Retrograde endocannabinoid signaling,Retrograde endocannabinoid signaling

GNG2 N + PLCB4 C- Serotonergic synapse,Chemokine signaling pathway,Dopaminergic synapse

GNG2 N + MAPK3 C- Retrograde endocannabinoid signaling,Retrograde endocannabinoid signaling

GNG3 N + MAPK3 C- Retrograde endocannabinoid signaling,Retrograde endocannabinoid signaling

GNG3 N + MAPK13 C- Retrograde endocannabinoid signaling,Retrograde endocannabinoid signaling

GNG3 N + PLCB4 C- Serotonergic synapse,Chemokine signaling pathway,Dopaminergic synapse

GNG4 N + PLCB4 C- Serotonergic synapse,Chemokine signaling pathway,Dopaminergic synapse

GNG4 N + MAPK3 C- Retrograde endocannabinoid signaling,Retrograde endocannabinoid signaling

GNG4 N + MAPK13 C- Retrograde endocannabinoid signaling,Retrograde endocannabinoid signaling

GNG7 DE + MAPK13 C- Retrograde endocannabinoid signaling,Retrograde endocannabinoid signaling

GNG7 DE + MAPK3 C- Retrograde endocannabinoid signaling,Retrograde endocannabinoid signaling

GNG7 DE + PLCB4 C- Serotonergic synapse,Chemokine signaling pathway,Dopaminergic synapse

GNG8 N + MAPK13 C- Retrograde endocannabinoid signaling,Retrograde endocannabinoid signaling

GNG8 N + PLCB4 C- Serotonergic synapse,Chemokine signaling pathway,Dopaminergic synapse

GNG8 N + MAPK3 C- Retrograde endocannabinoid signaling,Retrograde endocannabinoid signaling

GPC1 N + FGF23 C- Proteoglycans in cancer

GPC1 N + FGF11 C- Proteoglycans in cancer

GPC1 N + FGF12 C- Proteoglycans in cancer

GZMA DE + F2RL1 C+ Neuroactive ligand-receptor interaction

GZMA DE + F2RL2 C+ Neuroactive ligand-receptor interaction

HCK N + PTK2 C+ Chemokine signaling pathway

HCK N + SHC1 C+ Chemokine signaling pathway

HCK N + FCGR2A C+ Fc gamma R-mediated phagocytosis

HGF N + FGFR3 C- PI3K-Akt signaling pathway

HGF N + KIT C- PI3K-Akt signaling pathway

HGF N + FGFR2 C- PI3K-Akt signaling pathway

HGF N + TEK C- PI3K-Akt signaling pathway

HTR1B DE + GNG7 C- Serotonergic synapse

HTR1B DE + GNG13 C- Serotonergic synapse

IGF1 N + FGFR2 C- Prostate cancer,PI3K-Akt signaling pathway

IGF1 N + KIT C- PI3K-Akt signaling pathway

IGF1 N + FGFR3 C- PI3K-Akt signaling pathway

IGF1 N + TEK C- PI3K-Akt signaling pathway

IGF1R N + IRS1 C+ PI3K-Akt signaling pathway

IGF1R N + SHC1 C+ Glioma,Glioma,Focal adhesion

IGF1R N + NRAS C+ Melanoma

IGF1R N + PTK2 C+ Focal adhesion

INS N + FGFR3 C- PI3K-Akt signaling pathway

INS N + KIT C- PI3K-Akt signaling pathway

INS N + FGFR2 C- Prostate cancer,PI3K-Akt signaling pathway

INS N + TEK C- PI3K-Akt signaling pathway

KITLG N + KIT C- Cytokine-cytokine receptor interaction,PI3K-Akt signaling pathway,Pathways in cancer,Melanogenesis

KITLG N + FGFR2 C- PI3K-Akt signaling pathway

KITLG N + TEK C- PI3K-Akt signaling pathway

KITLG N + FGFR3 C- PI3K-Akt signaling pathway

LYN DE + FCER1G C+ Fc epsilon RI signaling pathway

LYN DE + FCGR2A C+ Fc gamma R-mediated phagocytosis

LYN DE + SHC1 C+ Chemokine signaling pathway

LYN DE + PTK2 C+ Chemokine signaling pathway

MAP3K1 DE + MAPK13 C- RIG-I-like receptor signaling pathway

MAP3K1 DE + MAP2K6 C- GnRH signaling pathway

MAPK1 N + JMJD7-PLA2G4B C- VEGF signaling pathway,Fc epsilon RI signaling pathway,Long-term depression

MAPK1 N + MKNK2 C- Insulin signaling pathway,HIF-1 signaling pathway

MAPK1 N + PLA2G4F C- VEGF signaling pathway,Fc epsilon RI signaling pathway,Fc gamma R-mediated phagocytosis,Long-term depression

MAPK1 N - NRAS C+ Neurotrophin signaling pathway

MAPK1 N + RPS6KA6 C- Long-term potentiation,Progesterone-mediated oocyte maturation,Oocyte meiosis,Neurotrophin signaling pathway

MAPK3 DE + PLA2G4F C- VEGF signaling pathway,Fc epsilon RI signaling pathway,Fc gamma R-mediated phagocytosis,Long-term depression

MAPK3 DE + RPS6KA6 C- Long-term potentiation,Progesterone-mediated oocyte maturation,Oocyte meiosis,Neurotrophin signaling pathway

MAPK3 DE - NRAS C+ Neurotrophin signaling pathway

MAPK3 DE + MKNK2 C- Insulin signaling pathway,HIF-1 signaling pathway

MAPK3 DE + JMJD7-PLA2G4B C- VEGF signaling pathway,Fc epsilon RI signaling pathway,Long-term depression

MET DE + PTK2 C+ Focal adhesion

MET DE + SHC1 C+ Focal adhesion

MET DE + NRAS C+ Melanoma

MET DE + IRS1 C+ PI3K-Akt signaling pathway

NGF N + FGFR2 C- PI3K-Akt signaling pathway

NGF N + TEK C- PI3K-Akt signaling pathway

NGF N + KIT C- PI3K-Akt signaling pathway

NGF N + NTRK2 C- MAPK signaling pathway

NGF N + FGFR3 C- PI3K-Akt signaling pathway

PDGFD DE + KIT C- PI3K-Akt signaling pathway

PDGFD DE + TEK C- PI3K-Akt signaling pathway

PDGFD DE + FGFR3 C- PI3K-Akt signaling pathway

PDGFD DE + FGFR2 C- Prostate cancer,PI3K-Akt signaling pathway

PF4 N + CXCR2 C- Chemokine signaling pathway

PF4 N + CCR2 C- Chemokine signaling pathway

PF4 N + CX3CR1 C- Chemokine signaling pathway

PF4V1 N + CXCR2 C- Chemokine signaling pathway

PF4V1 N + CCR2 C- Chemokine signaling pathway

PF4V1 N + CX3CR1 C- Chemokine signaling pathway

PIK3CA N + RAC2 C+ Fc epsilon RI signaling pathway,Natural killer cell mediated cytotoxicity

PIK3CA N + NRAS C+ Chemokine signaling pathway

PIK3CA N + PTK2 C+ Chemokine signaling pathway

PIK3CB N + NRAS C+ Chemokine signaling pathway

PIK3CB N + PTK2 C+ Chemokine signaling pathway

PIK3CB N + RAC2 C+ Fc epsilon RI signaling pathway,Natural killer cell mediated cytotoxicity

PIK3CD N + RAC2 C+ Fc epsilon RI signaling pathway,Natural killer cell mediated cytotoxicity

PIK3CD N + NRAS C+ Chemokine signaling pathway

PIK3CD N + PTK2 C+ Chemokine signaling pathway

PIK3CG N + RAC2 C+ Fc epsilon RI signaling pathway,Natural killer cell mediated cytotoxicity

PIK3CG N + NRAS C+ Chemokine signaling pathway,Cholinergic synapse

PIK3CG N + PTK2 C+ Chemokine signaling pathway

PPBP N + CXCR2 C- Cytokine-cytokine receptor interaction,Chemokine signaling pathway

PPBP N + CX3CR1 C- Chemokine signaling pathway

PPBP N + CCR2 C- Chemokine signaling pathway

PPP1CA N - CALML5 C- Insulin signaling pathway

PPP1CA N - CALML3 C- Insulin signaling pathway

PPP1CA N - CALML6 C- Insulin signaling pathway

PPP1R3B N - CALML3 C- Insulin signaling pathway

PPP1R3B N - CALML6 C- Insulin signaling pathway

PPP1R3B N - CALML5 C- Insulin signaling pathway

PRKACA N + ADCY6 C- Cholinergic synapse

PRKACA N + CFTR C- Bile secretion

PRKACA N - GLI3 C+ Hedgehog signaling pathway

PRKACA N + MAPK13 C- Dopaminergic synapse

PRKACA N + MAPK3 C- Long-term potentiation,Cholinergic synapse

PRKACG N - GLI3 C+ Hedgehog signaling pathway

PRKACG N + ADCY6 C- Cholinergic synapse

PRKACG N + MAPK13 C- Dopaminergic synapse

PRKACG N + MAPK3 C- Long-term potentiation,Cholinergic synapse

PRKACG N + CFTR C- Bile secretion

PRKX N - GLI3 C+ Hedgehog signaling pathway

PRKX N + CFTR C- Bile secretion

PRKX N + MAPK3 C- Long-term potentiation,Cholinergic synapse

PRKX N + ADCY6 C- Cholinergic synapse

PRKX N + MAPK13 C- Dopaminergic synapse

PTK2 DE + PXN C+ Chemokine signaling pathway,Focal adhesion

PTK2 DE + SHC1 C+ Focal adhesion

RAC3 N + MAP2K6 C- Fc epsilon RI signaling pathway

RAC3 N + MAP3K1 C- MAPK signaling pathway

RAC3 N + ABLIM2 C- Axon guidance

RAC3 N + ABLIM1 C- Axon guidance

RNF125 DE - DDX58 C+ RIG-I-like receptor signaling pathway

RNF125 DE - IFIH1 C+ RIG-I-like receptor signaling pathway

SDC4 DE + PTK2 C+ Proteoglycans in cancer

SDC4 DE + PXN C+ Proteoglycans in cancer

SOCS2 N - IL13RA2 C+ Jak-STAT signaling pathway

SOCS2 N - IL12RB2 C+ Jak-STAT signaling pathway

SOCS2 N - IL2RA C+ Jak-STAT signaling pathway

SOCS2 N - IL7R C+ Jak-STAT signaling pathway

SOCS2 N - IRS1 C+ Type II diabetes mellitus

SOCS2 N - IFNAR2 C+ Jak-STAT signaling pathway

SOCS2 N - OSMR C+ Jak-STAT signaling pathway

SRC N + MMP9 C+ Estrogen signaling pathway

SRC N + EGFR C+ GnRH signaling pathway

SRC N + SHC1 C+ Chemokine signaling pathway

SRC N + PTK2 C+ Proteoglycans in cancer,ErbB signaling pathway,Chemokine signaling pathway

SRC N + NRAS C+ Estrogen signaling pathway
