# Supplementary material for: Potential Therapeutic Targets for Oral Cancer: ADM, TP53, EGFR, LYN, CTLA4, SKIL, CTGF, CD70
Source: PLoS One. 2014 Jul 16;9(7):e102610. doi: 10.1371/journal.pone.0102610 (PMC4110113; doi:10.1371/journal.pone.0102610)
Supplement: Text S7 — Connectivity information of genes in Causal Network. File contains connectivity information of gene(s) in causal network generated on the basis of causal relationships mentioned in Text S5. It contains following columns: (i) Symbol→NCBI gene symbol of a constituting node or gene of the causal network; (ii) Connectivity→Total no. of the neighboring directly connected genes based on causal relationship. (DOCX) [file pone.0102610.s007.docx]

Symbol Connectivity

"RPS6KA6" 2

"COL4A6" 2

"IL7R" 1

"F2RL1" 1

"CD44" 5

"HCK" 3

"OSMR" 1

"PTK2" 14

"ITGA5" 1

"CCL21" 3

"CALML3" 5

"GNG8" 3

"CCL26" 3

"COL4A1" 2

"SOCS2" 7

"JMJD7-PLA2G4B" 7

"EFNA2" 5

"GNAQ" 5

"CXCL14" 3

"IL13RA2" 1

"IGF1" 4

"COL4A2" 2

"IFNAR2" 1

"CCL23" 3

"EFNA4" 5

"FGF2" 4

"GNG2" 3

"PLA2G3" 1

"PIK3CB" 3

"CX3CR1" 19

"CXCL12" 3

"CNGB1" 3

"FCGR2A" 2

"CALML5" 5

"INS" 4

"PXN" 4

"FLT1" 3

"RAPGEF1" 2

"CCL16" 3

"PIK3CD" 3

"FGF13" 4

"CX3CL1" 3

"GLI3" 3

"MKNK2" 2

"EFNA5" 5

"IGF1R" 4

"CXCR2" 19

"F2RL2" 1

"FGF3" 4

"CCR2" 19

"PDGFD" 4

"IRS1" 5

"MET" 4

"ITGB1" 1

"FGF18" 4

"CCL14" 3

"NGF" 5

"MAPK3" 20

"PPP1R3B" 3

"CHP2" 3

"SDC4" 7

"IL2RA" 1

"ABLIM2" 1

"FGF8" 4

"COL5A2" 2

"CRK" 3

"PPBP" 3

"CCL28" 3

"PLA2G4F" 7

"FGF6" 4

"ITGAV" 1

"FGFR2" 34

"FGF22" 4

"CRKL" 3

"GNAI2" 5

"CCL22" 3

"SHC1" 8

"FGF20" 4

"KIT" 34

"FGFR3" 34

"LYN" 4

"DUSP6" 2

"MAP3K1" 3

"EPHA7" 4

"FGF11" 5

"ABL2" 2

"MAPK13" 16

"DDX58" 1

"NTRK2" 1

"FGF1" 4

"NOS1" 3

"CD274" 1

"RNF125" 2

"CALML6" 5

"PLCB4" 11

"MAPK1" 5

"CCL2" 3

"CCL1" 3

"TEK" 34

"CD80" 2

"PRKACA" 5

"RAC2" 4

"HGF" 4

"HTR1B" 2

"PRKACG" 5

"IFIH1" 1

"EFNA3" 5

"PLA2G1B" 1

"ADCY6" 4

"GNG3" 3

"IL12RB2" 1

"PRKX" 5

"GNAZ" 2

"FGF14" 4

"GZMA" 2

"FGF5" 4

"CFTR" 3

"PIK3CG" 3

"GPC1" 3

"FGF4" 4

"GNAS" 3

"NRAS" 10

"ANGPT1" 4

"FGF7" 4

"CCL17" 3

"CCL19" 3

"GNG4" 3

"ABLIM1" 1

"EGFR" 5

"FGF12" 5

"CCL25" 3

"PF4V1" 3

"FGF23" 5

"SRC" 5

"GNG11" 3

"FN1" 5

"DUSP4" 2

"CCL15" 3

"FGF17" 4

"GNG7" 4

"PIK3CA" 3

"FGF10" 4

"FGF19" 4

"GNB3" 3

"PPP1CA" 3

"FGF21" 4

"RAC3" 4

"CTLA4" 1

"PF4" 3

"ANGPT4" 4

"MMP9" 1

"FCER1G" 1

"FGF9" 4

"FIGF" 4

"GNG13" 4

"MAP2K6" 2

"GNAO1" 5

"KITLG" 4
