# Supplementary material for: Potential Therapeutic Targets for Oral Cancer: ADM, TP53, EGFR, LYN, CTLA4, SKIL, CTGF, CD70
Source: PLoS One. 2014 Jul 16;9(7):e102610. doi: 10.1371/journal.pone.0102610 (PMC4110113; doi:10.1371/journal.pone.0102610)
Supplement: Text S9 — Statistics related with mining of PubMed articles. File contains search statistics in following main columns: (i) GeneSymbol→NCBI gene symbol; (ii) DE Information (columns:LogFC.Adjusted_pvalue) →Differential expression data for the concerned gene; (iii) CancerType→Source of annotation for the concerned gene, ‘MN’ means that annotations for this gene was inferred from articles related with mouth neoplasm or oral cancer, whereas ‘C’ means that annotations are not specific to oral cancer and were inferred using generic term ‘neoplasms’ or cancer; (iv) TotalHits→Total no. of articles in PubMed satisfying the search criteria; (v) QualifiedHits→No. of articles which were considered to be relevant by text mining logic mentioned in the paper; (vi) Marker (columns:Therapeutic.Diagnostic) →Total no. of articles used to infer that a gene can be used as a particular marker type(s) (therapeutic/prognostic/diagnostic); (vii) Cancer Hallmark (columns:Angiogenesis.Inflammation)→Total no. of articles used to infer that a gene is associated with a particular cancer hallmark(s). (DOCX) [file pone.0102610.s009.docx]

GeneSymbol LogFC pvalue Adjusted_pvalue CancerType TotalHits QualifiedHits Therapeutic Prognostic Diagnostic Angiogenesis Apoptosis Metastasis CellProliferation ImmunoModulation Immortalization Inflammation

MMP1 5.87283519450917 2.26742293704711e-45 1.43051713098302e-41 MN 23 4 0 0 0 0 1 4 0 0 0 0

MMP10 4.77039966203701 9.42203436373742e-25 5.57283888757682e-22 MN 6 2 0 0 0 0 0 2 0 0 0 0

MMP3 4.49248388034797 1.2897333904135e-23 6.9745096801018e-21 MN 21 6 0 0 0 0 0 5 1 0 0 1

CXCL11 4.33833652091202 4.57855283263818e-18 7.16184045151593e-16 C 71 7 3 0 0 3 1 0 1 0 0 1

CXCL10 3.55075451220976 1.92667122843639e-18 3.3151005764196e-16 C 327 38 10 3 2 9 4 9 11 0 0 1

PTHLH 3.05030905983481 7.07136642099383e-25 4.46132507500501e-22 MN 20 7 1 1 0 0 0 6 1 0 0 0

INHBA 3.0315773513014 2.41645529802468e-30 3.51817303274717e-27 C 36 2 0 1 0 0 0 0 1 0 0 0

NELL2 3.00394653890674 1.57383600299604e-13 8.27444278575167e-12 MN 1 1 1 0 0 1 0 0 0 0 0 0

RSAD2 2.86254184730827 1.89172680247093e-22 7.4593152479932e-20 C 2 0 0 0 0 0 0 0 0 0 0 0

S100A7A 2.82895894477586 1.4235877663706e-10 3.67087815450903e-09 C 4 1 1 0 0 0 0 0 0 0 0 1

IFIT3 2.74069597090459 1.56797548574275e-22 6.45153739535936e-20 MN 3 1 0 1 0 0 0 0 0 0 0 0

POPDC3 2.64625714326844 2.16605772650634e-14 1.41368877895123e-12 C 4 1 0 0 0 0 0 1 0 0 0 0

PLAU 2.62254432089499 7.60097219760979e-37 2.87727201568321e-33 MN 53 11 0 5 0 0 0 9 0 0 0 0

MMP13 2.54951672632095 2.90734300983021e-06 2.41030578830733e-05 MN 11 3 0 2 0 0 0 1 0 0 0 0

IFIT1 2.47549189191239 2.39746091536135e-18 4.01564095088887e-16 C 51 3 2 0 0 0 0 1 0 0 0 0

IDO1 2.39667433963065 7.35519935783336e-10 1.63011543613246e-08 MN 4 1 1 0 0 0 0 0 0 0 0 0

SERPINE1 2.39388455145986 6.12200676362941e-19 1.1141463655309e-16 C 1131 65 5 21 1 4 1 33 9 0 0 3

AIM2 2.34644460462062 9.74499247584358e-21 2.56171489708738e-18 MN 1 1 0 0 0 0 0 0 1 0 0 0

PPP4R4 2.28006932571122 1.02563465126825e-14 7.61262237041341e-13 NA

SEMA3C 2.24527470874723 6.95313685253468e-23 3.37441080020318e-20 C 15 2 0 1 0 0 0 1 0 0 0 0

FLRT3 2.23378610241962 1.09055630072301e-12 4.66990024972499e-11 C 2 0 0 0 0 0 0 0 0 0 0 0

GBP5 2.20650607904761 5.20617397636076e-17 6.44034345428628e-15 C 1 0 0 0 0 0 0 0 0 0 0 0

IL8 2.17310640004473 1.0997999373175e-10 2.91948294721014e-09 MN 28 2 1 0 0 1 0 1 0 0 0 0

TGFBI 2.14256642936308 8.69361514026187e-22 2.78888226711418e-19 C 73 8 0 2 1 0 1 3 2 0 0 0

SPP1 2.09519440005058 2.84867925971256e-13 1.40408730074426e-11 MN 13 3 0 1 0 0 0 1 1 0 0 0

IFI44L 2.0753843133825 4.53743795002935e-13 2.1523831599049e-11 C 1 0 0 0 0 0 0 0 0 0 0 0

MFAP5 2.06884998432105 5.45035938855082e-08 7.43755963569584e-07 C 3 0 0 0 0 0 0 0 0 0 0 0

SPINK6 2.06593382626014 4.0674131496744e-05 0.000233638630300113 C 7 2 1 0 0 0 0 2 0 0 0 0

LAMC2 2.01659151725905 3.6103455377657e-19 6.97275612176443e-17 MN 29 1 0 1 0 0 0 1 0 0 0 0

MMP7 2.0096290272713 7.46463171983292e-07 7.38156136683792e-06 MN 17 7 0 1 0 2 0 3 1 0 0 0

MIR31HG 2.00669531228185 7.0451549884801e-14 4.04339450884649e-12 C 4 1 0 0 0 0 0 0 0 1 0 0

MUCL1 1.97646814393208 2.83168401538652e-05 0.000172055484299264 C 15 5 1 2 2 0 0 2 0 0 0 0

PDCD1LG2 1.95593288127262 6.00211129931344e-16 5.70864123427666e-14 C 53 4 3 1 0 0 0 0 1 0 0 0

CD274 1.93848927822907 2.45256378181748e-16 2.53659424581746e-14 C 225 21 9 4 0 0 4 3 3 0 0 0

IFIT2 1.92782904145693 1.24922333372183e-13 6.67911018004324e-12 C 33 2 1 0 0 0 0 1 1 0 0 0

IL20 1.90664109435504 3.31065479963545e-12 1.26587400793334e-10 C 14 4 0 0 0 1 2 1 0 0 0 0

GALNT6 1.90183822940269 2.01423886646384e-23 1.05898608404336e-20 C 6 3 1 1 0 0 0 0 1 0 0 0

S100A7 1.90074050108546 7.48379966923924e-08 9.88456917932248e-07 MN 5 2 0 0 0 0 0 1 0 0 0 1

IFI35 1.90056489216424 1.74182976971226e-20 4.3378436909663e-18 C 1 0 0 0 0 0 0 0 0 0 0 0

FST 1.89076271825852 2.9203905100145e-22 1.0838084545695e-19 C 130 14 0 0 2 1 2 2 8 0 0 1

TNC 1.88059483480868 3.36561848972572e-22 1.20190681424601e-19 MN 44 7 0 1 1 0 0 5 1 0 0 1

DDX60 1.86678028167991 2.06951608060184e-14 1.3553540089118e-12 C 1 0 0 0 0 0 0 0 0 0 0 0

OAS2 1.86128610597224 1.38216517033279e-17 2.01232616760682e-15 C 2 0 0 0 0 0 0 0 0 0 0 0

CXCL9 1.86113707376204 9.35091263973497e-12 3.22964823963985e-10 MN 4 1 0 0 0 0 1 0 0 0 0 0

SAMD9L 1.84325831505054 1.88583327278253e-15 1.63730120889701e-13 C 2 1 0 0 0 0 0 0 1 0 0 0

OASL 1.84244670919812 2.68561469228701e-14 1.71725098922014e-12 C 2 0 0 0 0 0 0 0 0 0 0 0

IFI6 1.82043781736649 1.46502890517398e-19 3.11557326834022e-17 C 11 3 0 0 0 0 3 0 0 0 0 0

TNFRSF12A 1.80192314803269 3.03462477352702e-26 2.29745372354184e-23 C 49 15 7 2 1 0 2 8 1 0 0 0

IL24 1.79991317206366 3.18627259287004e-11 9.52710606086116e-10 C 181 6 3 0 0 1 2 0 1 0 0 0

DDX58 1.77562947852987 3.74420403179687e-18 6.05697006066832e-16 C 34 3 1 0 0 0 3 0 0 0 0 0

PTPRZ1 1.77438575566814 1.82271161125319e-16 1.97134072378224e-14 C 21 2 0 0 0 0 0 1 1 0 0 0

KIF14 1.77277373692575 4.36816679582824e-19 8.18577157867733e-17 C 15 6 3 1 1 0 1 2 1 0 0 0

SAA1 1.74057350664318 6.93661141375892e-09 1.1999061064366e-07 C 122 3 0 0 0 0 0 2 0 0 0 1

CA9 1.72622800543231 1.10929826991006e-08 1.80220500897748e-07 MN 83 3 1 2 0 0 0 1 0 0 0 0

PARP12 1.70879599319907 2.60376524089325e-33 6.16018308929831e-30 NA

DUSP6 1.70112693229154 4.45806545069481e-22 1.56255194046853e-19 C 44 6 0 0 0 0 4 1 1 0 0 1

DCBLD1 1.69489159287581 3.44693907215779e-26 2.50923906995117e-23 NA

TREM2 1.68115391410617 1.02961940302536e-13 5.61602491096861e-12 C 1 0 0 0 0 0 0 0 0 0 0 0

COL4A1 1.66015724347717 2.25704919524292e-27 1.94178045992558e-24 MN 2 1 0 0 0 0 0 1 1 0 0 0

MB21D1 1.65211971140237 2.82580762589218e-23 1.44551516041247e-20 NA

LINC00491 1.6371551920272 1.02262621338294e-08 1.68892709160201e-07 NA

IL12RB2 1.63607011946859 2.14163204846272e-07 2.49290712061832e-06 C 2 1 0 0 0 0 0 0 1 0 0 0

HMGA2 1.63416804972755 3.92338269308155e-11 1.13892429803611e-09 MN 10 2 0 2 1 0 0 0 0 0 0 0

CAV1 1.62966391556925 1.24765517581389e-10 3.27977354342075e-09 MN 4 2 0 1 1 0 0 0 0 0 0 0

CTSC 1.6155872031416 1.61229631689165e-28 1.60610170472675e-25 C 110 1 0 0 0 0 1 1 0 0 0 0

ITGA3 1.61145692647374 6.10061317017676e-18 9.38750450991346e-16 C 45 2 0 0 1 0 0 1 0 0 0 0

CA2 1.60748747411299 5.9543292188489e-13 2.72216398853027e-11 NA

LTBP1 1.60316445871773 5.60441933712903e-18 8.69465940933124e-16 C 13 1 0 0 1 0 0 1 0 0 0 0

TREM1 1.60295552085489 3.4079575440395e-10 8.06280155450445e-09 C 14 2 0 1 0 0 0 0 0 0 0 1

ARSJ 1.59366141292519 9.07008393413479e-17 1.06627005354888e-14 C 1 0 0 0 0 0 0 0 0 0 0 0

LY6E 1.57220455003779 7.96106587355219e-14 4.52489771137304e-12 C 23 2 0 0 0 0 1 0 1 0 0 0

SLCO1B3 1.56167267135366 4.6955586414892e-06 3.65431078978068e-05 C 14 1 0 0 0 0 1 0 0 0 0 0

CYP27B1 1.56009579037416 8.69833228516891e-12 3.03751540888177e-10 C 465 4 0 0 0 0 1 1 2 0 0 0

LOC100506305 1.55969287656673 6.61684112574057e-14 3.81819975569792e-12 NA

TLR2 1.55506395772161 7.79408434925091e-16 7.26692780681143e-14 MN 6 2 0 0 0 0 2 0 0 0 0 0

LAMA3 1.55432369368182 1.07649915663965e-13 5.83808009676754e-12 C 13 2 0 2 0 0 0 1 0 0 0 0

IL2RA 1.54887256529945 3.74222666738802e-13 1.80226778966038e-11 C 1498 62 20 2 3 0 6 12 25 0 0 1

DFNA5 1.54627147810128 2.19819829444563e-20 5.40328559986656e-18 C 8 3 0 0 0 0 1 1 1 0 0 0

OAS3 1.54377073149495 4.59760802162947e-17 5.80126180169206e-15 C 56 2 0 0 0 0 1 0 1 0 0 0

HOTAIRM1 1.54346755153173 4.7372396494546e-20 1.09343579079545e-17 NA

HOXC13 1.52915305513889 3.3187028106724e-13 1.6230772118242e-11 C 4 0 0 0 0 0 0 0 0 0 0 0

MET 1.5098179090927 1.18121903550997e-22 5.38813579758399e-20 MN 50 8 0 3 0 1 0 6 1 0 0 0

WARS 1.50682465900933 9.16793682183342e-14 5.08860821779593e-12 C 99 2 0 0 0 1 0 1 0 0 0 0

TNFAIP3 1.50410072840269 5.73576414197854e-14 3.34033255123778e-12 MN 3 2 0 0 1 0 0 1 0 0 0 0

NCF2 1.50272947286577 2.44237881501423e-22 9.24538076635488e-20 C 14 1 0 1 0 0 0 0 0 0 0 1

XAF1 1.50188660612361 4.64584243556301e-17 5.82330197204643e-15 C 65 15 4 5 3 0 7 1 0 0 0 0

IFI44 1.49448817288402 4.86018045306719e-14 2.8836562832352e-12 C 380 10 1 0 0 0 1 4 8 0 0 0

PDPN 1.48996131225998 2.41317507038806e-14 1.56418371771352e-12 MN 27 8 0 0 1 0 0 8 0 0 0 0

TAP1 1.48543211244276 3.56968495906863e-23 1.77798492684979e-20 C 91 8 2 1 0 0 1 2 3 0 0 1

CCL20 1.48218614252396 1.07490607149435e-07 1.35541287242996e-06 MN 5 1 0 1 0 0 0 1 0 0 0 0

PFN2 1.48097426439095 1.59925352605029e-10 4.08069922101908e-09 MN 1 1 0 1 0 0 0 0 0 0 0 0

ICAM1 1.45511612046245 4.88545363161044e-12 1.78852961093793e-10 MN 16 1 0 0 0 0 0 1 0 0 0 0

AMIGO2 1.45483882507275 3.76253741248088e-16 3.72338498860996e-14 C 3 0 0 0 0 0 0 0 0 0 0 0

ARL14 1.45436913262995 2.7472229264192e-07 3.11917746420733e-06 NA

CEP55 1.45287657713666 1.58720267492265e-16 1.7671167663683e-14 C 8 3 2 0 1 0 0 0 0 0 0 0

PLAC1 1.45043909282192 3.51433640382844e-11 1.03607235382026e-09 C 8 1 1 0 0 0 0 0 0 0 0 0

EIF2AK2 1.44647479616161 2.07535924365143e-21 6.13755068821727e-19 C 181 20 5 0 0 0 12 0 5 0 0 1

CDH3 1.4462405526067 4.1943632986223e-12 1.57201414164404e-10 C 66 2 0 0 0 0 0 2 0 0 0 0

ITGA1 1.43786424776848 3.04757438254897e-16 3.08456900205906e-14 C 40 2 0 1 0 0 0 1 0 0 0 0

ITGA5 1.4370705391871 2.72752493241706e-14 1.73817725238578e-12 C 173 4 0 0 0 0 0 3 2 0 0 0

MKI67 1.4354300499109 1.76347312150164e-15 1.54524332271581e-13 C 176 11 0 1 1 0 0 1 10 0 0 0

APOL1 1.43121097425108 3.53897669408198e-12 1.34773062150684e-10 C 4 0 0 0 0 0 0 0 0 0 0 0

TPM1 1.42454329910134 1.26708259569814e-13 6.75551332078274e-12 MN 1 1 0 1 0 0 1 0 0 0 0 0

PI15 1.42207934015317 3.26995538756763e-13 1.60337942022001e-11 C 2 0 0 0 0 0 0 0 0 0 0 0

TCN1 1.41030410794967 0.00296090394795293 0.00860580912513899 NA

SLC16A1 1.40852908688239 2.70711742856556e-21 7.76327448037277e-19 MN 1 1 1 0 0 0 0 0 0 0 0 0

CDK6 1.40293174305001 1.38517715036407e-22 5.95846543748654e-20 MN 12 1 0 0 0 0 0 0 1 0 0 0

FEZ1 1.40067807399553 2.36661360107141e-12 9.43008328999548e-11 C 11 3 0 1 0 0 0 0 2 0 0 0

NRG1 1.3944559830727 2.59551181117194e-15 2.19309160937729e-13 C 247 13 0 1 1 1 0 3 8 0 0 0

LY6K 1.39438076238718 1.19861079393064e-14 8.65881927355925e-13 C 11 3 1 0 1 0 0 2 0 0 0 0

FCGR3B 1.39170795533353 8.07861908962841e-10 1.76972249432172e-08 MN 3 1 0 0 0 0 1 0 0 0 0 0

MELK 1.38904867451686 3.14382833306948e-11 9.42999031061901e-10 C 24 5 4 1 0 0 0 1 3 0 0 0

CDC42EP3 1.38733441776463 1.01068918107594e-21 3.13595313610236e-19 C 4 0 0 0 0 0 0 0 0 0 0 0

SLC28A3 1.3841137472945 2.06451623138254e-10 5.11454171614887e-09 C 2 0 0 0 0 0 0 0 0 0 0 0

IFI30 1.37951434397638 1.24718540164619e-17 1.87345064261567e-15 C 10 0 0 0 0 0 0 0 0 0 0 0

IRS1 1.37197110357085 4.38266639688299e-15 3.52981816569381e-13 C 315 17 1 0 2 1 3 4 11 0 0 0

SCG5 1.36764758671563 2.07194078594562e-08 3.16471374925239e-07 C 7 0 0 0 0 0 0 0 0 0 0 0

HOXD10 1.36317634943072 2.79764837329319e-11 8.56813766364405e-10 C 24 5 0 0 0 0 0 5 0 0 0 0

CD80 1.36289795303033 4.51778376209228e-15 3.62322429089494e-13 MN 6 2 2 0 0 0 0 0 0 0 0 0

BATF2 1.35637967753265 1.77807796508263e-10 4.47522362302113e-09 C 4 2 1 2 0 0 0 1 0 0 0 0

TRIML2 1.35319956064287 2.48688140090835e-05 0.00015422412934139 NA

AREG 1.3504842860329 1.46904491323852e-08 2.30552347204522e-07 C 300 26 7 2 0 2 5 5 7 0 0 0

FSCN1 1.34941644631906 2.09733387035683e-17 2.91884104148852e-15 MN 5 2 1 1 0 0 0 0 0 0 0 0

SLC15A3 1.34845646651345 1.34163458588863e-15 1.21498171325905e-13 C 2 0 0 0 0 0 0 0 0 0 0 0

NT5E 1.34498071747629 9.05897818163408e-11 2.4459241090412e-09 NA

MICAL2 1.33986233771448 5.57729984781388e-22 1.91930098581043e-19 C 2 0 0 0 0 0 0 0 0 0 0 0

MYO10 1.33631415134887 8.6262581493579e-22 2.78888226711418e-19 C 5 0 0 0 0 0 0 0 0 0 0 0

AURKA 1.33424946120094 2.52804283854525e-13 1.2658271641573e-11 MN 5 2 2 0 0 0 1 0 0 0 0 0

FN1 1.33278979609501 3.99240295570842e-09 7.39743849540581e-08 MN 84 7 0 1 1 0 0 6 0 0 0 0

KRT75 1.32872695149314 2.6406427062769e-05 0.000162218255442074 C 2 0 0 0 0 0 0 0 0 0 0 0

KYNU 1.31616178140079 6.23071004097581e-10 1.39230990490613e-08 C 1 0 0 0 0 0 0 0 0 0 0 0

PTPRK 1.30566116408324 4.35024473427958e-20 1.01930827056325e-17 C 11 0 0 0 0 0 0 0 0 0 0 0

CTLA4 1.29530888250406 1.49275621830076e-11 4.87968859132617e-10 C 621 17 9 1 0 0 4 3 5 0 0 0

LRP12 1.29430122821755 5.56445592544217e-16 5.31911400509313e-14 C 3 1 0 0 0 0 1 1 1 0 0 0

STC1 1.29004610176082 9.53041782022713e-10 2.03362139891137e-08 C 58 4 0 0 1 0 1 2 1 0 0 0

ETS1 1.28569794288655 9.13904166060652e-14 5.08748945618528e-12 MN 13 6 0 2 0 1 0 5 1 0 0 0

PLEK2 1.27921310743977 8.03648631454057e-14 4.55408911602723e-12 C 2

PMEPA1 1.27862746208286 2.62658945849034e-10 6.39812853035349e-09 C 18 3 0 0 0 0 0 1 2 0 0 0

ANGPT2 1.27703228074842 9.03900160755030e-11 2.44401690608721e-09 MN 7 4 0 1 0 3 1 0 1 0 0 0

PHLDB2 1.27592322786073 8.38033566712938e-23 3.96536532929394e-20 C 2 1 1 0 0 0 0 0 0 0 0 0

SKAP2 1.26124036941541 1.87059564706077e-22 7.4593152479932e-20 C 16 0 0 0 0 0 0 0 0 0 0 0

F3 1.26031489711805 8.94026853266472e-05 0.000461320781127986 C 967 16 3 0 0 2 3 8 2 0 0 2

WISP3 1.26014647077913 0.000215130078124838 0.00096647685465673 C 168 4 1 1 0 1 0 3 1 0 0 0

HOXC9 1.25763565820895 1.4541500535306e-08 2.28593837734e-07 C 6 2 1 0 0 0 1 0 1 0 0 0

KLHDC7B 1.25317725730769 2.97342847076727e-06 2.46078183936214e-05 NA

PDE7A 1.24890929514314 4.6547017058057e-21 1.25856484551121e-18 C 7 2 0 0 0 0 2 0 0 0 0 0

ITGB6 1.24857893130855 1.38237887832197e-11 4.56619284991272e-10 C 6 0 0 0 0 0 0 0 0 0 0 0

LIMA1 1.24832075471553 8.13312755882274e-25 4.96566791309155e-22 MN 1 1 0 0 0 0 0 0 1 0 0 0

FAIM3 1.24505126062868 4.32865053505895e-12 1.61913772089053e-10 C 8 3 0 1 0 0 2 0 1 0 0 0

FAT1 1.23866702881548 2.82890448383605e-13 1.39798107481893e-11 MN 31 5 1 0 2 0 0 3 1 0 0 0

ESM1 1.23835248552035 2.73480322175825e-10 6.62760826865792e-09 C 38 9 2 4 1 2 0 1 1 0 0 0

LYN 1.23713359957268 2.37268061516199e-16 2.4715360524864e-14 C 226 37 16 3 0 0 8 8 11 0 0 0

ART3 1.23629829554092 1.04104742018135e-07 1.31710591723077e-06 C 1 0 0 0 0 0 0 0 0 0 0 0

TRIP13 1.23490015277217 9.78934820024407e-14 5.41763138555613e-12 C 2 0 0 0 0 0 0 0 0 0 0 0

SEMA3A 1.23067790359673 9.71369017083378e-06 6.8396954562266e-05 MN 1 1 0 1 0 0 0 0 0 0 0 0

IGF2BP2 1.230268951592 1.69913425986217e-13 8.78675249628723e-12 C 183 17 1 5 1 1 7 2 4 0 0 0

TNIP3 1.23016336663026 1.14136916969449e-06 1.07050021183388e-05 C 2 0 0 0 0 0 0 0 0 0 0 0

VEGFC 1.22817974330007 9.94325164672628e-14 5.48414888943424e-12 MN 63 24 3 2 2 11 0 18 1 0 0 0

CLMP 1.22651484605691 8.49131412128338e-07 8.27149265947146e-06 C 1

CTSL1 1.22504169226008 5.39461619125335e-19 1.00101863384169e-16 MN 7 2 0 0 0 0 0 2 0 0 0 0

IFITM1 1.22233622674757 1.12788632624935e-11 3.80525926861346e-10 C 30 2 0 1 0 0 0 0 1 0 0 0

WNT7A 1.21879591158585 2.13770352768396e-10 5.26141933270148e-09 C 25 4 2 1 0 0 0 1 1 0 0 0

ANLN 1.21308824793339 6.95940428779321e-12 2.46206813000116e-10 C 10 1 0 1 0 0 0 0 0 0 0 0

PANX1 1.21262419289123 1.60138720573639e-15 1.42297913816773e-13 C 7 2 0 0 0 0 0 2 1 0 0 0

ICOS 1.21072040001706 2.46159989293349e-09 4.77363741532297e-08 C 49 6 2 0 2 0 1 1 0 0 0 0

SLFN5 1.20978993234761 1.44461366047309e-13 7.61621246567524e-12 C 3 0 0 0 0 0 0 0 0 0 0 0

HOXD11 1.20692741346593 6.58080675642792e-08 8.82117064298239e-07 C 6 0 0 0 0 0 0 0 0 0 0 0

E2F7 1.20513903232902 4.8779196779856e-15 3.89554370233053e-13 C 6 2 1 0 0 1 1 0 0 0 0 0

DKK1 1.20478552644997 0.000151682032400876 0.000720965802926012 MN 2 1 0 0 0 0 0 1 0 0 0 0

OSMR 1.20036393996236 3.79910890059745e-19 7.26320547086949e-17 C 18 1 1 1 0 0 0 0 0 0 0 0

RIPK2 1.19918702999077 1.16806016784646e-14 8.47045011372798e-13 C 469 1 0 0 0 0 0 0 1 0 0 0

ADAMTS12 1.19736809557657 8.81706878883726e-09 1.4847033893801e-07 C 4 2 0 1 0 0 0 0 1 0 0 0

NLRC5 1.19654295290625 1.33930955277819e-18 2.3690758790124e-16 C 1

IFNAR2 1.19521884953136 1.27361958656728e-14 9.06233004321762e-13 C 20 1 1 0 0 0 0 1 0 0 0 0

EPSTI1 1.1906369333211 2.33352340504022e-11 7.38571864334386e-10 C 1 0 0 0 0 0 0 0 0 0 0 0

TNFRSF10B 1.18811050730273 1.39606183243088e-16 1.57281323228686e-14 MN 41 4 0 0 0 0 3 0 1 0 0 0

OLR1 1.18794916650453 1.98558515107499e-05 0.000127914125780791 C 5 0 0 0 0 0 0 0 0 0 0 0

SOD2 1.18533900234405 1.23053935045974e-13 6.62318732468230e-12 C 260 18 4 0 0 0 11 3 5 0 0 0

ISG15 1.1817138842324 2.69265942660312e-13 1.33763687578261e-11 MN 3 1 0 1 0 0 0 0 0 0 0 0

OCIAD2 1.17915189850982 1.34054242060925e-17 1.98222237459931e-15 C 1 1 0 1 0 0 0 1 0 0 0 0

LINC00152 1.17809038781885 4.44824699247593e-21 1.22017349024046e-18 C 1

IRF1 1.17714935411081 2.91042035311583e-11 8.85619389444103e-10 C 310 24 7 3 1 0 16 1 3 0 0 1

CSF2 1.17408423811762 4.94243241135135e-05 0.00027594518657713 C 5784 10 4 0 0 0 1 2 3 0 0 0

RTP4 1.17258994967192 2.35741446121486e-10 5.77215828038987e-09 C 1 0 0 0 0 0 0 0 0 0 0 0

TPX2 1.17228634967386 7.87979450602016e-13 3.50095940411839e-11 MN 3 2 0 1 0 0 0 0 2 0 0 0

CCL3 1.17010273818246 1.93442849112021e-10 4.8174905330832e-09 C 138 8 0 1 1 0 1 2 3 0 0 0

LAMB1 1.17001038331364 3.3817623993634e-19 6.59862030234546e-17 NA

TRIB1 1.16764701235713 2.48163234876315e-15 2.11575925518199e-13 C 14 2 0 1 0 0 1 0 0 0 0 0

SNAI2 1.16598592122696 4.39713253317509e-14 2.63368757770268e-12 MN 12 5 1 0 0 0 0 5 0 0 0 0

VAV2 1.16344386470556 5.19191833492194e-15 4.07748706743019e-13 MN 1 1 0 0 0 0 0 1 0 0 0 0

ACOT9 1.16263479405883 1.01673620353123e-19 2.21192714071673e-17 C 2 0 0 0 0 0 0 0 0 0 0 0

CCNA1 1.15366784648536 0.000274694411784429 0.00118618992305166 C 73 14 2 0 1 0 3 2 7 0 0 0

MAGEA4 1.14703753972133 0.0120450270043685 0.0280482561653153 C 5 2 1 0 0 0 1 0 0 0 0 0

SERPINE2 1.14691758455394 1.23797297096252e-06 1.145775766328e-05 C 706 1 1 0 0 0 0 0 0 0 0 0

DOCK5 1.14163249105458 2.86390906337051e-17 3.84434091080947e-15 C 3 1 0 0 0 0 1 0 0 0 0 0

PDGFA 1.1378822676107 9.87678434066026e-17 1.14685826512685e-14 C 208 9 0 1 2 1 0 1 6 0 0 0

IL1RL1 1.13343244087464 4.68668262747701e-06 3.64890341794559e-05 C 54 2 0 1 0 0 0 1 0 0 0 0

HIST1H2BB 1.13338284098157 6.56181284453015e-06 4.86659215158394e-05 C 4

SNX10 1.13189574632474 2.06513093488131e-15 1.77666969111357e-13 NA

GLIPR1 1.12946617236188 4.34687285072088e-12 1.6227467937987e-10 C 10 2 1 0 0 0 1 0 0 0 0 0

PSMB9 1.12912150445977 3.67136120548571e-13 1.77718295489074e-11 C 79 11 7 0 0 0 1 2 1 0 0 1

IL32 1.12725978924169 1.57736106983729e-09 3.18960608641136e-08 C 97 36 18 1 0 14 7 18 4 0 0 0

XDH 1.1193253846997 2.76446863357265e-07 3.13687636856293e-06 MN 6 1 0 0 0 0 0 0 1 0 0 0

KIF18A 1.11445627830732 7.42882709733131e-11 2.04964155205816e-09 C 4 3 2 0 0 0 0 1 1 0 0 1

LOC100134229 1.11445282432345 8.86681377330890e-10 1.91832629034619e-08 NA

PHLDA1 1.11416788278081 2.42753385719037e-09 4.71724161345402e-08 MN 2 2 0 1 0 0 1 0 0 0 0 0

KLF7 1.11399619620983 2.99380554938351e-11 9.0791352723294e-10 NA

DNA2 1.11311945797529 5.66902631606912e-07 5.81244101214736e-06 C 2 0 0 0 0 0 0 0 0 0 0 0

ECT2 1.11303673149685 7.39921796941596e-15 5.64697574625548e-13 C 26 7 0 1 0 0 2 5 3 0 0 0

FLJ13744 1.11145852404744 2.13184759384931e-06 1.84496933739304e-05 NA

TNS4 1.11071704932182 2.42644235088353e-12 9.60779798643779e-11 C 14 4 0 2 0 0 0 2 0 0 0 1

MYO1B 1.1078355937603 2.25517102187472e-12 9.06234011274372e-11 C 2 0 0 0 0 0 0 0 0 0 0 0

IRF9 1.10619336025127 4.09101839623485e-14 2.47382444682227e-12 C 57 2 0 0 0 0 1 0 1 0 0 0

COL12A1 1.1044442978239 1.88433194362206e-07 2.23044094414852e-06 C 7 0 0 0 0 0 0 0 0 0 0 0

ITGAV 1.102289987957 8.29481444925573e-08 1.08572581660486e-06 C 438 16 2 0 0 4 1 11 2 0 0 0

PLSCR1 1.10003639467296 5.35123847591146e-14 3.15380646802967e-12 C 11 1 1 0 0 0 0 0 0 0 0 0

NDC80 1.09500493251363 3.20674947183344e-12 1.23111860554547e-10 C 314 3 2 0 0 0 1 0 2 0 0 0

MTAP 1.09305923775585 1.50028165102858e-08 2.34870395442662e-07 C 74 13 9 3 2 0 0 2 3 0 0 0

TNFRSF9 1.09168446243619 2.958045303715e-10 7.10493952581395e-09 C 104 10 7 1 0 0 1 2 3 0 0 0

FAM83A 1.09108578410304 1.09789591304824e-08 1.78982566289957e-07 C 3 2 0 2 1 0 0 0 0 0 0 0

CNGB1 1.08715675963801 2.3559704696766e-11 7.43190884659483e-10 C 5 0 0 0 0 0 0 0 0 0 0 0

ARPC1B 1.08690076267019 2.68948843347376e-18 4.42643022437894e-16 C 4 0 0 0 0 0 0 0 0 0 0 0

ANXA3 1.08345052799677 5.29753153360277e-07 5.47729386570833e-06 C 30 5 1 3 0 0 1 2 0 0 0 0

IL7R 1.08255618894354 2.88045534173878e-07 3.24128289257371e-06 C 95 8 1 0 0 0 3 1 3 0 0 0

DDX60L 1.08106172728195 4.21824366267801e-11 1.21073779885369e-09 NA

CCL18 1.08088591674369 0.000488914660202521 0.00192504426329376 C 36 8 1 0 0 0 3 3 1 0 0 1

S100P 1.08015628921161 0.000322039371825831 0.00135660787681894 MN 2 1 0 0 1 0 0 0 0 0 0 0

TRIO 1.07973021492447 4.12199793067983e-20 9.87557656126292e-18 C 29 3 0 0 0 0 0 2 2 0 0 0

HERC5 1.07968025987654 1.01432097617747e-06 9.67156328267553e-06 C 3 0 0 0 0 0 0 0 0 0 0 0

C1QC 1.07947587712642 1.30708491498586e-08 2.08067251353552e-07 C 1 0 0 0 0 0 0 0 0 0 0 0

CCL3L3 1.0781009224989 6.94191993738086e-09 1.1999061064366e-07 C 5 2 0 0 0 0 0 0 2 0 0 0

HERC6 1.07206132564718 3.41920508190874e-11 1.00959897948965e-09 NA

LOC440173 1.07173588752248 0.000246245061036298 0.00108161528666373 NA

HTR7 1.07173252590352 3.7997586626535e-11 1.10984617605004e-09 C 4 1 0 0 0 0 0 0 0 1 0 0

ACTN1 1.07126158133758 6.85321835685835e-16 6.4855431920129e-14 C 11 0 0 0 0 0 0 0 0 0 0 0

STK17A 1.06997604126615 2.38182854755045e-19 4.90009444777036e-17 C 9 1 0 0 0 0 1 0 0 0 0 0

ENTPD7 1.06920443427825 3.36726486678247e-19 6.59862030234546e-17 NA

IFIH1 1.0689075269431 3.09284086844769e-14 1.95127330390364e-12 C 25 3 0 0 0 0 3 0 0 0 0 0

NCAPH 1.06458619402048 7.08211328524995e-12 2.49614819646044e-10 C 1 0 0 0 0 0 0 0 0 0 0 0

RGS1 1.06452141182994 6.62277590931242e-05 0.00035661245984511 C 13 2 0 1 0 0 0 1 0 0 0 0

CHST11 1.06391034733453 1.20484479808087e-11 4.02188668311758e-10 C 1 0 0 0 0 0 0 0 0 0 0 0

BUB1B 1.06290744964195 5.49079301396493e-12 1.99088581178763e-10 MN 2 1 0 1 0 0 0 0 0 0 0 0

TMEM2 1.06284541175159 3.57143777002882e-12 1.35736149946457e-10 C 3

STAT2 1.05960501044624 3.58540763526983e-18 5.8500870959269e-16 C 51 3 1 0 0 0 0 0 2 0 0 0

PTGFRN 1.05948594316417 5.6844275644371e-08 7.74022737497129e-07 C 8 0 0 0 0 0 0 0 0 0 0 0

SDC4 1.05873430399548 1.07173133027292e-09 2.25887070023113e-08 C 46 5 0 0 0 0 2 3 0 0 0 0

DTX3L 1.05809551215841 2.98324017377968e-12 1.15704481084279e-10 C 2

PARP9 1.05373611946229 2.23341657096795e-18 3.774274592742e-16 NA

UBE2L6 1.0529319558296 7.08558527783261e-16 6.6720832116188e-14 C 4 0 0 0 0 0 0 0 0 0 0 0

PARP14 1.05292367000818 1.01254585324138e-12 4.37544643020541e-11 C 2 0 0 0 0 0 0 0 0 0 0 0

CCL4 1.04956637595404 1.69383874586769e-10 4.28600079452376e-09 C 151 8 2 0 1 0 1 3 2 0 0 1

DLGAP5 1.04680069150244 6.52719789115724e-13 2.96259651045403e-11 C 9 0 0 0 0 0 0 0 0 0 0 0

MICALCL 1.04532612790855 1.02351105514213e-08 1.68892709160201e-07 NA

MOB3B 1.04323565713375 8.49611743374642e-14 4.75757439847688e-12 C 1 0 0 0 0 0 0 0 0 0 0 0

C1QB 1.04082553058653 4.57241295041079e-08 6.36338675826655e-07 C 1 0 0 0 0 0 0 0 0 0 0 0

ARHGAP11A 1.03858323466381 1.05211898789615e-10 2.8007673817033e-09 NA

LOC541471 1.03795783147738 1.92677420115946e-16 2.06034210764661e-14 NA

PI3 1.03696003204281 0.0010911654244463 0.00375226889325858 MN 10 1 0 0 0 0 1 0 0 0 0 0

NRIP1 1.0352441765814 9.88263150471861e-10 2.10166928640235e-08 C 7 2 1 0 0 0 0 1 1 0 0 0

LGALS1 1.03367684375028 3.8388992058805e-10 8.99243134525992e-09 MN 8 3 0 0 0 0 0 3 0 0 0 0

IFI27 1.03099682537442 2.79014893998704e-15 2.34707328831709e-13 MN 79 24 6 9 1 0 4 5 6 0 0 0

BST2 1.02721836860846 1.27401129123249e-15 1.16488945454866e-13 C 17 3 1 0 0 0 0 2 0 0 0 0

FOXM1 1.02443360091912 3.42516315312871e-14 2.13250207234431e-12 C 276 95 31 14 3 5 19 19 30 0 1 2

KCTD14 1.02361836885049 0.000273797010005136 0.00118314064117973 NA

CCL13 1.02330516095383 0.000189629234952933 0.000870378767584849 C 15 2 0 0 0 0 1 1 0 0 0 1

RTP3 1.02294608006455 0.00195932863969551 0.00613164900190425 C 2 1 1 0 0 0 0 1 1 0 0 0

TXNRD1 1.02237361474907 8.86847098881448e-10 1.91832629034619e-08 MN 6 1 1 0 0 0 0 1 0 0 0 0

P4HA1 1.02216465550892 1.93297160154963e-09 3.83494271514987e-08 C 1 0 0 0 0 0 0 0 0 0 0 0

ATP6V1C1 1.02038968726548 4.27434381081122e-15 3.45728655159077e-13 MN 2 1 0 0 1 0 0 0 0 0 0 0

SAMD9 1.01859768683841 3.52079255525213e-07 3.8541376919177e-06 C 2 0 0 0 0 0 0 0 0 0 0 0

ATAD2 1.01854966467789 8.42855789767262e-12 2.94874889702865e-10 C 18 5 4 1 0 0 0 0 2 0 0 0

RAD51 1.0166605750821 3.04490883576548e-10 7.2766400927441e-09 C 198 22 11 4 2 0 9 2 4 0 0 0

FMNL2 1.0141450179591 1.26720856015977e-16 1.45360341928146e-14 C 8 3 0 0 0 0 0 3 0 0 0 0

CALU 1.0132329899228 6.47130282990897e-13 2.9442872274444e-11 C 200 0 0 0 0 0 0 0 0 0 0 0

OSGIN2 1.01200118562636 1.45836042052072e-16 1.6332773774672e-14 C 1

PLAUR 1.01073076057025 2.16235411241216e-08 3.28994182360329e-07 MN 20 7 2 2 0 0 0 6 1 0 0 0

MSC 1.01000168784906 3.2323771944575e-06 2.65419536483719e-05 NA

CFB 1.00802989106055 4.9653499389637e-08 6.82988214351497e-07 C 22 1 0 0 0 0 1 0 0 0 0 0

ADM 1.00531975068803 4.33955349061627e-10 1.00655305045213e-08 C 722 21 4 1 1 8 4 5 6 0 0 2

ETV4 1.0035886428793 2.12291859834746e-10 5.23865453858179e-09 MN 5 5 0 0 0 0 0 5 0 0 0 0

MMP9 0.998012607507152 6.40859868370028e-07 6.44210869106631e-06 MN 161 45 5 6 2 1 3 38 0 0 0 0

PLA2G7 0.997320535502731 1.55858549540912e-09 3.15501044616132e-08 C 22 2 0 0 0 0 0 0 1 0 0 1

GBP1 0.99718650024625 4.16732614305374e-06 3.30297244177463e-05 C 18 1 1 0 0 0 0 1 0 0 0 0

TTK 0.995137309278391 3.31066471258504e-07 3.64731961671113e-06 C 33 2 1 0 0 0 0 0 1 0 0 0

IL13RA2 0.994538200539485 0.00400194343181994 0.0110900122011795 C 54 3 3 0 0 0 0 0 0 0 0 0

APBB2 0.993385270338043 1.16435541584977e-16 1.34376554608467e-14 C 2

PML 0.993337274071326 2.34287926830407e-13 1.17935308274445e-11 C 1950 110 31 7 2 0 61 3 20 0 0 0

SPRY4 0.991093760668702 6.17326511641215e-14 3.58409168277094e-12 C 11 1 0 0 0 0 0 1 0 0 0 0

MX2 0.990614764550512 3.08795707411582e-07 3.4403263988855e-06 C 25 1 1 0 0 0 0 0 0 0 0 0

HCP5 0.990001507782026 7.04982399703778e-14 4.04339450884649e-12 C 2

GPR39 0.989243248805824 1.59930562534236e-13 8.36926561974117e-12 C 3 0 0 0 0 0 0 0 0 0 0 0

HLA-A 0.988677648450668 1.59976690547692e-10 4.08069922101908e-09 MN 1 1 0 1 0 0 0 0 0 0 0 0

EXO1 0.988287761850426 1.8509080139787e-10 4.62775904631108e-09 C 10 1 0 0 0 0 1 0 0 0 0 0

CDC25B 0.986405957272854 1.53167199049507e-10 3.93350824478971e-09 C 96 16 4 5 2 0 3 2 3 0 0 0

SULF1 0.986386926298265 3.94741237399973e-05 0.000227574395378291 C 40 15 5 1 1 2 4 5 9 0 0 0

RTKN 0.985664346646691 1.89697329096242e-13 9.75652540164287e-12 C 10 2 1 0 0 0 0 0 1 0 0 0

RNF213 0.982058221788765 4.19603364225316e-17 5.33008917764602e-15 C 4

LGALS3BP 0.980795345960967 5.02347080876523e-12 1.83550640921814e-10 C 33 3 0 1 0 0 0 3 1 0 0 0

RBP1 0.980607263655091 0.000173562222471126 0.000807922327769553 C 75 2 0 0 0 0 0 0 2 0 0 0

FAP 0.980385393103558 9.90217232568999e-07 9.47035955575212e-06 C 518 8 1 0 0 0 0 6 2 0 0 0

STK17B 0.979910502092557 3.66548417272317e-09 6.84862970751544e-08 C 5 0 0 0 0 0 0 0 0 0 0 0

TRIM21 0.976455368538271 2.11549396420534e-11 6.75210021256569e-10 NA

ADAM12 0.975830755382605 9.85738612361704e-08 1.26061315649797e-06 MN 1 1 0 0 0 0 0 0 1 0 0 0

FBN2 0.975746774740365 1.03595681820171e-05 7.23526003620068e-05 C 246 3 1 0 1 0 0 1 0 0 0 1

SERINC2 0.9741216842963 3.14296236801036e-10 7.4638455130906e-09 C 2

TSPAN1 0.973355221123128 3.09897909393309e-07 3.44620313224862e-06 C 38 7 2 1 0 0 1 4 3 0 0 0

TTYH3 0.969048819537261 2.93946778581152e-17 3.91797935084892e-15 NA

WDR66 0.968384732667892 4.53599128286461e-13 2.1523831599049e-11 C 1 0 0 0 0 0 0 0 0 0 0 0

CCM2 0.967374532912698 1.83680611814385e-17 2.63372949985671e-15 C 147 4 0 0 0 2 2 0 0 0 0 0

FABP4 0.966310097806305 0.00902287834351084 0.0219788955479575 C 73 5 1 0 2 0 0 1 2 0 0 0

SLC7A5 0.965198120326481 2.99448276887442e-09 5.69613822778756e-08 MN 4 1 0 0 0 0 0 0 1 0 0 0

DSG2 0.961791405504814 7.59242413012848e-06 5.51426751768771e-05 MN 4 1 0 0 0 0 0 1 0 0 0 0

NUDCD1 0.961453970678919 2.19899062291915e-12 8.87426343709825e-11 C 2 2 1 0 0 0 0 2 2 0 0 0

GCNT1 0.958220171423258 5.03289907380555e-07 5.23969641198667e-06 MN 1 1 0 0 0 0 0 1 0 0 0 0

CYR61 0.957953519332059 9.42205144767941e-10 2.018073405239e-08 MN 6 3 0 2 0 0 0 2 0 0 0 0

CKAP2L 0.956362487296015 6.20709530932526e-10 1.38867249313947e-08 MN 1

APOBEC3A 0.955564520338708 0.00175773351159615 0.00558479472452247 C 12 2 0 1 0 0 0 2 0 0 0 0

MMP14 0.954012441758189 4.94760782448821e-13 2.30648702694799e-11 MN 29 3 0 1 0 0 0 2 0 0 0 0

CCL8 0.944655570833839 7.44194097897162e-06 5.41744680419215e-05 C 24 2 1 0 0 0 1 0 0 0 0 0

CDC20 0.942642573385793 2.39329198931164e-12 9.50967658062938e-11 C 49 6 2 1 0 0 3 0 2 0 0 0

DCBLD2 0.940361573825191 1.37005429369864e-06 1.25149698922945e-05 C 6 2 1 0 0 0 0 2 0 0 0 0

WDR54 0.938853456646245 1.35357808075952e-10 3.52385056573478e-09 NA

TRIM22 0.938385882944281 1.43213425986102e-10 3.68789185529109e-09 C 3 2 0 0 0 0 0 1 1 0 0 0

APOBEC3B 0.938325765053223 1.63534905654205e-07 1.96024392610331e-06 C 130 5 2 0 0 0 1 1 1 0 0 0

FJX1 0.936898355710949 2.00517149882715e-14 1.31777364438547e-12 C 1 0 0 0 0 0 0 0 0 0 0 0

FXYD5 0.935847834205966 1.25127200367118e-10 3.2847191696927e-09 MN 1 1 0 1 0 0 0 1 0 0 0 0

TRANK1 0.934861433912253 9.86777692097497e-12 3.38346764100169e-10 NA

APOL6 0.933770589709386 2.12260032070079e-07 2.47684687237385e-06 C 1 0 0 0 0 0 0 0 0 0 0 0

C1orf74 0.933056646299439 1.39646266536279e-07 1.71184254322031e-06 NA

PRKDC 0.932855537543936 2.44237892819371e-14 1.57771010149906e-12 C 148 11 4 1 0 1 6 2 2 0 0 1

CCNB1 0.932707980049435 9.15529496941725e-09 1.53482965355324e-07 MN 27 2 0 1 0 0 0 1 1 0 0 0

EIF2S1 0.931301659463255 6.39103954603378e-10 1.42309653515037e-08 C 91 5 0 0 0 0 1 0 5 0 0 0

PTPN12 0.931164585901959 7.5951870263456e-13 3.39844219497974e-11 C 21 6 0 0 0 0 0 6 0 0 0 0

STAT1 0.929670493032114 4.53507012943514e-09 8.24546324109691e-08 MN 5 1 0 0 1 0 0 0 0 0 0 0

DHRS2 0.928120421101982 0.000624615205847852 0.00236773322673389 C 24 0 0 0 0 0 0 0 0 0 0 0

LHFPL2 0.924302303663977 7.32881936083969e-16 6.86695861597094e-14 C 1

MTERFD1 0.924087527222878 9.76356308622985e-13 4.22871758656916e-11 NA

CENPW 0.921285343975538 2.94306443964431e-05 0.00017779566118464 C 4 0 0 0 0 0 0 0 0 0 0 0

OAS1 0.919554961643901 5.40104869419739e-07 5.56070482766779e-06 C 7 1 0 0 0 0 1 0 0 0 0 1

MMP11 0.918337148650495 0.000274753348267475 0.00118618992305166 MN 5 1 0 0 0 0 1 0 0 0 0 0

MSN 0.918331590261906 6.28728283076823e-20 1.41665954926131e-17 MN 3 1 1 1 0 0 0 1 0 0 0 0

CCNA2 0.91770525853966 4.35693802162465e-11 1.24756075545068e-09 C 1138 17 9 1 1 1 5 4 4 0 0 0

FBXO6 0.915611252470583 3.47885477064007e-13 1.69265512195128e-11 C 2 0 0 0 0 0 0 0 0 0 0 0

SOX9 0.91439135722362 3.38911846959757e-05 0.000199955876789505 C 95 21 3 7 1 0 5 4 8 0 0 0

UBE2C 0.913619141250937 4.51534965769699e-08 6.30716036688051e-07 C 57 23 11 9 4 1 0 2 8 0 0 0

HMMR 0.912594727001647 3.53283699899205e-07 3.86508704508223e-06 C 97 22 8 5 1 0 2 11 4 0 0 0

COL4A6 0.912027429422427 8.12226533090203e-07 7.9570453373697e-06 C 4 0 0 0 0 0 0 0 0 0 0 0

HLA-F 0.908951361524214 1.90571265181864e-14 1.26116864898501e-12 C 33 1 0 1 0 0 0 0 0 0 0 0

GREM1 0.908621080440287 3.57989102372321e-05 0.000209642937518593 C 42 9 1 1 1 2 1 1 4 0 0 0

DSCAM 0.908153018052512 3.02531271387853e-05 0.000181976501349029 C 8

CASP7 0.907782411815025 1.8361712810996e-15 1.60153059158397e-13 C 341 45 1 2 1 0 36 1 6 0 0 0

PFDN2 0.905237075867002 9.73506512354698e-22 3.07092629322289e-19 NA

C6orf141 0.904754985198047 6.29038873575467e-11 1.75085570002395e-09 C 1

ZNF114 0.904425615566047 5.33172795380807e-05 0.000294810444001535 NA

MCM4 0.900788877832151 1.16156603387528e-10 3.07481962561641e-09 C 17 3 1 0 2 0 0 1 0 0 0 0

LYPD1 0.899585327597495 1.01588642204469e-05 7.10820048430312e-05 C 3 0 0 0 0 0 0 0 0 0 0 0

KHDC1 0.897195049483785 8.60615319473624e-13 3.77057086844382e-11 NA

HAPLN3 0.895763527526771 4.84662984533239e-11 1.37735980604514e-09 C 1 0 0 0 0 0 0 0 0 0 0 0

STARD3NL 0.895450985071613 7.61905283257931e-13 3.40108049439218e-11 NA

IL1B 0.894040334641292 3.37655611857974e-05 0.000199401178334973 MN 15 2 0 0 0 0 0 0 2 0 0 0

NRAS 0.892898912672197 4.19736120545069e-07 4.48326498507705e-06 MN 14 1 0 0 0 0 0 1 1 0 0 0

ODC1 0.89242862591055 3.42028357257563e-05 0.000201606064086387 MN 15 3 0 0 0 0 0 0 3 0 0 0

THBS1 0.890024678773595 3.0651710464303e-08 4.43622654752353e-07 MN 9 2 0 1 0 0 0 1 0 0 0 0

PTK2 0.889798631438208 1.31392023362564e-17 1.95815498124666e-15 MN 33 1 0 0 0 0 0 0 1 0 0 0

CXCL3 0.88968392199848 9.97989466573091e-07 9.52063842430892e-06 C 25 3 1 0 0 1 0 1 0 0 0 0

LUZP1 0.889605633613938 3.78693611265936e-14 2.33470162229002e-12 C 1

ITGA2 0.886476013999129 2.25663139127118e-09 4.40776701161916e-08 C 147 12 0 2 1 0 1 11 1 0 0 0

ALDH1B1 0.885191552948934 2.33895730661741e-07 2.69278862179731e-06 C 5

AFAP1-AS1 0.884583929639328 0.0174760438873096 0.0381114278897464 C 1 0 0 0 0 0 0 0 0 0 0 0

APP 0.884251277986479 4.86614323286277e-12 1.78491265442623e-10 C 757 32 6 0 0 2 24 0 0 0 0 0

SLC6A15 0.883505924409985 0.000300040565278645 0.00127672387118456 C 3

TMEM132A 0.880451865676946 3.78743979161008e-13 1.81941301867523e-11 C 26 0 0 0 0 0 0 0 0 0 0 0

SHCBP1 0.87893639171004 1.74676804267594e-09 3.50083374805331e-08 NA

PDP1 0.878435714772711 8.85345288561911e-10 1.91832629034619e-08 NA

CDK1 0.878362767933483 7.7953495309006e-08 1.02674029625161e-06 MN 28 3 0 1 0 0 1 0 1 0 0 0

EXT1 0.87669621039824 5.68662552784149e-12 2.0540221634629e-10 C 85 6 0 1 0 0 1 1 3 0 0 0

FCER1G 0.874137884651291 5.2467437517568e-06 4.01882310762853e-05 C 113 0 0 0 0 0 0 0 0 0 0 0

ADAM10 0.874038297653912 2.40251220997811e-08 3.61178304990117e-07 MN 2 2 0 0 0 0 0 1 1 0 0 0

RRAS 0.872351573423583 1.24749044013225e-11 4.14232483515492e-10 C 145 8 3 0 0 0 5 2 0 0 0 0

AJAP1 0.872049726463854 0.00813377088684762 0.0201945268977936 C 7 2 0 1 0 0 0 1 1 0 0 0

VMP1 0.871610099134438 1.64793300402739e-07 1.97407771944471e-06 C 8 4 2 1 0 0 0 3 1 0 0 0

IFIT5 0.868934836066268 3.78740855914836e-11 1.10794871404947e-09 C 18 6 1 0 1 0 3 1 1 0 0 0

TGFA 0.868461094116915 1.07203084274465e-07 1.3526885173752e-06 MN 29 1 0 0 0 0 0 0 1 0 0 0

GBP4 0.868406829925219 6.26920944533464e-06 4.68815990406356e-05 C 1 0 0 0 0 0 0 0 0 0 0 0

FADS3 0.867556971150541 5.46747859423178e-06 4.17270029649294e-05 C 4 0 0 0 0 0 0 0 0 0 0 0

MTFR1 0.864560152660739 2.80762466864669e-14 1.78321852696228e-12 NA

ZYX 0.863889481233912 6.9618901785721e-13 3.15233721076158e-11 MN 2 2 0 0 0 0 0 1 2 0 0 0

ATP13A3 0.863683799463561 2.59830687069855e-13 1.29757662643038e-11 C 1 0 0 0 0 0 0 0 0 0 0 0

IL1A 0.863619410757365 0.00621505851115192 0.0160941869531499 MN 4 1 0 0 0 0 0 0 1 0 0 0

CDKN2A 0.863513805939649 0.0013449730573457 0.00445663603928258 MN 117 17 3 5 2 0 0 8 2 0 0 0

UBASH3B 0.862403817333077 2.86296937407375e-12 1.1195748211383e-10 C 314 2 0 0 0 0 0 0 1 0 0 1

F2RL1 0.862138965045483 1.39423980190554e-08 2.20457616797545e-07 C 102 12 1 0 0 1 1 4 7 0 0 1

DGKH 0.860395987938133 8.77440958643531e-11 2.37927292610976e-09 MN 1

RNF19B 0.860218889730907 3.43271507157896e-10 8.11123572531523e-09 C 2 0 0 0 0 0 0 0 0 0 0 0

CLSPN 0.859773754909184 5.6141348305667e-09 9.98672273854661e-08 C 9 2 0 0 1 0 0 0 2 0 0 0

CHEK1 0.859475264485983 6.7325270973575e-09 1.16905082909803e-07 C 391 53 16 3 1 0 32 1 8 0 0 0

LPAR3 0.858919037094656 2.19957160501788e-07 2.55406697964254e-06 C 232 6 2 0 0 1 1 2 1 0 0 0

MOCOS 0.856582272737875 3.12897502349816e-07 3.47343755247799e-06 NA

ITGB1 0.856260419438573 1.66494290997816e-10 4.22984892042371e-09 C 206 7 1 0 1 1 2 5 0 0 0 0

PROCR 0.854779922910958 4.86261964377192e-08 6.70807594735213e-07 C 26 5 1 1 0 0 1 2 1 0 0 0

SOCS3 0.854058008351021 1.00575182258659e-07 1.28015230303271e-06 MN 3 2 1 0 0 0 0 0 1 0 0 0

LOC100506328 0.853670705585107 0.000452432101315471 0.00180657856151855 NA

MYO5A 0.852589199052458 1.57313335979901e-11 5.1159269932845e-10 C 14 0 0 0 0 0 0 0 0 0 0 0

SLC7A8 0.850348795338228 0.000170335800098887 0.000795054423790786 C 9 0 0 0 0 0 0 0 0 0 0 0

SLC3A2 0.848267428581149 1.3338283000216e-14 9.45519409532168e-13 C 44 10 2 2 0 2 1 4 3 0 0 0

XPR1 0.847496414924512 1.91081533759131e-17 2.71464026117901e-15 NA

LRRC8C 0.846946471764565 1.45642820404743e-10 3.74535551875077e-09 NA

NEXN 0.846263154368973 3.87643256834074e-07 4.18296688831158e-06 NA

LAMA1 0.845723625122896 7.22443858393038e-06 5.28554113173754e-05 C 49 0 0 0 0 0 0 0 0 0 0 0

CEBPB 0.845214499959651 3.25493684431987e-12 1.24708885936118e-10 C 271 8 2 0 0 0 2 3 1 0 0 0

DYRK3 0.843662690279372 1.77404548441321e-11 5.72016335323489e-10 NA

CALB1 0.840680124719123 0.0031306858786098 0.00901894849687179 C 44 3 0 0 1 0 2 0 0 0 0 0

FKBP14 0.839344756040007 1.58688455378341e-08 2.46998058794889e-07 C 4 0 0 0 0 0 0 0 0 0 0 0

HJURP 0.838186242808567 4.24304233313488e-09 7.78178897667091e-08 C 5 2 2 1 0 0 0 0 1 0 0 0

PLXNA1 0.837174274826367 2.55423642692303e-11 7.96442056875982e-10 C 135 1 0 0 0 0 1 0 0 0 0 0

LAMP3 0.836657447107184 4.61719272532892e-06 3.60666969510113e-05 C 200 6 0 3 0 0 1 3 0 0 0 0

XRCC2 0.834317158845181 3.86541691084979e-09 7.18511136058095e-08 C 10 1 0 0 1 0 0 0 0 0 0 0

CD86 0.832941324136789 2.72923043827395e-05 0.000166823491806203 C 624 30 10 0 0 1 4 2 16 0 0 0

POSTN 0.832500996867182 0.00384505890538238 0.0107196096482799 MN 1 1 0 0 0 1 0 1 0 0 0 0

GNLY 0.830863604126041 5.76466030858466e-05 0.000316071047684188 C 22 4 2 1 1 0 2 0 1 0 0 0

NEDD1 0.830793199485689 2.57728588822539e-12 1.01414324337717e-10 C 1 1 0 0 0 0 0 1 0 0 0 0

C16orf74 0.830075554983001 1.11458076851745e-11 3.77382293483537e-10 C 1

PHEX 0.829639422099854 2.03592393121838e-05 0.000130667793306783 NA

CHST2 0.828362591644304 3.60677453436091e-05 0.00021102139601808 C 4 0 0 0 0 0 0 0 0 0 0 0

TYMP 0.827922689707452 1.45486030634964e-11 4.77229480386129e-10 C 676 13 2 2 0 5 0 4 3 0 0 0

SLC22A1 0.827020004136061 4.52280934865376e-06 3.54464648206914e-05 C 21 3 2 1 0 0 0 0 1 0 0 0

PRNP 0.82649125320383 3.27361404018827e-11 9.7561813444057e-10 NA

GFPT2 0.825510744414723 7.61038840491371e-07 7.51391869273875e-06 C 1 0 0 0 0 0 0 0 0 0 0 0

SCRN1 0.824665380491358 6.23932960742304e-12 2.22395087532384e-10 C 6 1 0 1 0 0 0 1 0 0 0 0

COL4A2 0.824418521666058 6.03577987403955e-11 1.69243267668069e-09 C 104 5 2 0 0 3 2 1 1 0 0 0

ADA 0.823062015494876 2.57539702663375e-10 6.28151282514135e-09 MN 2 1 0 0 1 0 0 0 0 0 0 0

AFAP1L1 0.822234739086932 2.7085089714249e-16 2.77102428660319e-14 C 2 1 0 1 0 0 0 0 0 0 0 0

CDC6 0.821349385517537 5.70648755740712e-10 1.28732646006013e-08 MN 2 2 0 1 1 0 0 1 1 0 0 0

ZNFX1 0.820453708812903 3.18118636156171e-10 7.53570891930896e-09 C 1 0 0 0 0 0 0 0 0 0 0 0

POP1 0.817246988058485 3.47456621965926e-13 1.69265512195128e-11 C 2 0 0 0 0 0 0 0 0 0 0 0

DCUN1D5 0.817200939451802 1.11126606398871e-08 1.80385358431513e-07 C 1 0 0 0 0 0 0 0 0 0 0 0

F2RL2 0.816930672238459 0.000292420542364949 0.00124935521565269 C 29 5 0 0 0 0 1 4 1 0 0 0

UBE2V2 0.816780579219389 4.06412137186654e-11 1.1743759573331e-09 C 2 0 0 0 0 0 0 0 0 0 0 0

SLC25A32 0.816464293058747 8.80848733704426e-15 6.66872959312947e-13 C 3 0 0 0 0 0 0 0 0 0 0 0

LOC375295 0.815930690424972 4.01824926414837e-05 0.000231185952831942 NA

AGRN 0.815035641121705 6.23173711750354e-12 2.22395087532384e-10 C 15 3 0 0 0 2 0 1 1 0 0 0

BMP1 0.813014411723666 4.12649471524583e-11 1.18876964194e-09 C 115 0 0 0 0 0 0 0 0 0 0 0

DEPDC1 0.811405815613613 3.16053525001863e-10 7.49617176404795e-09 C 5 1 1 1 1 0 0 0 0 0 0 0

DOCK7 0.809767932517714 4.02857367112895e-11 1.16766943144652e-09 C 1

DNAH17 0.809173170340404 3.56821338635798e-07 3.90153522608882e-06 C 1

RDH11 0.808345758563635 2.27433802013124e-05 0.000143440172299314 C 2 0 0 0 0 0 0 0 0 0 0 0

FSD1L 0.808178085793092 4.94790354344826e-09 8.89353944604419e-08 C 1 0 0 0 0 0 0 0 0 0 0 0

NFIL3 0.807482685527305 1.05038346883536e-08 1.727246560786e-07 C 7 4 1 0 0 0 3 0 0 0 0 0

RARRES3 0.807195894612875 1.80757322100024e-05 0.000118339461618372 C 20 3 0 0 0 0 2 0 1 0 0 0

FZD6 0.806240159124438 1.03274346647937e-07 1.30834910241332e-06 C 10 0 0 0 0 0 0 0 0 0 0 0

GPR153 0.805072532467691 1.29727838744433e-06 1.19076566630257e-05 C 1

COL5A2 0.804862032788063 7.5283924444653e-05 0.000399355055483169 C 5 0 0 0 0 0 0 0 0 0 0 0

CXCL5 0.80465799900345 0.0011395954061065 0.00389193833478486 C 100 18 6 3 3 2 0 7 5 0 0 1

ADAM19 0.80433856597664 2.35578024836354e-08 3.54434441659593e-07 C 7 2 0 0 0 0 0 2 0 0 0 0

EMR2 0.804146829193842 1.66142434781861e-08 2.5817552242334e-07 C 3 0 0 0 0 0 0 0 0 0 0 0

BNC1 0.803160062541992 2.73192475704378e-08 4.02703581593206e-07 C 17 0 0 0 0 0 0 0 0 0 0 0

PAK1 0.801891095416531 2.61337320889487e-11 8.12205496301368e-10 C 148 32 8 3 0 1 3 20 9 0 0 0

KIRREL 0.801614070783667 7.81182938876282e-11 2.14592880756334e-09 NA

NBN 0.799738631348545 4.75499748158392e-10 1.08966023244347e-08 C 247 10 2 1 0 0 3 4 1 0 0 0

KIF23 0.798477028405475 3.13297336146023e-14 1.97002613994544e-12 C 9 1 1 0 0 0 0 0 1 0 0 0

VNN3 0.798155803846283 0.000350772701988184 0.00145657633403474 C 2

FCGR2A 0.796129508417542 1.29629831967373e-07 1.60150380525226e-06 C 53 1 1 0 1 0 0 0 0 0 0 0

PICALM 0.795145571904313 1.82344962029737e-07 2.16378877513281e-06 C 178 3 1 1 0 0 0 1 1 0 0 0

TMEM92 0.794861971265349 3.28214019626125e-07 3.62010882835878e-06 NA

MYBL2 0.793274272636805 1.08957437050095e-08 1.7808613221478e-07 C 64 12 1 2 0 0 4 1 4 0 0 0

SLC6A2 0.793205860120951 0.000429693250071903 0.00173082968217921 MN 13 3 0 1 0 0 0 1 2 0 0 0

MYBPH 0.792776560604036 0.0118768274850971 0.0277282242272644 C 23 1 0 1 0 0 0 1 0 0 0 0

ACSL5 0.792317575381528 1.70400826267742e-05 0.000112532325148972 C 11 2 1 0 0 0 0 0 1 0 0 0

PLOD2 0.790921115837989 2.31225787837047e-07 2.66843186972336e-06 C 13 1 0 0 0 0 0 1 0 0 0 0

ANXA5 0.79038940142073 6.06877731027853e-13 2.76780116028052e-11 MN 57 4 0 0 0 0 3 1 0 0 0 0

TINAGL1 0.790057123180834 2.12817641989695e-07 2.48029526474073e-06 C 37 4 2 0 0 0 0 1 1 0 0 0

TMCC3 0.789304784293048 8.75223183876341e-08 1.13694915588384e-06 NA

HAVCR2 0.789200032328533 6.40983804901993e-07 6.44210869106631e-06 C 45 9 2 4 0 0 0 1 2 0 0 0

CLEC2D 0.78839039588447 6.74630192128141e-06 4.98973256991377e-05 C 1 0 0 0 0 0 0 0 0 0 0 0

JHDM1D 0.788294075789964 2.06411697354997e-10 5.11454171614887e-09 C 2 1 0 0 0 1 0 0 0 0 0 0

CTSL2 0.788104647799758 5.77114623894418e-05 0.000316335027119885 C 6 0 0 0 0 0 0 0 0 0 0 0

LAIR2 0.787971723801666 0.000298572536992028 0.00127219324800723 C 1 0 0 0 0 0 0 0 0 0 0 0

FERMT1 0.78792154571321 2.3996092807298e-06 2.04123167893811e-05 C 10 0 0 0 0 0 0 0 0 0 0 0

RHOB 0.787479030896153 2.32953839205585e-07 2.68521151927169e-06 C 107 32 9 0 0 1 10 9 9 0 0 0

DEPDC1B 0.786836233904609 5.13126300107981e-10 1.16677828510252e-08 C 1 0 0 0 0 0 0 0 0 0 0 0

GINS1 0.785911394811691 2.2655743436279e-07 2.62426717269555e-06 C 6 2 1 0 0 0 0 0 2 0 0 0

SLC38A1 0.785376756240543 1.47330323110663e-14 1.02519155349835e-12 C 22 1 0 0 1 0 0 0 0 0 0 0

MINPP1 0.785196797161284 4.62823081759838e-07 4.8665847047047e-06 C 2 0 0 0 0 0 0 0 0 0 0 0

SPOCD1 0.784571457002861 2.03762475231417e-05 0.000130732622667967 NA

LOXL2 0.784452807168327 1.76512263611942e-08 2.72722254153732e-07 C 34 6 2 1 0 0 0 5 0 0 0 0

CENPF 0.783653374388254 1.00208156098409e-08 1.66052134997681e-07 C 25 3 0 3 0 0 0 0 2 0 0 0

MAP7D3 0.782697780264269 4.49892761099413e-13 2.14500555254204e-11 NA

ZHX2 0.781824422447755 6.10716505624806e-11 1.7073901480001e-09 C 2245 2 1 0 0 0 1 0 0 0 0 0

HUS1B 0.781672006071812 1.12260909757094e-11 3.79421828387951e-10 C 1

CNN2 0.781225149839194 3.14233413208261e-08 4.53661007764512e-07 C 4 2 0 0 0 0 1 1 0 0 0 0

ASAP1 0.781017308016582 5.72323130745821e-15 4.45776127392023e-13 C 825 7 4 0 1 0 0 5 0 0 0 0

KIAA1324L 0.778620577048938 4.9568751074488e-06 3.82933776157891e-05 NA

PLEC 0.778430494851835 2.93567658439031e-09 5.58989443790295e-08 C 42 5 0 1 1 0 2 2 0 0 0 0

PSME4 0.777664437289886 4.23798997653408e-15 3.44259383201119e-13 C 2 0 0 0 0 0 0 0 0 0 0 0

KIF4A 0.777444526827114 2.73824657100543e-11 8.44084574094785e-10 C 5 2 0 1 0 0 0 0 1 0 0 0

FNDC3B 0.776934251324104 1.52976105435439e-12 6.41990853121185e-11 C 4 2 0 0 0 0 0 2 0 0 0 0

ABCA1 0.776604490999422 8.69672479506803e-10 1.88981527205801e-08 C 1415 58 10 0 4 1 9 18 17 0 0 1

FAM111B 0.776407189960637 3.22737714857548e-06 2.65123989978681e-05 C 5 0 0 0 0 0 0 0 0 0 0 0

MTHFD1L 0.775731776354529 2.09834392836031e-12 8.55934386467148e-11 C 1 0 0 0 0 0 0 0 0 0 0 0

BLM 0.774873821981915 1.01872503469076e-09 2.15917231036865e-08 C 208 6 3 0 0 0 2 0 3 0 0 0

AURKB 0.773968132499657 1.09850568701794e-09 2.30503515944441e-08 MN 3 2 1 1 1 0 0 1 1 0 0 0

CDKN3 0.773148372368613 2.45420319371392e-07 2.80668905422498e-06 C 34 3 0 0 0 0 0 0 3 0 0 0

C1QA 0.773099611114677 4.5416184179913e-05 0.000256671280374205 C 5 1 0 0 0 0 0 1 0 0 0 0

TIMP1 0.773004451042332 6.19192632736569e-07 6.26374075884824e-06 MN 51 1 0 0 0 0 0 1 0 0 0 0

EPHB2 0.772853260883053 2.74027340116132e-06 2.28884177686585e-05 MN 73 2 1 0 0 0 1 1 1 0 0 0

APOL2 0.772049160818161 1.14147392941574e-08 1.84183095158156e-07 C 2 1 0 0 0 0 1 0 0 0 0 0

ZWINT 0.772005559067686 3.37297001455457e-08 4.83637905041472e-07 C 5 1 0 0 0 0 0 0 1 0 0 0

KIF2C 0.771735168136465 1.80187127620676e-11 5.80000300081042e-10 C 20 6 4 2 0 1 0 0 1 0 0 0

GUCY1A2 0.771532568559895 1.12106138922581e-07 1.40798466581798e-06 NA

TNFSF10 0.771297239631918 5.21275205205085e-05 0.000289076349514112 MN 19 6 1 0 0 0 5 1 0 0 0 0

MCM10 0.771122164929394 9.18910255330302e-07 8.87810842401053e-06 C 4 0 0 0 0 0 0 0 0 0 0 0

BUB1 0.770956767969777 1.28704191317992e-09 2.65069012957087e-08 MN 1 1 0 0 0 0 0 0 1 0 0 0

PDIA4 0.770856855056611 1.99854163758092e-12 8.20529231550846e-11 C 3 1 0 0 0 0 0 1 0 0 0 0

NEIL3 0.76948161803532 3.6702700145486e-07 3.99696205784588e-06 C 4

CD14 0.769298952765735 1.47567865034174e-07 1.79962434375117e-06 C 610 31 6 4 7 1 5 2 12 0 0 1

KRT17 0.768894294165711 1.44205367199514e-06 1.30654618716381e-05 C 97 3 0 1 0 1 0 0 1 0 0 0

ITGA6 0.768154607065397 1.04843845156349e-10 2.7949006440482e-09 C 170 12 0 2 0 1 0 10 1 0 0 0

GABRP 0.767640532604446 0.00179148295156334 0.00567867992367096 C 3 1 0 1 0 0 0 0 0 0 0 0

MRPL15 0.766733941181674 1.74265805052071e-14 1.1737825239219e-12 NA

CAP1 0.766417563769389 5.67494460248932e-07 5.81535877050977e-06 NA

SPATA5 0.766400336860382 1.00125143880594e-13 5.49295245863192e-12 C 4

HELLS 0.765698088199596 2.68079495255889e-08 3.96711540790321e-07 C 22 3 0 0 1 0 1 0 1 0 0 0

FANCI 0.765253591925564 2.86828761322996e-09 5.48364440965691e-08 C 1 1 0 0 0 0 0 1 0 0 0 0

C3AR1 0.763620983165486 2.71949440674899e-06 2.27349251928172e-05 C 2 0 0 0 0 0 0 0 0 0 0 0

PXN 0.762142038748761 1.34021461945852e-14 9.46501570988484e-13 MN 10 1 1 0 0 0 0 1 0 0 0 0

NSMCE2 0.762036452329637 1.76156022174376e-11 5.68959903019526e-10 C 2 0 0 0 0 0 0 0 0 0 0 0

BIRC2 0.761859002809893 3.23792485110957e-05 0.000192535983842133 MN 7 2 0 0 0 0 2 0 0 0 0 0

PTGS1 0.760900683591678 0.000880770921721605 0.0031491388644394 C 150 2 0 0 0 0 0 0 1 0 0 2

IL6 0.76042942955891 0.000450430564865961 0.00180010542677746 MN 124 8 1 1 0 0 1 4 0 0 0 1

IGF2BP3 0.760422082184202 0.00487619874175651 0.0131376247096406 C 103 17 3 7 5 1 0 5 2 0 0 0

DHX33 0.758069787717203 1.16447740700515e-07 1.45671274834015e-06 NA

APOL3 0.757940180665747 6.48636757301276e-07 6.5094103422276e-06 MN 1

BRCA2 0.757917314639378 4.5486975833845e-08 6.33970538738722e-07 C 463 43 5 6 1 0 7 13 16 0 0 0

TPBG 0.757794097661719 3.59918545316049e-07 3.92859187266255e-06 C 2 0 0 0 0 0 0 0 0 0 0 0

SHC1 0.757677578380656 2.17966197320418e-09 4.27064825743638e-08 C 307 23 1 2 0 0 4 8 10 0 0 0

COL7A1 0.757330942344086 7.37748521298867e-08 9.75776817793407e-07 C 10 2 0 0 0 0 0 2 1 0 0 0

TMEM171 0.755098423860785 2.80604387050885e-07 3.1726399245592e-06 NA

AP2B1 0.754952849043923 1.38708947352597e-05 9.35175025971691e-05 C 2 0 0 0 0 0 0 0 0 0 0 0

KDM1A 0.752970513366542 5.55179431532916e-12 2.00915508616128e-10 C 48 7 3 1 1 0 2 0 3 0 0 0

MYO3B 0.751956979809879 0.000273522402589769 0.00118249395016367 C 1 0 0 0 0 0 0 0 0 0 0 0

CD44 0.750820140733018 8.71842829389246e-10 1.89235885686356e-08 MN 67 11 0 2 0 0 0 8 1 0 0 0

FOLR3 0.750287942807065 0.00302367313974128 0.00876267976050884 C 1 0 0 0 0 0 0 0 0 0 0 0

P2RY6 0.749435450763181 2.85119605238284e-06 2.37102757835897e-05 C 15 2 0 0 0 0 0 0 2 0 0 0

IGFL2 0.749367464399282 0.0214155415636575 0.0450719398615975 C 1 0 0 0 0 0 0 0 0 0 0 0

CALB2 0.749164054639433 0.0180226714190571 0.0391058574758583 C 264 41 1 1 26 0 1 17 2 0 0 0

MT1L 0.748648522694416 3.39639432714955e-06 2.76964909219989e-05 MN 29 2 0 0 0 0 0 2 0 0 0 0

BRMS1 0.748619625766698 1.96932544511185e-13 1.00738980269275e-11 C 85 26 3 0 0 1 1 24 1 0 0 0

EFCAB4B 0.748409506750924 9.59387258875343e-07 9.23148075685491e-06 NA

DDX3Y 0.747349312524305 0.015295681834892 0.0341796186645809 C 4 0 0 0 0 0 0 0 0 0 0 0

ABL2 0.747084940821999 1.81316656616969e-10 4.55143283791693e-09 MN 10 1 0 1 0 0 0 0 0 0 0 0

CKS2 0.74675860307953 3.19201279208532e-07 3.53512148132234e-06 C 29 5 1 1 0 0 2 3 1 0 0 0

TASP1 0.746700642741104 1.54597874624533e-05 0.000103067064917877 C 1 0 0 0 0 0 0 0 0 0 0 0

IFNG 0.746640650642046 0.000981668730180984 0.00344329949150028 MN 49 2 1 0 0 0 0 0 1 0 0 0

ITPR3 0.746068869213778 3.99644900503361e-10 9.34991227667133e-09 C 26 4 0 0 0 0 1 0 3 0 0 0

TM4SF19 0.744922077522583 2.30088409918951e-05 0.000144440574943151 NA

SMURF2 0.743768993384757 1.272466671923e-07 1.57514563109788e-06 C 21 6 1 1 0 0 0 2 2 0 0 0

VCL 0.742251925543914 1.43377628464928e-11 4.71949282427078e-10 MN 4 1 0 0 0 0 0 1 0 0 0 0

STK3 0.742189936194717 1.32811395562658e-13 7.06101484217538e-12 C 22 5 0 0 0 0 4 0 1 0 0 0

PLEKHB2 0.741848817647747 3.65486077877988e-06 2.95622008375927e-05 NA

TNFSF18 0.741389344454302 0.00235480994111541 0.00712883681309843 C 14 1 1 0 0 0 0 1 0 0 0 0

ELF4 0.741063423110933 3.66683167497234e-08 5.22606348736456e-07 C 146 1 0 0 0 0 1 0 0 0 0 0

SH2D5 0.740584370661432 2.49110263834651e-05 0.000154384740130925 NA

IL11 0.7403163423563 3.50092127956322e-05 0.000205718525483679 MN 2 1 0 0 0 0 0 1 0 0 0 0

ST3GAL5 0.739527052730975 7.52283953334442e-10 1.65949631524021e-08 C 16 3 1 0 0 0 0 3 0 0 0 0

TAP2 0.739258780171614 2.20341009558031e-09 4.31271384478269e-08 C 41 2 0 1 0 0 0 0 1 0 0 0

GLI3 0.739106653076486 2.19265075824662e-07 2.54759367104566e-06 C 95 3 0 0 0 0 0 0 3 0 0 0

GSDMB 0.738705470196786 1.58507210563009e-06 1.41791340819545e-05 C 3 0 0 0 0 0 0 0 0 0 0 0

SERPINH1 0.737797427657009 3.48190458439365e-08 4.9812553339999e-07 MN 4 1 0 0 0 0 0 1 0 0 0 0

TFRC 0.737068260551572 8.99104236662242e-07 8.70452475054028e-06 MN 4 2 1 1 1 0 0 0 2 0 0 0

GLS 0.736444058561463 9.56188591565809e-08 1.22946885003846e-06 C 133 12 6 0 0 0 3 2 2 0 0 0

TLN1 0.736374715659133 1.19569821234444e-11 3.99840637191578e-10 MN 2 1 0 0 0 0 0 0 1 0 0 0

SPHK1 0.732879882896349 4.57082995723397e-07 4.81693199335008e-06 C 162 46 19 2 2 2 8 13 13 0 0 1

TTC7B 0.73282674952909 9.67178120253186e-07 9.2828500415984e-06 NA

PTAFR 0.732433332866269 2.00983285078349e-06 1.75300029339995e-05 C 30 5 2 0 0 0 2 2 0 0 0 0

SLC2A9 0.732286072735897 1.27756168472357e-10 3.34445504934482e-09 C 2 0 0 0 0 0 0 0 0 0 0 0

MRPS23 0.73216405921408 1.57522127920875e-07 1.90263006710811e-06 C 1 0 0 0 0 0 0 0 0 0 0 0

NT5DC3 0.731096969381713 1.06448988327231e-07 1.34586506484269e-06 C 2

NUP37 0.730858257379724 1.19643367104108e-10 3.15828453163103e-09 C 12 5 2 0 0 0 1 2 1 0 0 0

GBP3 0.729879567348474 1.37551114129224e-06 1.25466502993919e-05 C 1

BTN3A2 0.729871594668061 1.11071981111823e-07 1.39777884740922e-06 C 2

FHOD1 0.729146571043238 1.42513169530381e-13 7.53448815559083e-12 C 1 0 0 0 0 0 0 0 0 0 0 0

SNX7 0.729077031057956 4.23426716793092e-07 4.51758594630375e-06 C 1

PSMD13 0.728528842746694 3.7950964012504e-15 3.0972474902187e-13 C 29 1 0 0 0 0 0 1 0 0 0 0

C12orf45 0.728109875366526 3.4172545988778e-11 1.00959897948965e-09 NA

WTAP 0.728046645968818 2.1780776197886e-08 3.31120282005935e-07 C 1 0 0 0 0 0 0 0 0 0 0 0

CKAP5 0.727405037587054 9.11108769922212e-12 3.15834353265892e-10 C 5 0 0 0 0 0 0 0 0 0 0 0

FAM3C 0.726974323967335 0.000190748612597 0.000874797054678393 C 5 1 0 0 0 0 0 1 0 0 0 0

C3orf52 0.726122632489151 2.83528521105069e-11 8.66937692884596e-10 C 2 1 0 0 0 0 0 0 0 1 0 0

TGFB1 0.725797916737982 3.59426234113754e-10 8.46126907098387e-09 MN 32 1 0 0 0 0 1 0 0 0 0 0

APLN 0.725361009245774 4.68617531660866e-06 3.64890341794559e-05 MN 1 1 1 1 0 0 0 0 1 0 0 0

LPP 0.72534116594014 4.83388401708615e-09 8.72172762548994e-08 C 36 2 0 0 0 0 0 1 1 0 0 0

BIRC5 0.725300564937009 1.82428676008299e-08 2.81404038370747e-07 MN 4 1 0 0 0 0 1 1 0 0 0 0

PPEF1 0.724963086234649 5.49298045421998e-05 0.000302401515581796 C 2 0 0 0 0 0 0 0 0 0 0 0

NRP2 0.724762082830013 2.21762760315263e-08 3.35515888448199e-07 MN 1 1 1 0 0 1 0 0 0 0 0 0

FLJ32255 0.723590583510803 0.00127917717642732 0.00427152195099502 NA

GGH 0.723392641151354 0.000115538794149256 0.000573362023299151 C 14 1 0 0 1 0 0 0 0 0 0 0

STX1A 0.7231033514325 3.99439010001835e-09 7.39743849540581e-08 C 14 1 1 0 0 1 0 0 1 0 0 0

NEK6 0.723092448158567 1.74791984567569e-09 3.50083374805331e-08 C 4 0 0 0 0 0 0 0 0 0 0 0

PSTPIP2 0.722501580676782 3.55315440916552e-06 2.88134333771533e-05 C 1 0 0 0 0 0 0 0 0 0 0 0

DHX58 0.721231918949616 4.18909242154611e-10 9.72846040031942e-09 C 4 0 0 0 0 0 0 0 0 0 0 0

CTGF 0.721070531605832 5.93558517621028e-06 4.47402710593915e-05 C 270 51 15 12 6 8 0 21 11 0 0 1

FBLIM1 0.720069971205795 8.19034229834729e-13 3.62193010936493e-11 C 148 5 1 1 2 0 2 2 0 0 0 0

HLA-E 0.719872871575578 8.55361195963657e-09 1.44936628075239e-07 MN 15 1 0 0 0 0 0 1 0 0 0 0

ALDH1L2 0.719682790144507 0.000293090615831197 0.00125193547412256 C 2

AATF 0.719541713073752 2.24213608714347e-12 9.02912972794989e-11 C 33 8 1 1 0 0 5 0 2 0 1 0

SAA2 0.719024175114939 5.01410377281317e-06 3.86565955633543e-05 C 5 0 0 0 0 0 0 0 0 0 0 0

KIF11 0.718253916221435 1.05039644096263e-05 7.31451561372322e-05 C 38 7 7 0 0 0 1 0 0 0 0 0

KIAA0196 0.717167414314742 1.56015613143069e-10 4.00122968829115e-09 C 5 0 0 0 0 0 0 0 0 0 0 0

NCS1 0.716764710550838 2.81848270129619e-09 5.3993342193758e-08 C 1 0 0 0 0 0 0 0 0 0 0 0

CCNE1 0.716255489792223 5.74970811351804e-08 7.81225595581881e-07 C 120 19 5 3 1 0 2 1 10 0 0 0

ACTC1 0.715422342596407 0.00047749357284202 0.00189090692329389 C 4 0 0 0 0 0 0 0 0 0 0 0

ERRFI1 0.715285097595905 1.70657336763465e-06 1.5121869910684e-05 C 26 4 1 0 1 0 1 2 0 0 0 0

BACH1 0.714758993222237 4.73609249575094e-09 8.56979184197687e-08 C 19 4 2 0 0 0 0 1 1 0 0 0

MNAT1 0.7143560515674 1.60929594413187e-11 5.22455305910529e-10 C 14 0 0 0 0 0 0 0 0 0 0 0

CCRN4L 0.713901566667229 1.85957663840812e-09 3.7048638984369e-08 C 38 0 0 0 0 0 0 0 0 0 0 0

PTP4A1 0.713786329507452 4.17305962011927e-09 7.66830091553374e-08 C 39 7 3 0 0 0 0 5 2 0 0 0

KIF20A 0.713088069219084 3.62763335681663e-07 3.95736118411921e-06 C 8 2 0 0 0 0 1 0 2 0 0 0

ARMC1 0.71298817748147 2.68180518683406e-10 6.51585709514869e-09 NA

DOCK4 0.712780252813706 9.57086591078068e-08 1.22978804544023e-06 C 3 1 0 0 0 0 0 1 0 0 0 0

AKIP1 0.71276576782552 5.34439428565457e-12 1.94152304500161e-10 C 5 0 0 0 0 0 0 0 0 0 0 0

ERAP2 0.711945010945489 0.0123814229594916 0.0287255690554422 C 1 0 0 0 0 0 0 0 0 0 0 0

NMI 0.711849819076099 5.46761082432173e-11 1.53996235226097e-09 MN 1 1 0 1 0 0 0 0 0 0 0 0

NAPB 0.711332620535551 5.42130700584903e-08 7.40325235928605e-07 C 4 2 1 0 0 0 1 0 0 0 0 0

MGC45800 0.711116082483587 5.05855995008374e-06 3.8961786899722e-05 NA

DUSP4 0.710971463264711 6.46824373508642e-08 8.68874727991346e-07 C 28 3 1 0 0 0 2 0 0 0 0 0

PRKCQ 0.710068536118603 1.50575899164264e-06 1.35776562338353e-05 C 3 1 0 0 0 0 1 0 1 0 0 0

MLLT11 0.709978572146189 7.52631400022852e-05 0.000399355055483169 C 9 4 1 1 0 0 1 2 0 0 0 0

CDCA3 0.708930071406986 6.19628060780456e-08 8.37094954060792e-07 C 3 1 1 0 0 0 0 0 1 0 0 0

LOC100507460 0.708909001277514 0.000504271320554299 0.00197441938025056 NA

GSG2 0.708904553845656 2.04517763046752e-07 2.39388231365855e-06 C 2

AVL9 0.708056644313372 6.98583091545592e-12 2.4668063756872e-10 NA

LMNB1 0.707764247999601 2.56519069034183e-08 3.82294206268503e-07 C 31 3 0 0 0 0 3 1 0 0 0 0

CD163 0.707269592978391 0.000396020938370691 0.0016115863901402 MN 2 1 0 1 0 0 0 0 0 0 0 0

PLOD3 0.706743432016141 3.12748866722623e-11 9.39586952453823e-10 C 3

CSTF3 0.705972496852763 9.50209161987116e-10 2.03054563663096e-08 C 1 0 0 0 0 0 0 0 0 0 0 0

SP110 0.703799501392687 4.40738896319656e-11 1.26010046686437e-09 C 2 0 0 0 0 0 0 0 0 0 0 0

RAB32 0.703401176296635 3.20869370936128e-07 3.55151730041409e-06 C 2 0 0 0 0 0 0 0 0 0 0 0

FRMD5 0.703088701222037 0.000645272967530481 0.00243094774212767 C 1 0 0 0 0 0 0 0 0 0 0 0

TDRD7 0.702834156060696 1.22266356291991e-08 1.96279501742028e-07 NA

APOBEC3F 0.701864143134644 9.64446040446036e-07 9.27073144109808e-06 C 3

FLJ45248 0.701607767827471 0.000197123816765892 0.00089995397622486 NA

UBD 0.701523692942449 0.00466878711896312 0.0126327567978006 C 17 3 1 1 0 0 1 0 0 0 0 0

SMS 0.701497781304112 9.75369000648216e-08 1.24819533977477e-06 C 821 1 1 0 0 0 0 0 0 0 0 0

LOC100507117 0.701361676381896 1.24085696357435e-08 1.98526625102043e-07 NA

XCL1 0.696786241131053 0.00100960370849164 0.00352951041570395 C 70 11 8 0 0 0 1 3 2 0 0 0

EGFR 0.696463100878059 9.39065861146574e-05 0.000481020285627096 MN 331 53 20 8 4 0 4 12 11 0 0 0

PSMB7 0.696430683549527 5.22235335588394e-13 2.41671105053338e-11 C 6 1 0 1 0 0 0 0 0 0 0 0

DDX21 0.695480443414221 9.11300768192667e-07 8.81358693897936e-06 C 12 0 0 0 0 0 0 0 0 0 0 0

SRGN 0.695437352805836 0.000220925479297572 0.000988757755182109 C 41 1 0 1 0 0 0 1 0 0 0 0

NCAPG2 0.694605135296579 3.2905891037409e-08 4.72541577894568e-07 C 27 2 1 0 0 0 0 0 1 0 0 0

MED10 0.694332715218311 5.23579669449814e-11 1.48128436527304e-09 C 1 0 0 0 0 0 0 0 0 0 0 0

DNMT1 0.693202583831346 1.3935955802397e-09 2.8515225456429e-08 NA

HRASLS2 0.691898692821146 0.00809523413314725 0.0201179748474367 NA

CDCA2 0.691634521395226 4.02316765186349e-06 3.20481877722308e-05 C 3 0 0 0 0 0 0 0 0 0 0 0

EXT2 0.690990674147425 4.89011274466999e-13 2.28531268934244e-11 C 15 0 0 0 0 0 0 0 0 0 0 0

AJUBA 0.690326181643075 4.86045906841501e-06 3.76715433201846e-05 C 5 1 0 0 0 0 0 0 1 0 0 0

SSFA2 0.690070618866848 9.84155203544287e-07 9.42190467247483e-06 MN 1 1 0 0 0 0 0 1 0 0 0 0

CALD1 0.690023551239241 2.9860828391851e-07 3.35018315929202e-06 C 167 4 0 0 0 1 0 3 0 0 0 0

LBH 0.690009519437236 5.94842382552467e-09 1.04633659614968e-07 C 8 0 0 0 0 0 0 0 0 0 0 0

PGM2L1 0.688655654946404 4.8782543906436e-05 0.000272764315662368 NA

TSHZ3 0.688306818887197 1.53798411837918e-06 1.38090253361303e-05 C 1 0 0 0 0 0 0 0 0 0 0 0

SLC9A1 0.688008692575923 2.91901370004041e-09 5.56937220772832e-08 C 82 6 1 0 0 0 2 2 1 0 0 0

NUAK1 0.687586969011843 4.51351937788059e-05 0.000255311958353694 C 13 5 1 1 0 0 0 4 1 0 0 0

HLA-L 0.687552419885756 4.14695323346337e-06 3.29233992658395e-05 C 1

MARVELD1 0.686865392154387 1.45752495444273e-09 2.96949136843245e-08 C 1 1 0 0 0 0 0 0 1 0 0 0

EXOSC9 0.686718541590072 3.70232010264697e-12 1.40428482129858e-10 C 1

STIL 0.685999277802305 5.35903675388694e-09 9.59607271909347e-08 C 468 5 1 2 2 0 0 2 0 0 0 0

NAV1 0.68471550130953 2.68817966581567e-08 3.97493566678853e-07 C 547 0 0 0 0 0 0 0 0 0 0 0

APAF1 0.684447948778913 1.8985972876242e-09 3.77465870408228e-08 C 361 54 8 4 1 0 41 8 1 0 0 0

SSH2 0.68227191788755 9.92876593070511e-09 1.64843642781101e-07 C 1 0 0 0 0 0 0 0 0 0 0 0

ZAK 0.681645646373731 4.93138713445812e-15 3.91184008922147e-13 C 129 5 0 0 0 0 3 3 0 0 0 0

CCND2 0.681639572918775 0.000639026150632321 0.0024102925374687 C 338 29 3 5 2 1 6 4 17 0 0 0

RFX7 0.681544817106631 3.78428921454681e-05 0.000219776747357249 C 1 0 0 0 0 0 0 0 0 0 0 0

STOM 0.681252682467525 1.09953852108782e-07 1.38462844901059e-06 C 15 0 0 0 0 0 0 0 0 0 0 0

TXLNG2P 0.681208990329985 0.00211927548706671 0.00654454676843067 C 1

HENMT1 0.679980863102562 1.99172838346693e-06 1.73961435689334e-05 C 2 2 0 0 0 0 2 0 0 0 0 0

USP31 0.679856019435396 8.2135922723012e-14 4.64055704292074e-12 NA

FGD6 0.679750173806756 9.64914412720604e-12 3.3205336526478e-10 NA

SCD5 0.678393445731436 0.000243558904173323 0.00107130824524483 C 5 2 0 1 1 0 0 0 0 0 0 0

SDC3 0.67810820913292 1.25180525801206e-06 1.1557521033363e-05 C 6 3 1 1 0 1 0 2 0 0 0 0

HOXD13 0.677800075379102 0.000241505960062544 0.00106425688151426 NA

ASPM 0.67690766864968 4.9396509239647e-08 6.7994744027549e-07 C 871 3 2 1 0 0 0 0 1 0 0 0

LITAF 0.676698724167372 3.63882817741281e-13 1.76595130548442e-11 NA

SPECC1 0.676441436765704 7.3872699799263e-10 1.63530829134579e-08 C 51 1 0 0 0 0 1 0 1 0 0 0

FLNA 0.673397635645711 3.35615586667797e-05 0.000198258308641117 C 88 5 2 3 0 0 0 2 0 0 0 0

CENPE 0.67249875754828 3.1760121582763e-11 9.51145286704042e-10 C 18 4 3 1 0 0 0 0 2 0 0 0

SELL 0.671982117993015 0.00506435339936074 0.0135596289135239 C 301 24 5 1 1 0 1 15 3 0 0 0

LEPREL1 0.671555100501394 0.00119344884090533 0.00404230605079012 C 2 1 1 0 0 0 0 0 0 0 0 0

PSMA7 0.671493380468086 8.0310875576948e-13 3.55982187832528e-11 C 42 3 2 1 0 0 0 2 0 0 0 0

RAD54L 0.671336460660044 4.66672808883473e-10 1.07584850837241e-08 C 4 0 0 0 0 0 0 0 0 0 0 0

FSTL3 0.671276482165296 0.000182319778300844 0.000842883840718144 C 5 1 0 0 0 0 0 0 1 0 0 0

RBM19 0.670723235261677 2.5885058772313e-11 8.05800176617709e-10 C 1 0 0 0 0 0 0 0 0 0 0 0

SEMA7A 0.669635948552775 0.000709074896909851 0.00263253443974358 C 4 0 0 0 0 0 0 0 0 0 0 0

ALOX12P2 0.669498709427576 1.2306399458155e-06 1.14010387931718e-05 NA

LPXN 0.667828675765064 8.89444897568418e-05 0.000459582953215327 C 7 1 0 0 0 0 0 1 0 0 0 0

TRAF3IP3 0.667475803120549 0.000136693205910923 0.000659661475848047 NA

DARS2 0.667247462258769 7.72200820096154e-09 1.32127045784479e-07 C 1 0 0 0 0 0 0 0 0 0 0 0

SLC7A6 0.666749891652767 1.65454919139295e-12 6.89772082499876e-11 C 4 1 0 0 0 0 0 1 0 0 0 0

POLA2 0.665899780028746 2.32402184687508e-06 1.98496216136303e-05 C 1 0 0 0 0 0 0 0 0 0 0 0

BMP8A 0.665834082393458 8.30250209716693e-06 5.95007410803024e-05 C 3 0 0 0 0 0 0 0 0 0 0 0

PSMB8 0.665183699594087 2.87188948452338e-09 5.48499013860485e-08 MN 2 1 0 1 0 0 0 0 0 0 0 0

TTPAL 0.664852210954574 5.37060606291404e-08 7.33931126012809e-07 NA

QSOX2 0.664715376461646 5.49952711241973e-11 1.54665006919418e-09 C 1 1 0 0 0 0 1 0 0 0 0 0

SLC38A2 0.664581692399914 3.12313781711818e-07 3.46899233947159e-06 C 13 0 0 0 0 0 0 0 0 0 0 0

VSIG1 0.664081905737226 2.22385259108024e-07 2.58067798843506e-06 C 1 1 1 1 0 0 0 0 0 0 0 0

SEC14L2 0.663489111882812 2.76369345215971e-05 0.000168549659363446 C 636 13 1 3 1 0 2 3 2 0 0 2

TK1 0.663198801060594 2.21613888761789e-08 3.35515888448199e-07 C 106 4 1 1 0 0 0 1 2 0 0 0

FMNL3 0.663067305914358 6.14027279490632e-07 6.21480979621347e-06 C 2 0 0 0 0 0 0 0 0 0 0 0

LCP2 0.66170610310606 0.000100155957446235 0.000508899813848293 C 11 2 0 0 0 1 1 0 0 0 0 0

PTGES 0.661344552772019 7.85188776420299e-05 0.000413157296950431 C 103 13 7 0 0 1 1 2 2 0 0 2

PAG1 0.661164167834947 1.57967974928149e-07 1.9067983810364e-06 MN 7 1 0 0 0 0 1 0 0 0 0 0

APOL4 0.660710957941696 3.08000416541193e-05 0.000184671269847089 NA

XPNPEP1 0.659939951122414 6.34301336439004e-08 8.53870653967356e-07 C 1 0 0 0 0 0 0 0 0 0 0 0

CD70 0.659834223968731 0.00124543435749637 0.00418261943657497 C 100 23 10 0 2 0 5 3 6 0 0 0

IFI16 0.659448283962562 9.89463036572892e-08 1.26281637850405e-06 C 24 6 1 1 0 0 3 0 2 0 0 0

CDK18 0.659333024569865 2.2042698772968e-06 1.89723583299666e-05 C 2 0 0 0 0 0 0 0 0 0 0 0

KIF20B 0.659270076855711 1.61435721607398e-05 0.000107323284259333 C 16 2 1 0 0 0 2 0 0 0 0 0

ZC3H12A 0.659257809796272 2.6763926401901e-06 2.24043815963027e-05 C 2 0 0 0 0 0 0 0 0 0 0 0

CEP72 0.659178962086324 2.52430461065933e-13 1.2658271641573e-11 C 1

IGF2BP1 0.659109687871956 0.017221888111785 0.0376743731266476 C 34 8 1 1 0 0 1 3 3 0 0 0

ELOVL5 0.659000554670254 9.03470737725879e-09 1.51730174382766e-07 C 2 0 0 0 0 0 0 0 0 0 0 0

RAB43 0.658092563390053 1.7709255450001e-14 1.17683051667244e-12 C 3

RHOC 0.657867802710384 4.97305652260524e-14 2.94140752510467e-12 MN 4 2 1 1 0 0 0 2 0 0 0 0

CCDC109B 0.65714176915037 2.1588378144153e-05 0.000137438019890475 NA

DKC1 0.657079079156703 1.80429412456791e-09 3.60230747844903e-08 C 10 0 0 0 0 0 0 0 0 0 0 0

CDCA4 0.656986221672343 3.01244944105797e-09 5.71881951563734e-08 C 2 0 0 0 0 0 0 0 0 0 0 0

TMEM140 0.656135027475572 7.06362483225615e-08 9.38856932585057e-07 NA

GNS 0.65598762466168 1.89256041656557e-10 4.71943227988624e-09 C 19 0 0 0 0 0 0 0 0 0 0 0

LIMS1 0.654965371068617 7.97658855441731e-11 2.18484647712672e-09 C 41 4 0 0 0 0 3 1 1 0 0 0

CD151 0.654225564554324 9.19764152839204e-10 1.98273076546556e-08 MN 4 2 2 0 0 0 0 2 0 0 0 0

STK10 0.653754861681736 2.19826839009868e-11 6.9927102217475e-10 C 13 1 1 0 0 0 0 0 0 0 0 0

C11orf82 0.65324724253161 2.17177145188799e-06 1.87352407793455e-05 NA

MAD2L1 0.653194362681846 1.66160592160144e-09 3.34922420427587e-08 C 79 10 2 4 0 0 4 1 1 0 0 0

TPST1 0.652876955680232 0.000313511073384802 0.00132629058693656 C 1 1 0 0 0 0 0 1 0 0 0 0

CMBL 0.652370601108371 0.000122717685834765 0.000602181537406727 C 2 0 0 0 0 0 0 0 0 0 0 0

RNF175 0.65146696194089 4.00030347168673e-08 5.65449916419826e-07 NA

COX15 0.651208130158301 5.726211162597e-10 1.28981648003164e-08 NA

EFNB1 0.651030985866348 8.77580172418332e-09 1.47907034045964e-07 C 26 6 2 1 0 0 0 4 0 0 0 0

CIT 0.650793190248333 2.50820847608313e-09 4.84911765340402e-08 C 28 2 1 0 0 0 0 0 1 0 0 0

SLC1A5 0.65029096719987 3.41011557536472e-07 3.74380843938098e-06 C 30 1 0 1 0 0 0 0 0 0 0 0

ASPH 0.65006881472375 8.50664758954441e-08 1.10885205872801e-06 C 94 0 0 0 0 0 0 0 0 0 0 0

TMEM194A 0.6500644674081 5.38697935409607e-07 5.55334195179609e-06 NA

ANTXR2 0.648715728045896 4.64972928505931e-05 0.000262233093499158 C 476 1 1 0 0 0 0 0 0 0 0 0

GALNT2 0.648301594513049 1.63429782487223e-07 1.96022528082109e-06 C 4 0 0 0 0 0 0 0 0 0 0 0

HOXA1 0.648253601137012 6.30720863265956e-08 8.50690914585739e-07 MN 2 1 0 1 0 0 0 0 1 0 0 0

ANO6 0.648240184596077 1.77313964319515e-09 3.54384519817895e-08 C 20 3 0 0 1 0 0 1 1 0 0 0

SLC30A7 0.648003531942494 1.7151310141981e-14 1.16352274930923e-12 C 1 0 0 0 0 0 0 0 0 0 0 0

RAB23 0.646915294606224 6.13845320466587e-09 1.07377545106017e-07 C 7 0 0 0 0 0 0 0 0 0 0 0

DHRSX 0.646873486055724 0.00464290635501721 0.0125789133383067 NA

TMEM67 0.646630956554243 3.8558832673936e-05 0.000223249625579562 NA

ANKRD1 0.646023607821053 0.00701867320325511 0.0178288052231928 C 27 2 0 0 0 0 2 0 0 0 0 0

RGS20 0.645594934301416 2.27093078770581e-08 3.42758429177893e-07 C 1 0 0 0 0 0 0 0 0 0 0 0

ATIC 0.645501897678974 2.38964431038779e-11 7.51546991067433e-10 C 79 10 2 0 0 0 4 0 5 0 0 0

SPIRE1 0.645285766015232 6.46357930263796e-09 1.12690432912081e-07 C 1

SFTA1P 0.644837802367851 0.00186370636195374 0.00587906171878307 NA

PUS7 0.64436572193955 3.80209384964356e-07 4.11212744526878e-06 NA

MARVELD3 0.643957409673134 4.15350181586944e-08 5.84485716497851e-07 C 2

CTNNAL1 0.643663342652626 7.30578951864759e-06 5.33063524361769e-05 C 9 0 0 0 0 0 0 0 0 0 0 0

KBTBD2 0.643595134543776 7.73072204788953e-09 1.32127045784479e-07 NA

SAMHD1 0.643512387956787 0.000289740507608569 0.00124014441146707 C 2 0 0 0 0 0 0 0 0 0 0 0

ACER3 0.643374007888518 4.71815299488473e-06 3.66887763903793e-05 C 1 1 0 0 0 0 0 0 1 0 0 0

HIST1H2AD 0.643118152354891 1.04275209917758e-05 7.27623222841448e-05 NA

IKBIP 0.643090707152806 1.19312241725356e-09 2.47883951606565e-08 NA

FAM111A 0.642984235495102 4.75912210719955e-10 1.08966023244347e-08 C 1

KIAA0101 0.642812326680505 6.63772068752993e-08 8.88147658349397e-07 C 143 7 3 2 1 0 0 2 3 0 0 0

ATG5 0.642392858116945 5.81052040013149e-08 7.889219484454e-07 MN 7 2 0 1 0 0 1 0 0 0 0 0

PPFIA1 0.642371815100105 0.000340718451403465 0.00142137494593638 C 19 2 0 0 0 1 0 2 0 0 0 0

FABP6 0.64178789525579 0.000272857528796807 0.0011809683163817 C 4 0 0 0 0 0 0 0 0 0 0 0

PHF15 0.641645870942621 4.30687657740583e-06 3.40218084226044e-05 NA

PRIM2 0.641145370440275 2.85566478726679e-11 8.71760764977395e-10 C 18 6 1 0 1 0 3 1 1 0 0 0

TEAD4 0.640309104445088 8.50811393580938e-11 2.31369356987161e-09 C 3 0 0 0 0 0 0 0 0 0 0 0

SPC25 0.640216658976239 3.95386244637251e-07 4.26166028032418e-06 C 1 0 0 0 0 0 0 0 0 0 0 0

NFE2L1 0.639547008004151 2.12620274395183e-07 2.47952183208727e-06 C 8 0 0 0 0 0 0 0 0 0 0 0

RBBP8 0.639090522426142 4.96921914374359e-06 3.83574268897369e-05 C 614 6 1 2 0 0 2 2 3 0 0 0

GNPNAT1 0.6385665622811 4.00972095208876e-08 5.66358122837194e-07 NA

FRK 0.638307977231143 8.82780747368361e-06 6.2860764505045e-05 C 14 1 0 0 0 0 0 0 1 0 0 0

NUF2 0.637350630188743 1.97478223818395e-07 2.32009332229097e-06 C 5 1 0 0 0 0 0 0 1 0 0 0

NME1 0.636889975237099 3.43181556427279e-08 4.91331113350916e-07 MN 34 22 1 3 0 0 0 21 2 0 0 0

SAMSN1 0.636767415595723 0.0005654402428649 0.0021752210318504 MN 24 2 2 1 0 0 0 0 0 0 0 0

C20orf197 0.636577617576839 0.000472552156756097 0.0018752185666459 NA

ASCC3 0.636521307706942 5.32382461941531e-08 7.28063790257757e-07 C 18 1 0 0 0 0 0 0 1 0 0 0

B3GNT3 0.63646031297661 0.00632586739120446 0.0163319727340509 C 5 0 0 0 0 0 0 0 0 0 0 0

SKA3 0.636246123752392 5.05409628783613e-07 5.25887193182377e-06 C 1 1 0 0 0 0 0 0 1 0 0 0

CASP1 0.635991959036009 1.36033238651188e-08 2.15817360264127e-07 C 622 41 1 3 0 1 31 3 6 0 0 1

EMR1 0.635629125826687 0.0111572290182089 0.0262947171744041 C 2 0 0 0 0 0 0 0 0 0 0 0

FOXD1 0.635516231173722 2.29841611470286e-06 1.96664203449281e-05 C 1 0 0 0 0 0 0 0 0 0 0 0

ZC3H7A 0.635095517840793 4.08556578921129e-11 1.17877292214028e-09 C 1 0 0 0 0 0 0 0 0 0 0 0

LOC100652770 0.635047813355151 0.00238522932084349 0.007211698938595 NA

ABCC3 0.634481547053419 4.51075235687779e-05 0.000255231718560915 C 218 6 4 1 0 0 2 0 0 0 0 0

IFNE 0.633667225907397 0.00269371994287902 0.00796003705837176 C 1

TTI1 0.633402079614395 7.50353608128311e-09 1.28991305549905e-07 C 1 0 0 0 0 0 0 0 0 0 0 0

PLOD1 0.6332638754085 2.34113678300701e-08 3.52511502720555e-07 C 6 0 0 0 0 0 0 0 0 0 0 0

LIMK1 0.632733407382474 3.52245031112414e-14 2.18588252585727e-12 C 54 4 1 0 0 0 0 4 0 0 0 0

PGS1 0.632690960316583 5.91424456693611e-13 2.71696376015533e-11 C 1 0 0 0 0 0 0 0 0 0 0 0

PKP2 0.632657017125771 8.06706231689816e-06 5.79450810140157e-05 C 10 4 1 1 0 0 0 2 2 0 0 0

TMC7 0.631969185366741 1.57283874121646e-05 0.000104783945283365 C 1

HCG4 0.631069441346697 0.0051157143991967 0.0136662140343819 NA

PLEKHA2 0.63058645755291 6.90217677867865e-06 5.08119408362702e-05 C 1 1 1 0 0 0 0 1 0 0 0 0

RARRES1 0.630485048242723 0.0036242048433667 0.0102076376592859 C 23 3 2 1 0 0 0 0 0 0 0 0

C7orf49 0.63048252428495 1.40876085530756e-10 3.63760118804995e-09 NA

ZNF484 0.630462697619439 2.00673853131665e-07 2.35471420844576e-06 C 1

FAM69A 0.629395411566354 6.24173707863178e-07 6.30738695607388e-06 NA

SP140L 0.629364730286094 2.47196509116856e-08 3.7044246461241e-07 NA

BAK1 0.627357750219149 4.28945635264457e-09 7.83653864734593e-08 MN 15 3 0 2 0 0 1 0 0 0 0 0

BIRC3 0.626975885429247 0.00208733477779283 0.00646066808491984 MN 10 3 1 0 1 0 1 0 0 0 0 0

PRSS23 0.626396399781039 8.49161858961526e-05 0.000442391591097297 C 3 1 1 0 0 0 0 0 1 0 0 0

OSBPL9 0.625398282947875 4.28518021501252e-09 7.83653864734593e-08 NA

SLC37A3 0.62497178506671 3.86835128463675e-09 7.18511136058095e-08 NA

RAI14 0.624512355050961 0.0014704997796659 0.00480527440085229 C 2 0 0 0 0 0 0 0 0 0 0 0

RAPGEF1 0.623599052003548 3.54600212367179e-09 6.63849477692739e-08 C 22 3 0 0 0 0 2 0 1 0 0 0

TGIF1 0.622892637937773 5.70517821432168e-09 1.01391462969452e-07 C 16 1 1 0 0 0 1 0 0 0 0 0

ADAM9 0.622569707694594 1.29219933649501e-08 2.05870848837046e-07 C 45 12 2 3 0 0 1 7 2 0 0 0

SERPINB7 0.622558662728885 0.0173731603513824 0.0379429166660612 C 3 0 0 0 0 0 0 0 0 0 0 0

HPRT1 0.622348002705237 7.48807676181858e-10 1.65529476129113e-08 C 141 4 1 0 0 1 0 0 2 0 0 0

SLC36A4 0.621529044460836 6.7571255663357e-05 0.000362917467633473 NA

DNAJB5 0.621197483463579 3.61906435544739e-06 2.92977036165752e-05 C 1 0 0 0 0 0 0 0 0 0 0 0

FADD 0.620739249130743 0.000858726915784041 0.00307950444753357 MN 9 2 0 0 0 0 1 1 0 0 0 0

NID1 0.620437189353824 7.31957944941386e-05 0.00038980776657022 C 38 0 0 0 0 0 0 0 0 0 0 0

NRIP3 0.620050621521969 0.000106212557539171 0.000534934826115992 C 1 0 0 0 0 0 0 0 0 0 0 0

TMEM38B 0.618187475739092 4.96656018458572e-06 3.83525437020212e-05 NA

VCPIP1 0.617660726901406 4.44411142807401e-14 2.64508481129424e-12 C 1

GAST 0.617068964448658 0.0147669199361975 0.0332097782357902 C 2199 152 34 7 18 6 13 52 42 0 0 1

HBEGF 0.616893083338347 0.000129244975477266 0.000629333586534144 NA

MICU1 0.616728555348462 5.49928351422759e-09 9.80084172069545e-08 C 10 1 0 0 0 0 1 0 0 0 0 0

ALCAM 0.615806698459675 7.6948536481519e-05 0.000406249637373978 MN 7 1 0 0 0 0 0 1 0 0 0 0

RAC2 0.61517366955518 2.77519071733138e-07 3.14338927031305e-06 C 92 9 1 0 0 0 2 4 2 0 0 0

LAMB3 0.614364872206008 0.000313706718623219 0.00132682169014115 C 19 3 1 1 0 0 0 2 1 0 0 0

CCL11 0.614218112576379 0.0231776204208765 0.0481804307200363 C 52 9 1 1 1 2 1 1 2 0 0 3

TMEM138 0.613717097128947 1.00525684304985e-10 2.68356787988779e-09 NA

PLEK 0.613689795313691 0.000759633000646915 0.00278896149089947 C 159 2 0 0 0 0 2 0 0 0 0 0

DENND5A 0.613422109212061 9.02941128844538e-07 8.73720181269967e-06 NA

GM2A 0.612461434275722 0.00102792340647896 0.0035809877258287 C 1 0 0 0 0 0 0 0 0 0 0 0

HOXB9 0.612004158282474 0.00657207503483303 0.0168663951436318 C 14 3 1 0 0 0 1 2 1 0 0 0

TBP 0.610809547884852 1.4002604811513e-10 3.62554447698367e-09 C 73 1 0 0 0 0 1 0 0 0 0 0

CAV2 0.610688624143163 1.77247686716358e-06 1.56545355412063e-05 C 292 8 3 3 1 0 0 2 1 0 0 0

BCL2L11 0.610419357119556 1.07485166209443e-14 7.91584334959583e-13 MN 15 1 0 0 0 0 1 0 0 0 0 0

FAM208B 0.61004986935097 4.85626842866246e-05 0.000271856233508709 NA

LOC440354 0.609904092905885 0.0030554976318795 0.00883596694859943 C 4 1 0 0 0 0 0 0 0 1 0 0

SYNC 0.609575627020575 4.39132494299799e-07 4.64846796398898e-06 C 3

IL23A 0.60951055248166 0.00028127160338659 0.00120991537211318 MN 4 1 0 0 0 0 0 0 1 0 0 0

FAM96A 0.609288520458537 1.77142651392004e-05 0.00011633514791452 NA

C5AR1 0.608768618554974 5.98958029055188e-05 0.00032707670559514 C 56 6 2 0 0 0 3 1 0 0 0 0

SKA2 0.608192685184097 1.1985507865465e-08 1.93063580740134e-07 C 2 1 0 0 0 0 0 0 1 0 0 0

PSMB4 0.608097549936166 8.2933515785061e-11 2.26505433371407e-09 C 8 1 0 0 0 0 0 1 0 0 0 0

HEXB 0.608055140049318 6.83141271439083e-09 1.18404897843659e-07 C 2 0 0 0 0 0 0 0 0 0 0 0

MLKL 0.607733361380057 1.89688418856029e-05 0.000123422310199744 C 3 0 0 0 0 0 0 0 0 0 0 0

FAM76A 0.607690064075818 2.40792714663425e-11 7.55801610354003e-10 NA

VPS13C 0.607244214943331 2.13276237204321e-08 3.24752963923265e-07 C 1 0 0 0 0 0 0 0 0 0 0 0

UCHL1 0.606935361410251 0.000197331513106764 0.00089995397622486 C 195 19 3 3 6 0 3 8 1 0 0 0

RBM28 0.606893974447899 1.62255492799819e-13 8.46008185185172e-12 NA

GMFG 0.60684919442691 0.000518510902636232 0.00202180796336959 C 1 0 0 0 0 0 0 0 0 0 0 0

MYH9 0.606387980549615 3.30955279056662e-06 2.70933848040893e-05 C 39 3 0 0 0 0 0 3 0 0 0 0

DDHD1 0.606285813890795 9.46177975374692e-08 1.21991216211967e-06 NA

FAM72D 0.606276074518301 1.65889297469686e-05 0.000109975016925 NA

SYNPO 0.605619934286556 1.03938846345915e-07 1.31588665203287e-06 C 5 2 0 0 0 0 0 1 1 0 0 0

TAF2 0.605128203845814 3.39159072197293e-08 4.8593972441167e-07 C 1

UBE2T 0.605116955271989 1.91085836021521e-08 2.93323732228656e-07 C 60 1 0 0 0 0 0 0 1 0 0 0

MCL1 0.604860195641299 0.000185572715923864 0.000855415195881875 MN 21 6 1 0 1 0 4 0 1 0 0 0

FKBP15 0.604676227901687 2.93528382409123e-11 8.91751475739563e-10 C 7 1 0 0 0 0 0 1 0 0 0 0

CDCA8 0.60418489231156 6.41479401474644e-08 8.62919732175592e-07 C 20 1 1 0 0 0 0 0 0 0 0 0

PRF1 0.604178423179787 0.00095421505684278 0.00336258208543349 NA

C6orf170 0.603814756827667 5.35001248149923e-07 5.52426002385902e-06 NA

PRR5-ARHGAP8 0.602982126640359 0.0015279118367506 0.00495792034033704 NA

HIST1H2AL 0.602785275964684 4.74569620616339e-05 0.000266691781751943 C 5 1 0 0 0 0 0 0 1 0 0 0

RECQL 0.602490732001077 2.22909580282132e-05 0.000141009680013366 C 12 6 4 0 0 0 1 0 2 0 0 0

GPR158 0.601780724565064 0.00454314748477105 0.0123457505304037 C 1 0 0 0 0 0 0 0 0 0 0 0

PRPF4 0.601127168521392 1.86106148882477e-08 2.86376510560865e-07 C 2 0 0 0 0 0 0 0 0 0 0 0

CAB39 0.60079559874799 1.16850873824681e-06 1.08840378389751e-05 C 7 1 0 0 0 0 0 1 0 0 0 0

NCAPD2 0.600495958250888 3.78097274649337e-08 5.37209778873961e-07 C 2 0 0 0 0 0 0 0 0 0 0 0

GZMA 0.600234928964197 0.00451847436785443 0.0122928222452754 C 51 6 2 0 0 0 3 0 2 0 0 0

EFHD2 0.598883124189983 5.02029341866839e-09 9.00654915024992e-08 NA

BCL2A1 0.598813507429489 0.000133530324739355 0.000646540920015803 MN 30 5 0 0 0 0 1 4 1 0 0 0

MACF1 0.597788985287534 6.47981593129233e-07 6.50628520591883e-06 C 4 0 0 0 0 0 0 0 0 0 0 0

CCNB2 0.597674258602076 4.63106955386686e-07 4.8668658215457e-06 C 30 3 0 1 0 0 0 0 2 0 0 0

NOP2 0.595879492916891 4.7522263432384e-08 6.57976503280711e-07 MN 4 2 0 1 1 0 0 0 1 0 0 0

SPC24 0.595665733483241 5.84863023443201e-06 4.41727950706683e-05 C 2 0 0 0 0 0 0 0 0 0 0 0

CHMP5 0.595064629000781 3.09613492953234e-07 3.44506442159075e-06 C 6 5 1 0 0 0 4 0 0 0 0 0

TIMELESS 0.594801094514987 8.23867056763035e-08 1.0791233068065e-06 C 81 3 0 0 0 0 0 1 2 0 0 0

SLC35D2 0.594663113270775 0.000277098374188005 0.00119440695246103 C 2 0 0 0 0 0 0 0 0 0 0 0

SNRPB 0.59432635923069 2.83537296630601e-07 3.19815876837151e-06 C 17 1 0 1 0 0 0 0 0 0 0 0

MPHOSPH6 0.594072861607604 3.24954284782763e-06 2.66713345537006e-05 C 121 13 0 0 0 0 13 0 0 0 0 0

INTS8 0.593977552459567 9.7419920679917e-09 1.62169466904907e-07 NA

JUN 0.593605250424368 0.000154865579029564 0.000734141869664576 MN 65 7 1 0 0 0 4 1 2 0 0 1

SKIL 0.593330245535229 7.52717575033915e-08 9.93492715667148e-07 C 67 8 1 0 0 0 3 2 3 0 0 0

ADAMTS2 0.592497736154522 0.000216498949530121 0.000971934444439421 NA

RAD51AP1 0.591622191034689 2.76769714549815e-07 3.13706632538377e-06 C 5 0 0 0 0 0 0 0 0 0 0 0

SYNE2 0.591482188105179 0.00035216590720864 0.00146107937872379 C 6 0 0 0 0 0 0 0 0 0 0 0

YKT6 0.591450086517276 7.15266753027391e-09 1.23285906890914e-07 C 2 0 0 0 0 0 0 0 0 0 0 0

ZC3H12C 0.591426978935829 1.20079734787916e-05 8.21970755996706e-05 NA

POLR2K 0.590717271823721 2.06986085831921e-06 1.79790070974795e-05 C 1 0 0 0 0 0 0 0 0 0 0 0

IL1RAP 0.590424832215004 3.85093321005777e-08 5.45150432810497e-07 C 8 3 3 1 0 0 1 0 0 0 0 0

INPP4A 0.590072902652025 2.0870849414856e-08 3.18140065591436e-07 NA

CXCL2 0.590039617971557 0.00203216058524367 0.00632067935386348 C 57 5 1 0 0 0 0 2 2 0 0 0

FLJ43663 0.589985039099604 9.15989112032056e-06 6.49566351571027e-05 NA

SESTD1 0.589566221914619 0.00104559876152851 0.00362539828503296 C 7 0 0 0 0 0 0 0 0 0 0 0

CYP24A1 0.589260243080947 0.00283334872649193 0.00830393176622992 C 75 11 1 0 0 0 1 3 7 0 0 0

ACLY 0.588538624236233 4.72870680914878e-07 4.95571615596672e-06 C 69 3 2 0 0 0 1 1 1 0 0 0

THBS2 0.587818952170046 0.00159729059931586 0.00515111930026431 C 55 9 0 0 0 3 1 4 2 0 0 0

BTN3A1 0.587413716467386 9.25818184338538e-06 6.55063954204692e-05 C 1 0 0 0 0 0 0 0 0 0 0 0

KIAA1429 0.587290809640328 6.31475513424179e-09 1.10257721795013e-07 NA

TAF1D 0.587133937499371 1.89614801629816e-05 0.000123422310199744 NA

HIST1H3I 0.586639713202834 4.70669301484696e-05 0.000264814443198598 C 2 0 0 0 0 0 0 0 0 0 0 0

GRINA 0.586192174023276 3.69067854407313e-10 8.66668397067892e-09 NA

PYGB 0.586040202288943 2.29903804564844e-05 0.000144382696315835 MN 4823 2 1 0 1 0 0 0 0 0 0 0

MRPL28 0.585932410739329 3.83904352889192e-08 5.43874078378273e-07 MN 9 3 0 1 0 0 0 2 1 0 0 0

RNASEH2A 0.584966475504224 2.59698714627963e-07 2.95925199985759e-06 C 198 26 5 1 0 5 4 5 9 0 0 2

GPRC5D -0.584979851989856 0.00184093732761449 0.00581596074107152 C 24 0 0 0 0 0 0 0 0 0 0 0

FAM69C -0.585119005344423 3.25173636462309e-07 3.59285546837252e-06 NA

PTGIS -0.58514343942511 9.69186403162243e-05 0.000494441807349105 C 13 2 0 1 0 0 0 1 0 0 0 0

STOX1 -0.585492995016406 8.9076827686193e-05 0.000460141134720681 C 2

SLC38A4 -0.585536625984631 0.00126714955288437 0.00423958628026205 C 2 0 0 0 0 0 0 0 0 0 0 0

ANG -0.585983689322564 1.34566649273487e-05 9.1255570433511e-05 C 525 66 17 5 2 25 5 13 19 0 0 1

C15orf62 -0.586055427029166 0.0137253665773926 0.0313108655039501 NA

GPRC5B -0.586126854543426 5.7235546653872e-06 4.34187251109353e-05 NA

KCTD19 -0.58652708891428 4.84063404172248e-05 0.000271141404284349 NA

MRAP -0.586621343901738 5.8466734807322e-07 5.97516139145887e-06 C 36 0 0 0 0 0 0 0 0 0 0 0

ATOH1 -0.586656060832733 1.14179889376311e-05 7.86420220606057e-05 C 23 6 2 0 0 0 0 0 4 0 0 0

FMO3 -0.586727753062841 0.000785187417751504 0.0028630383758262 C 1 0 0 0 0 0 0 0 0 0 0 0

OR1D2 -0.586861987691905 0.000676976235941157 0.00253073853795344 NA

EPS8L1 -0.587036976310358 0.00379752901785925 0.0106011551210947 NA

FGFR3 -0.587402139645928 0.000343210806659486 0.00143019615535977 MN 9 1 1 0 0 0 0 0 1 0 0 0

SLC5A5 -0.587475465868238 3.51184412391266e-06 2.852732778253e-05 C 180 5 2 1 0 0 0 2 1 0 0 0

FLJ38379 -0.587760743465967 5.06100095609614e-06 3.8961786899722e-05 NA

SYNGR4 -0.587950519233416 9.34925272732173e-05 0.000479418332078077 NA

CD36 -0.587998717398604 0.00196145897159993 0.00613730103413324 NA

ZNF701 -0.588118329321929 5.42196865702084e-06 4.13963698150196e-05 NA

EFS -0.58814388936711 1.45964864880672e-08 2.29267800630413e-07 NA

TFF3 -0.588264889270536 0.00127981898940479 0.00427291127402795 NA

RFPL2 -0.588797610270402 0.000549343918162634 0.002124092408389 NA

XYLB -0.588914119884857 3.35851838667666e-08 4.81930837790972e-07 NA

ISX -0.589504711217863 1.76451234321424e-05 0.00011593222426302 C 3 1 1 1 0 0 0 0 0 0 0 0

CAPS -0.589663970924932 5.84559974955729e-06 4.41675315209065e-05 C 64 4 2 0 1 0 0 1 0 0 0 0

RGS22 -0.589805729958728 4.07025227239966e-05 0.00023373077900397 C 1 1 1 1 1 0 0 1 0 0 0 0

AOC4 -0.590048505475936 3.60001724947917e-08 5.14245482874659e-07 C 4 1 0 0 0 0 0 0 0 1 0 0

ITIH5 -0.590102575198269 1.7473029462087e-08 2.70189565873301e-07 C 5 3 1 3 0 0 0 2 1 0 0 0

CAMKK1 -0.590141120441655 1.24348275008176e-06 1.15031270825012e-05 C 3 0 0 0 0 0 0 0 0 0 0 0

MPV17L -0.590951068113486 7.2896723035756e-06 5.3229794633401e-05 NA

LOC100509780 -0.591288235454381 1.605879247893e-06 1.43370172287127e-05 NA

CTSF -0.591531733629116 5.81911341055428e-06 4.40377287171375e-05 C 2 0 0 0 0 0 0 0 0 0 0 0

SGSM1 -0.591564457531918 5.97945504948624e-06 4.501716218044e-05 C 1

SVEP1 -0.591579139580198 2.50893002715131e-06 2.11709846740494e-05 C 2 0 0 0 0 0 0 0 0 0 0 0

RAMP3 -0.59226301619088 2.89741354983295e-05 0.000175317603125602 C 11 0 0 0 0 0 0 0 0 0 0 0

ERBB3 -0.592390919843347 0.000340508094711089 0.00142081056185996 MN 18 3 0 0 0 0 1 2 1 0 0 0

MIR210HG -0.592745256481156 1.92407393218471e-05 0.000124634205003574 C 4 1 0 0 0 0 0 0 0 1 0 0

PTGFR -0.592758498469449 1.67914362135394e-05 0.000111122906718063 C 11 0 0 0 0 0 0 0 0 0 0 0

MTMR8 -0.592849066903711 1.44816097596785e-08 2.27841585969606e-07 NA

NCOA1 -0.592928588242678 5.77852608470449e-09 1.02215105799254e-07 C 43 1 0 0 0 0 0 0 1 0 0 0

RGS14 -0.592939717014789 8.327459145138e-11 2.27109249625399e-09 C 1 0 0 0 0 0 0 0 0 0 0 0

HOGA1 -0.592962234788924 8.36344015930046e-06 5.98921043870903e-05 NA

TBX6 -0.593945880483753 1.16112037218234e-06 1.08365509291396e-05 NA

PPM1D -0.593989429693561 9.4050218237649e-08 1.21342091382685e-06 MN 1 1 1 0 0 0 0 0 0 0 0 0

ZNF233 -0.594309561142567 3.42389128912931e-07 3.75675306836815e-06 NA

ZFP30 -0.5946142691875 1.9015083069662e-09 3.77647929967988e-08 NA

NKAIN1 -0.594796349531687 0.000260564850437409 0.00113503128290652 NA

HP07349 -0.594873936118117 3.74413948438139e-07 4.06105031638319e-06 NA

SIGLEC8 -0.595019080147206 0.000187743406324115 0.00086415842692036 C 2 0 0 0 0 0 0 0 0 0 0 0

SH3GLB2 -0.595028651817437 2.10651002704134e-09 4.15311617518869e-08 C 4 1 0 0 0 0 0 1 1 0 0 0

PDZD4 -0.595044092003603 6.61616924487334e-06 4.89922673308754e-05 C 4 1 0 0 0 0 0 0 1 0 0 0

ZNF256 -0.595047912069585 0.000328825395662364 0.00137844479816203 C 1

YOD1 -0.595296099813114 2.29519426971851e-05 0.000144274798880645 NA

STH -0.595306903693622 4.11419981971955e-05 0.000235825136244191 C 10 0 0 0 0 0 0 0 0 0 0 0

TEPP -0.595661692293628 3.75231593030214e-07 4.06758783578629e-06 NA

PRDM14 -0.596002682519825 1.41736394430617e-05 9.53998839754012e-05 C 1 1 1 0 0 0 1 0 1 0 0 0

KANK1 -0.596038659013228 3.15294194265883e-06 2.59798572697883e-05 C 3 0 0 0 0 0 0 0 0 0 0 0

WNT5A -0.596160629249997 0.000499698820715864 0.00196030696194251 C 150 42 8 4 2 0 2 27 7 0 0 1

IYD -0.59652832884174 3.48440166905288e-05 0.000204875024511227 C 3

ZNF135 -0.596550668964823 9.74241075618086e-08 1.24759545590146e-06 MN 6 1 1 0 0 0 0 1 0 0 0 0

RMND5B -0.596616361470161 6.28974146063817e-10 1.40384359228182e-08 NA

PKP1 -0.597309260189339 0.000843899798217618 0.00303544117842358 MN 1 1 0 0 0 0 0 1 0 0 0 0

PLAGL1 -0.597470845877138 3.00559493157116e-05 0.000181166518560942 C 34 8 1 0 0 0 3 3 2 0 0 0

CRISPLD1 -0.597508795226243 0.00103975609227567 0.00361157341870096 NA

CEACAM3 -0.597538992547039 0.000505079057117693 0.00197644894488258 MN 34 1 0 0 0 0 0 1 0 0 0 0

SPIN3 -0.597902138778023 5.62263334749475e-06 4.27559587657827e-05 NA

SCAMP2 -0.598035306800297 5.62099359076643e-08 7.65936254085214e-07 NA

ACACB -0.598173421002308 6.30061619262259e-06 4.70719529212561e-05 C 13 0 0 0 0 0 0 0 0 0 0 0

STEAP4 -0.598337409365956 0.0238267460163199 0.0493779504928159 C 5 0 0 0 0 0 0 0 0 0 0 0

ALPL -0.598787946663841 1.42090192657512e-07 1.73842344953376e-06 NA

ZNF818P -0.598850483059619 0.000485472616286309 0.00191467810136507 NA

CCDC85A -0.598892834292871 1.02813150189824e-05 7.18856480843295e-05 NA

OLFM2 -0.599335979203427 2.1833276731993e-06 1.88177790849923e-05 NA

SLC4A10 -0.599570992380036 4.60286110966745e-07 4.84259878947614e-06 NA

LDHD -0.599626678202023 0.00110875904255315 0.00380447469151622 MN 2 1 0 0 0 0 1 0 0 0 0 0

ISM1 -0.599746497429294 0.000753988036666487 0.00277263096366555 C 21 2 1 0 0 2 0 0 1 0 0 0

PLCB4 -0.59980763934293 9.37867751304752e-05 0.000480666745977391 C 30 0 0 0 0 0 0 0 0 0 0 0

IZUMO1 -0.599873865574807 0.000481139366586113 0.00190194753370413 C 2 0 0 0 0 0 0 0 0 0 0 0

TMCO5A -0.599888810186755 3.96492010604436e-05 0.000228444574877021 C 1

PLXNA2 -0.600111963149557 2.78413727026931e-06 2.31809643279607e-05 NA

CNNM1 -0.60021601979542 3.94253113999176e-05 0.000227431535771485 C 1 0 0 0 0 0 0 0 0 0 0 0

MDFIC -0.600315563835939 3.33991757008732e-07 3.67740662298096e-06 C 44 0 0 0 0 0 0 0 0 0 0 0

CYP4F2 -0.600338601607239 2.53507964533873e-05 0.000156444253170284 C 1 0 0 0 0 0 0 0 0 0 0 0

PARM1 -0.600525124615517 0.000318600853166362 0.00134511674054868 C 3 0 0 0 0 0 0 0 0 0 0 0

GFRA3 -0.600682659728213 1.19387751758879e-05 8.18121642845874e-05 C 2 0 0 0 0 0 0 0 0 0 0 0

ACAA2 -0.600819440641691 1.81289218666985e-05 0.000118605635731421 NA

IL11RA -0.600885806814051 3.16686827297041e-08 4.5650659407853e-07 C 3 0 0 0 0 0 0 0 0 0 0 0

RAB11FIP1 -0.600970843263931 0.000138491817294869 0.000665794926578608 C 27 2 1 0 0 0 0 0 1 0 0 0

CFP -0.601772918728493 6.87083997773811e-06 5.06600655468053e-05 C 100 2 0 0 0 1 0 0 1 0 0 0

MAPK4 -0.601994120863118 1.67739776042184e-06 1.48982202775712e-05 C 17 3 1 0 0 0 1 1 1 0 0 0

C19orf57 -0.602122860912587 1.0156034221753e-06 9.67404427353391e-06 NA

BCL9 -0.602156426641589 8.21632318084619e-07 8.04437324858573e-06 C 20 4 3 0 0 0 0 1 1 0 0 0

OSGIN1 -0.602284050061435 3.13467264631587e-05 0.000187693607013036 C 6 3 0 1 1 0 3 0 0 0 0 0

LOC100506795 -0.602576671193427 6.52441522576788e-08 8.75177937477736e-07 NA

TCEAL2 -0.602978759744758 1.32705481357078e-05 9.01549406190027e-05 C 1 0 0 0 0 0 0 0 0 0 0 0

ZNF214 -0.602979617998844 0.000110107473195581 0.000550014290095739 NA

ADCY6 -0.603274093357501 7.77309645299791e-06 5.61961025843742e-05 C 2 0 0 0 0 0 0 0 0 0 0 0

KIAA2022 -0.603732913909945 5.01083578077148e-07 5.21959762370181e-06 NA

NEFM -0.603821484470252 0.0158632491918741 0.0352274689023351 C 15 0 0 0 0 0 0 0 0 0 0 0

WSCD2 -0.603850404536083 1.96913008699788e-08 3.01534993176446e-07 NA

SPACA3 -0.603951433610538 2.9027960992463e-05 0.000175587158103019 C 1 0 0 0 0 0 0 0 0 0 0 0

SYTL1 -0.603974020761604 3.25751176050859e-09 6.15933317593867e-08 C 2 0 0 0 0 0 0 0 0 0 0 0

SYT13 -0.60465467866575 0.000322979526723996 0.00135935812815323 C 2 0 0 0 0 0 0 0 0 0 0 0

NUPR1 -0.604675371456473 2.7925739666083e-05 0.000169897291758262 C 43 7 1 2 0 0 3 1 3 0 0 0

RAX -0.604718406559397 1.02546693416739e-06 9.75817630114946e-06 C 1 0 0 0 0 0 0 0 0 0 0 0

GPR133 -0.60481290668287 4.2332336694672e-05 0.000242061672694881 C 1

HMGCLL1 -0.60545954364112 2.6309102379597e-07 2.99430174827801e-06 C 1

FOXI3 -0.606147021400061 4.25205781216525e-06 3.3602796747746e-05 NA

SIAE -0.606293642961858 1.48663692241137e-06 1.34180147975584e-05 C 4 0 0 0 0 0 0 0 0 0 0 0

TTLL12 -0.606351581513869 2.97206682512235e-05 0.000179261659652934 C 2 0 0 0 0 0 0 0 0 0 0 0

WNT2B -0.607007016135566 0.00108965134718984 0.00374774323973506 C 11 3 1 0 0 0 1 1 1 0 0 0

FAM43B -0.607078888613718 4.92974140238004e-09 8.86931706490941e-08 C 1 1 0 0 0 0 0 0 1 0 0 0

GOLGA6L10 -0.607246370458322 1.37340271656507e-06 1.25334586385859e-05 NA

P4HTM -0.60732067639738 1.27913485273641e-07 1.58236505606157e-06 C 80 0 0 0 0 0 0 0 0 0 0 0

PNPLA7 -0.607403066123084 1.43787140987611e-09 2.93260691537987e-08 NA

GFRA2 -0.607415024098215 2.28478115726508e-06 1.95674447798897e-05 C 3 0 0 0 0 0 0 0 0 0 0 0

SLC22A10 -0.607693350056952 1.24030345209833e-07 1.53935891395837e-06 NA

CYTL1 -0.608038511383999 0.00042971250998891 0.00173082968217921 C 44 4 1 0 0 0 1 1 1 0 0 0

GKAP1 -0.608384117063831 4.85036902719315e-05 0.000271606315318594 C 1

CD1E -0.608552303908688 1.81810330353129e-06 1.60275925598214e-05 C 297 14 1 3 4 0 0 5 3 0 0 1

SOX5 -0.608734974291711 2.33889623670419e-12 9.33930149200426e-11 C 6 1 0 0 0 0 0 0 1 0 0 0

HSPB2 -0.609056615934763 1.22301735801174e-07 1.52089681570882e-06 MN 11 4 0 1 0 0 3 0 0 0 0 1

RPL3 -0.609124450589527 1.89671553813078e-07 2.24369593688758e-06 C 15 0 0 0 0 0 0 0 0 0 0 0

FUT2 -0.60934285109434 0.00372140124158189 0.0104237092218407 C 12 0 0 0 0 0 0 0 0 0 0 0

GALR1 -0.610429323521959 4.38557589696679e-07 4.64498013440909e-06 MN 2 1 0 0 0 0 0 0 1 0 0 0

SYT9 -0.610685393217793 6.51918881994911e-06 4.84112391637005e-05 C 1 0 0 0 0 0 0 0 0 0 0 0

NCK2 -0.610954106171714 9.39007714429652e-10 2.01732111362202e-08 C 3 1 0 0 0 0 0 0 1 0 0 0

NMNAT3 -0.610986868301646 0.00393285182241866 0.0109226214122747 NA

SMOC2 -0.611057010890144 0.00219923709807455 0.00674962875875741 C 3 2 0 0 0 0 1 1 0 0 0 0

DIO3OS -0.611398288279717 0.000588587704108399 0.00224787907998614 NA

SCRG1 -0.611542871219906 3.30355717632022e-06 2.70560046197373e-05 NA

MBD3L2 -0.612016624582841 8.14785931247163e-06 5.84810516523134e-05 C 1

MARC1 -0.612436375995189 0.000842146966006871 0.00303028814175134 NA

FAM149A -0.612565781178724 1.13186351045558e-08 1.82944326749725e-07 NA

CD79B -0.612568909894719 0.00016874014482777 0.000789553701150855 C 47 4 2 0 2 0 0 0 0 0 0 0

PIP5K1B -0.612637335482644 0.000198159445889295 0.000902880075191788 C 2 0 0 0 0 0 0 0 0 0 0 0

KIAA1683 -0.613151895663649 6.06648950272468e-06 4.556366937225e-05 NA

SP5 -0.613164197818053 0.00383645383351772 0.0107003480263764 C 9 0 0 0 0 0 0 0 0 0 0 0

TMEM59L -0.613303672371101 8.5733631509985e-08 1.11677938306228e-06 NA

MYO1A -0.613767168922903 0.000375220838162979 0.00153785292419028 C 7 0 0 0 0 0 0 0 0 0 0 0

ND6 -0.613770754563986 7.11581569151903e-06 5.21615195946478e-05 C 9 1 0 0 0 0 0 0 1 0 0 0

MOBP -0.613848660807888 3.5139760212688e-07 3.84890186079598e-06 C 1 0 0 0 0 0 0 0 0 0 0 0

ZNF630 -0.614128347523951 1.11369504204384e-05 7.6958401098079e-05 NA

NNAT -0.614227422023952 1.20744646653984e-07 1.50449896459509e-06 C 23 3 1 1 0 0 0 1 2 0 0 0

FGF11 -0.614525981894726 1.03558555606726e-05 7.23526003620068e-05 C 2 0 0 0 0 0 0 0 0 0 0 0

HMGCR -0.614564680831095 2.2136072103516e-08 3.35443904486186e-07 C 276 20 12 0 1 0 4 0 5 0 0 1

ZNF880 -0.614743498797259 2.80536083205689e-07 3.1726399245592e-06 NA

ZNRF4 -0.615004700648312 9.87383862435053e-07 9.44803557346221e-06 NA

KRT35 -0.615280578952841 2.19979138097779e-08 3.34152901025414e-07 C 8 0 0 0 0 0 0 0 0 0 0 0

GNAZ -0.615293982060312 4.0154339199566e-08 5.66742116353606e-07 C 3 0 0 0 0 0 0 0 0 0 0 0

APLNR -0.615810659284011 0.000111380747368227 0.000555348631569662 C 5 1 0 0 1 1 0 0 1 0 0 0

FBXL14 -0.61585673600026 8.84962029076256e-08 1.14736016489399e-06 NA

MATN2 -0.615985112397363 0.000785229154589413 0.0028630383758262 C 3 0 0 0 0 0 0 0 0 0 0 0

LOC100128239 -0.616027328062294 4.51570316456848e-05 0.000255359168795302 NA

PSD3 -0.616475710948311 0.000179352938073074 0.000830994628863909 C 2 1 0 0 0 0 0 1 0 0 0 0

PCDHA11 -0.616681202045931 1.14336977917e-05 7.87215707906535e-05 NA

FAM129B -0.616754714520401 7.68318544946841e-08 1.01337735889957e-06 C 4 1 0 0 0 0 0 1 0 0 0 0

ACTL7A -0.6171000071473 0.000233320179719199 0.00103320570012139 C 1

NPBWR2 -0.617531535938215 1.95434905343997e-05 0.000126288714695999 C 1 1 0 0 0 0 1 0 0 0 0 0

COCH -0.617750054215673 0.000780320086432246 0.00285118113434423 C 1 0 0 0 0 0 0 0 0 0 0 0

NEURL1B -0.617991300246683 2.26502938704803e-13 1.14320563223088e-11 C 1 0 0 0 0 0 0 0 0 0 0 0

EFHD1 -0.618220247057604 1.09092420780414e-09 2.29224995380798e-08 C 3 1 0 0 0 0 0 0 1 0 0 0

APBB1 -0.618453790087581 5.50589350983448e-10 1.24653165622772e-08 C 9 1 0 0 0 0 1 0 0 0 0 0

GPR143 -0.618810358385985 0.000154303461856212 0.000732138787303214 C 2 0 0 0 0 0 0 0 0 0 0 0

FRAS1 -0.618850681433978 4.99896471248564e-07 5.21210770535596e-06 C 1 0 0 0 0 0 0 0 0 0 0 0

SLC27A5 -0.619198881564663 7.79323499119908e-05 0.000410756220212824 NA

GRIN2C -0.619528079005523 1.06632659223735e-07 1.34728727712126e-06 NA

HBM -0.619637413577094 3.33845435557598e-06 2.72592431354558e-05 NA

MS4A1 -0.619890110715547 0.00703424823796198 0.0178659710681571 C 1520 82 40 8 12 1 15 5 14 0 0 0

SLC13A4 -0.620375692158508 0.000303316273873179 0.00128834540296177 NA

ITGA10 -0.620482809464487 1.21613406729398e-07 1.51333132752617e-06 C 1 0 0 0 0 0 0 0 0 0 0 0

KCNH1 -0.620580042552848 2.10488021029838e-06 1.82329829475137e-05 C 43 14 7 5 4 0 1 2 5 0 0 0

NAP1L2 -0.621168693266027 0.0161475376897561 0.0357330113239815 C 1

MCF2L-AS1 -0.622001750795736 6.75112243011131e-07 6.73581941142418e-06 NA

MAP2K6 -0.622217156633643 6.87257351733297e-08 9.1668216323158e-07 C 47 5 1 0 0 0 0 3 1 0 0 0

SIX3 -0.622347931578768 0.00031847003266523 0.00134486441505016 C 3 2 0 0 0 0 0 0 2 0 0 0

CLDN5 -0.622540840657585 0.000135496049238208 0.000655220675506274 C 38 6 0 0 1 0 0 5 0 0 0 0

VAT1 -0.622757599616026 0.000114523089819495 0.00056891824698519 C 2

MAP6 -0.623097142569908 6.18095049378192e-08 8.35716879628272e-07 NA

CBX7 -0.62325294207604 1.46154095254924e-06 1.32230332738525e-05 C 11 2 0 1 0 0 0 0 2 0 0 0

RPH3AL -0.623616984035291 1.60075304264433e-05 0.000106606097248871 C 2 0 0 0 0 0 0 0 0 0 0 0

CA11 -0.623813025072252 4.7594376146444e-06 3.69839308427542e-05 C 4 0 0 0 0 0 0 0 0 0 0 0

NTRK2 -0.623977004464967 0.000126627346155187 0.000618604136043449 C 203 49 19 6 2 6 8 18 9 0 0 0

CCL21 -0.624195226209162 0.0133190125961465 0.0305155491353668 C 333 23 6 0 0 0 2 10 9 0 0 1

SOD3 -0.624669737350995 5.66352950499723e-06 4.29978431372173e-05 NA

TSC22D3 -0.6248496049921 1.90043459850961e-05 0.000123606617340177 C 47 1 0 0 0 0 0 0 1 0 0 0

BARX1 -0.62489187855543 0.00130450028764017 0.00434766394354511 C 2

ZNF454 -0.625108982654886 1.01175977819426e-07 1.28606966567379e-06 NA

EMCN -0.625178993830174 0.000277194364674658 0.00119454866580083 C 2 1 0 0 0 1 0 0 0 0 0 0

SPATA19 -0.625201723485422 0.00012997334348432 0.000632229625321957 C 1 1 1 0 0 0 0 0 0 0 0 0

PLA2G1B -0.625492185798897 2.656730095052e-07 3.02187082386113e-06 C 302 18 5 1 1 2 5 4 2 0 0 2

RETSAT -0.625528652886148 1.47669002581786e-06 1.33345954764574e-05 NA

ABCA2 -0.625559967338816 8.19983307619189e-09 1.39441366247155e-07 C 5 1 0 0 0 0 0 1 0 0 0 0

N4BP3 -0.625628842801227 3.05540160879804e-12 1.18260912576115e-10 NA

ID4 -0.625671142673117 2.34319484591042e-05 0.000146755952510081 C 44 10 3 0 1 3 2 1 3 0 0 0

XPNPEP2 -0.625831466981703 1.00462425266796e-08 1.66210867397259e-07 C 2 0 0 0 0 0 0 0 0 0 0 0

PDE6G -0.626190521480249 1.2159648725986e-07 1.51333132752617e-06 NA

SERPINA5 -0.626525027085495 5.5309037090684e-06 4.2160054168964e-05 C 403 4 0 1 0 0 0 3 0 0 0 1

MAB21L1 -0.626548211309722 5.94888056936809e-06 4.48226363600437e-05 NA

AGPAT9 -0.626596441570111 0.000488563623058731 0.00192446278743654 C 4 2 0 0 0 0 0 2 0 0 0 0

DENND1C -0.62704918420297 3.53266705669375e-06 2.86595753887881e-05 NA

LOC100130071 -0.627215680258479 4.48624572673707e-06 3.52182384363138e-05 NA

BEX5 -0.627775716614285 0.000238480859527626 0.00105337858302903 C 1 0 0 0 0 0 0 0 0 0 0 0

ZNF366 -0.627801676674313 2.21025898343458e-05 0.000140192934917783 C 1 0 0 0 0 0 0 0 0 0 0 0

LYNX1 -0.62802224835105 2.36919396955567e-05 0.000148233353220394 C 1 0 0 0 0 0 0 0 0 0 0 0

QPCT -0.628108094166124 0.00257059832823322 0.00766320909725472 NA

PDIK1L -0.628197115592201 4.45336429053952e-11 1.27132467461601e-09 C 175 2 0 0 0 0 0 0 2 0 0 0

WDR31 -0.628324680176323 7.21558835019091e-06 5.28315051079549e-05 NA

SV2B -0.628330752241443 0.00148408197970375 0.00484213405100032 C 3

CX3CL1 -0.62860006089504 1.73863622530659e-05 0.000114459714213488 C 80 7 3 1 0 2 0 2 1 0 0 0

CADM3 -0.62903030500944 1.33271392517085e-09 2.73879223254166e-08 C 8 1 0 0 0 0 0 1 0 0 0 0

LOC284933 -0.629402082522814 0.00232663646801158 0.00706166878710752 NA

ZNF157 -0.629624006594776 1.60195910972126e-09 3.23588901490867e-08 C 1

CPEB3 -0.629759395476174 1.37864599950367e-06 1.255708990982e-05 C 2

PPDPF -0.630009617724498 1.09887224992262e-08 1.78987565183179e-07 NA

C10orf91 -0.630071514866061 9.20378708679095e-06 6.52004793023716e-05 NA

WDR63 -0.631079296970775 9.78596249342819e-06 6.88546141684444e-05 NA

IL17RB -0.631110296248729 9.96440421311608e-05 0.000506570718618447 C 11 1 1 0 0 0 1 0 0 0 0 0

PCP4 -0.631264410588713 4.81245909225505e-06 3.73225543783551e-05 C 2 0 0 0 0 0 0 0 0 0 0 0

VSX1 -0.631610071615057 5.97526774174055e-07 6.07704957269873e-06 NA

KHK -0.631677502091941 5.81513153990491e-05 0.000318193103948483 C 1 0 0 0 0 0 0 0 0 0 0 0

LTB4R2 -0.631859210825312 0.000559630975467856 0.00215857661966173 C 14 5 2 0 0 0 0 3 0 0 0 0

MPZ -0.631951768776622 2.86668058816192e-07 3.23154636641696e-06 C 824 1 0 0 0 0 0 0 1 0 0 0

SMPDL3A -0.632244926822509 1.89694880525733e-05 0.000123422310199744 C 1

ICA1 -0.632969589609219 2.52008141320871e-07 2.87669536495796e-06 C 17 0 0 0 0 0 0 0 0 0 0 0

RNF208 -0.633012954947112 3.83615049702646e-07 4.14422491194177e-06 NA

DDAH2 -0.633102195650586 8.48866450171399e-09 1.43965011670198e-07 C 9 1 0 0 0 1 0 0 0 0 0 0

SSTR4 -0.633278783351875 2.49279090367352e-06 2.10629702829593e-05 C 593 2 1 0 0 0 1 0 0 0 0 0

HAL -0.633291732232576 0.00979202912683575 0.02354725458138 NA

TMIE -0.633700990776195 5.04245468525318e-05 0.000280866803495547 NA

PID1 -0.633842808049059 8.35410347728902e-05 0.000436167493457617 NA

GRID1 -0.634132568288996 2.82812572295426e-09 5.4123291767801e-08 C 5

TUB -0.634740427610609 1.15276988859075e-09 2.40556512473618e-08 NA

GPR6 -0.635001463479717 5.29873993137421e-07 5.47729386570833e-06 C 1 0 0 0 0 0 0 0 0 0 0 0

EMID1 -0.635406875623658 4.76845639771409e-09 8.61188685491742e-08 C 1 0 0 0 0 0 0 0 0 0 0 0

TPCN1 -0.635567658068672 8.62098599385953e-11 2.34102441758651e-09 C 23 2 0 0 0 0 0 1 1 0 0 0

LOC100289094 -0.635918282463969 1.51963603742063e-05 0.000101489595202047 NA

TMEM150C -0.636197437962557 4.58123724924406e-07 4.82521298922884e-06 NA

DLX5 -0.636288580340168 0.00415144870806612 0.0114406777185251 C 10 4 1 2 0 0 0 0 2 0 0 0

RAMP2 -0.636503710813262 9.68583160692716e-08 1.24118980923704e-06 C 11 0 0 0 0 0 0 0 0 0 0 0

FAM19A5 -0.636549668844769 0.000727877882437181 0.00269020595213601 C 1

C9orf129 -0.637259555418066 3.98667651523912e-06 3.18110566627027e-05 NA

PNOC -0.637309864866252 0.0024021493456523 0.00725461529547949 C 4 0 0 0 0 0 0 0 0 0 0 0

SYCN -0.637329512203232 3.26337358189458e-06 2.67500527434035e-05 C 1 0 0 0 0 0 0 0 0 0 0 0

TSGA10 -0.637728098858545 2.7352431059269e-06 2.28564884176064e-05 C 2 0 0 0 0 0 0 0 0 0 0 0

LOC100131496 -0.637752292162898 3.32326744066654e-06 2.71821447059186e-05 NA

BOC -0.638069097881855 1.96737003344433e-05 0.000126913471789369 NA

LPIN1 -0.63809761121867 6.2464148672946e-06 4.67480799498952e-05 C 1 0 0 0 0 0 0 0 0 0 0 0

NDNF -0.638218228132659 1.18537790349576e-05 8.13475256688333e-05 NA

PEBP4 -0.638223548264211 2.50240480304728e-06 2.11347682763391e-05 C 10 7 2 0 0 0 4 3 1 0 0 0

PSORS1C2 -0.638712898779426 0.000148325298076278 0.000707676560799021 C 4 1 1 0 0 0 0 0 0 0 0 0

MRAP2 -0.63899091408747 0.000396896240062053 0.00161410724874398 C 1

FGF23 -0.639084892097039 6.2746923160609e-06 4.69040685095121e-05 C 29 4 0 0 3 0 0 1 1 0 0 0

SYTL4 -0.639173107112077 0.000757199291824611 0.00278173738283471 C 2

GAB2 -0.63944905159887 4.33764134412446e-09 7.91692745614692e-08 C 52 16 5 1 0 1 1 6 6 0 0 0

B3GNT8 -0.639511570963152 3.80261995281263e-06 3.06134359195596e-05 C 2 0 0 0 0 0 0 0 0 0 0 0

ZNF788 -0.639678773680216 4.91809364963224e-06 3.80559110820071e-05 NA

FNDC4 -0.639842402185447 5.88882244365579e-06 4.44231735317151e-05 NA

MGC16025 -0.639925715589601 0.00555974875896389 0.0146681578980917 NA

ANK2 -0.639950445503761 1.09055485212562e-07 1.3742298060041e-06 C 2 0 0 0 0 0 0 0 0 0 0 0

PP2D1 -0.640034623323333 9.87427767078065e-08 1.26106918673998e-06 NA

CBFA2T3 -0.640207925420398 3.63655576760814e-08 5.18681921729611e-07 MN 2016 3 0 0 1 0 0 0 2 0 0 0

MLIP -0.640338108100911 0.0062594141949712 0.0161891134829489 NA

TMEM95 -0.640405610841907 1.85006591749228e-07 2.19399734463511e-06 NA

GNG13 -0.640579497458542 0.000222297770597797 0.000993255407012396 C 1 0 0 0 0 0 0 0 0 0 0 0

PARD6A -0.640908405970058 1.08374378559386e-08 1.77593234891212e-07 C 20 1 0 0 0 0 0 0 1 0 0 0

RNF112 -0.641003149803821 4.35120443873955e-06 3.43289897507392e-05 C 14 0 0 0 0 0 0 0 0 0 0 0

SLC1A1 -0.641080032216478 0.00115051153278674 0.00392179104167267 C 5 0 0 0 0 0 0 0 0 0 0 0

ANKFN1 -0.641407579302624 3.17741789686988e-06 2.61360228309675e-05 NA

NRG2 -0.641508859489764 7.10576279733864e-08 9.43794894492831e-07 C 10 0 0 0 0 0 0 0 0 0 0 0

GSTT2 -0.641798824130451 0.00010142936076296 0.000514127881939087 C 8 0 0 0 0 0 0 0 0 0 0 0

CES3 -0.641850994718745 7.65747607318935e-09 1.31161130893443e-07 C 1

MLC1 -0.642712296401936 0.000229738925487163 0.00101928472637026 NA

CHDH -0.642715923324413 4.50926725193283e-06 3.53696234054424e-05 NA

SEC14L5 -0.642975783665591 7.65393715739611e-09 1.31161130893443e-07 NA

OSBPL6 -0.642976851661389 5.82817686100904e-06 4.40886904269857e-05 C 2

MAT1A -0.643295638942866 0.000107177777512872 0.000538676848642658 NA

POU2AF1 -0.6434293702004 0.00777044771789866 0.019430739061523 C 21 1 0 0 0 0 0 0 1 0 0 0

OCM2 -0.643621354652845 7.89231322551749e-05 0.000414741853053639 C 63 0 0 0 0 0 0 0 0 0 0 0

DKK2 -0.644417838892227 3.74648726231577e-08 5.32755555325699e-07 C 16 2 0 0 0 0 0 2 0 0 0 0

RPS6KA6 -0.644507440465717 1.23396863384971e-08 1.97926477397232e-07 C 13 2 0 0 0 0 1 0 2 0 0 0

LOC349196 -0.644683403148524 0.000275739822219014 0.00118963474245254 NA

RALGPS2 -0.644920244268434 0.000159014481012449 0.000750540419481951 NA

ADSSL1 -0.645117011180077 8.8915711120364e-05 0.000459559711735426 NA

C9orf24 -0.645239132416898 5.58512644699791e-06 4.25049007890347e-05 C 1 0 0 0 0 0 0 0 0 0 0 0

PLAC4 -0.645777841189588 3.96019390503732e-06 3.16130704515569e-05 C 1

ZNF528 -0.64680256833761 0.000308039055209952 0.0013054758616119 NA

PLLP -0.646847606123454 2.41882215888849e-08 3.63341642867321e-07 MN 1

LOC283861 -0.646865761651551 0.000110216161446635 0.000550373769092665 NA

ZNF383 -0.647058503895686 4.06737948915539e-06 3.23187622129488e-05 NA

HILS1 -0.647075540044258 2.23796335551116e-06 1.92273864865001e-05 C 1 0 0 0 0 0 0 0 0 0 0 0

TYRO3 -0.647408840825229 6.60237227134335e-08 8.84381457747456e-07 C 194 9 3 0 2 0 2 3 4 0 0 0

RTDR1 -0.647509264631073 7.61415736185394e-06 5.52369323065579e-05 C 1 0 0 0 0 0 0 0 0 0 0 0

OR10H4 -0.647737964071053 6.02827728685336e-05 0.000329000012134584 NA

AOC3 -0.647799691406325 3.63825914083838e-07 3.9666665183553e-06 C 20 1 0 0 0 0 0 1 0 0 0 0

FGFBP3 -0.647918391106733 1.35559531698702e-07 1.66822838521543e-06 C 1

GDF7 -0.648225445927317 3.12209712608765e-06 2.57369043142252e-05 C 1

AOX1 -0.648252885388955 0.00196512663440895 0.00614674463881313 C 11 1 0 0 0 0 0 1 0 0 0 0

MYEF2 -0.648489882731847 4.73590534042796e-08 6.56196781685798e-07 C 1 0 0 0 0 0 0 0 0 0 0 0

MSMB -0.648501208699757 0.000650317818344594 0.00244703088425609 C 523 9 2 1 1 1 2 4 0 0 0 0

ZNF879 -0.648623234578428 1.99168400068218e-06 1.73961435689334e-05 NA

GPRC5A -0.648814718711199 0.00368540686188645 0.0103492130081491 C 49 6 1 0 1 0 1 0 0 0 0 3

LOC202181 -0.648907801898864 2.01913833403304e-06 1.75949499301304e-05 NA

UGT1A8 -0.649203428219441 0.00436109900314663 0.0119194975931489 C 10 0 0 0 0 0 0 0 0 0 0 0

LOC100130691 -0.649291735902981 1.43903890213006e-06 1.30443914274979e-05 NA

ISOC1 -0.649392288751549 2.65457331885215e-09 5.11640623278152e-08 C 1 0 0 0 0 0 0 0 0 0 0 0

ZNF365 -0.649959859752456 0.00370130809717215 0.0103804312131845 C 1 0 0 0 0 0 0 0 0 0 0 0

TACSTD2 -0.650295633976985 3.64412277406848e-06 2.9487948586915e-05 MN 1 1 0 1 1 0 0 0 0 0 0 0

LPAR4 -0.650435677650509 2.42709317979615e-07 2.78072594515748e-06 C 10 0 0 0 0 0 0 0 0 0 0 0

ZNF350 -0.650754011335133 0.000767662524310013 0.00281198927764963 C 6 1 0 0 0 0 0 1 0 0 0 0

NRXN3 -0.6508564950445 3.95618551161359e-05 0.000228010728313978 C 1 0 0 0 0 0 0 0 0 0 0 0

KCNA7 -0.651191309045635 1.97752404326033e-05 0.000127438193962507 NA

TESK2 -0.651261322377513 2.21091116805971e-10 5.42748582073491e-09 NA

PIM1 -0.651457016113079 6.26314471480884e-06 4.68547589000739e-05 C 228 23 16 0 1 0 8 0 3 0 0 0

TMEM98 -0.65155303632028 1.17562819971993e-05 8.07956243140856e-05 C 1 0 0 0 0 0 0 0 0 0 0 0

MRGPRX2 -0.651593368161605 2.39627668122864e-06 2.03931334287834e-05 C 1 0 0 0 0 0 0 0 0 0 0 0

LLGL2 -0.652070640342936 4.97620237647319e-09 8.93591863183189e-08 C 10 3 0 0 2 0 0 1 0 0 0 0

TMEM132B -0.652751335937612 3.22918519725592e-09 6.11799681966594e-08 NA

LINC00302 -0.653161974653116 0.0038582164042173 0.0107499575861359 NA

LOC729911 -0.653368634011216 0.000207272800303864 0.000937632000800963 NA

LOC100132319 -0.653435790347535 4.65547125777155e-08 6.46471786469861e-07 NA

CD34 -0.653774766209489 2.93271999082303e-07 3.29421906624971e-06 MN 91 7 0 0 3 1 1 3 1 0 0 1

CDH22 -0.654003216489025 1.94620658205934e-05 0.00012580550539152 C 6 0 0 0 0 0 0 0 0 0 0 0

CHRNA9 -0.654057171271474 0.000588602986231907 0.00224787907998614 C 1 0 0 0 0 0 0 0 0 0 0 0

ZSCAN16 -0.654244617640341 0.000134852169171696 0.000652607263081741 NA

MEGF6 -0.654294863029676 4.0420173749815e-06 3.21847971629259e-05 C 1 0 0 0 0 0 0 0 0 0 0 0

MAGI1 -0.654567431762285 1.07154942284261e-06 1.01002071345329e-05 C 32 5 1 1 0 0 1 0 2 0 0 0

FAM84A -0.654752934503785 0.00520682691155827 0.0138821824137292 C 3 2 0 0 0 0 0 2 0 0 0 0

HTR1B -0.655171190873776 7.23007244147172e-06 5.2876190533128e-05 C 16 2 1 0 1 0 1 0 0 0 0 0

RAB3C -0.656445219695903 6.46599091998308e-09 1.12690432912081e-07 C 2

TST -0.657162458816801 6.57835485254725e-07 6.58426876225075e-06 C 36 1 0 0 0 0 0 0 0 0 0 1

IL17RD -0.657459488561067 1.26429368854284e-08 2.01934908380171e-07 C 27 3 0 0 0 0 0 1 2 0 0 0

HBD -0.657461232733521 0.0001857737401241 0.000856133328300179 MN 6 1 0 0 0 0 0 0 1 0 0 0

LOC100507557 -0.657702338556014 9.27887496216554e-07 8.95569945991367e-06 NA

ZG16B -0.658955592698118 0.00616770194170177 0.0159911680139834 C 11 4 0 0 0 0 0 3 1 0 0 0

GAL3ST1 -0.659052545449273 5.9174713037725e-07 6.02798597236287e-06 C 29 1 0 0 0 0 0 0 1 0 0 0

IL36B -0.65905658162112 0.000756822571681089 0.00278089318854746 C 1 0 0 0 0 0 0 0 0 0 0 0

GALR2 -0.659199288941044 9.90290187711593e-08 1.26302037620063e-06 C 9 4 0 1 0 0 2 0 3 0 0 0

DPEP1 -0.659213855906014 3.46148774940641e-07 3.7958040922952e-06 C 505 2 0 0 0 0 1 2 1 0 0 0

SHANK1 -0.6599674489362 2.78509604701128e-06 2.31809643279607e-05 C 1

TSPAN12 -0.660325974543321 0.000120843016389121 0.000595691439987016 C 6 1 0 0 0 0 0 1 0 0 0 0

FAM102A -0.660462755582547 2.57847611104179e-08 3.83919756976802e-07 C 1

EFNA2 -0.660797757657039 4.19298028469537e-09 7.69743335096307e-08 C 21 4 0 2 0 2 0 2 1 0 0 0

PRRT1 -0.661742654990527 4.35366266441239e-07 4.62671382646453e-06 C 1 0 0 0 0 0 0 0 0 0 0 0

KANK3 -0.661811343933925 1.18834604767985e-08 1.91582841945796e-07 NA

GRAMD1C -0.662068820202049 1.51073005961583e-06 1.36159942087375e-05 NA

TF -0.662545794740592 1.88524671436059e-05 0.000122914449062015 C 1875 105 26 1 4 2 8 15 59 0 0 0

ABCA7 -0.662579531815649 2.21574070426072e-06 1.90451064076034e-05 C 2 1 1 0 0 0 0 0 0 0 0 0

MC2R -0.662757899868303 1.53211019457598e-06 1.37693493127919e-05 C 16 0 0 0 0 0 0 0 0 0 0 0

TTLL3 -0.663271934985553 0.000154952422297912 0.000734141869664576 NA

SPRY2 -0.663380819939427 1.87569321354216e-06 1.64968612698478e-05 C 43 11 6 3 0 1 1 1 5 0 0 0

PTGDR2 -0.66368942943762 2.06700840560689e-07 2.4164464541644e-06 C 12 0 0 0 0 0 0 0 0 0 0 0

LRRC4C -0.664453017138891 2.24144426345122e-08 3.38848367207198e-07 C 3

KIF26A -0.664568266803105 7.2275035361143e-07 7.16579148392014e-06 NA

ARMCX6 -0.664633181484109 2.06207008631653e-05 0.000132121870425569 NA

CEL -0.665625713089251 3.00391116245968e-07 3.36741410200827e-06 MN 6852 0 0 0 0 0 0 0 0 0 0 0

SYT8 -0.665673518070121 0.00899614220419075 0.0219335287258429 NA

CYP27A1 -0.665781392678457 1.70534224612707e-05 0.000112581139492316 NA

RNF150 -0.666369961635134 0.000735335886026491 0.00271353135403069 NA

SLC9A2 -0.666404362345862 2.16874572480788e-05 0.000137975967507357 C 1 0 0 0 0 0 0 0 0 0 0 0

BZRAP1 -0.667557332529315 5.73183114041514e-09 1.0155537427485e-07 NA

NCAM2 -0.667661821463385 2.69447110747514e-08 3.98112838807041e-07 C 3 1 1 0 0 0 0 0 0 0 0 0

KRT20 -0.667856066734554 1.33565756839671e-06 1.22184585775953e-05 MN 36 3 0 1 2 0 0 0 0 0 0 0

ZNF682 -0.669824908917493 2.09365387702538e-07 2.44457661508077e-06 NA

KREMEN1 -0.670226682555064 6.84393546587742e-09 1.18513418630066e-07 C 2 0 0 0 0 0 0 0 0 0 0 0

AK7 -0.670234746325125 3.22756065804664e-05 0.000192221650644584 C 1 0 0 0 0 0 0 0 0 0 0 0

POU3F1 -0.671081788335921 9.18365632028656e-06 6.51007727243684e-05 C 6 0 0 0 0 0 0 0 0 0 0 0

MAPK13 -0.67156675311726 1.53188118939236e-06 1.37693493127919e-05 C 15 4 3 0 1 0 0 2 3 0 0 0

SCN4B -0.671654990668381 0.00123936683254949 0.00416429895964215 C 1 0 0 0 0 0 0 0 0 0 0 0

CACNA2D3 -0.672191312769258 9.33097899929577e-05 0.000478610947207781 C 5 2 1 1 0 0 1 1 1 0 0 0

ACSBG1 -0.672230036878914 4.20379145392161e-07 4.4876007246686e-06 C 123 2 0 0 1 0 0 1 0 0 0 0

THEM5 -0.672389149614292 9.87136328248966e-08 1.26106918673998e-06 NA

RNLS -0.672427322032743 0.00493877376866927 0.0132606434939689 C 1 0 0 0 0 0 0 0 0 0 0 0

LOC100127983 -0.672771296920613 0.000122480614693433 0.000601658602206748 NA

NBEA -0.673056231290644 3.21525579365739e-05 0.000191835917784646 C 4

SGCZ -0.673112353305208 6.55557598223078e-05 0.000353396145302426 NA

GAL3ST4 -0.673419648596352 1.03003501163313e-08 1.69821190463243e-07 NA

LTC4S -0.673934642056965 1.27416986933433e-08 2.03340751407174e-07 C 4 0 0 0 0 0 0 0 0 0 0 0

ANO8 -0.674333757656542 2.38506678447865e-05 0.000148885748779114 NA

AGT -0.674449564845758 7.81456960976496e-05 0.000411422417257362 C 436 35 15 1 1 5 1 15 6 0 0 3

RTKN2 -0.674477180592996 0.0070875516699292 0.0179868718767431 C 1

DBI -0.674878086686729 2.89686287592745e-10 6.98457626148775e-09 C 20 2 0 0 1 0 0 0 1 0 0 0

FAM155A -0.675509884711136 1.52133466561483e-05 0.000101567200056762 NA

CDCP2 -0.67605490339509 6.43183493484585e-05 0.000347815256605221 NA

DUSP13 -0.676667555800995 0.000430575592758024 0.0017331995414996 C 1 0 0 0 0 0 0 0 0 0 0 0

BAI3 -0.676918140471703 3.36118298276718e-06 2.7432992805017e-05 C 4 0 0 0 0 0 0 0 0 0 0 0

ZNF823 -0.67693051040009 3.20450124089622e-08 4.60878381355948e-07 NA

ZBTB7B -0.677313787396111 7.16729592941312e-13 3.23131918017812e-11 C 1

KIR3DL1 -0.677732017984656 3.66927485041598e-07 3.99696205784588e-06 C 99 0 0 0 0 0 0 0 0 0 0 0

BCL11A -0.678006584244206 2.92249081735212e-07 3.28663004753556e-06 C 17 1 0 1 1 0 0 0 0 0 0 0

TP53 -0.678444890303695 3.44356053251455e-05 0.000202851759100227 MN 584 139 18 38 18 4 40 22 32 0 2 1

S1PR1 -0.678588926759264 6.78045259492532e-06 5.01107482483996e-05 C 64 2 1 0 1 0 0 1 0 0 0 0

CYP17A1 -0.678624769996405 2.25506921306703e-07 2.61369840757622e-06 C 100 3 2 0 0 0 0 2 0 0 0 0

ZNF93 -0.678825034131167 5.63213077010378e-06 4.28109795525117e-05 C 1

TTC29 -0.679060260079579 1.40256195310673e-08 2.2158839805051e-07 NA

AMPD1 -0.679631533125186 0.00498026461918086 0.0133571586293377 C 115 6 2 0 0 0 1 0 6 0 0 0

NKX2-3 -0.679698142453636 1.89696748551988e-12 7.83928026166699e-11 C 3 0 0 0 0 0 0 0 0 0 0 0

MMAB -0.680626003383096 1.02368974546576e-07 1.29861768179829e-06 C 223 13 2 0 0 0 10 0 2 0 0 0

NR0B1 -0.681962301382667 9.94464784566763e-08 1.26749057087509e-06 C 911 8 3 5 0 0 0 1 0 0 0 0

NIPAL2 -0.681979198282566 2.20247093839539e-08 3.3426156077281e-07 NA

KRT10 -0.682082264952045 0.00364873500703272 0.0102605690611206 C 141 4 0 0 2 0 0 2 1 0 0 0

EPB41L1 -0.682096653809511 8.2389552530721e-06 5.90900742989373e-05 C 2 0 0 0 0 0 0 0 0 0 0 0

LOC388242 -0.683359967557192 7.14555395370207e-10 1.58615608149884e-08 NA

DBP -0.683389314998022 5.43512961391842e-11 1.53309535324343e-09 C 46 1 0 0 1 0 0 0 0 0 0 0

KRTAP9-4 -0.683467977482582 0.0025090452755389 0.00751284605760555 NA

MTRNR2L2 -0.683725835165911 3.27330201680847e-05 0.000194212499285686 C 19 0 0 0 0 0 0 0 0 0 0 0

SPINK2 -0.685292806498052 0.00270261277847626 0.00797935021588557 C 2 0 0 0 0 0 0 0 0 0 0 0

PARD3B -0.685432618853793 6.5285884788084e-09 1.13676719538553e-07 C 1

FAM74A3 -0.685824583608892 4.94773189183278e-06 3.82383509664023e-05 NA

COX7A1 -0.686571079966684 4.16080644184769e-08 5.85078629456547e-07 C 1 0 0 0 0 0 0 0 0 0 0 0

SMR3A -0.686787736223391 0.00298935072555807 0.00867915956168701 C 25 1 1 0 0 0 0 0 0 0 0 0

CYP11A1 -0.68696660800201 1.10162832172522e-11 3.75685031446723e-10 C 45 2 0 0 0 0 0 1 1 0 0 0

TLR5 -0.687354511101612 0.000985958481783182 0.0034551446370506 MN 3 1 0 0 0 0 0 1 0 0 0 0

TMEM40 -0.687882773760547 8.13688974407529e-05 0.000425786320669375 NA

PLEKHA6 -0.688079723627387 2.87883221729308e-05 0.00017430472609311 C 2

SERTAD4 -0.688278809935323 3.71222238953244e-07 4.03105181679177e-06 NA

C7orf57 -0.68831125536691 7.73481094358336e-09 1.32127045784479e-07 NA

ANKS1B -0.689129686576909 4.631534744742e-13 2.18062333616248e-11 C 41 4 1 1 0 0 0 3 2 0 0 0

AADAT -0.689525303190475 1.62291980701983e-05 0.0001078546460234 C 1 0 0 0 0 0 0 0 0 0 0 0

DCN -0.690058013985393 0.00777015390071965 0.019430739061523 MN 5 2 0 0 0 0 0 2 0 0 0 0

DIO3 -0.690528342002875 0.000988930302376739 0.0034649174070871 C 8 0 0 0 0 0 0 0 0 0 0 0

CD79A -0.691065898296051 0.000282098060014824 0.0012129191235576 C 799 54 10 5 18 0 1 11 12 0 0 5

CMTM2 -0.691590929768747 1.36172917692265e-07 1.67251447966353e-06 C 1

ZNF395 -0.691624147870407 1.69265404478723e-12 7.02562787405435e-11 C 23 0 0 0 0 0 0 0 0 0 0 0

INPP5J -0.691646466084825 4.68117087966011e-10 1.07758290826236e-08 C 7

AQP5 -0.691651914654085 0.00060096768152421 0.00228909545345315 MN 1 1 0 0 0 0 0 0 1 0 0 0

MPPED2 -0.691883303232082 1.51911448005024e-08 2.37425927034773e-07 C 2 2 1 0 0 0 0 1 0 0 0 0

GSTO2 -0.692770312979741 0.0149397665519055 0.0335148703956282 C 4 1 0 0 0 0 0 1 0 0 0 0

DUOX1 -0.693412304406814 1.43759400421305e-05 9.65555064504629e-05 C 19 1 0 0 0 0 0 1 1 0 0 0

KIR3DL2 -0.694081944774005 3.89967150397429e-06 3.12221161403221e-05 C 31 3 1 0 1 0 0 0 1 0 0 0

CYP2F1 -0.694083082077394 0.000181641939067447 0.000839955284810549 C 4 0 0 0 0 0 0 0 0 0 0 0

FRMPD1 -0.69444489555354 1.51438031029884e-06 1.36423970171471e-05 C 1

ZNF285 -0.694717646079836 6.04190608674518e-05 0.000329553765140709 NA

USP54 -0.694756108521066 1.1033618269592e-09 2.31266105192212e-08 NA

SEMA4G -0.695300793510152 1.06862768701664e-05 7.42507938038327e-05 C 1

ADAMTS17 -0.695403603584355 9.33318579158765e-06 6.59631095882671e-05 C 1 0 0 0 0 0 0 0 0 0 0 0

KCNN2 -0.695464094476326 1.44399477684976e-06 1.30767890628877e-05 C 19 0 0 0 0 0 0 0 0 0 0 0

MCEE -0.695722849243797 8.81483058217323e-12 3.07252851618403e-10 NA

SDCBP2 -0.695761958455249 0.000364199284589753 0.00150047885490428 C 11 0 0 0 0 0 0 0 0 0 0 0

JSRP1 -0.695886280909895 0.000725314629249267 0.00268230363184855 NA

ZNF71 -0.696243431102817 4.76300811271503e-05 0.000267426444821588 C 2

IL36RN -0.696410180273475 0.0114771535984126 0.0269179784581357 NA

CMA1 -0.696581552663138 0.000492032635694438 0.00193611261866708 C 118 10 4 2 1 2 0 3 1 0 0 0

SALL2 -0.696668095300671 1.55391774500417e-07 1.88049879537685e-06 C 6 0 0 0 0 0 0 0 0 0 0 0

CNTFR -0.697136256042127 5.74188526918079e-07 5.87759126499648e-06 C 8 1 1 0 0 0 0 0 0 0 0 0

PLXDC2 -0.697359942619472 3.19524986902937e-05 0.000191052453792986 C 2 0 0 0 0 0 0 0 0 0 0 0

MYZAP -0.697573306101267 0.000822006152571235 0.00297137327152707 NA

LOC100132330 -0.697629496882031 0.000271903380794639 0.00117818573450094 NA

ANKRD37 -0.697844287405342 1.04401421144818e-06 9.88497097552763e-06 NA

KAT2B -0.698028379693512 5.39554657461811e-06 4.1227900693499e-05 C 359 5 2 0 0 0 1 1 2 0 0 0

PCDH1 -0.698058860175832 4.41052437419026e-09 8.03445571032714e-08 C 4 0 0 0 0 0 0 0 0 0 0 0

ELANE -0.698198833116872 8.00533936324264e-06 5.76548927428057e-05 C 306 15 0 1 1 0 5 7 2 0 0 0

SCARA5 -0.698492619985595 6.69440388975492e-07 6.68980899796153e-06 C 3 0 0 0 0 0 0 0 0 0 0 0

MID2 -0.699288481887926 3.24141478162611e-05 0.000192682969760796 C 1 0 0 0 0 0 0 0 0 0 0 0

ECSCR -0.699689045544766 0.000165699330474298 0.000776900699323913 C 5 0 0 0 0 0 0 0 0 0 0 0

CILP2 -0.70023722897927 1.34500714453323e-08 2.13744334379349e-07 C 2 0 0 0 0 0 0 0 0 0 0 0

ZNF527 -0.700240365981505 1.2573133369873e-10 3.29600685999426e-09 NA

SOX10 -0.700372899499034 1.6597981037227e-07 1.98577741524396e-06 MN 2 1 1 0 1 0 0 0 0 0 0 0

BAMBI -0.700838138858308 0.00155574737122768 0.00503521172001437 C 52 2 0 1 0 0 0 1 1 0 0 0

SLC3A1 -0.701612729248059 3.39236995797505e-09 6.36346741274468e-08 C 53 0 0 0 0 0 0 0 0 0 0 0

DIO2 -0.701764752606857 0.00029226470415137 0.00124897133788055 C 8 2 0 0 0 0 0 2 0 0 0 0

ASAH1 -0.702061188193999 1.37176971739289e-08 2.17449626809843e-07 C 63 9 4 0 0 0 3 2 2 0 0 0

KRT27 -0.702308278283931 0.00059765476126634 0.00227876947350685 C 9 0 0 0 0 0 0 0 0 0 0 0

FAM47B -0.702381914400943 1.42351220356537e-08 2.24523462307348e-07 NA

RGMA -0.702384264891568 1.23977042648025e-07 1.53935891395837e-06 NA

HCG9 -0.702432687812394 4.42044650453479e-06 3.47593647658205e-05 C 6

MGC57346 -0.702652736215555 5.13513135462778e-10 1.16677828510252e-08 NA

CUBN -0.703248481696196 7.93966537835949e-06 5.72447947446523e-05 C 3 0 0 0 0 0 0 0 0 0 0 0

PKNOX2 -0.703599363349658 1.4060733939814e-05 9.47072993874948e-05 NA

CBLN2 -0.70584444763672 0.000156121705761363 0.000738728881236328 NA

CRISP2 -0.70773409809053 8.29622012165964e-05 0.000433524456771541 C 78 0 0 0 0 0 0 0 0 0 0 0

KRT31 -0.708125909520942 7.33419795806032e-07 7.26016552051295e-06 C 37 10 4 0 1 0 0 4 1 0 0 0

POMC -0.708234172593736 4.10221911240163e-07 4.39652894339896e-06 C 1423 116 24 6 17 3 1 51 24 0 0 2

CADM1 -0.708858780455248 0.000332800122966885 0.00139356370075094 C 492 23 2 5 5 0 5 9 4 0 0 0

MTUS1 -0.709689408496 8.77035840833947e-09 1.47907034045964e-07 MN 1 1 1 0 0 0 0 0 0 0 0 0

TMC4 -0.709945026811862 9.16014147341519e-05 0.000470997005344551 C 1

LOC100506388 -0.71008501582899 6.33497168006452e-07 6.38138125180939e-06 NA

SLC9A9 -0.710244680010814 1.22579727287836e-07 1.523352920799e-06 C 1 0 0 0 0 0 0 0 0 0 0 0

ADAM33 -0.710958365764033 4.51898060978877e-10 1.04560814182729e-08 C 3 1 0 0 0 0 0 1 1 0 0 0

RSPH1 -0.711234072994176 6.63148402458888e-05 0.000356878300066516 C 1

PI16 -0.711737363292611 2.84879863268893e-05 0.000172817986284947 C 1 0 0 0 0 0 0 0 0 0 0 0

ZDHHC13 -0.711858019316645 1.43760403226245e-06 1.30376289020754e-05 NA

ZNF568 -0.71203857632024 4.61652760487866e-12 1.7065823823738e-10 NA

LOC100509814 -0.712228254409445 2.52971622922128e-05 0.000156266119681695 NA

VENTXP1 -0.712657289350608 2.08765235623482e-08 3.18140065591436e-07 NA

LCN6 -0.712905626417594 1.34681865332675e-09 2.76477620949191e-08 NA

MGC2889 -0.713879047379788 3.07517822417939e-11 9.28291838102765e-10 NA

KCNIP3 -0.713892965430936 5.60477644924789e-07 5.7559199053128e-06 C 41 2 1 0 0 0 2 0 0 0 0 0

KCTD11 -0.714187344452164 2.51778594157781e-05 0.000155732465739357 NA

MAP3K9 -0.714544857414091 2.94041263760115e-06 2.43665455305941e-05 C 7 0 0 0 0 0 0 0 0 0 0 0

FXYD1 -0.715103751736273 8.41450722986281e-07 8.21358320472478e-06 NA

EPS8 -0.71561590032344 0.000199939211841424 0.000910112905849598 MN 2 1 1 0 0 0 0 1 0 0 0 0

ZNF790 -0.715770148141477 8.39365557733103e-05 0.000437890625998193 NA

CACNA2D1 -0.716052509436163 0.000539588631870547 0.00209149990485641 C 2 0 0 0 0 0 0 0 0 0 0 0

C1QTNF7 -0.716743724695764 5.1358174074771e-08 7.03369146681035e-07 NA

DSCAML1 -0.718003634355809 1.07102533848298e-06 1.01002071345329e-05 C 1

TMEM47 -0.718064523260882 3.47400310326724e-06 2.82441824465374e-05 C 2 0 0 0 0 0 0 0 0 0 0 0

CCDC3 -0.718687976780083 0.000661718954985928 0.00248351272278776 NA

C11orf92 -0.718865221388553 0.000396627600082614 0.00161336139840181 C 1 0 0 0 0 0 0 0 0 0 0 0

TMEM74 -0.719618998719605 7.83105995973175e-13 3.48749345547866e-11 NA

KGFLP2 -0.719929414570988 0.00027240163907283 0.00117935015981772 NA

CYB5A -0.72025557535486 5.44078969097389e-05 0.000300041820277952 C 11 1 0 1 0 0 0 0 0 0 0 0

CRHBP -0.721013744561168 2.07401057224103e-09 4.10185978064848e-08 C 3

PDZD2 -0.721032631054655 3.00677148820176e-07 3.36741410200827e-06 MN 5 4 0 0 1 0 0 3 0 0 0 0

QRICH2 -0.721246066247267 2.65852222879489e-12 1.04393880133612e-10 NA

FOXO4 -0.721530137510597 1.35209578211399e-10 3.52385056573478e-09 C 76 3 1 0 0 0 1 1 1 0 0 0

PRM1 -0.721603543223138 8.67455775967262e-09 1.4672328392969e-07 C 1 0 0 0 0 0 0 0 0 0 0 0

OLFML1 -0.721918274532496 0.000106363064164617 0.000535550336643708 NA

TNFRSF17 -0.723379736669618 0.0173679904356157 0.0379369826860818 C 74 4 1 0 0 0 0 0 4 0 0 0

KCNG3 -0.723684131457421 2.75358791202597e-06 2.29692192203242e-05 C 4 0 0 0 0 0 0 0 0 0 0 0

C2orf40 -0.723997980471025 2.41412849924743e-07 2.76754755331654e-06 C 10 2 0 2 0 0 0 0 1 0 0 0

LOC728485 -0.724516364187132 3.95655854095007e-06 3.15973770061443e-05 NA

RAI2 -0.724795790633963 5.95009300504322e-07 6.05795644467203e-06 NA

CTNNBIP1 -0.724957784758989 9.87277355436241e-10 2.10166928640235e-08 C 30 1 0 0 0 0 1 0 0 0 0 0

CRYM -0.725433960293886 8.6961246689565e-06 6.20865905731195e-05 C 4 1 1 0 0 0 1 0 1 0 0 0

ISL1 -0.725620775779399 2.16903477383737e-10 5.33160015122336e-09 C 20 2 0 0 0 0 0 2 0 0 0 0

LOC100131860 -0.725968643625544 4.97899726441461e-08 6.84368055363656e-07 NA

AGFG2 -0.726844969193852 1.92191998202561e-17 2.71464026117901e-15 C 26 0 0 0 0 0 0 0 0 0 0 0

HS3ST4 -0.727414955120892 1.37412829986435e-10 3.56275703171678e-09 C 1 0 0 0 0 0 0 0 0 0 0 0

PLEKHG6 -0.728077407915724 1.07083994971323e-07 1.35208724004151e-06 C 4 1 0 0 0 0 0 1 0 0 0 0

SH2D4A -0.72820389528595 5.21499278063498e-07 5.40760749229186e-06 C 1

LPHN2 -0.728478718875226 5.11282240328799e-06 3.93056009857968e-05 C 40 0 0 0 0 0 0 0 0 0 0 0

CYP7B1 -0.729513360163347 0.000591021568221696 0.00225575019595322 C 3 1 0 0 0 0 0 0 1 0 0 0

PLD1 -0.729917852977518 3.35746914789124e-07 3.69243570959544e-06 C 41 7 1 0 0 0 0 6 2 0 0 0

FAM180A -0.730012204380351 4.68563815448788e-10 1.07758290826236e-08 NA

ALS2CL -0.730766857237278 1.16017408432535e-06 1.08330611218677e-05 C 1

TP53INP2 -0.731443373046018 1.72950460809044e-05 0.000113937813147678 C 58 1 0 0 0 0 0 1 0 0 0 0

ZBTB7C -0.731618418707828 4.03395245945768e-09 7.45611505860893e-08 C 2 1 0 0 0 0 0 0 1 0 0 0

ZNF554 -0.732470944585814 1.96865665093202e-11 6.31538380206616e-10 NA

GNG7 -0.732654028814577 3.17054851847431e-06 2.61135647559457e-05 C 6 2 0 0 0 0 0 1 0 1 0 0

ZNF585B -0.73340688867318 1.66340040855516e-06 1.47877780801895e-05 NA

TEC -0.733460056195217 9.9851323911066e-10 2.12108418368658e-08 C 94 2 0 0 0 0 0 0 2 0 0 0

ZBED3 -0.733691216375678 2.11564604876983e-09 4.16678800885188e-08 NA

MYB -0.733921725362545 4.13243466844001e-07 4.42140141150729e-06 MN 12 2 0 0 0 0 0 1 1 0 0 0

REEP6 -0.733994206911068 4.05528708770695e-11 1.17361496496987e-09 C 1 0 0 0 0 0 0 0 0 0 0 0

APPL1 -0.734356133714976 6.47757914733348e-11 1.80031043350339e-09 C 18 1 0 0 0 0 0 0 1 0 0 0

XKR6 -0.734904204825154 6.85983439888256e-08 9.15628248714036e-07 NA

TUBB2B -0.735954201064604 1.31542078533395e-07 1.62195239114109e-06 C 3

ICOSLG -0.736280256498103 7.34290894763118e-13 3.29334686378709e-11 C 27 3 1 0 0 0 0 1 1 0 0 0

DGAT2 -0.736447767037513 0.00408607075966245 0.0112851395400746 C 2 0 0 0 0 0 0 0 0 0 0 0

FAM92B -0.736605206142911 0.000207845451132949 0.00093977325695015 NA

ZNF649 -0.736734811843034 2.40189945399331e-05 0.00014978830631213 NA

KATNAL2 -0.737109442245788 2.4957558444815e-09 4.82997657142141e-08 C 2

GFOD2 -0.737977190742275 2.66288195126588e-11 8.24883251908498e-10 NA

DSC2 -0.737999505360438 0.000928208025708394 0.00328561685105344 MN 3 1 0 0 0 0 0 1 1 0 0 0

IRX5 -0.740188513304589 9.79767758029029e-10 2.08829553560985e-08 C 3 1 0 0 0 0 1 0 0 0 0 0

SNCAIP -0.740202396024233 8.64306481346886e-06 6.17310519715189e-05 C 3 1 0 0 0 0 1 0 0 0 0 0

MALL -0.740407628202313 2.66255007345951e-05 0.000163351978088714 C 11 1 0 1 0 0 0 0 0 0 0 0

S100A16 -0.740628368678288 7.91612339110412e-08 1.04119852274793e-06 C 4 0 0 0 0 0 0 0 0 0 0 0

MGC21881 -0.74089915752157 2.28609498945095e-10 5.60478236597645e-09 NA

GLUL -0.741400770379723 0.000321088849220789 0.00135380901073778 C 115 4 0 0 0 0 1 1 2 0 0 0

PPARGC1A -0.742411334172802 4.80573943340612e-08 6.63928688000566e-07 C 10 0 0 0 0 0 0 0 0 0 0 0

BDNF -0.743163984402652 1.97468602440331e-08 3.01897272890804e-07 MN 4 1 0 0 0 0 0 1 0 0 0 0

OTOP2 -0.743954123965167 2.67528887844021e-09 5.15108775200792e-08 NA

SLC35G3 -0.745029427437747 9.11863475931853e-09 1.53003900788672e-07 NA

DACT3 -0.745066855497764 4.42886569604398e-08 6.19550192380077e-07 C 4 1 1 0 0 0 1 0 0 0 0 0

SLC44A2 -0.74617534782552 9.76575775607419e-07 9.3588099771755e-06 C 2 0 0 0 0 0 0 0 0 0 0 0

CTRB2 -0.74621259519929 5.85923235047414e-07 5.98153671507141e-06 NA

DDX11L2 -0.746304928075706 3.15896820217427e-12 1.21771468762836e-10 C 4 1 0 0 0 0 0 0 0 1 0 0

CLEC12B -0.746679666147495 1.8567255105352e-06 1.63452296455348e-05 NA

ITPR2 -0.749822476831104 1.10853330590276e-06 1.04176811722054e-05 C 16 3 0 0 0 0 2 0 1 0 0 0

NPDC1 -0.749841659262437 9.30986604937909e-10 2.00463975786801e-08 C 74 1 0 0 0 0 0 0 1 0 0 0

THSD7A -0.750537427160465 1.18709413727331e-07 1.48256064868261e-06 C 1

NEIL1 -0.751296826207855 1.34902377078069e-08 2.14202792865488e-07 C 4 0 0 0 0 0 0 0 0 0 0 0

EXTL1 -0.751588671991641 1.06539205533903e-08 1.74925294330028e-07 C 1 0 0 0 0 0 0 0 0 0 0 0

FAM150B -0.752170202346813 2.75059792580444e-08 4.04763475546557e-07 NA

RECQL5 -0.752359897490773 4.57393081262424e-12 1.69414458885595e-10 C 1 0 0 0 0 0 0 0 0 0 0 0

C1orf177 -0.752360565142734 4.77613931775935e-07 5.00265572037804e-06 NA

CYP26A1 -0.753455457346176 0.000174781940523071 0.000812800439380876 C 30 3 0 1 0 0 1 1 1 0 0 0

ASPG -0.753624453587528 2.84634913358987e-05 0.000172724751687898 C 1 0 0 0 0 0 0 0 0 0 0 0

MYO15B -0.75468452015293 1.81618113133999e-10 4.55296162554595e-09 NA

TLE2 -0.755187410324786 1.64772547755223e-12 6.88443711117682e-11 C 8 0 0 0 0 0 0 0 0 0 0 0

LOC100128252 -0.755435688332188 1.522513450791e-07 1.84485352644823e-06 NA

ME1 -0.755775456332209 0.0013782464447204 0.00455016055454788 NA

ARHGEF26 -0.755933243039925 3.47887499007316e-05 0.000204614195536263 C 2 1 0 0 0 0 0 1 0 0 0 0

ELN -0.756491952915278 1.58631175528238e-10 4.05731386381482e-09 C 321 11 1 1 3 1 0 6 1 0 0 1

CCDC6 -0.756650808411875 4.05468253166831e-14 2.45971077810532e-12 C 1396 5 0 0 0 1 0 2 3 0 0 0

EPHA7 -0.756742092596214 8.63269271267178e-09 1.46145773678657e-07 MN 1 1 0 1 0 0 0 0 0 0 0 0

STXBP5 -0.757221401331433 5.3272683966787e-09 9.54822054393349e-08 C 1 0 0 0 0 0 0 0 0 0 0 0

KIF21A -0.75731432942681 7.27939197218814e-09 1.25251865325095e-07 C 1 0 0 0 0 0 0 0 0 0 0 0

DUOXA1 -0.757364546174486 7.76806322623559e-06 5.61811741241731e-05 C 1039 5 1 0 0 0 2 1 1 0 0 0

CYP4F8 -0.75790989123844 1.12904835622694e-05 7.78658943218961e-05 C 1 0 0 0 0 0 0 0 0 0 0 0

PGLYRP3 -0.757922230773866 0.000206378797751847 0.000934481221303639 NA

ACOX2 -0.758084457190409 1.04335589467984e-05 7.27623222841448e-05 C 2 0 0 0 0 0 0 0 0 0 0 0

PLEKHA7 -0.758491661351861 8.87442038604447e-12 3.0876131368872e-10 C 1 0 0 0 0 0 0 0 0 0 0 0

MAMDC2 -0.758721859547545 2.75484395184916e-05 0.000168196553150481 NA

FAM13C -0.759474295116454 1.23717293911756e-08 1.9810467190083e-07 NA

RGS11 -0.759838630174695 9.83115448290187e-09 1.63366339682075e-07 C 2 0 0 0 0 0 0 0 0 0 0 0

ASAP3 -0.760260882350819 1.59330965364292e-07 1.91957809131124e-06 C 5 2 0 0 0 0 0 2 0 0 0 0

GLB1L2 -0.760553656199588 4.59215728715306e-13 2.16747533600863e-11 NA

FUT7 -0.761151129388509 4.81233885595549e-05 0.000269659800323809 C 19 3 0 1 0 0 0 2 0 0 0 0

PSCA -0.761624446958592 0.000161015287352643 0.000758470966581254 C 117 22 9 2 6 0 1 7 2 0 0 0

C10orf90 -0.762673694833167 1.64612851919937e-10 4.18767130146324e-09 C 94 1 1 0 0 0 0 0 0 0 0 0

SERPINI2 -0.762702502489675 1.10319464553357e-08 1.79537102803215e-07 C 2 0 0 0 0 0 0 0 0 0 0 0

MS4A2 -0.7630120055 0.0015790592 0.0051036293 MN 8567 1 1 0 0 0 0 0 0 0 0 0

LOC100291666 -0.764321012785359 3.53470055396407e-08 5.05296656985484e-07 NA

PANK1 -0.765024739909225 2.20403882202392e-08 3.3426156077281e-07 C 2 0 0 0 0 0 0 0 0 0 0 0

FLVCR2 -0.765127366707768 8.11246555051064e-07 7.95156061494121e-06 NA

AWAT1 -0.765429673379886 4.71855405915155e-08 6.54751265964527e-07 C 1

PALMD -0.766089258957511 6.82089269750804e-06 5.03506380989605e-05 C 1 0 0 0 0 0 0 0 0 0 0 0

KLB -0.766138281871913 9.68022725729474e-08 1.24118980923704e-06 C 7 0 0 0 0 0 0 0 0 0 0 0

FAM43A -0.76708863948318 5.50065640430496e-05 0.000302736038860948 C 1

ZNF415 -0.76738446521513 9.01043160532903e-08 1.16648727082122e-06 C 1

RORC -0.76789679316732 1.09583741367595e-08 1.78800989040041e-07 C 151 2 0 0 0 0 1 0 1 0 0 0

LIFR -0.768246566733975 2.90734258965918e-05 0.000175805984646899 C 66 1 0 1 0 0 0 1 0 0 0 0

KBTBD11 -0.768373722903743 1.35085616369227e-07 1.66347785362418e-06 NA

SLC35G5 -0.768478137402945 2.66361680525987e-08 3.94786807150772e-07 C 4 0 0 0 0 0 0 0 0 0 0 0

AS3MT -0.768976587595381 9.42003664534543e-10 2.018073405239e-08 C 5

KRT72 -0.769317745082776 8.20131609461086e-08 1.07571940209771e-06 C 1 0 0 0 0 0 0 0 0 0 0 0

CALML6 -0.769486374970527 8.95085593116457e-10 1.9339366462232e-08 C 3 0 0 0 0 0 0 0 0 0 0 0

MAST4-AS1 -0.770037341950631 1.69701291704319e-06 1.50512481166244e-05 NA

MYLIP -0.770284378798224 1.24407606998523e-07 1.54302934315927e-06 C 3559 0 0 0 0 0 0 0 0 0 0 0

C15orf48 -0.770471334909097 0.000151968182016393 0.000721963298449869 C 4

DUOXA2 -0.770651627203002 8.4918516865256e-08 1.10768626375514e-06 C 2 0 0 0 0 0 0 0 0 0 0 0

CDON -0.771209050495622 4.94464342383814e-10 1.12891756433033e-08 C 3 1 1 0 0 0 0 0 0 0 0 0

SLITRK5 -0.771219557276965 3.07468367923042e-10 7.33292613882276e-09 C 2

PITPNM3 -0.771574823796316 3.47499731408878e-09 6.51200734294637e-08 C 4 2 1 0 0 0 0 2 0 0 0 0

NOV -0.771670026925236 2.01365388784333e-06 1.75552405044729e-05 C 140 12 2 0 1 0 0 5 5 0 0 0

FAIM2 -0.772457786035401 3.12278100803291e-11 9.39586952453823e-10 C 5 0 0 0 0 0 0 0 0 0 0 0

CMTM5 -0.773157182769729 4.09788247752913e-09 7.55215400702959e-08 C 7 1 0 0 0 0 1 0 0 0 0 0

ARHGAP20 -0.774001004451437 4.08208996081513e-10 9.51259784176779e-09 C 2

ISM2 -0.774398668863024 1.02860329082289e-09 2.17638095646099e-08 NA

KRTAP11-1 -0.774721706901962 2.62542479883976e-07 2.9898565082816e-06 C 1

SNX21 -0.774850973086729 1.79189298248139e-16 1.94914703904743e-14 NA

OR10A2 -0.775301798724679 4.02006673385364e-07 4.31826351144426e-06 NA

BDKRB1 -0.77565548773847 1.02250401084514e-06 9.7348759624074e-06 C 10 1 0 1 0 0 0 0 0 0 0 0

RNASE4 -0.77615839791592 2.30355761648086e-07 2.66012416150905e-06 C 1 0 0 0 0 0 0 0 0 0 0 0

SSC5D -0.777624323120016 2.95117012111427e-05 0.00017820102274918 NA

PYGO1 -0.779138418346971 1.63456834709439e-06 1.4558811814332e-05 C 1 0 0 0 0 0 0 0 0 0 0 0

BMX -0.77928976660651 1.68456518870316e-05 0.000111403792196313 C 40 11 3 1 0 1 5 2 0 0 0 0

MUC20 -0.779451088925884 7.31061572685683e-05 0.00038943997709603 C 3 0 0 0 0 0 0 0 0 0 0 0

IL1RL2 -0.780325080959304 2.75812071566944e-11 8.47450499764213e-10 NA

LOC100131232 -0.781600653750995 2.37327853875884e-06 2.02338031094993e-05 NA

LGR4 -0.781734382270375 1.66056100294853e-12 6.90756881380369e-11 C 6 1 0 0 0 0 0 1 0 0 0 0

FA2H -0.782565069100741 0.00132153238596472 0.00439435057434193 C 14 0 0 0 0 0 0 0 0 0 0 0

UNC13B -0.783348032882323 1.11722473539035e-08 1.8119719423079e-07 C 1 0 0 0 0 0 0 0 0 0 0 0

MAB21L2 -0.783901531467561 4.77549465981524e-08 6.6023219449469e-07 C 1

TNFRSF11A -0.784846121136994 4.35912681499654e-07 4.62784951095579e-06 NA

LINC00282 -0.786026491144033 5.48622467557216e-09 9.78678364133404e-08 NA

PIGN -0.786224581687332 1.48151256018712e-09 3.01187843465752e-08 C 1 0 0 0 0 0 0 0 0 0 0 0

CRABP1 -0.786425738897591 8.6472679628042e-07 8.39747771841946e-06 C 73 5 2 1 0 0 0 2 0 0 0 0

BTC -0.786450487123256 4.51591934077649e-07 4.76702762760049e-06 C 113 5 1 0 0 0 1 1 2 0 0 0

PHACTR3 -0.78712283858558 0.00221351603692407 0.00678275635728165 C 2 0 0 0 0 0 0 0 0 0 0 0

MAS1L -0.787153744624268 5.84179033048474e-07 5.97339630389436e-06 C 9 1 0 0 0 0 1 0 0 0 0 0

SLC38A3 -0.788176656100005 5.51933327667526e-09 9.82732087748191e-08 C 60 3 1 0 1 0 0 2 0 0 0 0

GAS7 -0.788264396629643 2.02178534075315e-07 2.36943226900526e-06 C 4 0 0 0 0 0 0 0 0 0 0 0

HTR2A -0.788480677515938 0.000970653360788936 0.00341035013173421 C 2 0 0 0 0 0 0 0 0 0 0 0

TSPYL5 -0.789040735924412 1.40775760270463e-06 1.2779198151746e-05 C 7 3 0 0 0 0 1 1 3 0 0 0

PRR15L -0.790619344721774 0.000652691827124741 0.0024549877209837 C 1

MAPK7 -0.791773880483723 3.15694895170425e-15 2.62068301793449e-13 C 81 16 4 0 0 0 3 8 5 0 0 0

SNED1 -0.792486699841947 3.24524416691961e-11 9.68812876140182e-10 C 29 2 1 0 1 0 0 0 1 0 0 0

KRT73 -0.793398052530236 9.07363463412942e-11 2.44639149174028e-09 NA

CD1C -0.793791058481279 1.85353873647394e-05 0.000120930464202834 C 472 20 3 4 5 0 0 5 5 0 0 1

NELL1 -0.794084146802358 7.0137107386119e-06 5.15328040177436e-05 C 75 6 2 0 0 3 1 1 0 0 0 0

SDR16C5 -0.794179689904733 0.000243363278671937 0.00107069660051691 C 3 0 0 0 0 0 0 0 0 0 0 0

TMEM220 -0.794804036932448 2.16464846159849e-07 2.51660328210532e-06 NA

DNASE1L2 -0.795978780471266 0.000147092330655328 0.000702502281683923 C 1

DBX2 -0.796227716217375 3.40299099524631e-11 1.00795634690183e-09 NA

FCN1 -0.797239361656749 3.584666477187e-07 3.91500187038191e-06 C 1 0 0 0 0 0 0 0 0 0 0 0

IL20RB -0.797856074310983 8.86227136204532e-05 0.000458420907541492 C 10 0 0 0 0 0 0 0 0 0 0 0

ADAMTS18 -0.798248101051443 2.89626209495727e-12 1.12710563162115e-10 C 4 1 0 0 0 0 0 1 0 0 0 0

TTC18 -0.79832181272441 0.000397950395203868 0.00161774385733428 NA

FAM90A1 -0.798583787680574 1.50209923124717e-05 0.000100460184274965 NA

ZNF829 -0.799083989245166 2.12314604796194e-12 8.64188930102702e-11 NA

ADAMTSL3 -0.799129980233491 7.31703775985435e-08 9.70494559781102e-07 C 2

C9orf9 -0.79931130956083 5.66652471838448e-13 2.6094966750575e-11 C 2

FOXP2 -0.800553797951101 3.94983039357477e-06 3.15663691384886e-05 C 16 0 0 0 0 0 0 0 0 0 0 0

PALM -0.801089204240914 4.25344736303244e-09 7.79332025557745e-08 C 97 4 0 1 0 0 0 2 2 0 0 0

TPSAB1 -0.801633750398322 0.000775425985220005 0.0028376812881398 C 1 0 0 0 0 0 0 0 0 0 0 0

SVIP -0.80167784986941 5.60185324113742e-10 1.26523002738673e-08 C 1 0 0 0 0 0 0 0 0 0 0 0

SLC9A3 -0.801987248037388 1.47991694002608e-11 4.84608787610269e-10 C 7 1 0 0 0 0 0 0 1 0 0 0

ABLIM2 -0.803020586646038 4.88719561625145e-06 3.78632629671679e-05 NA

WDR49 -0.803909896952095 2.75181372519663e-13 1.36344446012557e-11 NA

UNC93A -0.804518060732107 0.000577165537631676 0.00221313049650622 C 1

CD19 -0.80455619913164 0.000352746993340596 0.0014628488919714 C 880 39 21 1 6 0 5 1 8 0 1 0

FAAH2 -0.805368305871501 8.32042876356632e-07 8.12594196119813e-06 NA

GAS1 -0.806198293544761 6.41249684258939e-07 6.44210869106631e-06 C 33 5 1 0 0 0 4 1 2 0 0 0

CADM4 -0.807410901478702 9.55336552433884e-07 9.19717951572539e-06 C 5 2 0 0 0 0 0 2 0 0 0 0

BEND5 -0.807455068752795 1.96092552279711e-06 1.71508490619135e-05 NA

NOXA1 -0.808080893819178 1.40864272645876e-08 2.22363476928149e-07 C 4 0 0 0 0 0 0 0 0 0 0 0

PLCD1 -0.80809652090015 5.9956034466253e-07 6.08791772716079e-06 C 4 1 0 1 0 0 0 1 0 0 0 0

KCTD4 -0.808500313143826 1.13335534442718e-09 2.36766187681823e-08 NA

MKNK2 -0.809120108974065 5.4916134823332e-13 2.53511630195416e-11 C 10 1 1 0 0 0 0 0 0 0 0 0

RALGPS1 -0.810073071888256 1.66847025356084e-14 1.13594016148008e-12 C 1

SLC5A10 -0.810142524080371 3.95053820792936e-06 3.15663691384886e-05 NA

NUCB2 -0.811015882217087 1.09120156833148e-09 2.29224995380798e-08 C 6 2 1 0 0 0 1 1 2 0 0 0

OR10H3 -0.812044624539142 2.44413945064203e-07 2.79686985382719e-06 NA

PPIL6 -0.812959895145893 1.50788623877018e-11 4.92064876572469e-10 C 12 0 0 0 0 0 0 0 0 0 0 0

AMOT -0.813249106518339 6.95626210359776e-09 1.20128807331017e-07 C 10 4 1 1 0 2 0 1 0 0 0 0

SORCS1 -0.813337067482384 1.17048077728263e-07 1.46325559257783e-06 C 1

LNX1 -0.81342071409478 2.39323943569539e-06 2.03764475031069e-05 C 6 0 0 0 0 0 0 0 0 0 0 0

ZNF418 -0.813487945939249 1.36802588753313e-18 2.39746536790181e-16 NA

NDRG2 -0.8140515614428 7.68371418943488e-05 0.000405774716694849 C 44 7 0 2 0 0 0 3 2 0 0 0

VWA5A -0.814825448075411 4.06831110990598e-07 4.36760773551847e-06 C 7 3 2 1 0 0 0 0 0 0 0 0

UGT1A6 -0.817047872871673 0.00231478331207606 0.00703128172588215 C 22 1 1 0 1 0 0 0 0 0 0 0

NOTCH3 -0.817131998573868 1.33492012090519e-06 1.22176175669113e-05 C 123 30 13 4 1 0 5 4 13 0 0 0

SYDE2 -0.817415406539858 8.54701804868219e-07 8.31719334742456e-06 NA

SPRR1B -0.817820970551177 0.00513132084527738 0.0137021035043122 C 7 1 0 0 0 0 0 0 1 0 0 0

ZNF91 -0.818079733462524 0.000358444402432165 0.00148095987881108 C 1 0 0 0 0 0 0 0 0 0 0 0

PPARG -0.818453771050163 2.04408175932346e-05 0.000131102458348746 MN 13 1 0 0 0 0 0 0 1 0 0 0

MEX3B -0.818751163254516 6.17203062273796e-11 1.72044217373433e-09 NA

SNX31 -0.819402812398413 1.25399564016518e-08 2.00459252376742e-07 C 1

TEK -0.819936207299094 3.21540298392876e-05 0.000191835917784646 C 418 54 16 5 2 31 1 8 3 0 0 2

GABRQ -0.820700084480113 1.5129861360663e-07 1.83565952546967e-06 NA

TNMD -0.82128608119009 1.10768021425469e-11 3.76336692416532e-10 NA

RARG -0.821491263445638 4.03330596776823e-12 1.52068490143325e-10 C 123 5 1 0 0 0 2 1 3 0 0 0

SMPD3 -0.822245946497873 8.45109337247361e-07 8.23655222764202e-06 C 15 2 0 1 0 1 0 1 0 0 0 0

FAM124B -0.822462081948734 7.65874073238784e-07 7.5498430125992e-06 C 1

RASL11A -0.822621343017563 1.10950427626367e-11 3.76336692416532e-10 C 2

ALAS2 -0.824527495755341 6.10585482090798e-06 4.58229635984637e-05 C 14 0 0 0 0 0 0 0 0 0 0 0

NKPD1 -0.825340434721941 9.34830988855209e-08 1.20692674802609e-06 NA

FBLL1 -0.825787568145692 2.05509363306647e-06 1.78671369742991e-05 NA

LOC100131662 -0.825812029226191 5.88089288953759e-12 2.11611520380756e-10 NA

MLPH -0.82709554302194 4.22363970977702e-08 5.92593245270198e-07 C 3 1 0 0 0 0 0 0 1 0 0 0

LGALSL -0.827796401919223 0.000647652149539492 0.00243902256189497 C 389 2 1 0 0 0 0 0 1 0 0 0

DLG2 -0.829562809689234 3.69426887593979e-13 1.7837098728294e-11 C 4

KRT33B -0.830379153215296 2.84317990946208e-08 4.16444386602581e-07 C 1

ATP1A2 -0.830871440462576 0.00089828602038741 0.00320306320796392 C 1 0 0 0 0 0 0 0 0 0 0 0

ZNF542 -0.830970335978266 3.84357276346195e-11 1.11918925683145e-09 NA

SAMD5 -0.831267827279765 1.41847947234018e-05 9.54410272768666e-05 C 1

FGFR2 -0.831345581468402 9.62365129762476e-11 2.57633448529199e-09 MN 16 2 1 0 0 0 1 0 1 0 0 0

C11orf93 -0.83191101263524 3.72880418659625e-05 0.000217220919789804 NA

KRT74 -0.832471771363094 1.35082924678513e-07 1.66347785362418e-06 C 1 0 0 0 0 0 0 0 0 0 0 0

DLX4 -0.833027304530317 8.4314707838689e-06 6.03336285543617e-05 C 329 12 4 2 0 0 2 5 1 0 0 0

CLDN8 -0.834829782681597 3.46525301860838e-06 2.81851499283201e-05 C 6 0 0 0 0 0 0 0 0 0 0 0

CDH19 -0.834992571970881 7.15862057764646e-09 1.23285906890914e-07 C 1 0 0 0 0 0 0 0 0 0 0 0

PMP2 -0.835154258637542 9.62575407547889e-13 4.1881987904963e-11 C 4 0 0 0 0 0 0 0 0 0 0 0

ZDHHC21 -0.835327768436954 0.000502254407021877 0.00196855853421062 NA

EHD3 -0.836675411661776 3.01663063508365e-08 4.37180459649528e-07 C 1 0 0 0 0 0 0 0 0 0 0 0

PKHD1L1 -0.836884985692764 6.40309344714375e-12 2.27375890570525e-10 NA

NLGN1 -0.837004959075988 2.77717420810799e-10 6.71310041339208e-09 C 3 0 0 0 0 0 0 0 0 0 0 0

ALAD -0.837299896327537 1.87514182547051e-19 3.90008893743741e-17 C 6 0 0 0 0 0 0 0 0 0 0 0

MYO5C -0.838387682820291 2.38721021854885e-06 2.03342609390072e-05 NA

ZNF681 -0.83943383857761 2.54401274592322e-08 3.7943679465791e-07 NA

SYT15 -0.839534928313991 7.64101847427139e-09 1.31116551824601e-07 NA

PCDHB5 -0.839887628102139 7.72281868877556e-06 5.59180525334564e-05 NA

SLIT3 -0.840230126614868 1.69218302497491e-09 3.40145773915102e-08 C 58 18 3 2 2 4 2 13 4 0 0 0

SELENBP1 -0.840463647698296 2.26567360435523e-08 3.42237863604401e-07 C 21 2 0 1 0 0 0 1 1 0 0 0

XYLT1 -0.840859421633447 5.45302983319151e-07 5.60616489151633e-06 C 2 0 0 0 0 0 0 0 0 0 0 0

SPNS2 -0.841235486977098 4.25893034693654e-08 5.9666006422256e-07 NA

TRPV6 -0.842203041127859 2.58615557687476e-06 2.17257730153168e-05 C 53 16 6 4 0 0 6 1 5 0 0 0

TCEA3 -0.84238390576728 2.63367868930189e-05 0.000161948136947423 C 2 1 0 0 0 0 0 0 1 0 0 0

CXCR2 -0.842449208353187 8.96518198678623e-08 1.16142367873992e-06 C 194 39 11 1 1 17 4 18 7 0 0 2

RGS5 -0.84309460201483 0.000117814574588995 0.000583279218740756 C 22 3 0 0 0 0 0 3 0 0 0 0

CYP3A5 -0.843098974682472 1.68637403747545e-07 2.01502534136981e-06 MN 3 1 0 0 0 0 0 0 1 0 0 0

C11orf45 -0.843429993196529 1.35781218688625e-09 2.78131074255368e-08 NA

NLRP2 -0.844092410118797 0.00489638757187287 0.0131770123095177 C 86 8 0 1 0 0 4 3 1 0 0 0

GAMT -0.844709859955181 1.9973181353143e-06 1.74288798280746e-05 C 3 0 0 0 0 0 0 0 0 0 0 0

ABCB1 -0.845504420790796 8.28069966868339e-09 1.40690128033367e-07 C 1456 178 83 23 5 1 58 28 12 0 0 0

TIFA -0.845737376379583 1.49764134091345e-14 1.03452035253536e-12 C 1

TRIM43 -0.846108919831995 0.000112622610986795 0.000560507009767831 NA

RERGL -0.847581362341463 1.47767490555162e-07 1.8008984505715e-06 NA

ZNF717 -0.847701949322369 3.86289282586607e-07 4.17073431347217e-06 C 2 0 0 0 0 0 0 0 0 0 0 0

CERS3 -0.847870598299894 2.37422394954349e-05 0.0001484046786427 C 1 0 0 0 0 0 0 0 0 0 0 0

LOC441461 -0.848061656288325 1.91137888497101e-05 0.000124147797377647 NA

KCNK12 -0.848757963935869 3.38644652742813e-11 1.00462811010395e-09 C 1 0 0 0 0 0 0 0 0 0 0 0

CP -0.848961885017891 0.00310685906629583 0.00896395145545444 MN 7 1 1 0 0 0 0 0 0 0 0 0

LOC100128288 -0.849239652294815 8.30683369949326e-13 3.65635910303044e-11 NA

LGR6 -0.849599875471977 1.23529555295223e-08 1.97971540480329e-07 C 190 2 2 0 0 0 1 0 0 0 0 0

ZNF540 -0.851245573468813 9.96749203764663e-14 5.48414888943424e-12 C 1

KIAA0125 -0.851435146461986 0.000163783338859206 0.000769524303180681 C 2

ZNF347 -0.851634956444886 5.86857959145219e-07 5.98462316419266e-06 NA

TMEM132C -0.853085349666775 1.19674740692409e-12 5.07866326700722e-11 NA

VASN -0.853271931323859 3.86488299759842e-11 1.12366575261974e-09 C 2 1 0 0 0 0 0 0 1 0 0 0

SMAD5-AS1 -0.854618421091241 9.72642889193379e-11 2.60017118132247e-09 C 29 0 0 0 0 0 0 0 0 0 0 0

FRY -0.85464443595547 2.58674709906013e-09 4.99585330039909e-08 C 5 0 0 0 0 0 0 0 0 0 0 0

SGCG -0.854951987389929 0.00707691902436621 0.0179647057905283 NA

PVRL4 -0.856251526213889 1.08122177003491e-05 7.50157054305382e-05 C 11 3 1 1 0 0 0 0 1 0 0 0

LOC730101 -0.856523637965956 2.4116666294055e-10 5.89736618795321e-09 NA

C11orf52 -0.856850053625416 3.60877452925349e-05 0.000211073162902289 NA

C1orf170 -0.857346395368344 2.0378931979368e-09 4.03464482817466e-08 NA

LOC440300 -0.857478812254139 4.10502739557921e-08 5.78094148185474e-07 NA

HLA-DQA1 -0.859340961895127 4.37693356341459e-07 4.64356126829012e-06 C 94 2 0 1 0 0 0 1 0 0 0 0

ZDHHC15 -0.860346867630844 7.42154235492973e-20 1.63334339711343e-17 NA

PLA2G4F -0.861051041458551 3.73336446905413e-06 3.01071109100074e-05 NA

MAL2 -0.861884730527103 0.000833894547899149 0.00300516414853146 C 9 0 0 0 0 0 0 0 0 0 0 0

HSPB8 -0.86196057172676 0.00207355587019201 0.00642853267078201 C 34 7 3 0 0 0 2 0 3 0 0 1

TTR -0.862533277155353 4.06749617140711e-10 9.49266338301139e-09 C 273 6 0 1 1 1 1 4 0 0 0 1

ZFP2 -0.863400284737458 1.10478091801888e-08 1.79640794118069e-07 NA

LINC00341 -0.864562705009247 3.07621036309255e-10 7.33292613882276e-09 NA

FGF12 -0.864874217544292 4.966629571394e-10 1.13257105900933e-08 C 4 1 0 1 1 0 0 0 0 0 0 0

KAZALD1 -0.864877416313482 4.66856947842647e-09 8.45569516920362e-08 C 2

RHOV -0.865256905156061 2.09604148709262e-05 0.000133981010557926 C 96 1 0 0 0 0 1 0 0 0 0 0

MMRN1 -0.865592923819283 6.60601356558048e-06 4.8936210863304e-05 C 2036 49 5 2 0 4 4 35 6 0 0 0

ETNK2 -0.866382392610668 3.19372450601511e-08 4.59677746960821e-07 NA

ADAD2 -0.866494076821878 1.40101919618322e-11 4.61970214741461e-10 NA

ZNF287 -0.86718277963645 1.37088569420637e-10 3.55922545051357e-09 NA

KCNB2 -0.867637987984998 3.22622547559388e-08 4.63650490330792e-07 C 1 0 0 0 0 0 0 0 0 0 0 0

PAIP2B -0.868365102146973 4.15167584312467e-06 3.29332643264127e-05 NA

XAGE3 -0.868965938688916 0.000143069718064663 0.000685301582689132 C 1 0 0 0 0 0 0 0 0 0 0 0

LOC642620 -0.869500370993115 3.79244548797548e-08 5.376750243514e-07 NA

ANGPT4 -0.869629128107776 6.63987391855232e-08 8.88147658349397e-07 C 10 3 1 1 0 2 1 1 1 0 0 0

MAP2 -0.870504725947381 3.29885919578439e-07 3.6364302853006e-06 C 110 6 1 0 2 2 0 2 1 0 0 0

SPRR1A -0.870709740021461 0.00226801951200437 0.00691141608496324 C 7 1 0 0 0 0 0 1 0 0 0 0

PGM5 -0.871466354948333 9.95430324149766e-15 7.41752352172544e-13 NA

GREM2 -0.872149259634467 1.70416388344232e-10 4.30636980265858e-09 C 1 0 0 0 0 0 0 0 0 0 0 0

FOXE1 -0.872410678670719 4.23122969528819e-05 0.000242020200793955 C 14 1 0 0 0 0 1 0 0 0 0 1

FREM1 -0.873290886935911 5.15265900180701e-06 3.95636417554569e-05 C 3

NEGR1 -0.873341931266632 5.73115475091989e-10 1.28981648003164e-08 C 1 1 0 1 0 0 0 0 1 0 0 0

ALDOC -0.873753625030242 2.22338104867707e-05 0.00014083645618578 C 1 0 0 0 0 0 0 0 0 0 0 0

PTGR1 -0.874397421319414 3.69884911034438e-07 4.02345500640736e-06 C 2 0 0 0 0 0 0 0 0 0 0 0

ZNF229 -0.87456329258055 5.36548677923365e-14 3.15380646802967e-12 NA

MIA -0.875289944941797 0.000607320752366409 0.00231143371808547 MN 4 1 0 0 0 0 0 1 0 0 0 0

LONRF2 -0.875997636506204 3.37563360561941e-09 6.33835488626573e-08 NA

ZNF570 -0.876693507091166 1.17008910161702e-11 3.93361925866879e-10 NA

EDNRB -0.877652019927425 4.88787017605687e-09 8.80235193360878e-08 C 192 10 2 3 0 0 1 4 2 0 0 0

SPATA6 -0.877922106142075 9.06059423347286e-10 1.95541467567778e-08 C 12 0 0 0 0 0 0 0 0 0 0 0

DIRAS1 -0.879054700052474 1.79718291734029e-07 2.1366382585741e-06 C 45 1 1 0 0 0 1 1 0 0 0 0

GNRHR -0.87930348806224 9.80226060456733e-09 1.63029337840638e-07 C 116 18 6 0 0 0 2 7 6 0 0 0

FLJ13197 -0.879657244524921 7.72697282028075e-10 1.70039342854276e-08 NA

CYP4F3 -0.882510689373999 0.000749254027786861 0.00275682950698326 C 114 2 0 0 0 0 1 0 1 0 0 0

SLC35F1 -0.882663333910343 8.91057089836726e-12 3.09450230079628e-10 NA

APCDD1 -0.883924268064864 2.02181549218277e-06 1.76101711093158e-05 NA

ABCA3 -0.884139272967391 1.78338695581596e-07 2.12290345363074e-06 C 5 2 2 0 0 0 0 0 0 0 0 0

SCNN1B -0.884269201584425 3.67056446028327e-09 6.85135833725656e-08 C 2

SFRP4 -0.884475976761113 0.0199234296489197 0.0424651748834575 MN 216 18 3 1 2 3 5 1 7 0 0 0

NGEF -0.884503209937402 0.00101159548548794 0.00353559776404218 C 1

ZNF582 -0.885130851609982 4.22194192023794e-11 1.21073779885369e-09 C 1

CFD -0.886615404064353 4.90534439680363e-08 6.75716545839172e-07 C 32 1 0 1 0 0 0 0 0 0 0 0

LBP -0.886631144429475 8.98548678628987e-09 1.51037574071144e-07 C 33 1 1 0 0 0 0 0 0 0 0 0

SYT17 -0.88666594920364 6.59887626755853e-09 1.1479497345228e-07 NA

NRTN -0.886751157478802 1.43185920170875e-05 9.62382736197965e-05 C 10 1 0 0 0 0 1 0 0 0 0 0

RFPL1 -0.886837007345736 6.4364734700543e-06 4.79396954484479e-05 C 1 0 0 0 0 0 0 0 0 0 0 0

PRIMA1 -0.887217049639225 1.40730153271603e-13 7.46106333605497e-12 NA

ARSF -0.887956743145482 6.1034336877558e-08 8.26912594188647e-07 C 36 2 1 0 0 0 0 1 0 0 0 0

JMJD7-PLA2G4B -0.888340606490981 8.34603155815824e-08 1.09162132501857e-06 NA

GPIHBP1 -0.889518274291833 1.42394685769103e-05 9.57408247798155e-05 C 1

NDN -0.889733465691762 1.59541833031072e-06 1.42548356336426e-05 C 20 2 0 0 0 0 1 0 1 0 0 0

CCR2 -0.890155779291881 0.001260682698719 0.00422467093460596 C 134 16 6 0 0 2 0 8 1 0 0 0

LOC284244 -0.890429982442271 2.5251801252904e-11 7.91292785287606e-10 NA

CIDEB -0.890665517735758 1.9000861970385e-07 2.24627929115226e-06 C 7 2 0 1 0 0 1 1 0 0 0 0

CRABP2 -0.891382365630345 5.023023963769e-05 0.000279866866535931 C 56 6 4 1 0 0 0 1 1 0 0 0

TMPRSS2 -0.891627196977131 2.28840455797081e-06 1.95896124236605e-05 C 140 20 3 2 4 0 2 11 2 0 0 0

KLHDC9 -0.892065143493129 9.59050713784948e-06 6.76302267504013e-05 C 1

ZNF521 -0.894644286253021 2.67923120677575e-06 2.24141841953312e-05 C 1 0 0 0 0 0 0 0 0 0 0 0

SLC25A21 -0.894758920247109 6.33380552881655e-05 0.000343357743056989 NA

EPB41L4A -0.896210353307542 1.45881002769276e-12 6.13575497647574e-11 C 2 0 0 0 0 0 0 0 0 0 0 0

ZNF420 -0.896339863659822 1.49726261897193e-09 3.03737294633246e-08 NA

CYP4X1 -0.897354535714809 0.000716694549533694 0.00265509448796716 C 1 0 0 0 0 0 0 0 0 0 0 0

LINC00087 -0.899359111066989 7.12057054194564e-12 2.50503789307444e-10 NA

MKX -0.899630475125422 1.2199028194969e-12 5.16534690483621e-11 NA

RIPPLY1 -0.902752471333237 0.00128016635994803 0.00427331723011224 NA

RNF125 -0.902787436724924 1.15022626027631e-06 1.07720595884461e-05 C 1 0 0 0 0 0 0 0 0 0 0 0

COL21A1 -0.902870079187537 1.69837924653296e-08 2.63701591461274e-07 C 1 0 0 0 0 0 0 0 0 0 0 0

FBXL16 -0.903065144491881 1.1883477632568e-09 2.47163275990785e-08 NA

TPRG1 -0.903625742157775 6.95149190940473e-06 5.11153408583152e-05 C 1

KIT -0.90380939610136 4.08292155181662e-07 4.38080817523997e-06 MN 81 4 0 2 2 0 0 1 0 0 0 0

GPD1L -0.904017257939004 4.76118250240792e-10 1.08966023244347e-08 C 1

ACADL -0.904600357910924 3.2842377401089e-14 2.05151048538089e-12 C 2 0 0 0 0 0 0 0 0 0 0 0

ATP1B2 -0.90530542502951 1.31421907756667e-07 1.6215270196287e-06 C 4 2 0 0 1 0 0 1 0 0 0 0

TIMP4 -0.906059034836015 6.14683236420824e-08 8.32196682098493e-07 C 35 11 2 4 1 0 1 6 0 0 0 0

CIDEA -0.906623713523335 8.69623648598664e-08 1.13122795855855e-06 C 5 0 0 0 0 0 0 0 0 0 0 0

IL34 -0.907151511010142 9.48751721423238e-11 2.54348779481269e-09 C 5 0 0 0 0 0 0 0 0 0 0 0

ZSCAN18 -0.907564144775138 2.53849563458759e-17 3.45655445149923e-15 C 3 0 0 0 0 0 0 0 0 0 0 0

ZFP28 -0.908688197449888 1.16612390661565e-14 8.47045011372798e-13 NA

TMEM25 -0.909042638776164 1.48765000213985e-07 1.81188877673751e-06 C 2

CTSG -0.909696282608141 0.000114749156900728 0.000569891706287086 C 43 3 2 0 0 0 0 2 0 0 0 0

CAMSAP3 -0.90974775887031 1.39142646193653e-08 2.20196727801612e-07 NA

SLC25A23 -0.91075308383424 1.30489982838682e-07 1.61107886835469e-06 C 5 0 0 0 0 0 0 0 0 0 0 0

SLC27A2 -0.911777707100267 4.77999714456955e-06 3.71239252996585e-05 C 10 0 0 0 0 0 0 0 0 0 0 0

NDUFA4L2 -0.912482064881154 0.00129829474741234 0.00432848770200342 NA

ASPA -0.913676279851097 9.21289523401521e-15 6.91954238469071e-13 C 859 1 1 0 0 0 0 0 0 0 0 0

IGSF10 -0.91375625685685 9.14854145296641e-12 3.16552914223575e-10 C 1

SORT1 -0.915825113090493 2.43682965734865e-06 2.06917339276078e-05 C 28 1 0 0 0 0 0 1 0 0 0 0

CFTR -0.91612841808863 2.44252089774872e-10 5.96510877828258e-09 C 39 8 2 2 1 0 3 0 1 0 0 1

GDPD3 -0.917517578827038 5.49282311048897e-05 0.000302401515581796 NA

FOXN1 -0.918113934032981 5.00527877162263e-06 3.86042833376126e-05 C 14 0 0 0 0 0 0 0 0 0 0 0

SLC46A2 -0.918570996752605 4.97014131498039e-13 2.31129888620722e-11 C 2

NTRK3 -0.918579980653812 7.12814780432145e-17 8.53888946154381e-15 C 171 20 1 4 1 0 8 7 7 0 0 0

EVPL -0.918763496866283 2.08969033671741e-12 8.54245550821821e-11 C 207 16 5 0 0 0 6 0 6 0 0 0

ACPP -0.918987359472572 9.97859002363117e-06 7.00277246485974e-05 C 1174 97 15 15 17 0 3 62 8 0 0 2

SCN2B -0.919181773456747 1.58304883569364e-07 1.90964724749354e-06 C 1 0 0 0 0 0 0 0 0 0 0 0

CBR1 -0.91961381259576 5.60489975934078e-14 3.28433243792702e-12 C 64 2 1 1 0 0 0 0 0 0 0 0

ZNF606 -0.919876854437925 6.20099393322984e-08 8.37134180986028e-07 NA

SMPD2 -0.921423622035985 1.70238126236035e-16 1.87331221817991e-14 C 62 8 0 0 0 0 6 0 2 0 0 0

ZNF132 -0.92216392731752 2.86266594009989e-11 8.72490793047837e-10 C 1 0 0 0 0 0 0 0 0 0 0 0

SEMA3G -0.923104062616436 1.553056753193e-08 2.42330627928144e-07 C 6 2 0 1 0 0 0 1 0 0 0 0

ZNF300P1 -0.923213200199948 1.35889645008414e-07 1.67011903316509e-06 NA

ALDH3A2 -0.924028387251312 4.61141233191515e-09 8.36818803510624e-08 NA

KRT71 -0.924527878669456 5.15722801759169e-11 1.46124033965506e-09 C 4

PDLIM2 -0.924764312815328 4.44378630713585e-14 2.64508481129424e-12 NA

SPON1 -0.925676846906618 0.000546851428636799 0.00211618421382308 C 7 0 0 0 0 0 0 0 0 0 0 0

SCNN1G -0.926095960922636 8.87950688202032e-07 8.60534699211463e-06 C 1 0 0 0 0 0 0 0 0 0 0 0

DIRAS3 -0.926407196869845 1.62817220826301e-10 4.14756600077981e-09 C 42 9 1 1 0 0 4 4 3 0 0 0

ZNF486 -0.927394560971534 7.49504734203252e-10 1.65529476129113e-08 NA

KLK10 -0.929508378599468 0.00815984506908168 0.0202386826919813 MN 1 1 0 0 0 0 0 0 1 0 0 0

PEG3-AS1 -0.929841098084641 3.77464299860096e-10 8.85287088407936e-09 NA

SLC25A41 -0.929899800446132 1.83132693052637e-08 2.82030307681632e-07 C 1

PHYHIP -0.92995442590462 1.61581701235848e-14 1.10406384811946e-12 C 2

CSRNP3 -0.930072991739198 3.29464761956199e-09 6.2109358063197e-08 NA

SPAG17 -0.930175015742127 0.00038444564564969 0.0015702207024626 C 3 0 0 0 0 0 0 0 0 0 0 0

PPAP2B -0.932787893010496 1.62063337164403e-07 1.94506834655083e-06 C 5 0 0 0 0 0 0 0 0 0 0 0

SLITRK3 -0.934385181155633 4.51709043160045e-11 1.28757485841719e-09 C 1

ALDH2 -0.934554390718497 3.16390082482188e-08 4.56426455117406e-07 C 14 0 0 0 0 0 0 0 0 0 0 0

NSUN7 -0.937985871311602 4.43725624274442e-12 1.64997934983151e-10 NA

LCE3C -0.938113551401742 0.00590637448587835 0.0154065531827756 NA

ESYT3 -0.940635451132649 2.02947576707056e-07 2.3769732576327e-06 C 1 0 0 0 0 0 0 0 0 0 0 0

CALML3 -0.94067552621185 3.3635289904826e-12 1.28349825005774e-10 C 20 1 0 0 0 0 0 0 1 0 0 0

ADAM23 -0.942350948789121 0.000332887328446756 0.00139362054092275 C 10 3 0 1 0 0 0 3 0 0 0 0

LINC00086 -0.942649309692149 3.0156254382438e-11 9.11769052230677e-10 NA

GP1BB -0.9431001084946 2.59504689094916e-08 3.85359586660696e-07 C 1

SPTBN2 -0.943589164700658 4.12734426577055e-10 9.59683598504168e-09 C 1

TNFRSF19 -0.944897811720395 3.87629490608872e-14 2.37432471480716e-12 C 172 1 0 0 0 0 0 1 0 0 0 0

ZNF571 -0.945032971107297 2.59392484840417e-09 5.00460913412291e-08 NA

STAB2 -0.945339327192327 4.71967317577254e-10 1.08409289075057e-08 C 21 1 0 0 0 0 0 1 0 0 0 0

SNTG2 -0.945836094461119 4.73327651531361e-08 6.56196781685798e-07 NA

KRT80 -0.94615201061547 0.000479398440695961 0.00189585756102224 NA

CRAT -0.947773053456961 8.68623671173965e-09 1.46789644859907e-07 MN 25 2 0 0 0 0 0 2 0 0 0 0

CLEC3B -0.948094101194457 2.08227138208254e-10 5.14914458172646e-09 C 32 2 0 0 0 1 0 2 0 0 0 0

C1orf51 -0.948557567885018 1.61644032094016e-06 1.44177030888004e-05 NA

CCDC64B -0.949417319771753 5.29553541228427e-06 4.05127723315701e-05 NA

EFHC2 -0.95035734998602 1.42839706453916e-12 6.02121853909416e-11 NA

HTR3B -0.951383901578941 3.10941539151704e-11 9.37132247058011e-10 C 1 0 0 0 0 0 0 0 0 0 0 0

CH25H -0.951532913952155 1.85274472016722e-06 1.6317775392557e-05 C 2 0 0 0 0 0 0 0 0 0 0 0

DARC -0.951996475190938 3.25321529095911e-05 0.000193263043979859 C 49 3 0 0 0 0 0 3 1 0 0 0

CNKSR3 -0.952199449932697 4.43316174347257e-10 1.02700676032687e-08 C 5 2 0 1 0 0 0 1 0 0 0 0

FAM117B -0.952316518539685 2.63299936537639e-11 8.16963589975064e-10 NA

PCDH20 -0.95344748749025 4.14730561945724e-12 1.55746137816403e-10 C 1 0 0 0 0 0 0 0 0 0 0 0

RHBG -0.953911664942209 4.22130497449451e-08 5.92593245270198e-07 C 1 0 0 0 0 0 0 0 0 0 0 0

PRELP -0.956180745559633 7.59504009909662e-07 7.5026787033195e-06 C 1 1 0 0 0 0 0 1 0 0 0 0

MPZL2 -0.956464850384544 1.08321427300414e-09 2.2805335422858e-08 C 10 0 0 0 0 0 0 0 0 0 0 0

MACC1 -0.956873529034088 1.70894443635147e-06 1.51357937982332e-05 C 33 4 1 1 0 0 0 4 0 0 0 0

C8orf48 -0.957404078395075 2.39663746444773e-12 9.50967658062938e-11 NA

HBG1 -0.957705385112093 0.000129945029326647 0.000632229625321957 C 35 1 0 0 0 0 0 0 1 0 0 0

CITED2 -0.957963167590466 1.61737646028922e-12 6.77258501413585e-11 C 18 2 0 0 0 0 1 1 0 0 0 0

MAPK3 -0.958819863927114 1.0227689074855e-13 5.59478240230582e-12 MN 67 2 0 0 0 0 0 1 1 0 0 0

DLEC1 -0.95891645322242 6.57668199822179e-07 6.58426876225075e-06 C 102 24 3 1 2 3 4 12 9 0 0 0

CPLX3 -0.959392366325499 2.01242988834342e-07 2.35992939880272e-06 NA

ULK3 -0.959792714869407 3.51929396415013e-17 4.59377081789446e-15 C 2 0 0 0 0 0 0 0 0 0 0 0

LOC100506874 -0.960052600321514 4.14051943235708e-14 2.49578379924275e-12 NA

TMPRSS11F -0.962915787092977 0.000658597407297792 0.00247326847776296 NA

MASP1 -0.962920232006273 9.29735952123919e-09 1.55401588136543e-07 C 16 0 0 0 0 0 0 0 0 0 0 0

TMEM125 -0.963415036987847 2.34275600941254e-06 2.00006057691254e-05 NA

CALML5 -0.963433095745887 0.0197136301952073 0.0421177429994944 C 3 0 0 0 0 0 0 0 0 0 0 0

PTK6 -0.964443849120419 1.23176517054376e-13 6.62318732468230e-12 C 78 15 6 2 0 0 3 3 6 0 1 0

PRRG3 -0.964575752976325 7.17046576676077e-13 3.23131918017812e-11 C 1 0 0 0 0 0 0 0 0 0 0 0

S100A14 -0.969147291275563 2.95694647157508e-08 4.30177754554201e-07 MN 2 2 0 0 0 0 0 1 1 0 0 0

MPPED1 -0.969431610182186 5.74593915964645e-08 7.81225595581881e-07 NA

FAM198A -0.970409138734795 7.83548980544697e-11 2.14930892098108e-09 NA

COL6A5 -0.971803585555719 4.78981045714462e-07 5.00865980786609e-06 NA

RIC3 -0.972428165747243 1.51497755908855e-17 2.18885345502817e-15 NA

LOC100291323 -0.973803634731255 0.000652309431420004 0.00245403709172857 NA

LOC283143 -0.975681895023162 1.68863657876097e-09 3.40008771555413e-08 NA

PACRG -0.97665243525431 3.70314547563922e-17 4.76798873587915e-15 C 2 1 0 1 0 0 0 0 0 0 0 0

SHROOM4 -0.979192152296382 2.99807658118343e-11 9.0791352723294e-10 C 2 0 0 0 0 0 0 0 0 0 0 0

GGT8P -0.980526056515288 2.77487286976778e-11 8.51215863956156e-10 NA

KRT81 -0.980663262046451 8.85056184680734e-08 1.14736016489399e-06 C 38 2 2 0 0 0 0 1 0 0 0 0

LOC100292909 -0.981232840215665 5.63988426790864e-07 5.78569590995701e-06 NA

FBXO16 -0.982248062686834 1.40387546033539e-07 1.71870315897593e-06 NA

ZNF471 -0.983081534436022 8.29102243189433e-17 9.8694453816644e-15 C 1

ZNF433 -0.985480663004188 3.14399446606071e-07 3.48806466935118e-06 C 1 0 0 0 0 0 0 0 0 0 0 0

PDZRN4 -0.98560458983296 9.27066108661886e-13 4.05232799968672e-11 C 1

POU2F3 -0.985869341460906 1.15877721315976e-11 3.90252247570726e-10 C 4 0 0 0 0 0 0 0 0 0 0 0

LOC440356 -0.986817996266707 5.33636776761668e-11 1.50748407071165e-09 NA

STMN2 -0.986835955760563 1.97133013314977e-08 3.01627853113385e-07 C 8 0 0 0 0 0 0 0 0 0 0 0

RIMKLA -0.9880801677036 2.35540589047719e-11 7.43190884659483e-10 NA

KLK7 -0.98811063070832 0.015855402417717 0.035218307893455 MN 1 1 0 0 0 0 1 0 0 0 0 0

CLU -0.98927667438153 2.27438862399236e-06 1.94875578937825e-05 C 381 88 26 11 7 2 37 19 14 0 0 2

C6orf132 -0.989397489150399 5.17816744038704e-10 1.17514598494251e-08 NA

MXI1 -0.990108592123516 4.93966176004613e-15 3.91184008922147e-13 C 104 14 1 4 0 0 5 1 4 0 0 0

CSTA -0.991105583123612 7.28432016795922e-06 5.3229794633401e-05 C 21 0 0 0 0 0 0 0 0 0 0 0

LOC100505633 -0.991371586233993 0.000129836903761551 0.00063189073733476 NA

CXCR2P1 -0.99400413444955 7.60215717448669e-09 1.30568084248194e-07 NA

FIBIN -0.994020707377262 2.31308886117833e-05 0.000144966334024908 NA

HBA2 -0.99438348756382 7.72001411767986e-06 5.59180525334564e-05 C 21 1 1 0 0 0 0 0 0 0 0 0

C1orf210 -0.994946200190957 1.09886095830085e-12 4.69484003561179e-11 NA

MMP27 -0.996287348456594 6.83076065132585e-10 1.51922217212273e-08 C 2 0 0 0 0 0 0 0 0 0 0 0

PRSS12 -0.997556557000074 0.000248338403503725 0.00108979850756202 NA

CEACAM6 -0.998130507600339 0.000787767999964617 0.00287008372191151 C 116 17 5 2 6 0 3 5 1 0 0 0

TRIM29 -1.00011170639518 1.95200170424222e-12 8.04913643925763e-11 C 17 1 0 0 0 0 0 1 0 0 0 0

C15orf59 -1.001919189781 3.56140602924698e-11 1.04831620397446e-09 NA

GGT6 -1.00764552686445 8.97870672930461e-11 2.4311871568748e-09 NA

FLJ31485 -1.00935448386064 7.82886685677381e-14 4.46316153608909e-12 NA

CBR3 -1.01096394957073 2.12871156038946e-10 5.24610985722544e-09 C 10 0 0 0 0 0 0 0 0 0 0 0

TRPM1 -1.0132845964959 2.04999532692033e-13 1.04582915236176e-11 C 13 3 0 1 1 0 0 1 0 0 0 0

DUOX2 -1.01430295712796 4.66354417705924e-05 0.000262933871430444 C 17 0 0 0 0 0 0 0 0 0 0 0

ITM2A -1.01623249136842 2.40550004254245e-07 2.76100056429357e-06 C 1 0 0 0 0 0 0 0 0 0 0 0

RDH12 -1.01703179693692 0.00210941354570495 0.00651728210570642 C 5 0 0 0 0 0 0 0 0 0 0 0

CGNL1 -1.01843624624475 6.95366666478029e-08 9.26845415241525e-07 NA

FAM107A -1.01866365366766 5.61647008936228e-11 1.57719479794302e-09 C 3 1 0 0 0 0 0 0 1 0 0 0

DBNDD1 -1.01907450785654 2.39334673051011e-09 4.65558823929751e-08 NA

ABCG4 -1.01972030759557 1.02625595509109e-06 9.76077711658744e-06 NA

JAM2 -1.02013997821679 8.86015924815937e-09 1.49063319191033e-07 C 8 2 0 0 0 0 0 2 0 0 0 0

TTC22 -1.02157960135923 1.22210595700368e-11 4.07232384651561e-10 NA

GAN -1.0216573405468 6.4310089158539e-15 4.96815125511701e-13 C 18 0 0 0 0 0 0 0 0 0 0 0

ATP13A5 -1.02207041300159 1.82836501446338e-10 4.5774424112101e-09 NA

CX3CR1 -1.02246523359741 2.85893863424689e-08 4.17615157966003e-07 C 46 4 1 0 0 1 0 3 0 0 0 0

ANKRD20A2 -1.02427155331827 4.8247293562368e-12 1.77660802578782e-10 NA

LOC283278 -1.02574954026884 2.87985274732106e-12 1.12385511234115e-10 NA

ENPP5 -1.02576262603975 4.49921912801389e-13 2.14500555254204e-11 C 1 0 0 0 0 0 0 0 0 0 0 0

CAMK1D -1.02589731248226 1.11262558922363e-12 4.74294246108912e-11 C 2 1 1 0 0 0 0 1 1 0 0 0

NOS1 -1.0259863355036 3.36283717589352e-11 9.99190254758818e-10 MN 30 1 0 0 0 0 1 0 0 0 0 0

RAB25 -1.02612316677621 4.04946308007097e-06 3.22169767618761e-05 MN 1 1 0 0 0 0 0 1 0 0 0 0

HBA1 -1.02854732234786 6.15475919032918e-06 4.61350998793506e-05 MN 50 1 0 0 0 0 0 1 0 0 0 0

ZNF425 -1.0292383226961 1.67824378361939e-16 1.85755088260609e-14 NA

HSPA1A -1.03062216074576 1.30866455049805e-06 1.20005300132154e-05 C 132 24 2 1 1 0 16 2 3 0 0 0

ERC2 -1.03116757267185 2.12925702377302e-13 1.08334536798258e-11 NA

PCSK2 -1.03319411213923 1.25687136844008e-11 4.16616539237572e-10 C 34 1 0 0 0 0 0 0 1 0 0 0

SYBU -1.03551814777838 1.58291896837809e-06 1.41721415867985e-05 NA

ZIM2 -1.03629248800407 7.69206368626361e-10 1.69485086600595e-08 C 1

NR3C2 -1.03648749285853 8.1757013609498e-10 1.78479238360665e-08 C 260 4 2 0 0 0 0 1 2 0 0 0

LPHN3 -1.03701649203665 1.97993463670085e-07 2.32470365191296e-06 C 1 1 0 0 0 0 0 1 0 0 0 0

IKZF2 -1.03705167730783 1.28120215864224e-09 2.64153739178885e-08 C 27 2 0 0 0 0 0 1 1 0 0 0

SFTPD -1.03766097045607 1.96307681954132e-09 3.89059214277052e-08 C 36 3 0 0 1 0 0 0 0 0 0 2

MAP3K1 -1.03795871388273 2.56729072037184e-19 5.1692671770721e-17 C 87 11 1 0 0 1 4 3 1 0 0 1

LRRC20 -1.04449393257374 1.23245361093114e-14 8.86944847684171e-13 NA

GDF10 -1.04553766489342 1.585006034837e-15 1.41506647270565e-13 C 155 10 4 0 1 0 6 1 2 0 0 0

ST6GALNAC1 -1.0493850985817 1.22869840965981e-06 1.13886262485951e-05 C 8 1 0 0 0 0 0 1 0 0 0 0

ANKRD20A5P -1.05163015993823 3.6935843269022e-11 1.08385225667097e-09 NA

ARHGEF26-AS1 -1.05195693593042 8.60579263819166e-13 3.77057086844382e-11 NA

LRRC16B -1.05523760454196 3.5141520800447e-15 2.90446971262035e-13 C 1

SLC16A7 -1.05761510678908 7.19380302063538e-07 7.13611686433783e-06 C 5 1 0 1 0 0 0 0 0 0 0 0

SPATA18 -1.05789575128121 1.66137603333619e-11 5.3843945518757e-10 NA

PBOV1 -1.06078247333917 6.06198946390087e-11 1.69726737549189e-09 C 2 1 0 0 1 0 0 0 0 0 0 0

NUAK2 -1.06126141925564 2.84902104582305e-17 3.84434091080947e-15 C 7 1 1 0 0 0 0 0 0 0 0 0

DLX3 -1.06218105152376 3.17682972468091e-12 1.22211089835438e-10 C 12 0 0 0 0 0 0 0 0 0 0 0

XKR4 -1.06220731920677 2.17047187317233e-12 8.7966854697072e-11 NA

GGTA1P -1.06618668793407 1.34376081910116e-10 3.50804979629348e-09 C 3

LOC388152 -1.06717956483675 3.1818441725679e-17 4.21138214364984e-15 NA

LOC100128398 -1.06731954248893 2.22454995124996e-11 7.06443908176308e-10 NA

PRSS3 -1.06766338280152 0.00165029651977968 0.00528782160654648 C 24 2 1 0 0 0 0 2 0 0 0 0

STOX2 -1.06851018630265 4.0104706142463e-14 2.44071309697234e-12 NA

SYNGR1 -1.0708514926869 3.75399390656271e-11 1.09987372553425e-09 C 1

TCP11L2 -1.0712507640058 2.53243892484917e-11 7.92255727778847e-10 NA

MIR600HG -1.07188102736504 2.20299211678719e-13 1.11486716027891e-11 NA

VSIG10 -1.07240550920099 7.27304022407915e-13 3.26975848743815e-11 NA

ELOVL4 -1.07533900367259 1.88762954368469e-05 0.000123027425527962 C 2 0 0 0 0 0 0 0 0 0 0 0

TPPP3 -1.07862839495284 2.40845108193834e-07 2.76271234108164e-06 C 30 2 0 0 0 0 2 0 0 0 0 0

OCLN -1.08015270376756 9.66503154071203e-07 9.28107823293032e-06 C 90 10 1 0 0 0 1 9 0 0 0 0

GSTA2 -1.08100986235583 9.65010881583975e-08 1.23912896578968e-06 C 6 0 0 0 0 0 0 0 0 0 0 0

NFIX -1.08434683854526 3.81691950231198e-18 6.12227418815753e-16 C 43 2 2 0 0 0 0 2 0 0 0 0

PCP4L1 -1.08477501494539 0.000190299389169097 0.000872951172758967 NA

KLK1 -1.08567964857669 5.74137709395341e-05 0.000315159641117332 C 123 23 5 6 2 1 5 10 2 0 0 0

CSTB -1.08624460983377 5.72628268669624e-14 3.34033255123778e-12 MN 2 1 0 0 0 0 0 0 1 0 0 0

GSTA4 -1.08729275938779 3.12298593741756e-08 4.51556568659298e-07 C 8 0 0 0 0 0 0 0 0 0 0 0

P2RY2 -1.0884711183602 3.922624980494e-14 2.39495235502613e-12 C 33 10 1 0 0 0 3 1 7 0 0 1

PHGDH -1.08889805966426 8.56590400870827e-07 8.33128803560233e-06 NA

RUNDC3B -1.08897212042761 1.96716432769975e-10 4.89257808546297e-09 C 2 0 0 0 0 0 0 0 0 0 0 0

SEPT5 -1.08941142306091 1.23748732900473e-13 6.63510557395821e-12 C 2 0 0 0 0 0 0 0 0 0 0 0

CCL15 -1.09048281084519 2.39245930438346e-14 1.55608512900569e-12 C 64 4 1 0 0 0 0 3 0 0 0 0

PBX1 -1.09182914749394 2.08385702190941e-11 6.66235842122963e-10 C 68 4 0 0 0 1 0 1 1 0 1 0

LRRTM1 -1.0944553932613 1.30145879255002e-15 1.18426493108626e-13 NA

RBM20 -1.09467091753338 3.2218548815524e-17 4.23472550994044e-15 NA

SOST -1.09470813052694 4.0198286615413e-05 0.000231185952831942 C 28 3 1 2 0 0 0 1 0 0 0 0

PTGDS -1.09547889863263 4.78949234668944e-10 1.0948154788139e-08 C 153 20 4 0 1 0 3 6 8 0 0 0

CYB561D1 -1.09650019306867 2.01089211576769e-12 8.2381288041418e-11 NA

ANGPTL7 -1.09659072551483 7.24160146278676e-11 2.00382735213691e-09 C 4 0 0 0 0 0 0 0 0 0 0 0

KRBOX1 -1.09674855260824 6.64534232876813e-12 2.35536318832574e-10 NA

SCUBE2 -1.09830804190497 1.59929981499252e-07 1.92239140725052e-06 C 2 0 0 0 0 0 0 0 0 0 0 0

CGREF1 -1.09843163114186 2.80867329015814e-07 3.17371697688497e-06 NA

LINC00478 -1.09998297968255 1.94623516138474e-06 1.70381095742502e-05 NA

TTC9 -1.10037982708764 9.43418265680134e-11 2.53277695241531e-09 C 2 0 0 0 0 0 0 0 0 0 0 0

FAM63A -1.10696452606375 7.25100021799885e-14 4.14621393130104e-12 NA

ZNF750 -1.10801036329598 4.05902886675596e-06 3.22659552125536e-05 NA

HES5 -1.1091796609373 9.48561800520499e-08 1.22132171418037e-06 C 16 0 0 0 0 0 0 0 0 0 0 0

EMP2 -1.109814249711 6.94974039129113e-15 5.34706245471411e-13 C 15 0 0 0 0 0 0 0 0 0 0 0

SOX14 -1.11181360767529 1.57992297817517e-20 3.98709362772287e-18 C 1

FUT6 -1.11345884591706 1.59094674792466e-18 2.76255496311651e-16 C 10 1 0 0 0 0 0 0 1 0 0 0

FLG -1.1141095708011 0.000640757619547613 0.00241586045123061 C 63 0 0 0 0 0 0 0 0 0 0 0

LONRF1 -1.11810292260015 1.73057428663442e-14 1.16980641154035e-12 NA

GRHL3 -1.12441185736902 1.6714591897901e-10 4.2407115395653e-09 C 35 0 0 0 0 0 0 0 0 0 0 0

WNK4 -1.12494227319775 9.73773683038943e-13 4.22720516029314e-11 C 3 0 0 0 0 0 0 0 0 0 0 0

SLC7A2 -1.12550047345429 2.79144440340777e-09 5.35295524045581e-08 C 3 0 0 0 0 0 0 0 0 0 0 0

KCNK10 -1.12681128316141 2.32031832657693e-08 3.49933585395391e-07 NA

USH1G -1.12773833630299 1.09061029804616e-13 5.89770888889134e-12 C 1 0 0 0 0 0 0 0 0 0 0 0

BLNK -1.12895377586816 1.19366336545498e-11 3.99840637191578e-10 NA

GATM -1.12930156722145 5.80648550929862e-09 1.02518051524715e-07 C 13 0 0 0 0 0 0 0 0 0 0 0

LYPD3 -1.12941724342828 1.58612404422149e-07 1.91213820286497e-06 C 21 13 2 5 1 0 0 9 0 0 0 0

EYA2 -1.13117687596491 5.36480408925943e-09 9.59732013208064e-08 C 10 1 0 0 0 0 0 1 0 0 0 0

CD1A -1.1323615946473 2.52149712604494e-07 2.87669536495796e-06 C 464 19 2 4 5 0 0 5 5 0 0 1

CYP2C19 -1.13544681736662 1.9953787930821e-12 8.20529231550846e-11 C 22 1 0 0 0 1 0 0 0 0 0 0

MEIS1 -1.13560748475288 1.11567989357359e-14 8.18467959134395e-13 C 63 9 4 1 1 0 3 0 4 0 0 0

ZDHHC11 -1.13712616728146 2.05172467065577e-07 2.40006136226835e-06 C 2

TGFBR3 -1.1371848810466 2.13103459785938e-09 4.18889300688139e-08 C 43 3 0 0 0 0 0 2 1 0 0 0

SPRR2D -1.14027825795177 0.00275512545899491 0.00810605620432094 NA

C4orf26 -1.14074062003649 1.23257454030104e-05 8.40682462136133e-05 NA

DDIT4L -1.14122399892688 2.11119520875993e-06 1.82793191748395e-05 C 2 0 0 0 0 0 0 0 0 0 0 0

CILP -1.14190773801589 4.28211655954802e-07 4.56092403616012e-06 NA

GDA -1.14367404411538 9.55410042528922e-06 6.74237355516215e-05 C 31 2 1 0 0 0 1 0 0 0 0 0

AGSK1 -1.14387913368594 1.43227951854896e-15 1.2908930689322e-13 C 3 1 0 0 1 0 0 0 0 0 0 0

ANKRD6 -1.14444436348714 8.93930602269514e-15 6.74080657735262e-13 C 2 0 0 0 0 0 0 0 0 0 0 0

HOMER2 -1.14537208266341 1.19437690148357e-05 8.18167629908776e-05 C 61 0 0 0 0 0 0 0 0 0 0 0

WISP2 -1.14791218536633 1.22192644743136e-09 2.53312178209565e-08 C 36 9 2 1 0 0 0 4 4 0 0 0

ATP4A -1.14804874255937 9.34074338783352e-10 2.00900284206278e-08 NA

ROBO2 -1.14847406966689 2.74293564052801e-19 5.46479398613407e-17 C 3 0 0 0 0 0 0 0 0 0 0 0

CLN8 -1.15079769495039 5.17385709913118e-16 4.99620374057427e-14 C 8 0 0 0 0 0 0 0 0 0 0 0

LRRK2 -1.15082200723516 3.08894338657551e-12 1.1931516628105e-10 C 5 0 0 0 0 0 0 0 0 0 0 0

MGLL -1.15426163984218 3.8342713142145e-13 1.83724691554779e-11 C 27 3 0 0 0 0 1 0 3 0 0 0

CEACAM1 -1.15519623132041 2.87814487048827e-07 3.24060963496321e-06 MN 2 2 0 1 0 1 0 0 0 0 0 0

ITGBL1 -1.15548559641136 2.13068978349072e-11 6.78915244648633e-10 NA

CHRNA3 -1.15698190711061 6.83776303871305e-08 9.13326330513211e-07 C 9 1 0 1 1 0 0 0 0 0 0 0

RSPO1 -1.1618097236707 1.38836019727593e-16 1.57281323228686e-14 C 4 1 1 0 0 0 0 1 0 0 0 0

ART4 -1.16490336785587 2.44087284138437e-07 2.79482155286643e-06 C 9 3 1 0 0 0 0 1 2 0 0 0

ECHDC3 -1.165078534194 1.43780957888991e-09 2.93260691537987e-08 NA

BEX2 -1.1655951884031 1.87974847380156e-06 1.65248487522722e-05 C 15 6 1 0 0 1 4 2 1 0 0 0

SIAH3 -1.16590908327462 2.47878691816389e-16 2.54978260870043e-14 C 1 0 0 0 0 0 0 0 0 0 0 0

CRYAB -1.16613423811424 1.06159657279447e-09 2.24250427826796e-08 C 17 1 0 0 0 0 0 1 0 0 0 0

KRT222 -1.16763235666369 3.84047509829764e-11 1.11918925683145e-09 C 1

CAPNS2 -1.17029590580832 2.53471886470523e-06 2.13600284738539e-05 NA

ACADSB -1.17287675385818 1.3556340721238e-11 4.48567938515511e-10 C 1 0 0 0 0 0 0 0 0 0 0 0

CCL19 -1.1742089773848 8.52398619911204e-05 0.000443467528286403 C 87 7 0 1 0 0 2 3 3 0 0 0

GIPC2 -1.17502082619943 1.06704801576447e-09 2.25150700048765e-08 C 5 0 0 0 0 0 0 0 0 0 0 0

CYP2R1 -1.17778477357794 4.12952495486157e-07 4.42078726361227e-06 C 11 0 0 0 0 0 0 0 0 0 0 0

PLBD1 -1.17825344154825 4.13396630150113e-06 3.28340663820864e-05 NA

UPK1A -1.17959639746633 5.27169293600229e-07 5.45827856672404e-06 C 9 1 0 1 0 0 0 1 0 0 0 0

AIF1L -1.18608181946016 2.74634335529573e-06 2.29289989791276e-05 NA

KRT32 -1.18891902709869 9.58512795063306e-17 1.11986244889896e-14 C 8 0 0 0 0 0 0 0 0 0 0 0

ZNF711 -1.19022109520407 5.81303288726812e-12 2.09568139918712e-10 NA

LY6D -1.19342198149757 0.000110237594897781 0.000550373769092665 C 32 9 5 0 1 0 0 5 0 0 0 0

EMP1 -1.19751440295894 4.47157528051466e-16 4.38515571680316e-14 MN 2 1 0 0 0 0 0 1 0 0 0 0

DPT -1.19769785752997 1.16513677549148e-06 1.08579732888859e-05 NA

CYP2J2 -1.1980566671606 1.72376998557708e-08 2.67205524299896e-07 C 15 1 0 0 0 0 1 0 0 0 0 0

APOD -1.20082126840777 9.23466745519593e-09 1.54539832824486e-07 C 34 10 3 2 0 0 0 3 3 0 0 0

ATP10B -1.20454620195336 1.46471180637682e-09 2.98092476981656e-08 C 1 0 0 0 0 0 0 0 0 0 0 0

SASH1 -1.20499171426796 1.55154730429095e-15 1.39175999186327e-13 C 9 5 0 1 0 0 2 4 2 0 0 0

PDGFD -1.20592745072784 1.31718377882176e-09 2.70981928062602e-08 C 42 5 3 0 0 0 0 1 1 0 0 0

C14orf132 -1.20643724463217 2.32773425486432e-15 1.99353059917724e-13 NA

LUZP2 -1.20890602700524 9.60939649088961e-12 3.3128788230067e-10 C 2

NDRG4 -1.20967957638349 5.74698406987966e-08 7.81225595581881e-07 C 9 0 0 0 0 0 0 0 0 0 0 0

VWC2 -1.21256320872093 1.76081649572479e-16 1.92641467136318e-14 NA

VLDLR -1.21390841465323 1.87913287350594e-08 2.88922403711186e-07 C 12 4 1 0 0 1 0 1 1 0 0 0

ARSG -1.21521974857458 4.56363658140079e-18 7.16184045151593e-16 C 6 0 0 0 0 0 0 0 0 0 0 0

CDHR1 -1.21885740945421 7.33438577073016e-08 9.71434006176416e-07 C 2

EPHB6 -1.21921590912715 3.15699834886212e-14 1.97855985923554e-12 MN NA

LYPD2 -1.22158669464409 6.33489321474616e-05 0.000343357743056989 NA

TNFAIP8L3 -1.22489242625979 8.21328202349104e-15 6.24308388990421e-13 NA

CYP2C9 -1.22586508378911 1.70718801756744e-07 2.03860868192422e-06 C 26 3 1 0 0 2 0 0 1 0 0 0

TSPAN8 -1.22677027661477 3.04453895761618e-06 2.51633139086469e-05 C 28 11 0 1 0 1 0 9 1 0 0 0

SOSTDC1 -1.22727439574692 2.26378043458178e-06 1.94227435563596e-05 C 5 3 0 0 0 1 0 1 1 0 0 0

RBP7 -1.22928995375531 1.59653284615699e-07 1.92101571387244e-06 C 2

LRRC4 -1.22929897583261 2.70198028090118e-08 3.9891092649467e-07 C 15 4 0 0 0 0 0 2 2 0 0 0

MEGF9 -1.2317982863729 4.74051473223365e-17 5.90287646953857e-15 NA

CAPN14 -1.23423039492177 0.000269388095225625 0.00116885204306988 NA

CAPN6 -1.239686558088 6.20499461745343e-10 1.38867249313947e-08 C 3 2 2 0 0 1 2 0 1 0 0 0

AR -1.24346997263729 1.6356252219703e-13 8.50480180665712e-12 MN 31 5 1 0 3 0 0 3 1 0 0 0

KLHDC8A -1.24430783384051 1.39824968958744e-14 9.76556157742493e-13 NA

MPZL3 -1.24533671765946 4.08608973707255e-10 9.51259784176779e-09 NA

CA13 -1.24719093737652 4.8539094282883e-12 1.78388240289733e-10 C 3 0 0 0 0 0 0 0 0 0 0 0

SLC24A3 -1.25060092222501 3.79158679236359e-12 1.43526726438131e-10 C 3 0 0 0 0 0 0 0 0 0 0 0

PIK3C2G -1.25345693597689 2.16999225494525e-09 4.25610812532111e-08 C 4

MAF -1.25599198441014 5.09060197450292e-15 4.01457598214236e-13 C 101 4 1 0 1 2 0 0 1 0 0 0

TSPAN7 -1.25757496022594 1.7985382425339e-05 0.000117911095657912 C 24 3 1 1 2 0 0 0 0 0 0 0

RORB -1.25800920277261 4.17248292900419e-18 6.63635163002204e-16 C 1 0 0 0 0 0 0 0 0 0 0 0

KLF8 -1.26240032766508 2.84625469091409e-08 4.16444386602581e-07 C 19 0 0 0 0 0 0 0 0 0 0 0

LOC727849 -1.26417421747437 1.05521259210654e-15 9.69514986932066e-14 NA

PDE6A -1.26436360105965 1.69634959741014e-15 1.50031816963466e-13 C 1 0 0 0 0 0 0 0 0 0 0 0

CD24 -1.26487569418723 8.53964016134365e-10 1.85781344061783e-08 MN 4 1 0 1 0 0 0 0 0 0 0 0

MYRIP -1.26596048598326 2.75474997116317e-11 8.47450499764213e-10 C 2

SDR9C7 -1.26945154690485 1.74790270709211e-05 0.000115029744565829 C 2 1 1 1 0 0 0 1 0 0 0 0

BEX4 -1.27066928866167 3.57380618572537e-17 4.6329746354263e-15 C 2 0 0 0 0 0 0 0 0 0 0 0

KLF4 -1.27161021835847 2.78357238935747e-10 6.71998400680726e-09 C 170 18 3 3 0 1 5 6 6 0 0 1

PADI1 -1.27191835547572 2.78839968443712e-12 1.09267165273999e-10 C 64 2 0 0 0 0 2 0 0 0 0 0

XK -1.27197485546823 1.1792303361332e-12 5.01557136449284e-11 C 6

GULP1 -1.27248491157543 2.43612986065233e-17 3.34120506322946e-15 C 3 1 0 0 0 0 0 0 1 0 0 0

VIT -1.27476955393648 1.08696234799113e-08 1.77812760245705e-07 C 45 2 0 0 0 0 0 1 2 0 0 0

ECM1 -1.27731742467049 3.20439388921884e-09 6.0771105351949e-08 C 28 6 1 0 1 1 0 3 2 0 0 0

FUT5 -1.27909591581423 2.17980254961842e-13 1.10608908462809e-11 C 3 0 0 0 0 0 0 0 0 0 0 0

SFTA2 -1.28304399457023 4.22313356855213e-14 2.53749996990432e-12 C 1 0 0 0 0 0 0 0 0 0 0 0

FABP12 -1.28605301969064 6.12223609625363e-12 2.19046432124371e-10 NA

BCL2L10 -1.28667965369149 1.5146166625459e-05 0.000101190079675278 C 25 4 2 0 0 0 3 0 1 0 0 0

ELMOD1 -1.29379671450466 5.06054772452439e-07 5.26269158143259e-06 NA

SULT2B1 -1.29916107564519 2.18607690610716e-06 1.88316673883856e-05 C 12 0 0 0 0 0 0 0 0 0 0 0

FZD10 -1.30118211992429 2.46726097987565e-12 9.74902892820592e-11 C 11 1 1 0 0 0 0 1 0 0 0 0

SLC9A3R1 -1.30199027022197 1.19688980480188e-18 2.1574793652843e-16 C 34 6 1 2 0 0 1 1 1 0 0 0

AQP3 -1.305260791243 7.68782419429294e-07 7.57062687436954e-06 MN 2 1 0 0 0 0 1 0 0 0 0 0

OXGR1 -1.30657486090772 4.57996795443861e-13 2.16712633684149e-11 C 5 0 0 0 0 0 0 0 0 0 0 0

SMO -1.31240415480443 2.32866670856888e-12 9.3181130640768e-11 C 202 20 10 0 1 0 6 0 9 0 0 0

BMP7 -1.3125535377509 1.07184771116705e-11 3.66851024037228e-10 C 97 13 4 3 0 0 1 11 3 0 0 0

FCER1A -1.31390911192965 9.13330347291981e-08 1.18158602072422e-06 MN 184 1 1 0 0 0 0 0 0 0 0 0

TGM1 -1.3217218953787 7.03160616260094e-06 5.16242086266672e-05 C 75 13 1 0 0 0 7 3 3 0 0 0

CLIC3 -1.32214724982492 3.69312231549911e-08 5.25957306737785e-07 C 3 1 0 1 0 0 0 0 0 0 0 0

MFSD6L -1.32263500873117 1.26382731750658e-09 2.61140389066016e-08 NA

CACNB4 -1.32467069184715 3.12093136250942e-15 2.60219682370995e-13 C 10 0 0 0 0 0 0 0 0 0 0 0

CYSLTR1 -1.33216715388541 4.58966965386736e-09 8.33672529162644e-08 C 14 2 1 0 0 0 2 0 1 0 0 0

LCE3A -1.33319863755737 2.03462651724752e-05 0.000130667793306783 NA

TEX101 -1.33479823194108 8.21544894545891e-13 3.62456415362939e-11 C 4 4 1 1 1 0 0 3 0 0 0 0

CNFN -1.34113972590005 5.48994490695963e-06 4.18647007469883e-05 NA

TRPS1 -1.34571137127665 6.93328383634598e-17 8.35836071149811e-15 C 8 2 0 0 0 0 2 0 0 0 0 0

ABLIM1 -1.34670261147571 9.96110465682563e-21 2.58265517588683e-18 C 2

AQP1 -1.34847352146121 3.48720869824967e-10 8.2297255650588e-09 C 69 10 3 1 0 5 0 3 0 0 0 0

CHRDL1 -1.3502574783261 1.49241743491796e-09 3.03079235951633e-08 NA

ANGPTL1 -1.35560376881923 3.35578893307101e-09 6.30735026179097e-08 C 14 3 1 0 0 2 1 1 2 0 0 0

EVPLL -1.35713302002372 2.50245097584332e-08 3.73924095482551e-07 NA

PRSS35 -1.35783196205884 1.93167542282928e-13 9.90808149807313e-12 NA

SERPINB12 -1.35866769014602 2.14463311022129e-07 2.49486606497593e-06 MN 1

HRASLS -1.36257898973991 9.9460108959824e-09 1.64985230699613e-07 C 141 1 0 0 0 0 0 1 0 0 0 0

NYNRIN -1.3636335538356 4.65517987416186e-13 2.18631735678068e-11 C 1 0 0 0 0 0 0 0 0 0 0 0

CD207 -1.36412917642117 1.27329145025532e-09 2.62809021581052e-08 C 11 0 0 0 0 0 0 0 0 0 0 0

PLA2G3 -1.36488535735627 2.41256319265085e-06 2.05040788268085e-05 C 2

SLURP1 -1.36672503707721 2.53234875458084e-05 0.000156377699438668 MN 5 1 0 1 0 0 0 1 0 0 0 0

PHYHD1 -1.36687549125459 1.77205419375308e-14 1.17683051667244e-12 NA

SPRR2E -1.36989894517333 4.89394527137985e-05 0.000273560845101614 NA

LGI3 -1.37858526145931 4.98342182313606e-24 2.77415367195577e-21 NA

EHF -1.37987968615777 6.16771199443548e-11 1.72044217373433e-09 C 11 2 2 0 1 0 0 0 0 0 0 0

VSIG10L -1.38145281360607 1.56891394500208e-14 1.07981215407471e-12 C 1 0 0 0 0 0 0 0 0 0 0 0

SUSD4 -1.39042089466859 1.11936133896605e-09 2.34101127763651e-08 C 1

RORA -1.39118408204374 1.03700588352316e-12 4.45065994499836e-11 C 55 9 5 1 0 0 1 3 3 0 0 0

STAC2 -1.39193384803068 2.64526981134612e-14 1.69718717692705e-12 NA

GSTA1 -1.39254227027791 2.94262365146038e-10 7.07687901539907e-09 C 13 1 1 0 0 0 0 0 0 0 0 0

KRT15 -1.3949832931509 6.91438488544515e-11 1.91889388162493e-09 C 38 3 0 0 1 1 0 0 1 0 0 0

DYNLT3 -1.39783325941335 1.03335760841779e-08 1.70072690908901e-07 C 7 0 0 0 0 0 0 0 0 0 0 0

TFAP2B -1.4008611757334 4.0565380320878e-09 7.49054588617813e-08 C 10 1 0 0 0 1 0 0 0 0 0 0

PRLR -1.40430716404043 5.50874979206062e-20 1.25619406402809e-17 C 123 12 6 1 0 0 1 2 4 0 0 0

DEPTOR -1.40746519150887 1.44045919058582e-11 4.73325887156558e-10 C 17 2 0 0 0 0 1 0 1 0 0 0

ABCA8 -1.41215203640079 9.46734030666055e-13 4.12876382451991e-11 C 2 0 0 0 0 0 0 0 0 0 0 0

LDOC1 -1.41461460618355 2.90009215723305e-12 1.12710563162115e-10 C 6 1 0 0 0 0 1 0 0 0 0 0

PPM1L -1.41602793109953 1.60832962938521e-14 1.10292952519471e-12 C 1

SERPINB13 -1.41626479978417 2.51284982797117e-06 2.11946117174734e-05 C 8 0 0 0 0 0 0 0 0 0 0 0

FADS6 -1.42177538061396 1.165767870916e-14 8.47045011372798e-13 NA

BARX2 -1.42981182740642 2.07437943923593e-11 6.64327912798959e-10 C 4 3 1 0 0 0 0 2 1 0 0 0

SLC16A14 -1.43419593009465 8.886225682163e-18 1.35636768940564e-15 C 1

SPRR2C -1.44051077394805 0.000143092505668487 0.000685301582689132 C 1 0 0 0 0 0 0 0 0 0 0 0

LOC643669 -1.44702393242755 3.59875976705534e-10 8.46133243615607e-09 NA

ANKRD31 -1.45252549035222 7.13191006317429e-15 5.46500654921862e-13 NA

EPHX2 -1.45830500701737 2.86004821822335e-16 2.91032971109211e-14 C 23 4 0 0 1 0 0 1 2 0 0 0

RRAGD -1.46051723336113 2.5402424368952e-11 7.93385620513455e-10 C 2 0 0 0 0 0 0 0 0 0 0 0

MPP7 -1.47474461123348 1.34119341962716e-22 5.90343438448446e-20 NA

RAPGEFL1 -1.4749189575341 7.22405805426729e-12 2.53672999616173e-10 NA

ADRA2A -1.47549021868358 8.45966576586857e-14 4.75121940506215e-12 C 4 0 0 0 0 0 0 0 0 0 0 0

TM7SF2 -1.47618939943954 1.75039506819634e-14 1.17481303034582e-12 C 56 7 0 0 0 4 0 2 1 0 0 0

CLDN7 -1.47853792088663 2.72298481235786e-10 6.60742737737143e-09 MN 7 4 0 2 0 0 0 3 0 0 0 0

GSTA3 -1.48974457000675 2.75180569525475e-10 6.66028470512617e-09 C 2 1 0 0 0 0 0 1 0 0 0 0

FAM189A2 -1.49147473433354 1.5933221189772e-19 3.35075641620906e-17 NA

ZNF185 -1.49447332155561 5.92252644929271e-12 2.12705233597274e-10 C 2 1 0 0 0 0 0 1 0 0 0 0

FAM46B -1.49448570173288 2.00069063584964e-10 4.96943197699817e-09 NA

IL1RN -1.50065539914496 1.64281724816538e-13 8.5187950838428e-12 C 204 18 7 0 0 4 0 1 7 0 0 3

PGD -1.50367289333174 8.37473627493695e-14 4.71751885344439e-12 C 63 1 0 0 0 0 0 1 0 1 0 0

SLC5A1 -1.506577165208 2.48488866725384e-06 2.10055774029091e-05 C 13 2 1 1 0 0 1 0 0 0 0 0

ATP6V1C2 -1.50824568035895 2.83498030397117e-08 4.1562875455664e-07 NA

MAPT -1.51092029115089 7.72296460986021e-22 2.56443072229516e-19 C 309 37 9 5 4 0 17 4 2 0 0 0

FMO2 -1.51689115559478 2.39040148265755e-11 7.51546991067433e-10 C 2 0 0 0 0 0 0 0 0 0 0 0

COBL -1.53354532409552 6.3930297559137e-21 1.7042376646504e-18 C 1 0 0 0 0 0 0 0 0 0 0 0

GBP6 -1.53427927865326 2.69437537250115e-11 8.33275207113223e-10 NA

SULT1B1 -1.53429656171051 7.35971602703278e-11 2.0335378867686e-09 C 1

PTN -1.53731259236515 1.03934390199028e-13 5.65277644625576e-12 C 150 24 9 3 0 6 2 7 7 0 0 0

PITX1 -1.53765485683685 2.4245874733163e-17 3.34120506322946e-15 C 45 4 0 1 1 0 1 0 2 0 0 0

KRT19 -1.54109359613661 9.07809980926162e-05 0.000467540666911278 MN 44 3 0 0 2 0 0 1 0 0 0 0

COMP -1.54891613511402 5.39539434840188e-09 9.6429300124837e-08 NA

IGFBP5 -1.55259311495644 5.08753128916327e-12 1.85533149730238e-10 C 147 18 2 2 0 0 6 2 6 0 0 0

SOX21 -1.55479210643523 2.53378734509449e-18 4.20675377900029e-16 C 5 3 2 0 0 0 0 1 0 0 0 0

NPR3 -1.55730867703137 1.60071546169298e-13 8.36926561974117e-12 C 5 0 0 0 0 0 0 0 0 0 0 0

CPXM2 -1.55969834206748 5.19242902299858e-13 2.40875255191898e-11 C 1 0 0 0 0 0 0 0 0 0 0 0

GALNT5 -1.56170695847942 2.93080264942173e-07 3.29402029368201e-06 C 1

PAX9 -1.56190009345758 2.27303921519846e-09 4.43523847691353e-08 MN 1 1 0 0 0 0 0 0 1 0 0 0

COL14A1 -1.57179677372502 1.39935215884501e-09 2.86020932078397e-08 C 111 1 0 1 0 0 0 0 0 0 0

GSTA5 -1.5765100715912 1.54644406912621e-11 5.03778776185057e-10 C 3 0 0 0 0 0 0 0 0 0 0 0

DKK4 -1.58371249857469 2.30479428111549e-16 2.42400526834363e-14 C 16 3 0 0 0 0 0 2 1 0 0 0

IVL -1.58453612078278 8.20372881348775e-05 0.00042916521628768 C 231 9 0 1 6 0 0 3 1 0 0 0

P2RY1 -1.59005864299876 1.12554217457287e-07 1.4122373713648e-06 C 18 3 1 0 0 0 1 0 2 0 0 0

HBB -1.59053442699414 4.76398591113893e-08 6.5912252441613e-07 MN 6 1 0 0 0 0 0 1 0 0 0 0

BSPRY -1.59477354487014 1.19325258731782e-11 3.99840637191578e-10 C 1 0 0 0 0 0 0 0 0 0 0 0

LANCL3 -1.59908576897711 2.51723153607953e-15 2.13648615620526e-13 NA

FUT3 -1.60178501967164 2.29017455420662e-12 9.18350292107386e-11 C 56 5 1 0 0 0 0 5 1 0 0 0

CXCL12 -1.60356518516063 3.26507371566435e-09 6.16132105846252e-08 C 934 126 23 9 5 9 9 75 23 0 0 1

OTOP3 -1.60954149765969 1.08415073322631e-19 2.33178646906526e-17 NA

CORIN -1.6127515053732 1.27286033680371e-14 9.06233004321762e-13 C 14 0 0 0 0 0 0 0 0 0 0 0

CYP4F12 -1.61697677911196 2.26141131786184e-16 2.40459168613321e-14 C 1

NPPC -1.62670452731924 4.12030662939526e-20 9.87557656126292e-18 C 35 1 0 0 0 0 0 0 1 0 0 0

C6orf15 -1.62908030666777 4.72452689097172e-07 4.95461208741945e-06 NA

CYP3A4 -1.6362243853636 1.75097541647476e-11 5.6650789243791e-10 C 163 11 5 1 0 0 1 2 4 0 0 2

IGFBP2 -1.63716088626068 1.43558961743451e-08 2.26051619710342e-07 C 290 33 4 4 6 3 2 9 11 0 0 0

MANSC1 -1.64086222610462 1.88189640347717e-10 4.69903076894623e-09 C 1 0 0 0 0 0 0 0 0 0 0 0

SLC16A9 -1.64116630579798 2.99406639709708e-10 7.17325249339956e-09 C 1

SCEL -1.64792252943972 8.77178966915309e-08 1.13870825149561e-06 C 2 0 0 0 0 0 0 0 0 0 0 0

ARG1 -1.65340261835225 2.10176336206982e-06 1.82143201254101e-05 C 67 6 3 0 1 0 0 2 1 0 0 0

CXCL17 -1.65727010277771 7.46400698567979e-08 9.86531146773474e-07 C 41 3 0 0 0 2 0 0 1 0 0 0

EYA1 -1.65958686472982 1.89461763355469e-15 1.63741680138308e-13 NA

FETUB -1.66090910299583 1.86474054916929e-09 3.71124546520791e-08 C 2 1 0 0 0 0 1 0 0 0 0 0

BCMO1 -1.66444398254322 1.55083749308921e-20 3.96658124752696e-18 C 4 0 0 0 0 0 0 0 0 0 0 0

CD177 -1.66650621500151 6.10993384161709e-09 1.06977537299063e-07 NA

ROR1 -1.66901609806203 3.35610773842206e-16 3.34321321921654e-14 C 23 7 5 0 0 0 1 1 2 0 0 0

SLC16A6 -1.67060047797922 3.15714406008819e-22 1.14913972356325e-19 C 1 0 0 0 0 0 0 0 0 0 0 0

FIGF -1.67230692952294 1.11842542389164e-09 2.34101127763651e-08 MN 13 4 1 0 0 2 0 3 0 0 0 0

CYP4B1 -1.67960514488027 4.88685170286302e-08 6.73659447779231e-07 C 5 2 2 0 0 0 0 0 0 0 0 0

CCL14 -1.69855804412807 1.24572494089372e-11 4.14232483515492e-10 C 100 1 1 0 0 1 0 1 0 0 0 0

FRZB -1.71223210112643 1.77982405338962e-13 9.17894546553277e-12 C 126 3 0 0 0 0 1 3 1 0 0 0

PRSS27 -1.73347334759443 1.31843546051965e-10 3.44668894492478e-09 C 44 0 0 0 0 0 0 0 0 0 0 0

CRTAC1 -1.75077854361169 1.71706599234035e-26 1.36740196869852e-23 C 1

TMEM45B -1.75993453458495 0.000117564661772961 0.000582194231652758 C 1 0 0 0 0 0 0 0 0 0 0 0

PLP1 -1.76598372942976 2.43904410119837e-22 9.24538076635488e-20 C 65 2 0 1 0 0 0 0 1 0 0 0

FAM3D -1.76782594055719 9.44689107444932e-09 1.57534191511984e-07 C 1

MAP1LC3A -1.77857641850132 1.12367818101924e-28 1.18154760734173e-25 MN 12 2 0 0 0 0 2 0 0 0 0 0

SELE -1.78594388428705 2.04942094735632e-06 1.78260065581862e-05 C 506 86 9 7 5 8 0 64 2 0 0 3

TMPRSS11E -1.80069872968955 1.08429656781573e-05 7.51739235862574e-05 C 1 1 1 0 0 0 0 0 0 0 0 0

OMD -1.8021301294855 1.71201032468317e-08 2.65600159141627e-07 C 6

SH3GL3 -1.80700822317434 1.35750311171851e-14 9.55147263773097e-13 C 2 1 0 0 0 0 0 1 0 0 0 0

SCIN -1.80709773092428 1.03819990230061e-14 7.67578498079827e-13 C 3 0 0 0 0 0 0 0 0 0 0 0

LY6G6C -1.81995789705874 4.82937493797449e-11 1.37451999174501e-09 C 1 0 0 0 0 0 0 0 0 0 0 0

KLK11 -1.83033903663167 4.32910360659282e-08 6.06042484925904e-07 C 30 5 0 5 0 0 0 0 0 0 0 0

AADAC -1.8323143521389 2.2291829546273e-18 3.774274592742e-16 C 120 1 0 0 0 0 1 0 1 0 0 0

HPGD -1.83820712503417 7.51756941779293e-11 2.07110678850898e-09 C 74 0 0 0 0 0 0 0 0 0 0 0

PPL -1.84818976276634 5.94163207197093e-13 2.72216398853027e-11 C 17 1 1 0 0 0 0 0 0 0 0 0

HOPX -1.84953058661878 1.31011882698623e-12 5.53495960677865e-11 C 136 4 1 0 0 0 0 2 1 0 0 0

SH3BGRL2 -1.86986345237477 1.35262651592615e-16 1.54223867873098e-14 NA

ACER1 -1.87054630014081 1.87168188966124e-16 2.01280245031922e-14 NA

BPIFC -1.87101120309716 2.59005181682091e-08 3.85089636582635e-07 NA

SCNN1A -1.88090978964631 2.15149168757306e-12 8.73847278341099e-11 C 2 1 0 0 0 0 1 0 1 0 0 0

ANXA9 -1.89357726105854 2.72246949748297e-11 8.40590214989561e-10 C 1

CRCT1 -1.90988168659482 8.4223880160913e-07 8.21570687648953e-06 C 1 0 0 0 0 0 0 0 0 0 0 0

SELP -1.91701567258695 4.65803918101199e-09 8.44470379109328e-08 MN 5 1 0 0 0 0 0 1 0 0 0 0

TGM7 -1.91848542593321 4.76786466049928e-13 2.23369738686312e-11 C 37 0 0 0 0 0 0 0 0 0 0 0

ANKRD35 -1.92095858663723 5.77252466709266e-15 4.47772845795339e-13 NA

DSG1 -1.92351605265587 2.79628724966158e-08 4.10592154960006e-07 C 59 5 0 1 0 0 0 4 0 0 0 0

ATP6V0A4 -1.95201318710714 1.25982163618075e-09 2.60597203366044e-08 C 2

DNASE1L3 -1.95322799024652 4.82428407768402e-16 4.68252434555515e-14 C 25 1 0 0 0 0 1 0 0 0 0 0

RAET1E -1.96459891798966 9.08791486718059e-16 8.39058364346961e-14 C 7 1 0 0 0 0 1 0 0 0 0 0

OCA2 -1.96677625758408 2.03264510781208e-34 6.4119789925932e-31 NA

BNIPL -2.008357383689 1.76012381883473e-14 1.17683051667244e-12 C 4 1 0 0 0 0 0 1 0 0 0 0

MFAP4 -2.02928737210132 3.13330155884013e-10 7.45025108092551e-09 C 3

CYP4F22 -2.03355347195453 1.03058316955569e-12 4.43314719322287e-11 C 1

KRT24 -2.03428113055739 9.25427240825482e-07 8.93651091178771e-06 C 2 0 0 0 0 0 0 0 0 0 0 0

PITX2 -2.03433113422977 4.20109903339974e-31 6.62618345042974e-28 C 85 5 1 2 0 0 2 0 1 0 0 0

TGM5 -2.04323831337721 2.67282654345192e-13 1.33127863126091e-11 C 1 0 0 0 0 0 0 0 0 0 0 0

CEACAM5 -2.04406633777832 8.4389802107249e-08 1.10154881688545e-06 MN 34 1 0 0 0 0 0 1 0 0 0 0

KLK12 -2.05008584660038 1.66727589847471e-06 1.48152727372915e-05 C 4 1 0 1 0 0 0 0 0 0 0 0

EXPH5 -2.06350576186077 3.02503946166063e-21 8.41984145453686e-19 NA

C2orf54 -2.07760560227272 3.2677621468003e-13 1.60337942022001e-11 NA

FAM3B -2.08175988094383 5.88366199409936e-17 7.18452068143991e-15 C 4 1 0 0 0 0 1 0 0 0 0 0

ATP13A4 -2.08645036463321 6.10926964580587e-17 7.4121888837287e-15 NA

CHP2 -2.10782850416985 7.78317935969058e-10 1.70895865128612e-08 C 4 0 0 0 0 0 0 0 0 0 0 0

HTR3A -2.115658978337 3.29063278642304e-16 3.30983386461584e-14 C 1 0 0 0 0 0 0 0 0 0 0 0

AADACL2 -2.12680389590068 3.30511227565062e-16 3.30983386461584e-14 NA

SLC6A4 -2.13789344178131 7.14282244547233e-33 1.50213556028283e-29 NA

SLC27A6 -2.15495096695275 3.21372809017683e-30 4.05508210418513e-27 NA

BBOX1 -2.16145859104392 3.79648870782871e-15 3.0972474902187e-13 C 1

OGN -2.17916577590669 8.59306529101804e-17 1.01650591726937e-14 C 20 2 0 0 0 0 0 2 0 0 0 0

GPX3 -2.18495967033051 1.49302184582473e-22 6.27964988353881e-20 C 19 6 0 1 0 0 2 2 1 0 0 0

TMPRSS11D -2.21851602416845 3.92267811897774e-08 5.54891844229385e-07 NA

ELF5 -2.22270789850264 4.78741485477937e-16 4.67069077094893e-14 C 11 3 0 0 0 0 0 1 2 0 0 0

HLF -2.27864753586457 7.4502612333433e-22 2.51805525649087e-19 C 111 3 0 0 0 0 2 0 1 0 0 0

GUCY2C -2.33603024862338 2.89976997652217e-21 8.19163378293062e-19 C 182 9 5 1 2 0 0 4 2 0 0 0

MAOB -2.34226118618442 4.36223225633347e-20 1.01930827056325e-17 C 27 0 0 0 0 0 0 0 0 0 0 0

DACT2 -2.37332975402612 3.66232261943716e-15 3.01377305296031e-13 C 4 1 0 1 0 0 0 0 0 0 0 0

ALDH3A1 -2.39906391975284 5.39992124696221e-17 6.63664347021128e-15 C 22 3 2 0 1 0 0 1 0 0 0 0

NEBL -2.40147125000441 8.6036058970441e-16 7.98237494183106e-14 C 3 0 0 0 0 0 0 0 0 0 0 0

DCT -2.40879486157283 2.59126182165623e-32 4.90448124984874e-29 C 198 14 6 1 0 0 3 3 4 0 0 0

A2ML1 -2.44157653515568 3.08188504482754e-14 1.95086415529936e-12 C 1

CEACAM7 -2.49635601196027 9.38013307906892e-11 2.52184344868661e-09 MN 35 1 0 0 0 0 0 1 0 0 0 0

RHCG -2.4975810327694 1.81675656936657e-07 2.15719897041412e-06 C 3 0 0 0 0 0 0 0 0 0 0 0

CYP2C18 -2.54219853597482 1.02807574336554e-12 4.43243498739854e-11 C 9 2 1 0 0 1 0 0 0 0 0 0

MLANA -2.58718723832744 2.53467810434398e-28 2.39869262404593e-25 MN 12 1 0 0 1 0 0 0 0 0 0 0

POF1B -2.58953263589308 2.90921204403565e-10 7.0054270174889e-09 C 15 0 0 0 0 0 0 0 0 0 0 0

CRISP3 -2.62017963298658 4.97327500694487e-10 1.13272173353123e-08 C 8 0 0 0 0 0 0 0 0 0 0 0

CTTNBP2 -2.64630869774061 1.34012155669403e-33 3.62349724336399e-30 C 2 0 0 0 0 0 0 0 0 0 0 0

LAMB4 -2.66690508671135 2.79606224085917e-29 3.11300411957303e-26 C 1

RPTN -2.69636834652364 1.6852704306926e-10 4.27002857318859e-09 C 6 0 0 0 0 0 0 0 0 0 0 0

PPP1R3C -2.71094550921467 3.81412466555033e-17 4.87769848276156e-15 C 13 0 0 0 0 0 0 0 0 0 0 0

MUC22 -2.741180776725 2.53332608018199e-19 5.15572717415103e-17 NA

KLK13 -2.7451454314277 1.90404243369746e-11 6.11847387819895e-10 C 8 2 1 2 1 0 0 1 0 0 0 0

CHL1 -2.74734564393517 6.63555831190852e-20 1.47754367258226e-17 MN 1 0 1 0 0 0 0 0 0 0 0

KRT1 -2.77803209084653 5.73586385057404e-09 1.0155537427485e-07 C 91 2 0 0 0 0 1 1 1 0 0 0

TYR -2.80347148637074 3.02474123215374e-32 5.20447975463399e-29 C 1597 95 21 15 5 0 10 42 18 0 0 0

ODAM -2.88261288469489 8.69733316654377e-14 4.8558827387367e-12 C 7 1 1 1 1 0 0 0 0 0 0 0

HCG22 -2.93856847602065 2.9569660102104e-30 3.99760683394659e-27 NA

PMEL -2.94021970115986 5.22877397114581e-39 2.47412512379692e-35 C 886 16 6 2 2 0 1 5 2 0 0 0

GYS2 -2.94943014094768 4.33618291930561e-28 3.90813971969987e-25 C 1

KRT2 -2.95567319584141 1.72500026990058e-10 4.3532106811211e-09 C 4 0 0 0 0 0 0 0 0 0 0 0

SPINK5 -2.9610735200216 1.81869725537011e-12 7.53227198082933e-11 NA

CWH43 -2.97121058802285 1.32216342856767e-08 2.1029064884454e-07 NA

IL36A -3.04913372590631 5.43499686650747e-15 4.25075147489202e-13 NA

EPHX3 -3.06229407686585 1.20868611546561e-21 3.63123842974884e-19 C 6 1 0 1 0 0 0 0 0 0 0 0

SPRR3 -3.22966922415469 5.969439307426e-09 1.04905828943038e-07 C 8 1 0 0 0 0 0 0 1 0 0 0

TYRP1 -3.2402921269041 6.03754797413971e-30 7.1420419066589e-27 C 521 14 5 4 1 1 1 6 0 2 0 0

MUC15 -3.34962149900287 1.1452963737018e-17 1.73416195720432e-15 C 5 2 0 0 0 0 0 2 0 0 0 0

ALOX12 -3.38232594712071 6.45770589435806e-25 4.21465515387983e-22 C 24 0 0 0 0 0 0 0 0 0 0 0

ADH7 -3.45186671390821 3.769115075588e-14 2.33130853057693e-12 C 9 0 0 0 0 0 0 0 0 0 0 0

CLCA4 -3.47241051670182 6.43033268151172e-14 3.72192375116123e-12 C 15 2 0 0 1 0 0 1 1 0 0 0

XIST -3.49405902423673 1.00644766603696e-07 1.2801770816587e-06 C 10 3 2 0 1 0 0 0 0 0 0 0

KRT13 -3.57706094087026 5.39031866347501e-16 5.17881022048688e-14 MN 28 2 0 0 0 0 0 2 0 0 0 0

CLDN17 -3.6514750510105 1.25533862695926e-14 8.99992204259768e-13 C 2

SERPINB11 -3.66087547780389 1.36367968851513e-17 2.00080352438185e-15 C 1 1 0 0 1 0 0 0 0 0 0 0

KRT78 -3.69933021539535 1.14632210493941e-21 3.49942556132067e-19 C 1 0 0 0 0 0 0 0 0 0 0 0

LOR -3.80686593833357 3.77709049407256e-16 3.72338498860996e-14 C 32 0 0 0 0 0 0 0 0 0 0 0

SPINK7 -3.82896796753354 2.38589692900399e-21 6.94736479619363e-19 C 11 2 2 0 0 0 0 2 0 0 0 0

ENDOU -3.84235188213589 5.3034288906813e-26 3.71770365236759e-23 C 220 2 0 0 0 0 1 1 0 0 0 0

CRNN -3.86297625561296 2.30528318434963e-16 2.42400526834363e-14 C 10 0 0 0 0 0 0 0 0 0 0 0

TMPRSS11A -4.17218761645292 2.00050491777264e-17 2.80470789471724e-15 MN 2 1 1 0 0 0 0 0 0 0 0 0

KRT4 -4.78802657935764 4.55071848818954e-25 3.07612317235583e-22 C 36 1 0 0 0 0 0 1 0 0 0 0

TGM3 -4.80415596249301 1.75123902757465e-15 1.54166051511188e-13 C 17 2 0 0 0 0 0 2 0 0 0 0

MAL -4.89158993623382 1.73390644311114e-26 1.36740196869852e-23 C 115 9 2 1 3 0 1 2 0 0 0 0

KRT3 -5.95266022203564 7.1795095419909e-51 1.35886577101262e-46 C 11 0 0 0 0 0 0 0 0 0 0 0

TMPRSS11B -6.01736526520917 1.5668066778815e-24 8.98634848250399e-22 NA

KRT76 -6.97831600293701 4.00864888109902e-46 3.79358486862805e-42 C 5 0 0 0 0 0 0 0 0 0 0 0
