# Supplementary material for: Potential Therapeutic Targets for Oral Cancer: ADM, TP53, EGFR, LYN, CTLA4, SKIL, CTGF, CD70
Source: PLoS One. 2014 Jul 16;9(7):e102610. doi: 10.1371/journal.pone.0102610 (PMC4110113; doi:10.1371/journal.pone.0102610)
Supplement: Text S10 — Detailed report of all genes annotated by literature mining method. (DOCX) [file pone.0102610.s010.docx]

Literature Mining Statistics - All Annotations

----------------------------------------------

Total no. of genes annotated: 1014

Genes related with oral cancer : 199 EMP1,PFN2,CD44,CEACAM5,BDNF,SFRP4,NMI,AURKB,CDK1,SEMA3A,KRT19,AQP5,PTK2,CD34,IFNG,SLC6A2,XDH,VCL,TNFSF10,PDPN,CEACAM1,DCN,COL4A1,CD151,ADAM12,CD163,PLAU,PTHLH,CYP3A5,KRT20,IDO1,SAMSN1,TP53,ALCAM,CDK6,MAP1LC3A,TPX2,LIMA1,TLN1,MMP7,S100P,PXN,TLR2,MMP13,CHL1,RAB25,CRAT,PSMB8,HSPB2,PAG1,PAX9,CCNB1,NOP2,CDKN2A,IL23A,TGFB1,TACSTD2,MRPL28,VAV2,TNC,PKP1,TFRC,IL1A,LDHD,MMP3,BAK1,MET,ZYX,ICAM1,CXCL9,THBS1,CAV1,SERPINH1,JUN,KLK10,HOXA1,BIRC2,SOCS3,MSN,VEGFC,MAPK3,MLANA,MMP10,FCER1A,CD24,TXNRD1,FCGR3B,SELP,LAMC2,TIMP1,PHLDA1,NELL2,EPHB2,AR,SNAI2,S100A14,MTUS1,NRP2,FGFR2,SOX10,IL8,FAT1,MIA,EPS8,PLAUR,NME1,PI3,KIT,FGFR3,BUB1,BCL2L11,HLA-A,ATG5,ETS1,EPHA7,ABL2,APLN,CDC6,NOS1,PPARG,GALR1,CYR61,DSG2,GCNT1,CP,CSTB,TNFAIP3,ETV4,TNFRSF10B,CCL20,MCL1,SPP1,PYGB,CD80,SLC16A1,DSC2,KLK7,CA9,MMP1,POSTN,ADA,STAT1,PDZD2,CTSL1,ZNF135,TGFA,IL6,SLC7A5,RHOC,BIRC5,PPM1D,ANXA5,MMP14,HBD,NRAS,CEACAM7,AURKA,ADAM10,SLURP1,CLDN7,EGFR,AIM2,KRT13,TLR5,HMGA2,SSFA2,HBB,BCL2A1,MT1L,IL1B,CEACAM3,FN1,MS4A2,BUB1B,S100A7,TPM1,ISG15,ATP6V1C1,DKK1,FSCN1,LGALS1,IFIT3,FXYD5,ODC1,ANGPT2,TMPRSS11A,BIRC3,MMP9,AQP3,CBFA2T3,HLA-E,FADD,ERBB3,MMP11,IL11,FIGF,HBA1,MYB,IFI27

Genes related with cancer : 815 NPDC1,QSOX2,HMGCR,SMR3A,ABCA3,XCL1,CDH3,LGALSL,C10orf90,STK10,DACT2,PTN,SKA3,ITGA5,LPP,TGM1,NR3C2,CYP26A1,APOL2,EMCN,CYP11A1,CCNA1,TK1,NBN,PANX1,DIRAS3,CADM4,SLC7A6,HLA-F,ADAMTS18,GGH,FAM3C,GSTA1,CNN2,MPPED2,EIF2AK2,DBP,CCL11,CXCL3,RGS5,PAK1,HSPB8,PRDM14,COL4A2,EHF,PACRG,ARSF,UBASH3B,CITED2,FCGR2A,WISP2,TRIO,PEBP4,ITIH5,IGF2BP3,LGR6,PADI1,RTP3,FAM43B,EXT1,RECQL,UPK1A,IKZF2,MKNK2,MS4A1,SOD2,DIRAS1,PML,F2RL2,GDA,IGF2BP2,BATF2,CEACAM6,CENPE,IRF1,IL20,LPAR3,ASPM,CCL18,LMNB1,CRABP2,TSPAN12,CAPS,MGLL,SLCO1B3,PMEL,PALM,RORA,SASH1,IL17RD,BRCA2,OCIAD2,IL2RA,CALB1,ENDOU,OGN,FAP,LY6K,PLEK,TRIM22,TTR,CLCA4,CLDN5,DKK2,PTGDS,MPZ,BST2,SHC1,KCNIP3,STX1A,IRX5,ITGA3,LYN,DUSP6,CDC25B,PTPN12,INHBA,CTNNBIP1,ASCC3,CDCA3,PTPRZ1,EFNB1,GINS1,SERPINE2,SOX21,NTRK2,C5AR1,CYP2C19,ADAM33,IFI6,SMO,AGRN,BTC,CRYM,KRT1,AOC4,LOXL2,ANXA3,ATAD2,CTSG,ELANE,ATOH1,TEK,NUCB2,AADAC,CLIC3,PSMB7,FOXO4,RAC2,CCL3L3,HTR1B,ID4,SOX5,ISL1,PLA2G1B,P2RY6,SLC5A5,IL12RB2,NFIX,CYP27B1,CADM3,CD1A,OCLN,ANLN,KRT4,UCHL1,EPHX3,GZMA,CDON,SELL,CYTL1,FBN2,SLC16A7,CHMP5,TAP2,CCL19,OSGIN1,CXCL2,KLK13,GALNT6,DCBLD2,STIL,VWA5A,LIFR,UBE2C,STK17A,FOXE1,HTR7,TNFRSF9,CCNE1,FBLIM1,MXI1,DUSP4,NCAM2,CD79A,S1PR1,MSMB,BRMS1,HOXD10,CYSLTR1,ISM1,HENMT1,FOXM1,NLRP2,NELL1,DACT3,TRPV6,ACSBG1,CTLA4,ANGPT4,CCNB2,HOXC9,BCL2L10,CYP2C18,ALDH3A1,CNKSR3,PLCD1,IFIT5,F2RL1,POU2AF1,MIR31HG,OSMR,FUT3,GDF10,DBI,CALML3,IFIT1,LAMP3,ABCA1,PDGFA,CCL8,ZHX2,RARRES3,PLXNA1,SORT1,DNASE1L3,LLGL2,CRYAB,CHEK1,SLC38A3,WNT7A,TSPAN8,TPCN1,RHOB,KIF11,MUCL1,GBP1,CEP55,MPHOSPH6,IL24,XIST,CLU,BDKRB1,TRIM29,CXCL12,MYH9,NUP37,TRPM1,LBP,TMPRSS11E,NCF2,GLUL,EFHD1,PLSCR1,ADAM19,TF,GUCY2C,FEZ1,APOD,NNAT,BARX2,GABRP,CX3CL1,BCL9,RBBP8,COL14A1,ADAM23,CXCR2,APBB1,CBX7,IRS1,TSPAN7,MICU1,MTAP,BAMBI,CD19,HPRT1,HELLS,TNS4,FANCI,NR0B1,LTBP1,GNRHR,PSMA7,C3orf52,TSC22D3,PLAC1,JAM2,DLX5,CCM2,LRRC4,TAP1,SMPD3,CNTFR,NRIP1,SLC9A1,PDE7A,FUT6,PLEKHG6,AFAP1L1,THBS2,PSMB4,PLEC,DIO2,ITGA1,PRSS23,AGT,WNT2B,SLC3A2,PTGES,MAPK13,SCD5,TEC,MAL,RGS22,GSTA3,NCOA1,PLD1,COL7A1,NFIL3,LOC440354,APLNR,TFAP2B,HIST1H2AL,CLEC3B,MMAB,FRK,PLA2G7,NUPR1,MACC1,SLC1A5,LPXN,CXCL11,PICALM,MCM4,TP53INP2,ANTXR2,KIF18A,MYBL2,HLF,MAP2,SPRY2,MAPT,IL1RL1,KIR3DL2,APOBEC3B,PRLR,SLC9A3R1,SLC9A3,CYP4F3,CFTR,CAMK1D,FKBP15,ASAH1,TST,ZNF350,RIPK2,ECM1,DDX11L2,SIX3,CAB39,CD70,NPPC,SAA1,KIF14,KRT15,DLX4,SMPD2,VASN,SELE,PBX1,ABCB1,KRT17,GNG7,TIMELESS,MAD2L1,DLEC1,SPATA19,RAB11FIP1,LRP12,IL1RN,PLAGL1,SEC14L2,SDC3,PIM1,IFIT2,FAIM3,CADM1,MUC15,KLF4,PRIM2,DPEP1,ISX,HJURP,MAP2K6,AQP1,ASAP3,PLEKHA2,RPS6KA6,CMTM5,CYP17A1,IL7R,MYBPH,TSPAN1,GAS1,TM7SF2,IFI16,FAM84A,GALR2,CXCL10,CCL21,CAPN6,NUAK1,PPL,LAMB3,ZNF185,TYR,VIT,LCP2,DEPTOR,RNASEH2A,SERPINE1,KDM1A,CCDC6,VMP1,EFNA2,RARG,MKI67,SLC22A1,GLS,BACH1,IL13RA2,DOCK5,PITX2,AOC3,SOX9,CRABP1,STAT2,TGIF1,OAS3,CLSPN,PTAFR,CD1E,PITX1,P2RY1,ODAM,OAS1,NDRG2,FETUB,ANKS1B,GSTO2,PSORS1C2,SDR9C7,MARVELD1,RAPGEF1,GLIPR1,STC1,ASAP1,SEMA3G,SULF1,MEIS1,KIF20B,LTB4R2,TGFBR3,SPINK6,VSIG1,CCR2,CMA1,PDGFD,RAD51,CIT,FST,BEX2,FAM3B,ARHGEF26,BLM,SH3GL3,SLC38A1,ITGB1,IL32,CCL14,SCRN1,PPFIA1,VLDLR,SDC4,HMMR,WISP3,KIF4A,PRKCQ,ND6,ELN,WARS,PSMD13,SNCAIP,NCAPG2,DCT,ELF5,LY6E,CHRNA3,LGALS3BP,CXCL5,ASPA,ITGAV,ABCC3,HOXB9,RARRES1,SPRR3,SAMD9L,PITPNM3,DUOXA1,SCNN1A,NAPB,FABP4,EXO1,JHDM1D,ABCA2,SOSTDC1,EDNRB,TYRP1,SLIT3,CCND2,EIF2S1,CFB,DDX58,TNFRSF17,FSTL3,RSPO1,AJUBA,MAF,ABCA7,RTKN,FRZB,CASP1,ADM,SPHK1,SLC5A1,XAF1,SFTPD,ADAM9,CYP7B1,APAF1,FAM107A,SPRY4,RORC,AMPD1,WNT5A,IFIH1,KRT10,NUAK2,BMP7,ARG1,KIF2C,GPX3,APOBEC3A,NOV,KIF20A,DDAH2,NEGR1,LYPD3,KLK1,FNDC3B,SNRPB,P2RY2,FGF12,ICOS,GPRC5A,STK3,ELF4,CAV2,TGM3,PTP4A1,TCEA3,CYP2C9,CCL15,FAM129B,ITGA6,NCK2,SH3GLB2,DKK4,IGFBP2,BMX,ATP1B2,PRKDC,SPINK7,CCL4,PDCD1LG2,CALD1,POMC,RRAS,SKIL,BCL11A,SOST,ACLY,HSPA1A,PDZD4,S100A7A,MMRN1,SMS,MAPK7,PARD6A,ST3GAL5,IL17RB,TPPP3,CALB2,CD79B,TNFRSF19,LY6D,RHOV,C1QA,ANKRD1,PTK6,TPST1,TNFSF18,PTGS1,SPECC1,PMEPA1,MELK,F3,CFP,FUT7,IL1RAP,PRELP,SYNPO,TREM1,SRGN,TSPYL5,CASP7,TRIB1,IFNAR2,CYB5A,TIMP4,ICOSLG,AATF,KLK12,CDC20,CX3CR1,DSG1,UGT1A6,ACSL5,ANG,AGSK1,PLP1,ERRFI1,MLLT11,PRSS3,GAL3ST1,NPBWR2,TBP,ACPP,SNED1,LIMS1,ZWINT,RAET1E,MAS1L,NDC80,KLK11,AREG,CIDEB,ZBTB7C,TINAGL1,PBOV1,ITGA2,GLI3,LIMK1,IFITM1,DOCK4,KIAA0101,PSCA,UBD,CEBPB,NEDD1,FMNL2,TRPS1,PDIA4,GAST,GREM1,POPDC3,ITPR2,SPRR1A,CCL3,AMOT,FZD10,STAB2,LDOC1,KRT31,NTRK3,NOTCH3,RBP1,IRF9,ART4,MLPH,ATIC,MALL,CCNA2,NRG1,LGR4,ROR1,TTK,LEPREL1,TGFBI,TYRO3,IVL,MAPK4,PLOD2,HAVCR2,PHLDB2,PGD,CDKN3,PCSK2,ZG16B,E2F7,MAGI1,CYP2J2,MAP3K1,LAMA3,NUF2,NUDCD1,GAB2,CD86,APPL1,FLNA,C2orf40,TEX101,CD274,TNFRSF12A,AOX1,BNIPL,SERPINA5,CYP24A1,EVPL,SELENBP1,ANO6,PVRL4,PROCR,SSTR4,KRT81,PKP2,DARC,PDIK1L,HRASLS,TYMP,HLA-DQA1,DFNA5,GULP1,CFD,CYP3A4,IGFBP5,TMPRSS2,ZAK,SKA2,NRTN,CTGF,SEMA3C,EPHX2,KAT2B,CYP4B1,LPHN3,APP,UBE2T,IFI44,CENPF,IGF2BP1,FGF23,CKS2,CXCL17,ANGPTL1,PTGIS,RGS1,MIR210HG,SMURF2,SPRR1B,PSD3,ACER3,MX2,ADAMTS12,HBG1,NDN,EYA2,CD1C,HBA2,PSMB9,MAGEA4,ST6GALNAC1,AJAP1,CACNA2D3,HOPX,CSF2,DUOX1,CBR1,ITPR3,ECT2,KIF23,DEPDC1,GNLY,CCL13,AGPAT9,FAM83A,SMOC2,SERPINB11,CD14,CTSC,CDCA8,XRCC2,ESM1,KCNH1

>>>>>>>>>>>>>>>>>>>>>>>>>>>>>>>>>>>>>>>>>>>>>>>>>>

Annotations or Biological Concepts detected

###########################################

Annotation No. of genes GeneList

--------------------------------------------

Apoptosis 389 MMP1,CXCL11,CXCL10,SERPINE1,TGFBI,CD274,IL20,FST,CXCL9,IFI6,TNFRSF12A,IL24,DDX58,KIF14,DUSP6,CTSC,LY6E,SLCO1B3,CYP27B1,TLR2,IL2RA,DFNA5,OAS3,XAF1,IFI44,TAP1,EIF2AK2,TPM1,FCGR3B,IRS1,AREG,AURKA,CTLA4,LRP12,STC1,ANGPT2,F3,HOXC9,PDE7A,FAIM3,LYN,IGF2BP2,ICOS,E2F7,TNFRSF10B,SOD2,IRF1,CSF2,CCL3,TRIB1,CCNA1,MAGEA4,DOCK5,GLIPR1,PSMB9,IL32,PHLDA1,ECT2,IRF9,ITGAV,NDC80,TNFRSF9,ANXA3,IL7R,CCL18,STK17A,IFIH1,SDC4,CCL4,PI3,IFI27,FOXM1,CCL13,RAD51,CFB,ADM,MMP9,PML,EXO1,CDC25B,SULF1,TSPAN1,CCM2,CCL8,CDC20,APOBEC3B,PRKDC,EIF2S1,OAS1,MMP11,CCNA2,SOX9,HMMR,GREM1,CASP7,ITGA2,APP,CDK1,EXT1,RRAS,IFIT5,F2RL1,CHEK1,LPAR3,ITGB1,PROCR,SLC3A2,CEBPB,CALB1,PLXNA1,LAMP3,CD86,GNLY,COL4A2,F2RL2,NFIL3,RARRES3,PAK1,NBN,MYBL2,ANXA5,RHOB,ZHX2,CNN2,PLEC,ABCA1,BLM,EPHB2,APOL2,TNFSF10,CD14,HELLS,BIRC2,IL6,BRCA2,SHC1,KDM1A,CALB2,BRMS1,CKS2,ITPR3,STK3,ELF4,GLS,SPHK1,PTAFR,NUP37,TGFB1,BIRC5,FBLIM1,AATF,KIF11,CCNE1,ERRFI1,KIF20A,NAPB,DUSP4,PRKCQ,MLLT11,LMNB1,UBD,XCL1,EGFR,SLC9A1,APAF1,ZAK,CCND2,HENMT1,SPECC1,SELL,QSOX2,SEC14L2,LCP2,PTGES,PAG1,CD70,IFI16,KIF20B,IGF2BP1,LIMS1,MAD2L1,ANKRD1,ATIC,ATG5,PRIM2,RBBP8,CASP1,ABCC3,BAK1,BIRC3,RAPGEF1,TGIF1,ADAM9,FADD,GAST,MICU1,RAC2,CCL11,PLEK,HOXB9,TBP,BCL2L11,C5AR1,UCHL1,MCL1,RECQL,GZMA,BCL2A1,CHMP5,MPHOSPH6,JUN,SKIL,IL1RAP,CYP24A1,ACLY,THBS2,RNASEH2A,ANG,ERBB3,PRDM14,WNT5A,PLAGL1,LDHD,MAPK4,OSGIN1,NUPR1,WNT2B,CYTL1,HSPB2,SMOC2,HMGCR,NPBWR2,APBB1,MS4A1,KCNH1,NTRK2,CCL21,PLA2G1B,ID4,IL17RB,SSTR4,PEBP4,GAB2,RPS6KA6,TYRO3,MSMB,GPRC5A,PIM1,CD34,MAGI1,HTR1B,GALR2,DPEP1,TF,SPRY2,CACNA2D3,AGT,TP53,AMPD1,MMAB,CD79A,KAT2B,ELANE,ASAH1,POMC,CADM1,KCNIP3,FOXO4,CTNNBIP1,CRYM,IRX5,SNCAIP,GLUL,DACT3,ITPR2,CYP26A1,DUOXA1,PSCA,RORC,CMTM5,BMX,BTC,MAS1L,TSPYL5,MAPK7,NELL1,CD19,GAS1,NUCB2,NOTCH3,TEK,RARG,FGFR2,DLX4,SLIT3,TRPV6,CXCR2,NLRP2,ABCB1,LGR6,HSPB8,TTR,RHOV,MMRN1,ANGPT4,FOXE1,EDNRB,DIRAS1,GNRHR,CYP4F3,SFRP4,NRTN,NDN,CIDEB,TMPRSS2,TIMP4,CFTR,NTRK3,EVPL,ACPP,SMPD2,DIRAS3,CITED2,DLEC1,PTK6,KLK7,CLU,MXI1,CEACAM6,NOS1,HSPA1A,MAP3K1,GDF10,TPPP3,OCLN,KLK1,P2RY2,PTGDS,MEIS1,GDA,MGLL,BEX2,CCL19,CYP2J2,SASH1,CAPN6,KLF4,PADI1,BCL2L10,SLC9A3R1,AQP3,SMO,BMP7,TGM1,CYSLTR1,TRPS1,ANGPTL1,RORA,PRLR,DEPTOR,LDOC1,SLC5A1,MAPT,PTN,PITX1,IGFBP5,P2RY1,CXCL12,CYP3A4,IGFBP2,FETUB,ROR1,FRZB,MAP1LC3A,AADAC,SCNN1A,DNASE1L3,RAET1E,PITX2,FAM3B,GPX3,HLF,DCT,KRT1,TYR,PMEL,TYRP1,ENDOU,MAL

Therapeutic 490 CXCL11,CXCL10,PTHLH,NELL2,S100A7A,IFIT1,IDO1,SERPINE1,IL8,SPINK6,MUCL1,PDCD1LG2,CD274,IFIT2,GALNT6,TNFRSF12A,IL24,DDX58,KIF14,CA9,IL2RA,XAF1,IFI44,TAP1,CEP55,PLAC1,EIF2AK2,SLC16A1,LY6K,MELK,IRS1,CD80,BATF2,AREG,FSCN1,AURKA,CTLA4,PHLDB2,F3,WISP3,HOXC9,FAT1,ESM1,LYN,IGF2BP2,VEGFC,WNT7A,ICOS,E2F7,OSMR,IFNAR2,SOD2,IRF1,CSF2,SNAI2,CCNA1,MAGEA4,SERPINE2,GLIPR1,PSMB9,IL32,KIF18A,ITGAV,PLSCR1,NDC80,MTAP,TNFRSF9,ANXA3,IL7R,CCL18,STAT2,CCL4,NRIP1,IFI27,BST2,FOXM1,RTP3,TXNRD1,ATAD2,RAD51,PLAUR,ADM,MMP9,GBP1,TTK,IL13RA2,PML,MX2,CDC25B,SULF1,RTKN,FAP,FBN2,TSPAN1,FABP4,NUDCD1,CCL8,CDC20,DCBLD2,APOBEC3B,PRKDC,MSN,CCNA2,SOX9,UBE2C,HMMR,GREM1,CASP7,MCM4,CXCL3,APP,RRAS,VMP1,IFIT5,CDKN2A,F2RL1,CHEK1,LPAR3,ITGB1,PROCR,SOCS3,SLC3A2,CEBPB,HJURP,CD86,GNLY,TYMP,SLC22A1,COL4A2,DEPDC1,NFIL3,CXCL5,PAK1,NBN,KIF23,FCGR2A,PICALM,MYBL2,ACSL5,TINAGL1,HAVCR2,RHOB,GINS1,LOXL2,ZHX2,ASAP1,ABCA1,BLM,AURKB,EPHB2,KIF2C,TNFSF10,CD14,PXN,IL6,IGF2BP3,BRCA2,SHC1,KDM1A,CALB2,BRMS1,CKS2,IFNG,SMURF2,TNFSF18,ST3GAL5,TFRC,GLS,SPHK1,PTAFR,NUP37,APLN,NRP2,STX1A,CTGF,FBLIM1,AATF,KIF11,CCNE1,ERRFI1,BACH1,PTP4A1,NAPB,DUSP4,MLLT11,CDCA3,UBD,SMS,XCL1,EGFR,NCAPG2,SLC9A1,NUAK1,STIL,APAF1,CCND2,SDC3,ASPM,FLNA,CENPE,SELL,LEPREL1,PSMA7,VSIG1,SEC14L2,TK1,PTGES,CD70,IFI16,KIF20B,IGF2BP1,RHOC,CD151,STK10,MAD2L1,EFNB1,CIT,ANTXR2,ATIC,KIAA0101,PRIM2,RBBP8,NME1,SAMSN1,CASP1,ABCC3,LIMK1,PKP2,PLEKHA2,RARRES1,BIRC3,PRSS23,TGIF1,ADAM9,HPRT1,GAST,RAC2,LAMB3,CCL11,HOXB9,CAV2,C5AR1,UCHL1,MCL1,CDCA8,RECQL,GZMA,CHMP5,JUN,SKIL,IL1RAP,CXCL2,CYP24A1,ACLY,PYGB,RNASEH2A,ANG,ATOH1,FGFR3,SLC5A5,ISX,CAPS,RGS22,ITIH5,PPM1D,PRDM14,WNT5A,ZNF135,PLAGL1,ISM1,RAB11FIP1,MAPK4,BCL9,NUPR1,WNT2B,CYTL1,CD1E,CD79B,NNAT,HMGCR,MS4A1,KCNH1,MAP2K6,NTRK2,CCL21,SPATA19,PLA2G1B,ID4,CX3CL1,IL17RB,LTB4R2,SSTR4,DLX5,PEBP4,PSORS1C2,GAB2,TYRO3,MSMB,GPRC5A,PIM1,MAGI1,HTR1B,TF,ABCA7,SPRY2,NCAM2,MAPK13,CACNA2D3,AGT,TP53,S1PR1,CYP17A1,AMPD1,MMAB,NR0B1,SMR3A,ANKS1B,CD79A,MPPED2,KIR3DL2,CMA1,CNTFR,KAT2B,SOX10,ASAH1,KRT31,POMC,CADM1,MTUS1,KCNIP3,EPS8,FOXO4,TNFRSF17,CRYM,PLD1,ICOSLG,DACT3,ELN,DUOXA1,PSCA,C10orf90,MS4A2,CDON,PITPNM3,NOV,BMX,CRABP1,BTC,SLC38A3,MAPK7,SNED1,CD1C,NELL1,CD19,GAS1,MKNK2,NUCB2,AMOT,VWA5A,UGT1A6,NOTCH3,TEK,RARG,LGALSL,FGFR2,DLX4,SLIT3,TRPV6,CXCR2,ABCB1,CP,LGR6,PVRL4,HSPB8,MMRN1,ANGPT4,MAP2,EDNRB,DIRAS1,GNRHR,ABCA3,SFRP4,LBP,ARSF,CCR2,CRABP2,TMPRSS2,TIMP4,CTSG,ASPA,CFTR,NTRK3,EVPL,ACPP,CBR1,DIRAS3,MACC1,DLEC1,PTK6,KRT81,CLU,MXI1,HBA2,CEACAM6,CX3CR1,CAMK1D,HSPA1A,NR3C2,MAP3K1,GDF10,NUAK2,PRSS3,OCLN,NFIX,KLK1,P2RY2,CCL15,SOST,PTGDS,LYPD3,CD1A,MEIS1,GDA,WISP2,RSPO1,ART4,BEX2,LY6D,APOD,PDGFD,VLDLR,CYP2C9,CAPN6,AR,MAF,TSPAN7,SDR9C7,KLF4,ECM1,BCL2L10,FZD10,SLC9A3R1,SMO,BMP7,FCER1A,TGM1,CYSLTR1,TEX101,AQP1,ANGPTL1,EHF,RORA,GSTA1,PRLR,BARX2,IL1RN,SLC5A1,MAPT,PTN,IGFBP5,SOX21,P2RY1,FUT3,CXCL12,CYP3A4,IGFBP2,ARG1,ROR1,FIGF,CYP4B1,CCL14,SELE,TMPRSS11E,PPL,HOPX,PITX2,GUCY2C,ALDH3A1,DCT,CYP2C18,KLK13,CHL1,TYR,ODAM,PMEL,TYRP1,XIST,SPINK7,TMPRSS11A,MAL

Diagnostic 220 CXCL10,SERPINE1,TGFBI,MUCL1,FST,TNC,TNFRSF12A,KIF14,HMGA2,CAV1,ITGA3,LTBP1,IL2RA,TNFAIP3,XAF1,PDPN,CEP55,MKI67,NRG1,LY6K,IRS1,FN1,STC1,FAT1,ESM1,IGF2BP2,VEGFC,ICOS,IRF1,CCL3,CCNA1,PDGFA,MTAP,FAM83A,S100P,CCL4,IFI27,FOXM1,ATP6V1C1,RAD51,ADM,MMP9,PML,CDC25B,SULF1,FBN2,FABP4,STAT1,CCNA2,SOX9,UBE2C,HMMR,GREM1,CASP7,MCM4,ITGA2,IFIT5,CDKN2A,CLSPN,CHEK1,ITGB1,CALB1,XRCC2,GNLY,ADA,CDC6,DEPDC1,CXCL5,FCGR2A,SLC38A1,ASAP1,PLEC,ABCA1,AURKB,CD14,HELLS,IGF2BP3,BRCA2,KDM1A,CALB2,TFRC,SPHK1,GGH,CTGF,FBLIM1,CCNE1,ERRFI1,EGFR,STIL,APAF1,CCND2,SCD5,SELL,SEC14L2,CD70,ANO6,KIAA0101,PRIM2,BIRC3,GAST,CCL11,CAV2,UCHL1,MCL1,NOP2,PYGB,ANG,CAPS,RGS22,WNT5A,OSGIN1,CD1E,CD79B,HMGCR,APLNR,MS4A1,KCNH1,CLDN5,NTRK2,PLA2G1B,ID4,FGF23,CBFA2T3,TYRO3,MSMB,GPRC5A,TACSTD2,PIM1,LLGL2,CD34,HTR1B,TF,KRT20,MAPK13,ACSBG1,AGT,DBI,BCL11A,TP53,S1PR1,KRT10,DBP,CD79A,KIR3DL2,CMA1,ELANE,SOX10,KRT31,POMC,CADM1,PDZD2,ELN,PSCA,NOV,SLC38A3,SNED1,CD1C,CD19,UGT1A6,NOTCH3,TEK,SLIT3,CXCR2,ABCB1,TTR,FGF12,MAP2,SFRP4,TMPRSS2,KIT,ATP1B2,TIMP4,CFTR,NTRK3,ACPP,DLEC1,CLU,CEACAM6,TRPM1,HSPA1A,SFTPD,GDF10,PBOV1,KLK1,PTGDS,LYPD3,CD1A,MEIS1,AGSK1,CHRNA3,LY6D,AR,MAF,TSPAN7,ECM1,SMO,TEX101,EHF,KRT15,EPHX2,MAPT,PITX1,KRT19,IVL,CXCL12,IGFBP2,ARG1,SELE,GUCY2C,ALDH3A1,MLANA,KLK13,TYR,ODAM,PMEL,TYRP1,CLCA4,XIST,SERPINB11,MAL

Metastasis 595 MMP1,MMP10,MMP3,CXCL10,PTHLH,POPDC3,PLAU,MMP13,IFIT1,SERPINE1,SEMA3C,IL8,TGFBI,SPP1,SPINK6,LAMC2,MMP7,MUCL1,CD274,IFIT2,IL20,S100A7,FST,TNC,TNFRSF12A,PTPRZ1,KIF14,SAA1,CA9,DUSP6,COL4A1,CTSC,ITGA3,LTBP1,CYP27B1,LAMA3,IL2RA,DFNA5,MET,WARS,TNFAIP3,XAF1,IFI44,PDPN,TAP1,CCL20,ICAM1,CDH3,ITGA1,ITGA5,MKI67,NRG1,LY6K,MELK,IRS1,HOXD10,BATF2,AREG,FN1,CTLA4,LRP12,STC1,ETS1,PMEPA1,F3,WISP3,FAT1,ESM1,LYN,IGF2BP2,VEGFC,CTSL1,WNT7A,PANX1,ICOS,DKK1,IFNAR2,SOD2,OCIAD2,IRF1,CSF2,CCL3,SNAI2,VAV2,CCNA1,PDGFA,IL1RL1,PSMB9,IL32,KIF18A,ECT2,TNS4,ITGAV,MTAP,TNFRSF9,ANXA3,IL7R,CCL18,TRIO,RGS1,SDC4,CCL4,NRIP1,LGALS1,IFI27,BST2,FOXM1,CCL13,RTP3,TXNRD1,RAD51,FMNL2,PLAUR,ADM,ETV4,MMP9,GBP1,PML,SPRY4,CDC25B,SULF1,LGALS3BP,FAP,FBN2,TSPAN1,FABP4,DSG2,NUDCD1,GCNT1,CYR61,APOBEC3A,MMP14,DCBLD2,TRIM22,APOBEC3B,FXYD5,PRKDC,CCNB1,PTPN12,MSN,CCNA2,SOX9,UBE2C,HMMR,GREM1,CASP7,MCM4,NRAS,THBS1,CXCL3,ITGA2,EXT1,ADAM10,RRAS,AJAP1,VMP1,IFIT5,ZYX,CDKN2A,F2RL1,CHEK1,LPAR3,ITGB1,PROCR,SLC3A2,CEBPB,LAMP3,CD86,POSTN,NEDD1,TYMP,SCRN1,COL4A2,CDC6,F2RL2,AGRN,CXCL5,ADAM19,PAK1,NBN,PICALM,MYBL2,SLC6A2,MYBPH,PLOD2,ANXA5,TINAGL1,HAVCR2,RHOB,LOXL2,CNN2,ASAP1,PLEC,FNDC3B,ABCA1,AURKB,C1QA,TIMP1,EPHB2,TNFSF10,PDIA4,CD14,ITGA6,FANCI,PXN,IL6,IGF2BP3,BRCA2,SHC1,COL7A1,CD44,CALB2,MT1L,BRMS1,CKS2,SMURF2,VCL,TNFSF18,IL11,ST3GAL5,SERPINH1,GLS,SPHK1,PTAFR,NUP37,PSMD13,FAM3C,LPP,BIRC5,CTGF,FBLIM1,HLA-E,CCNE1,ERRFI1,BACH1,PTP4A1,DOCK4,MLLT11,LMNB1,XCL1,EGFR,SRGN,SSFA2,CALD1,SLC9A1,NUAK1,STIL,APAF1,ZAK,CCND2,SDC3,FLNA,SELL,PSMA7,LPXN,SLC7A6,SEC14L2,TK1,PTGES,CD70,IGF2BP1,RHOC,LIMS1,CD151,MAD2L1,TPST1,EFNB1,ANO6,KIAA0101,PPFIA1,PRIM2,RBBP8,NME1,CASP1,LIMK1,PKP2,PLEKHA2,ADAM9,FADD,GAST,ALCAM,RAC2,LAMB3,CCL11,HOXB9,CAV2,C5AR1,PSMB4,UCHL1,MYH9,SYNPO,FKBP15,CAB39,BCL2A1,TIMELESS,JUN,SKIL,CXCL2,CYP24A1,ACLY,THBS2,MRPL28,RNASEH2A,PTGIS,ANG,SLC5A5,CAPS,RGS22,ITIH5,ERBB3,SH3GLB2,WNT5A,ZNF135,PKP1,PLAGL1,CEACAM3,MAPK4,BCL9,NUPR1,WNT2B,CYTL1,CD1E,SMOC2,NNAT,PSD3,FAM129B,MS4A1,KCNH1,MAP2K6,CLDN5,NTRK2,CCL21,PLA2G1B,ABCA2,ID4,SERPINA5,AGPAT9,CX3CL1,CADM3,LTB4R2,TPCN1,PEBP4,FGF23,GAB2,DKK2,TYRO3,AOC3,AOX1,MSMB,ZNF350,LLGL2,CD34,FAM84A,IL17RD,ZG16B,DPEP1,TSPAN12,EFNA2,TF,SPRY2,MAPK13,CACNA2D3,ACSBG1,AGT,TP53,S1PR1,CYP17A1,NR0B1,KRT10,CYP11A1,TLR5,ANKS1B,DCN,CD79A,MPPED2,GSTO2,DUOX1,CMA1,KAT2B,ELANE,BAMBI,DIO2,ASAH1,KRT31,POMC,CADM1,ADAM33,EPS8,PDZD2,FOXO4,ISL1,PLEKHG6,PLD1,TP53INP2,GNG7,MYB,ICOSLG,DSC2,GLUL,BDNF,CYP26A1,ARHGEF26,ELN,CCDC6,DUOXA1,ASAP3,FUT7,PSCA,LIFR,PITPNM3,NOV,BMX,LGR4,CRABP1,BTC,SLC38A3,TSPYL5,MAPK7,CD1C,NELL1,ADAMTS18,PALM,CD19,GAS1,CADM4,PLCD1,NUCB2,AMOT,NDRG2,NOTCH3,TEK,RARG,SMPD3,DLX4,SLIT3,SELENBP1,TRPV6,CXCR2,RGS5,NLRP2,ABCB1,HLA-DQA1,TTR,MMRN1,ANGPT4,MAP2,SPRR1A,MIA,EDNRB,DIRAS1,GNRHR,SFRP4,ARSF,CCR2,CIDEB,CRABP2,TMPRSS2,KIT,ATP1B2,TIMP4,CTSG,SORT1,NTRK3,ACPP,SEMA3G,DIRAS3,ADAM23,TNFRSF19,STAB2,CRAT,CLEC3B,DARC,CNKSR3,PRELP,MACC1,CITED2,MAPK3,DLEC1,PTK6,S100A14,KRT81,CLU,MXI1,CEACAM6,TRIM29,TRPM1,JAM2,CX3CR1,CAMK1D,RAB25,HBA1,HSPA1A,NR3C2,LPHN3,IKZF2,MAP3K1,GDF10,ST6GALNAC1,PRSS3,OCLN,NFIX,KLK1,P2RY2,CCL15,PBX1,SOST,PTGDS,LYPD3,EYA2,CD1A,TGFBR3,WISP2,RSPO1,ART4,BEX2,CRYAB,CCL19,UPK1A,LY6D,EMP1,APOD,SASH1,PDGFD,VLDLR,TSPAN8,SOSTDC1,LRRC4,AR,SDR9C7,KLF4,VIT,ECM1,FZD10,SLC9A3R1,BMP7,TGM1,TEX101,AQP1,ANGPTL1,HRASLS,SLURP1,RORA,PRLR,BARX2,EPHX2,TM7SF2,CLDN7,GSTA3,ZNF185,IL1RN,PGD,MAPT,PTN,KRT19,IGFBP5,SOX21,DKK4,IVL,HBB,FUT3,CXCL12,CYP3A4,IGFBP2,ARG1,ROR1,FIGF,CCL14,FRZB,SELE,SH3GL3,HOPX,SELP,DSG1,BNIPL,CEACAM5,OGN,GPX3,ELF5,GUCY2C,ALDH3A1,DCT,CEACAM7,KLK13,KRT1,TYR,PMEL,TYRP1,MUC15,CLCA4,KRT13,SPINK7,ENDOU,KRT4,TGM3,MAL

Immortalization 6 FOXM1,AATF,TP53,CD19,PTK6,PBX1

ImmunoModulation 10 MIR31HG,HTR7,C3orf52,LOC440354,AOC4,MIR210HG,GNG7,DDX11L2,PGD,TYRP1

Prognostic 364 CXCL10,PTHLH,INHBA,IFIT3,PLAU,MMP13,SERPINE1,SEMA3C,TGFBI,SPP1,LAMC2,MMP7,MUCL1,PDCD1LG2,CD274,GALNT6,TNC,TNFRSF12A,KIF14,CA9,HMGA2,CAV1,TREM1,LAMA3,IL2RA,MET,NCF2,XAF1,TAP1,CCL20,PFN2,ITGA1,MKI67,TPM1,FEZ1,NRG1,MELK,BATF2,AREG,FSCN1,FN1,CTLA4,ETS1,ANGPT2,WISP3,FAIM3,ESM1,LYN,SEMA3A,IGF2BP2,VEGFC,IFITM1,WNT7A,ANLN,OSMR,ADAMTS12,ISG15,OCIAD2,IRF1,TPX2,CCL3,TRIB1,PDGFA,IL1RL1,IL32,PHLDA1,ECT2,TNS4,MTAP,TNFRSF9,FAM83A,ANXA3,RGS1,BUB1B,IFI27,FOXM1,ATAD2,RAD51,PLAUR,ADM,MMP9,PML,HLA-A,CDC25B,SULF1,LGALS3BP,TSPAN1,CYR61,APOBEC3A,MMP14,CDC20,FXYD5,PRKDC,CCNB1,MSN,CCNA2,SOX9,UBE2C,HMMR,HLA-F,GREM1,CASP7,THBS1,ITGA2,CDK1,EXT1,AJAP1,VMP1,CDKN2A,CHEK1,PROCR,SLC3A2,HJURP,LAMP3,GNLY,TYMP,SLC22A1,SCRN1,AFAP1L1,CDC6,DEPDC1,CXCL5,PAK1,NBN,PICALM,MYBL2,SLC6A2,MYBPH,HAVCR2,LOXL2,CENPF,PLEC,KIF4A,AURKB,KIF2C,CD14,KRT17,ITGA6,GABRP,IL6,IGF2BP3,BRCA2,SHC1,KDM1A,CD44,CALB2,ABL2,CKS2,SMURF2,TAP2,TFRC,SPHK1,APLN,CTGF,FBLIM1,AATF,CCNE1,NMI,MLLT11,CD163,UBD,EGFR,PSMB7,SRGN,NUAK1,STIL,APAF1,CCND2,SCD5,SDC3,ASPM,FLNA,CENPE,SELL,PSMA7,PSMB8,VSIG1,SEC14L2,TK1,IFI16,IGF2BP1,RHOC,MAD2L1,EFNB1,SLC1A5,HOXA1,KIAA0101,ATG5,RBBP8,NME1,SAMSN1,CASP1,ABCC3,PKP2,RARRES1,BAK1,ADAM9,GAST,LAMB3,CCL11,CAV2,UCHL1,CCNB2,NOP2,SNRPB,IL1RAP,MRPL28,RNASEH2A,PTGIS,ANG,SLC5A5,ISX,RGS22,ITIH5,WNT5A,OSGIN1,NUPR1,CD1E,HSPB2,NNAT,MS4A1,KCNH1,CBX7,NTRK2,PLA2G1B,SERPINA5,CX3CL1,DLX5,GAB2,MSMB,TACSTD2,MAGI1,GALR2,EFNA2,TF,SPRY2,KRT20,CACNA2D3,AGT,BCL11A,TP53,NR0B1,ANKS1B,CD79A,CMA1,ELANE,BAMBI,POMC,CADM1,CYB5A,C2orf40,MALL,CYP26A1,ELN,EPHA7,FUT7,PSCA,LIFR,BDKRB1,BMX,CRABP1,CD1C,PALM,CD19,PLCD1,AMOT,NDRG2,VWA5A,NOTCH3,TEK,SMPD3,DLX4,SLIT3,SELENBP1,TRPV6,CXCR2,NLRP2,ABCB1,PVRL4,HLA-DQA1,TTR,FGF12,MMRN1,ANGPT4,NEGR1,EDNRB,SFRP4,CFD,CIDEB,CRABP2,TMPRSS2,KIT,TIMP4,CFTR,NTRK3,ACPP,CBR1,SEMA3G,DIRAS3,ADAM23,CNKSR3,MACC1,DLEC1,PTK6,PACRG,CLU,MXI1,CEACAM6,TRPM1,HSPA1A,SLC16A7,KLK1,SOST,LYPD3,CD1A,MEIS1,WISP2,CEACAM1,CHRNA3,CCL19,UPK1A,APOD,SASH1,TSPAN8,TSPAN7,CD24,SDR9C7,KLF4,SLC9A3R1,BMP7,CLIC3,TEX101,AQP1,SLURP1,RORA,PRLR,CLDN7,SLC5A1,MAPT,PTN,PITX1,IGFBP5,COL14A1,IVL,CXCL12,CYP3A4,IGFBP2,PLP1,SELE,KLK11,DSG1,PITX2,KLK12,GPX3,GUCY2C,DACT2,DCT,KLK13,TYR,ODAM,PMEL,EPHX3,TYRP1,MAL

CellProliferation 536 MMP3,CXCL11,CXCL10,PTHLH,INHBA,SERPINE1,AIM2,TGFBI,SPP1,MMP7,PDCD1LG2,CD274,IFIT2,GALNT6,FST,TNC,SAMD9L,TNFRSF12A,IL24,PTPRZ1,KIF14,DUSP6,COL4A1,IL12RB2,LY6E,CYP27B1,IL2RA,DFNA5,OAS3,MET,IFI44,TAP1,EIF2AK2,ITGA5,MKI67,CDK6,FEZ1,NRG1,MELK,IRS1,AREG,CTLA4,LRP12,STC1,ETS1,PMEPA1,ANGPT2,F3,WISP3,HOXC9,LIMA1,FAIM3,FAT1,ESM1,LYN,IGF2BP2,VEGFC,IFITM1,WNT7A,PANX1,RIPK2,ADAMTS12,TNFRSF10B,SOD2,IRF1,CSF2,TPX2,CCL3,CCNA1,PDGFA,PSMB9,IL32,XDH,KIF18A,ECT2,IRF9,ITGAV,NDC80,MTAP,TNFRSF9,IL7R,CCL18,TRIO,CCL3L3,STAT2,CCL4,NRIP1,IFI27,FOXM1,RTP3,ATAD2,RAD51,PLAUR,ADM,PLA2G7,TTK,PML,CDC25B,SULF1,RTKN,LGALS3BP,RBP1,FAP,ADAM12,TSPAN1,FABP4,SLC7A5,NUDCD1,CDC20,TRIM22,APOBEC3B,PRKDC,CCNB1,EIF2S1,CCNA2,SOX9,UBE2C,HMMR,GREM1,CASP7,IL1B,NRAS,ODC1,PTK2,ITGA2,CDK1,EXT1,ADAM10,AJAP1,VMP1,IFIT5,TGFA,ZYX,IL1A,CDKN2A,UBASH3B,F2RL1,CLSPN,CHEK1,LPAR3,PROCR,SOCS3,SLC3A2,CEBPB,HJURP,CD86,GNLY,TYMP,SLC22A1,COL4A2,CDC6,F2RL2,AGRN,RARRES3,CXCL5,PAK1,NBN,KIF23,PICALM,MYBL2,SLC6A2,ACSL5,TINAGL1,HAVCR2,RHOB,GINS1,CENPF,KIF4A,ABCA1,BLM,AURKB,CDKN3,EPHB2,ZWINT,KIF2C,BUB1,CD14,KRT17,ITGA6,HELLS,PTGS1,IGF2BP3,BRCA2,SHC1,COL7A1,KDM1A,CD44,P2RY6,CALB2,BRMS1,CKS2,IFNG,ITPR3,SMURF2,STK3,TAP2,GLI3,TFRC,GLS,TLN1,SPHK1,NUP37,APLN,LPP,STX1A,CTGF,AATF,CCNE1,BACH1,PTP4A1,KIF20A,PRKCQ,CDCA3,XCL1,EGFR,NCAPG2,AJUBA,SLC9A1,NUAK1,MARVELD1,APAF1,CCND2,ASPM,SPECC1,CENPE,SELL,FSTL3,SEC14L2,TK1,PTGES,CD70,IFI16,IGF2BP1,LIMS1,MAD2L1,CIT,HOXA1,ANO6,ATIC,ACER3,KIAA0101,PRIM2,RBBP8,FRK,NUF2,NME1,ASCC3,SKA3,CASP1,PKP2,PRSS23,RAPGEF1,ADAM9,HPRT1,GAST,RAC2,LAMB3,CCL11,HOXB9,CAV2,IL23A,SKA2,UCHL1,SYNPO,UBE2T,MCL1,HIST1H2AL,RECQL,GZMA,BCL2A1,CCNB2,NOP2,TIMELESS,JUN,SKIL,CXCL2,CYP24A1,ACLY,THBS2,MRPL28,RNASEH2A,ANG,ATOH1,FGFR3,SLC5A5,ITIH5,ERBB3,NCOA1,SH3GLB2,PDZD4,PRDM14,WNT5A,PLAGL1,ISM1,RAB11FIP1,CFP,MAPK4,BCL9,NUPR1,WNT2B,FAM43B,CYTL1,CD1E,SOX5,GALR1,NCK2,ND6,NNAT,HMGCR,APLNR,EFHD1,MS4A1,KCNH1,MAP2K6,SIX3,CBX7,NTRK2,CCL21,TSC22D3,PLA2G1B,ID4,PDIK1L,CX3CL1,MPZ,TPCN1,DLX5,PEBP4,FGF23,GAB2,CBFA2T3,PARD6A,POU2AF1,RPS6KA6,TYRO3,PIM1,CD34,MAGI1,IL17RD,HBD,ZG16B,GAL3ST1,GALR2,DPEP1,EFNA2,TF,SPRY2,MAPK13,CACNA2D3,AGT,DBI,TP53,AMPD1,MMAB,KRT10,CYP11A1,ANKS1B,CD79A,AQP5,DUOX1,KIR3DL2,CMA1,KAT2B,ELANE,BAMBI,ASAH1,KRT31,POMC,CADM1,ADAM33,FOXO4,TNFRSF17,C2orf40,CRYM,CYP7B1,PLD1,ZBTB7C,TEC,MYB,APPL1,ICOSLG,DSC2,GLUL,ITPR2,NPDC1,CYP26A1,ELN,CCDC6,DUOXA1,PSCA,RORC,NOV,BTC,TSPYL5,MAPK7,SNED1,CD1C,PALM,SLC9A3,CD19,GAS1,NUCB2,NDRG2,NOTCH3,SPRR1B,PPARG,TEK,RARG,MLPH,LGALSL,FGFR2,DLX4,SLIT3,SELENBP1,TRPV6,TCEA3,CXCR2,CYP3A5,NLRP2,ABCB1,VASN,PVRL4,HSPB8,MMRN1,ANGPT4,MAP2,NEGR1,EDNRB,GNRHR,CYP4F3,SFRP4,NDN,CCR2,CRABP2,TMPRSS2,CFTR,NTRK3,EVPL,ACPP,SMPD2,DIRAS3,KLK10,CALML3,DARC,HBG1,MAPK3,DLEC1,PTK6,S100A14,CLU,MXI1,CEACAM6,FAM107A,CAMK1D,HSPA1A,PCSK2,NR3C2,IKZF2,MAP3K1,GDF10,KLK1,CSTB,P2RY2,PBX1,PTGDS,FUT6,CD1A,MEIS1,TGFBR3,WISP2,MGLL,ART4,BEX2,CCL19,APOD,SASH1,PDGFD,VLDLR,CYP2C9,TSPAN8,SOSTDC1,LRRC4,CAPN6,AR,MAF,KLF4,GULP1,VIT,ECM1,BCL2L10,SLC9A3R1,SMO,BMP7,TGM1,CYSLTR1,ANGPTL1,RORA,KRT15,PRLR,DEPTOR,BARX2,EPHX2,TM7SF2,IL1RN,MAPT,PTN,PITX1,IGFBP5,PAX9,DKK4,IVL,P2RY1,FUT3,CXCL12,NPPC,CYP3A4,IGFBP2,ARG1,CXCL17,ROR1,FRZB,PLP1,SELE,AADAC,HOPX,SCNN1A,PITX2,GPX3,ELF5,HLF,GUCY2C,DCT,KRT1,TYR,PMEL,SPRR3,CLCA4

Inflammation 79 MMP3,CXCL11,CXCL10,S100A7A,SERPINE1,S100A7,FST,TNC,SAA1,DUSP6,TREM1,IL2RA,NCF2,TAP1,EIF2AK2,F3,IRF1,PSMB9,KIF18A,TNS4,CCL18,CCL4,FOXM1,CCL13,ADM,PLA2G7,FBN2,PRKDC,OAS1,UBASH3B,F2RL1,CXCL5,ABCA1,CD14,PTGS1,IL6,SPHK1,CTGF,SEC14L2,PTGES,CASP1,GAST,CCL11,JUN,RNASEH2A,ANG,WNT5A,CD1E,HSPB2,HMGCR,CCL21,PLA2G1B,SERPINA5,GPRC5A,CD34,TST,AGT,TP53,CD79A,POMC,ELN,CD1C,TEK,CXCR2,HSPB8,TTR,FOXE1,CFTR,ACPP,CLU,SFTPD,MAP3K1,P2RY2,CD1A,KLF4,IL1RN,CXCL12,CYP3A4,SELE

Angiogenesis 142 CXCL11,CXCL10,NELL2,SERPINE1,IL8,MMP7,IL20,FST,IL24,MET,WARS,NRG1,IRS1,AREG,ETS1,ANGPT2,F3,WISP3,ESM1,IGF2BP2,VEGFC,E2F7,PDGFA,IL32,ITGAV,FOXM1,ADM,MMP9,SULF1,CCM2,PRKDC,CCNA2,UBE2C,GREM1,CXCL3,APP,F2RL1,LPAR3,ITGB1,SLC3A2,CD86,POSTN,TYMP,COL4A2,AGRN,CXCL5,PAK1,JHDM1D,RHOB,ABCA1,KIF2C,CD14,KRT17,ITGA6,IGF2BP3,BRMS1,SPHK1,NRP2,STX1A,CTGF,CALD1,CCND2,SDC3,LCP2,PTGES,PPFIA1,CASP1,HPRT1,GAST,CCL11,THBS2,RNASEH2A,ANG,ISM1,CFP,APLNR,MS4A1,NTRK2,EMCN,PLA2G1B,ID4,CX3CL1,DDAH2,GAB2,MSMB,CD34,EFNA2,TF,SPRY2,AGT,TP53,CMA1,POMC,ELN,CCDC6,BMX,NELL1,AMOT,TEK,SMPD3,SLIT3,CXCR2,ABCB1,TTR,MMRN1,ANGPT4,MAP2,SFRP4,CCR2,CLEC3B,DLEC1,CLU,CX3CR1,MAP3K1,KLK1,PBX1,CYP2C19,CEACAM1,BEX2,VLDLR,CYP2C9,TSPAN8,SOSTDC1,CAPN6,MAF,KLF4,ECM1,AQP1,ANGPTL1,KRT15,TFAP2B,TM7SF2,IL1RN,PTN,CXCL12,IGFBP2,CXCL17,FIGF,CCL14,SELE,CYP2C18,TYRP1

<<<<<<<<<<<<<<<<<<<<<<<<<<<<<<<<<<<<<<<<<<<<<<<<<<<

>>>>>>>>>>>>>>>>>>>>>>>>>>>>>>>>>>>>>>>>>>>>>>>>>>

Genes sharing common annotation or function

###########################################

CommonAnnotation No. of genes Genelist

--------------------------------------------

Therapeutic#Prognostic#Diagnostic#Metastasis#CellProliferation#Inflammation 1 CD1E,CD1C,CD1A

Angiogenesis#CellProliferation 1 CFP,CXCL17

Therapeutic#Diagnostic#Angiogenesis#Apoptosis#Metastasis#CellProliferation 1 IRS1,ID4

Therapeutic#Diagnostic#Apoptosis 1 BIRC3,HTR1B

Therapeutic#Apoptosis#Metastasis 1 RRAS,TNFSF10,PTAFR,C5AR1,DIRAS1,OCLN

Therapeutic#Prognostic#Apoptosis#CellProliferation 1 CDC20,IFI16,MAGI1,PITX2

Apoptosis#Metastasis#Inflammation 1 CCL13

Therapeutic#Prognostic#Diagnostic#Angiogenesis#Apoptosis#Metastasis 1 MMP9,MSMB

Therapeutic#Prognostic#Angiogenesis#Metastasis#CellProliferation 1 WISP3,TYMP,CX3CL1

Therapeutic#Diagnostic#Metastasis 1 LY6K,MCM4,ASAP1,CAPS,S1PR1,SLC38A3,LY6D,ALDH3A1

Therapeutic#Angiogenesis#Metastasis#CellProliferation 1 CCR2,VLDLR

Therapeutic#Prognostic#Apoptosis#Metastasis 1 ANXA3,MLLT11

Therapeutic#Diagnostic#Angiogenesis#CellProliferation 1 MAF

Therapeutic#Prognostic#Angiogenesis#Apoptosis#Metastasis#CellProliferation#Inflammation 1 PRKDC,CASP1,RNASEH2A,KLF4

Therapeutic#Prognostic#Diagnostic#Angiogenesis#Apoptosis#Metastasis#ImmunoModulation 1 TYRP1

Therapeutic#Angiogenesis#CellProliferation 1 STX1A,HPRT1,ISM1,CYP2C9

Diagnostic#Apoptosis 1 CALB1

Prognostic#Metastasis#Inflammation 1 TNS4,SERPINA5

Therapeutic#Metastasis 1 IFIT1,SPINK6,IFNAR2,SNAI2,BST2,TXNRD1,GBP1,DCBLD2,PXN,TNFSF18,ST3GAL5,CD151,LIMK1,PLEKHA2,ZNF135,LTB4R2,CYP17A1,MPPED2,EPS8,PITPNM3,ARSF,CTSG,KRT81,PRSS3,NFIX,CCL15,RSPO1,FZD10,SOX21,SPINK7

Prognostic#Apoptosis#Inflammation 1 HSPB2

Therapeutic#Diagnostic#Apoptosis#Metastasis#CellProliferation#Inflammation 1 CCL4

Angiogenesis#Apoptosis 1 CCM2,LCP2

Prognostic#Diagnostic#Angiogenesis#Apoptosis#Metastasis#Inflammation 1 TTR

Prognostic#Metastasis#CellProliferation 1 SPP1,LGALS3BP,CCNB1,AJAP1,SLC6A2,CD44,MRPL28,BAMBI,PALM,NDRG2,SELENBP1

Therapeutic#Prognostic#Angiogenesis#Apoptosis#Metastasis 1 BMX

Therapeutic#Angiogenesis#Metastasis 1 IL8,CXCL3,CX3CR1,FIGF,CCL14

Therapeutic#Diagnostic#Apoptosis#Metastasis 1 ICOS,ERRFI1

Therapeutic#Diagnostic#Metastasis#CellProliferation 1 FAT1,FABP4,MAPK13,KRT31,NOV,AR,ARG1

Prognostic 1 IFIT3,PFN2,SEMA3A,ANLN,ISG15,BUB1B,HLA-A,HLA-F,AFAP1L1,GABRP,ABL2,NMI,CD163,PSMB7,PSMB8,SLC1A5,SNRPB,CYB5A,MALL,EPHA7,BDKRB1,CFD,PACRG,SLC16A7,CD24,CLIC3,COL14A1,KLK11,KLK12,DACT2,EPHX3

Metastasis#Inflammation 1 S100A7,SAA1

Therapeutic#Prognostic#Apoptosis#Metastasis#CellProliferation#Immortalization 1 PTK6

Apoptosis#Metastasis 1 MMP1,CTSC,SDC4,ANXA5,CNN2,BIRC5,LMNB1,ZAK,FADD,SMOC2,CITED2,ENDOU

Prognostic#Angiogenesis#Apoptosis#CellProliferation 1 ANGPT2

Diagnostic#Inflammation 1 SFTPD

Prognostic#Diagnostic#Metastasis#CellProliferation#Inflammation 1 TNC

Therapeutic#Prognostic#Diagnostic#Metastasis#CellProliferation 1 MTAP,CDKN2A,AURKB,KIAA0101,CAV2,GUCY2C

Therapeutic#Diagnostic#Angiogenesis#Apoptosis#Metastasis 1 ITGB1

Therapeutic#Prognostic#Diagnostic#Apoptosis#Metastasis#CellProliferation#Immortalization 1 CD19

Prognostic#Diagnostic#Apoptosis#Metastasis 1 PLEC

Prognostic#Apoptosis#Metastasis 1 LAMP3,CIDEB

Therapeutic#Prognostic#Diagnostic#Apoptosis#CellProliferation 1 GNLY,KDM1A,MEIS1

Metastasis 1 MMP10,POPDC3,ICAM1,CDH3,HOXD10,CTSL1,DKK1,VAV2,LGALS1,FMNL2,ETV4,SPRY4,DSG2,GCNT1,PTPN12,NEDD1,ADAM19,PLOD2,FNDC3B,C1QA,TIMP1,PDIA4,FANCI,MT1L,VCL,IL11,SERPINH1,PSMD13,FAM3C,HLA-E,DOCK4,SSFA2,LPXN,SLC7A6,TPST1,ALCAM,PSMB4,MYH9,FKBP15,CAB39,PKP1,CEACAM3,PSD3,FAM129B,ABCA2,AGPAT9,CADM3,DKK2,AOC3,AOX1,ZNF350,FAM84A,TSPAN12,TLR5,DCN,GSTO2,DIO2,ISL1,PLEKHG6,TP53INP2,BDNF,ARHGEF26,ASAP3,LGR4,ADAMTS18,CADM4,RGS5,SPRR1A,MIA,SORT1,TNFRSF19,STAB2,CRAT,PRELP,TRIM29,JAM2,RAB25,HBA1,LPHN3,ST6GALNAC1,EYA2,CRYAB,EMP1,HRASLS,GSTA3,ZNF185,HBB,SH3GL3,SELP,BNIPL,CEACAM5,OGN,CEACAM7,MUC15,KRT13,KRT4,TGM3

Therapeutic#Prognostic 1 FSCN1,OSMR,VSIG1,SAMSN1,RARRES1,ISX,VWA5A,CBR1

Diagnostic#Angiogenesis#Apoptosis#Metastasis#CellProliferation#Inflammation 1 FST,CD34

Prognostic#CellProliferation 1 INHBA,FEZ1,IFITM1,ADAMTS12,TPX2,CENPF,KIF4A,TAP2,HOXA1,CCNB2,CBX7,C2orf40,NEGR1,PLP1

Metastasis#CellProliferation#Inflammation 1 MMP3

Inflammation 1 TST

Metastasis#CellProliferation 1 PTPRZ1,COL4A1,ITGA5,PMEPA1,PANX1,TRIO,TRIM22,NRAS,ADAM10,ZYX,COL7A1,LPP,SYNPO,TIMELESS,SH3GLB2,TPCN1,IL17RD,ZG16B,CYP11A1,DUOX1,ADAM33,MYB,DSC2,DARC,MAPK3,S100A14,IKZF2,TGFBR3,LRRC4,VIT,DKK4,ELF5

Apoptosis#Metastasis#CellProliferation 1 CYP27B1,DFNA5,LRP12,F2RL2,LIMS1,BCL2A1,ERBB3,DPEP1,GLUL,TSPYL5,FRZB,KRT1

Therapeutic#Prognostic#Diagnostic#Apoptosis#CellProliferation#Inflammation 1 CFTR

Therapeutic#Prognostic#Apoptosis#Metastasis#CellProliferation#Inflammation 1 TAP1,CYP3A4

Diagnostic#CellProliferation 1 CLSPN,CBFA2T3,DBI

Prognostic#Diagnostic 1 HMGA2,CAV1,FAM83A,SCD5,TACSTD2,KRT20,BCL11A,FGF12,CHRNA3

Therapeutic#Diagnostic#Apoptosis#CellProliferation#Inflammation 1 HMGCR

Therapeutic#Prognostic#Apoptosis#Metastasis#Inflammation 1 IL6

Therapeutic#Prognostic#Diagnostic#Angiogenesis#Apoptosis#Metastasis#CellProliferation 1 IGF2BP2,SULF1,CCNA2,GREM1,CCND2,MS4A1,NTRK2,TF,SLIT3,ABCB1,SFRP4,DLEC1,KLK1,IGFBP2

Therapeutic#Metastasis#CellProliferation 1 IFIT2,NRIP1,RTP3,FAP,NUDCD1,TINAGL1,BACH1,PTP4A1,CXCL2,BCL9,MAP2K6,PLD1,ICOSLG,CAMK1D,NR3C2,ART4,PDGFD,BARX2,FUT3,HOPX

Therapeutic#Angiogenesis 1 NELL2,NRP2,CYP2C18

Therapeutic#Prognostic#Diagnostic#Angiogenesis#Apoptosis#Metastasis#CellProliferation#Immortalization#Inflammation 1 FOXM1,TP53

CellProliferation#Inflammation 1 PLA2G7,UBASH3B,PTGS1

Prognostic#Metastasis 1 PLAU,MMP13,SEMA3C,LAMC2,LAMA3,CCL20,ITGA1,OCIAD2,IL1RL1,RGS1,CYR61,APOBEC3A,MMP14,FXYD5,THBS1,SCRN1,MYBPH,SRGN,PTGIS,FUT7,LIFR,PLCD1,HLA-DQA1,SEMA3G,ADAM23,CNKSR3,UPK1A,SLURP1,CLDN7,DSG1

Therapeutic#Prognostic#Diagnostic#Angiogenesis#Metastasis#CellProliferation 1 ESM1,VEGFC,UBE2C,IGF2BP3,CMA1

Therapeutic#Prognostic#Diagnostic#Metastasis 1 MUCL1,STIL,RGS22,LYPD3,TEX101,KLK13

Therapeutic#Diagnostic#Apoptosis#Inflammation 1 GPRC5A

Prognostic#Diagnostic#Apoptosis#CellProliferation 1 PITX1

Apoptosis 1 CXCL9,IFI6,SLCO1B3,TLR2,FCGR3B,PDE7A,DOCK5,STK17A,IFIH1,PI3,CFB,EXO1,MMP11,PLXNA1,APOL2,BIRC2,ELF4,TGFB1,HENMT1,QSOX2,PAG1,ANKRD1,MICU1,PLEK,TBP,BCL2L11,MPHOSPH6,LDHD,NPBWR2,APBB1,CTNNBIP1,IRX5,SNCAIP,CMTM5,MAS1L,RHOV,NRTN,KLK7,NOS1,TPPP3,CYP2J2,PADI1,AQP3,TRPS1,LDOC1,FETUB,MAP1LC3A,DNASE1L3,RAET1E,FAM3B

CellProliferation 1 AIM2,SAMD9L,IL12RB2,CDK6,LIMA1,RIPK2,XDH,CCL3L3,RBP1,ADAM12,SLC7A5,IL1B,ODC1,PTK2,TGFA,IL1A,CDKN3,ZWINT,BUB1,P2RY6,GLI3,TLN1,AJUBA,MARVELD1,FSTL3,ACER3,FRK,NUF2,ASCC3,SKA3,IL23A,SKA2,UBE2T,HIST1H2AL,NCOA1,PDZD4,FAM43B,SOX5,GALR1,NCK2,ND6,EFHD1,SIX3,TSC22D3,PDIK1L,MPZ,PARD6A,POU2AF1,HBD,GAL3ST1,AQP5,CYP7B1,ZBTB7C,TEC,APPL1,NPDC1,SLC9A3,SPRR1B,PPARG,MLPH,TCEA3,CYP3A5,VASN,KLK10,CALML3,HBG1,FAM107A,PCSK2,CSTB,FUT6,GULP1,PAX9,NPPC,SPRR3

Therapeutic#Angiogenesis#Apoptosis#CellProliferation 1 IL24,CAPN6

Prognostic#Angiogenesis#CellProliferation 1 KRT17

Therapeutic#Prognostic#Diagnostic#CellProliferation 1 TFRC

Angiogenesis#Metastasis 1 WARS,POSTN,CALD1,PPFIA1,CLEC3B

Therapeutic#Inflammation 1 S100A7A

Therapeutic#Diagnostic 1 CEP55,FCGR2A,PYGB,CD79B,SOX10,UGT1A6,EHF,XIST

Therapeutic#Prognostic#Angiogenesis#Metastasis 1 SDC3,AMOT,AQP1

Angiogenesis#Apoptosis#Metastasis#CellProliferation 1 THBS2

Prognostic#Angiogenesis#Metastasis 1 SMPD3

Therapeutic#Apoptosis#CellProliferation#Inflammation 1 EIF2AK2,HSPB8

Therapeutic#Prognostic#Diagnostic#Apoptosis#Metastasis#CellProliferation 1 TNFRSF12A,KIF14,IFI27,RAD51,PML,CDC25B,SOX9,HMMR,CASP7,CHEK1,BRCA2,CALB2,CCNE1,EGFR,APAF1,SELL,UCHL1,KCNH1,CADM1,PSCA,NOTCH3,TMPRSS2,NTRK3,CEACAM6,HSPA1A,MAPT,TYR,PMEL

Angiogenesis#Metastasis#CellProliferation#Immortalization 1 PBX1

Therapeutic#Prognostic#Metastasis 1 CA9,BATF2,MSN,LOXL2,FLNA,PSMA7,RHOC,EFNB1,NR0B1,CRABP1,MACC1,SOST,SDR9C7

Prognostic#Diagnostic#Angiogenesis#Metastasis#CellProliferation 1 NRG1,PDGFA

Therapeutic#Prognostic#Diagnostic#Apoptosis#Metastasis 1 XAF1,FBLIM1,TIMP4,MAL

Therapeutic#Angiogenesis#Metastasis#CellProliferation#Inflammation 1 IL1RN

Angiogenesis 1 JHDM1D,EMCN,DDAH2,CYP2C19,TFAP2B

Therapeutic#Prognostic#Apoptosis#CellProliferation#Immortalization 1 AATF

Angiogenesis#Metastasis#CellProliferation 1 AGRN,CCDC6,SOSTDC1,TM7SF2

Therapeutic#Prognostic#Diagnostic#Angiogenesis#Metastasis#CellProliferation#Inflammation 1 CXCL5,CTGF,ELN,SELE

ImmunoModulation 1 MIR31HG,HTR7,C3orf52,LOC440354,AOC4,MIR210HG,DDX11L2

Diagnostic#Apoptosis#Metastasis#CellProliferation 1 STC1

Prognostic#Angiogenesis 1 CEACAM1

Therapeutic#Apoptosis 1 DDX58,AURKA,MAGEA4,GLIPR1,CCL8,NFIL3,ZHX2,KIF11,NAPB,DUSP4,KIF20B,TGIF1,CHMP5,IL17RB,SSTR4,KCNIP3,DACT3,LGR6,GDA

Angiogenesis#Apoptosis#Metastasis 1 IL20

Prognostic#Diagnostic#CellProliferation 1 NOP2

Prognostic#Apoptosis#CellProliferation 1 FAIM3,CDK1,GALR2

Therapeutic#Angiogenesis#Apoptosis#CellProliferation#Inflammation 1 CXCL11

Therapeutic#Prognostic#Angiogenesis#Apoptosis#Metastasis#CellProliferation 1 AREG,IL32,SLC3A2,PAK1,GAB2,SPRY2,MMRN1,ANGPT4,PTN

Apoptosis#Metastasis#CellProliferation#Inflammation 1 DUSP6

Therapeutic#Diagnostic#Metastasis#Inflammation 1 FBN2

Prognostic#Inflammation 1 TREM1,NCF2

Apoptosis#CellProliferation 1 LY6E,OAS3,TNFRSF10B,IRF9,EIF2S1,RARRES3,ITPR3,STK3,KIF20A,PRKCQ,SPECC1,RAPGEF1,RPS6KA6,ITPR2,RORC,CYP4F3,NDN,SMPD2,MGLL,DEPTOR,AADAC,SCNN1A,HLF

Prognostic#Diagnostic#Apoptosis 1 OSGIN1

Diagnostic#Angiogenesis#CellProliferation 1 APLNR,KRT15

Prognostic#Apoptosis#Metastasis#CellProliferation 1 ECT2,EXT1,CYP26A1,NLRP2,CCL19,SASH1,GPX3

Therapeutic#Diagnostic#Apoptosis#Metastasis#CellProliferation 1 CCNA1,IFIT5,CD70,PRIM2,TYRO3,GDF10,PTGDS

Therapeutic#Prognostic#Apoptosis#Metastasis#CellProliferation 1 CD274,CTLA4,LYN,TNFRSF9,TSPAN1,PROCR,NBN,MYBL2,SHC1,CKS2,IGF2BP1,MAD2L1,RBBP8,ADAM9,NUPR1,CACNA2D3,DLX4,TRPV6,EDNRB,DIRAS3,MXI1,SLC9A3R1,BMP7,RORA,PRLR,IGFBP5,DCT

Therapeutic#Diagnostic#Angiogenesis#Apoptosis#Metastasis#CellProliferation#Inflammation 1 ABCA1

Metastasis#ImmunoModulation 1 GNG7,PGD

Apoptosis#Inflammation 1 OAS1,FOXE1

Prognostic#Diagnostic#Apoptosis#Metastasis#CellProliferation 1 TGFBI,CCL3,ITGA2,ELANE

Therapeutic#Prognostic#Diagnostic#Apoptosis#Metastasis#CellProliferation#Inflammation 1 IL2RA,IRF1,SEC14L2,WNT5A,CD79A,ACPP

Diagnostic#Metastasis#CellProliferation 1 ANO6,FGF23,KRT10,EPHX2,CLCA4

Therapeutic#Diagnostic#Angiogenesis#Metastasis#CellProliferation 1 MAP2,ECM1

Therapeutic#Metastasis#CellProliferation#Inflammation 1 KIF18A

Diagnostic 1 S100P,ATP6V1C1,STAT1,XRCC2,ADA,SLC38A1,GGH,DBP,PBOV1,AGSK1,MLANA,SERPINB11

Therapeutic#Prognostic#Diagnostic 1 DEPDC1,TSPAN7,ODAM

Therapeutic#Apoptosis#CellProliferation 1 HOXC9,NDC80,BLM,ATIC,RECQL,GZMA,PRDM14,AMPD1,MMAB,CRYM,FGFR2,EVPL,BCL2L10,CYSLTR1,P2RY1

Therapeutic#Angiogenesis#Apoptosis#Metastasis 1 NELL1

Therapeutic#Angiogenesis#Apoptosis#Metastasis#CellProliferation#Inflammation 1 F3,F2RL1,PTGES,MAP3K1

Therapeutic#Diagnostic#Apoptosis#CellProliferation 1 MCL1,PIM1,SMO

Diagnostic#Metastasis 1 ITGA3,LTBP1,TNFAIP3,PDPN,CLDN5,LLGL2,ACSBG1,PDZD2,ATP1B2,KRT19

Therapeutic#Apoptosis#Metastasis#CellProliferation#Inflammation 1 PSMB9,CCL18,JUN,CCL21,P2RY2

Diagnostic#Apoptosis#CellProliferation 1 HELLS

Therapeutic#Prognostic#Metastasis#CellProliferation 1 PTHLH,MELK,WNT7A,PLAUR,VMP1,PICALM,HAVCR2,SMURF2,NUAK1,TK1,NME1,PKP2,LAMB3,SLC5A5,ITIH5,NNAT,ANKS1B,CRABP2,WISP2,APOD

Therapeutic#Prognostic#CellProliferation 1 PDCD1LG2,GALNT6,ATAD2,HJURP,SLC22A1,APLN,ASPM,CENPE,DLX5,PVRL4

Prognostic#Diagnostic#Metastasis#CellProliferation 1 MKI67,CDC6,IVL

Prognostic#Angiogenesis#Metastasis#CellProliferation 1 MMP7,MET,ETS1,ITGA6,EFNA2,TSPAN8

Therapeutic#Prognostic#Angiogenesis#CellProliferation 1 KIF2C

Therapeutic 1 IDO1,PLAC1,SLC16A1,CD80,PHLDB2,SERPINE2,PLSCR1,IL13RA2,MX2,SMS,LEPREL1,STK10,ANTXR2,CDCA8,PPM1D,SPATA19,PSORS1C2,ABCA7,NCAM2,SMR3A,CNTFR,MTUS1,C10orf90,MS4A2,CDON,MKNK2,CP,ABCA3,LBP,ASPA,HBA2,NUAK2,FCER1A,GSTA1,CYP4B1,TMPRSS11E,PPL,CHL1,TMPRSS11A

Therapeutic#CellProliferation 1 STAT2,TTK,RTKN,SOCS3,KIF23,ACSL5,GINS1,IFNG,CDCA3,NCAPG2,CIT,PRSS23,ATOH1,FGFR3,RAB11FIP1,TNFRSF17,LGALSL

Therapeutic#Apoptosis#Metastasis#CellProliferation 1 IFI44,SOD2,CSF2,IL7R,APOBEC3B,CEBPB,EPHB2,GLS,NUP37,XCL1,SLC9A1,RAC2,HOXB9,SKIL,CYP24A1,ACLY,PLAGL1,MAPK4,WNT2B,CYTL1,PEBP4,KAT2B,ASAH1,FOXO4,DUOXA1,BTC,MAPK7,GAS1,NUCB2,RARG,GNRHR,TGM1,ROR1

Therapeutic#Angiogenesis#Apoptosis#Metastasis#CellProliferation 1 ITGAV,LPAR3,CD86,COL4A2,RHOB,BRMS1,BEX2,ANGPTL1

Therapeutic#Angiogenesis#Apoptosis 1 E2F7,APP

Therapeutic#Diagnostic#CellProliferation 1 KIR3DL2,SNED1

Therapeutic#Prognostic#Diagnostic#Angiogenesis#Apoptosis#Metastasis#CellProliferation#Inflammation 1 CXCL10,SERPINE1,ADM,CD14,SPHK1,GAST,CCL11,ANG,PLA2G1B,AGT,POMC,TEK,CXCR2,CLU,CXCL12

Therapeutic#Prognostic#Apoptosis 1 UBD,ABCC3,IL1RAP,SLC5A1

Prognostic#Apoptosis 1 TPM1,TRIB1,PHLDA1,ATG5,BAK1

Prognostic#Diagnostic#Metastasis 1 FN1,KIT,TRPM1

<<<<<<<<<<<<<<<<<<<<<<<<<<<<<<<<<<<<<<<<<<<<<<<<<<<

<<<<<<<<<<<<<<<<<<<<<<<<<<<<<<<<<<<<<<<<<<<<<<<<<<<

Total No. of Genes Differentially expressed :2290

Total No. of Genes Annotated :1014

Total no. of Genes Significantly Annotated: 841
