# Supplementary material for: Potential Therapeutic Targets for Oral Cancer: ADM, TP53, EGFR, LYN, CTLA4, SKIL, CTGF, CD70
Source: PLoS One. 2014 Jul 16;9(7):e102610. doi: 10.1371/journal.pone.0102610 (PMC4110113; doi:10.1371/journal.pone.0102610)
Supplement: Text S11 — Detailed report of significant annotations by literature mining method. (DOCX) [file pone.0102610.s011.docx]

Literature Mining Statistics - Statistically Significant Annotations

----------------------------------------------

Total no. of genes annotated: 841

Genes related with oral cancer : 147 PFN2,CD44,SFRP4,NMI,AURKB,CDK1,SEMA3A,KRT19,CD34,IFNG,SLC6A2,TNFSF10,PDPN,CEACAM1,DCN,CD151,CD163,PLAU,PTHLH,KRT20,IDO1,SAMSN1,TP53,MAP1LC3A,TPX2,MMP7,S100P,PXN,TLR2,MMP13,CHL1,CRAT,PSMB8,HSPB2,PAG1,CCNB1,NOP2,CDKN2A,TGFB1,TACSTD2,MRPL28,TNC,TFRC,LDHD,MMP3,BAK1,MET,ZYX,CXCL9,CAV1,THBS1,JUN,HOXA1,BIRC2,SOCS3,VEGFC,MSN,MLANA,MMP10,FCER1A,CD24,TXNRD1,FCGR3B,LAMC2,PHLDA1,NELL2,EPHB2,AR,SNAI2,MTUS1,NRP2,FGFR2,SOX10,IL8,FAT1,EPS8,PLAUR,NME1,PI3,KIT,FGFR3,BCL2L11,HLA-A,ATG5,ETS1,EPHA7,ABL2,APLN,CDC6,NOS1,CYR61,CP,TNFAIP3,ETV4,TNFRSF10B,CCL20,MCL1,SPP1,CD80,PYGB,SLC16A1,KLK7,CA9,MMP1,POSTN,ADA,STAT1,PDZD2,CTSL1,ZNF135,IL6,RHOC,BIRC5,ANXA5,PPM1D,MMP14,AURKA,SLURP1,CLDN7,EGFR,KRT13,HMGA2,BCL2A1,MT1L,IL1B,FN1,BUB1B,S100A7,MS4A2,TPM1,ISG15,ATP6V1C1,FSCN1,ODC1,FXYD5,LGALS1,IFIT3,ANGPT2,TMPRSS11A,BIRC3,MMP9,AQP3,CBFA2T3,FADD,MMP11,FIGF,IFI27

Genes related with cancer : 694 QSOX2,HMGCR,SMR3A,ABCA3,XCL1,CDH3,LGALSL,C10orf90,STK10,DACT2,PTN,ITGA5,TGM1,NR3C2,CYP26A1,APOL2,EMCN,CCNA1,TK1,NBN,PANX1,DIRAS3,CADM4,HLA-F,GGH,GSTA1,MPPED2,EIF2AK2,DBP,CCL11,CXCL3,RGS5,PAK1,HSPB8,PRDM14,COL4A2,EHF,PACRG,ARSF,UBASH3B,FCGR2A,WISP2,TRIO,PEBP4,ITIH5,IGF2BP3,LGR6,PADI1,RTP3,EXT1,RECQL,UPK1A,MKNK2,MS4A1,SOD2,DIRAS1,PML,F2RL2,GDA,BATF2,IGF2BP2,CEACAM6,CENPE,IRF1,IL20,LPAR3,ASPM,CCL18,LMNB1,CRABP2,CAPS,MGLL,SLCO1B3,PMEL,PALM,RORA,SASH1,IL17RD,BRCA2,OCIAD2,IL2RA,CALB1,OGN,FAP,LY6K,PLEK,TTR,CLCA4,CLDN5,DKK2,PTGDS,BST2,SHC1,KCNIP3,STX1A,IRX5,ITGA3,LYN,DUSP6,CDC25B,PTPN12,INHBA,CTNNBIP1,CDCA3,EFNB1,GINS1,SERPINE2,SOX21,NTRK2,C5AR1,CYP2C19,IFI6,SMO,AGRN,BTC,CRYM,AOC4,LOXL2,ANXA3,ATAD2,CTSG,ELANE,ATOH1,TEK,NUCB2,AADAC,CLIC3,PSMB7,RAC2,CCL3L3,HTR1B,ID4,ISL1,PLA2G1B,P2RY6,SLC5A5,NFIX,CYP27B1,CD1A,OCLN,ANLN,UCHL1,EPHX3,GZMA,CDON,SELL,FBN2,SLC16A7,CHMP5,TAP2,CCL19,OSGIN1,CXCL2,KLK13,GALNT6,DCBLD2,STIL,VWA5A,LIFR,UBE2C,STK17A,FOXE1,HTR7,TNFRSF9,CCNE1,FBLIM1,MXI1,DUSP4,NCAM2,CD79A,S1PR1,MSMB,BRMS1,HOXD10,CYSLTR1,ISM1,HENMT1,FOXM1,NLRP2,NELL1,DACT3,TRPV6,ACSBG1,CTLA4,ANGPT4,CCNB2,HOXC9,BCL2L10,CYP2C18,ALDH3A1,CNKSR3,PLCD1,IFIT5,F2RL1,MIR31HG,OSMR,FUT3,GDF10,DBI,IFIT1,LAMP3,ABCA1,PDGFA,CCL8,ZHX2,RARRES3,PLXNA1,DNASE1L3,LLGL2,CHEK1,SLC38A3,WNT7A,TSPAN8,RHOB,MUCL1,KIF11,GBP1,CEP55,MPHOSPH6,IL24,XIST,CLU,BDKRB1,CXCL12,MYH9,NUP37,TRPM1,LBP,TMPRSS11E,NCF2,GLUL,PLSCR1,ADAM19,TF,GUCY2C,FEZ1,APOD,NNAT,BARX2,GABRP,CX3CL1,BCL9,RBBP8,COL14A1,ADAM23,CXCR2,APBB1,CBX7,IRS1,TSPAN7,MICU1,MTAP,BAMBI,CD19,HPRT1,HELLS,TNS4,NR0B1,LTBP1,GNRHR,PSMA7,C3orf52,PLAC1,JAM2,DLX5,CCM2,LRRC4,TAP1,SMPD3,CNTFR,NRIP1,SLC9A1,PDE7A,AFAP1L1,THBS2,PLEC,DIO2,ITGA1,PRSS23,AGT,SLC3A2,PTGES,MAPK13,SCD5,TEC,MAL,RGS22,PLD1,COL7A1,NFIL3,LOC440354,APLNR,TFAP2B,CLEC3B,MMAB,PLA2G7,NUPR1,MACC1,SLC1A5,CXCL11,PICALM,MCM4,ANTXR2,KIF18A,MYBL2,HLF,MAP2,SPRY2,MAPT,IL1RL1,KIR3DL2,APOBEC3B,PRLR,SLC9A3R1,CFTR,CAMK1D,ASAH1,TST,ECM1,DDX11L2,SIX3,CD70,SAA1,KIF14,KRT15,DLX4,SMPD2,SELE,PBX1,ABCB1,KRT17,GNG7,TIMELESS,MAD2L1,DLEC1,SPATA19,RAB11FIP1,LRP12,IL1RN,PLAGL1,SEC14L2,SDC3,PIM1,IFIT2,FAIM3,CADM1,MUC15,KLF4,PRIM2,DPEP1,ISX,HJURP,MAP2K6,AQP1,ASAP3,PLEKHA2,RPS6KA6,CYP17A1,CMTM5,IL7R,MYBPH,TSPAN1,GAS1,TM7SF2,IFI16,FAM84A,GALR2,CXCL10,CCL21,CAPN6,NUAK1,PPL,LAMB3,TYR,VIT,LCP2,RNASEH2A,SERPINE1,KDM1A,CCDC6,VMP1,EFNA2,MKI67,RARG,SLC22A1,GLS,BACH1,IL13RA2,DOCK5,PITX2,SOX9,CRABP1,STAT2,TGIF1,CLSPN,PTAFR,CD1E,PITX1,P2RY1,ODAM,OAS1,NDRG2,FETUB,ANKS1B,PSORS1C2,SDR9C7,RAPGEF1,GLIPR1,STC1,ASAP1,SEMA3G,SULF1,MEIS1,KIF20B,LTB4R2,TGFBR3,SPINK6,VSIG1,CCR2,CMA1,PDGFD,RAD51,CIT,FST,BEX2,FAM3B,BLM,SLC38A1,ITGB1,IL32,CCL14,SCRN1,PPFIA1,VLDLR,SDC4,HMMR,WISP3,KIF4A,PRKCQ,ELN,WARS,NCAPG2,SNCAIP,DCT,ELF5,CHRNA3,LGALS3BP,CXCL5,ASPA,ITGAV,ABCC3,HOXB9,RARRES1,DUOXA1,PITPNM3,SCNN1A,NAPB,FABP4,EXO1,JHDM1D,SOSTDC1,EDNRB,TYRP1,CCND2,SLIT3,EIF2S1,CFB,DDX58,TNFRSF17,RSPO1,MAF,ABCA7,RTKN,FRZB,CASP1,ADM,SPHK1,SLC5A1,XAF1,SFTPD,ADAM9,APAF1,AMPD1,WNT5A,IFIH1,KRT10,NUAK2,BMP7,ARG1,KIF2C,GPX3,APOBEC3A,KIF20A,NOV,DDAH2,NEGR1,LYPD3,KLK1,FNDC3B,SNRPB,P2RY2,FGF12,ICOS,GPRC5A,STK3,ELF4,CAV2,TGM3,PTP4A1,CYP2C9,CCL15,ITGA6,DKK4,IGFBP2,BMX,ATP1B2,PRKDC,SPINK7,CCL4,PDCD1LG2,CALD1,RRAS,POMC,SKIL,BCL11A,SOST,ACLY,HSPA1A,S100A7A,MMRN1,SMS,MAPK7,ST3GAL5,IL17RB,TPPP3,CALB2,CD79B,LY6D,RHOV,ANKRD1,PTK6,TNFSF18,PTGS1,SPECC1,PMEPA1,MELK,F3,CFP,FUT7,IL1RAP,TREM1,SRGN,CASP7,TRIB1,TSPYL5,IFNAR2,CYB5A,TIMP4,AATF,KLK12,CDC20,CX3CR1,DSG1,ACSL5,ANG,UGT1A6,AGSK1,PLP1,ERRFI1,MLLT11,PRSS3,NPBWR2,TBP,ACPP,SNED1,LIMS1,RAET1E,MAS1L,NDC80,KLK11,AREG,CIDEB,TINAGL1,PBOV1,GLI3,ITGA2,LIMK1,IFITM1,KIAA0101,UBD,PSCA,CEBPB,FMNL2,TRPS1,GAST,GREM1,ITPR2,CCL3,AMOT,FZD10,LDOC1,KRT31,NTRK3,NOTCH3,RBP1,ART4,ATIC,CCNA2,NRG1,MALL,ROR1,TTK,LEPREL1,TGFBI,TYRO3,IVL,HAVCR2,PHLDB2,PGD,CDKN3,ZG16B,E2F7,MAGI1,CYP2J2,MAP3K1,LAMA3,NUDCD1,GAB2,CD86,FLNA,C2orf40,TEX101,CD274,TNFRSF12A,SERPINA5,CYP24A1,EVPL,ANO6,SELENBP1,PVRL4,PROCR,SSTR4,KRT81,PKP2,DARC,PDIK1L,TYMP,HLA-DQA1,CFD,CYP3A4,IGFBP5,TMPRSS2,ZAK,NRTN,CTGF,SEMA3C,EPHX2,KAT2B,CYP4B1,APP,IFI44,CENPF,IGF2BP1,FGF23,CKS2,CXCL17,ANGPTL1,PTGIS,RGS1,MIR210HG,ADAMTS12,MX2,CD1C,HBA2,PSMB9,MAGEA4,AJAP1,CACNA2D3,CSF2,CBR1,ITPR3,ECT2,KIF23,DEPDC1,GNLY,CCL13,AGPAT9,FAM83A,SERPINB11,CD14,CDCA8,XRCC2,ESM1,KCNH1

>>>>>>>>>>>>>>>>>>>>>>>>>>>>>>>>>>>>>>>>>>>>>>>>>>

Annotations or Biological Concepts detected

###########################################

Annotation No. of genes GeneList

--------------------------------------------

Metastasis 207 MMP1,MMP10,MMP3,CXCL10,PTHLH,PLAU,SERPINE1,SPINK6,TNC,TNFRSF12A,SAA1,MET,IFI44,PDPN,CDH3,ITGA5,LY6K,HOXD10,FN1,ETS1,F3,WISP3,FAT1,VEGFC,CTSL1,PANX1,SNAI2,IL32,ECT2,ITGAV,TRIO,SDC4,LGALS1,BST2,FOXM1,FMNL2,PLAUR,ETV4,MMP9,PML,SULF1,LGALS3BP,FAP,TSPAN1,NUDCD1,APOBEC3A,DCBLD2,PTPN12,HMMR,CASP7,ITGA2,APP,VMP1,CDKN2A,F2RL1,CHEK1,ITGB1,SLC3A2,LAMP3,F2RL2,CXCL5,ADAM19,PAK1,NBN,RHOB,LOXL2,ASAP1,FNDC3B,ABCA1,ITGA6,IL6,IGF2BP3,BRCA2,SHC1,COL7A1,CD44,CALB2,MT1L,BRMS1,CKS2,ST3GAL5,SPHK1,CTGF,PTP4A1,CALD1,NUAK1,ZAK,SDC3,SELL,PSMA7,RHOC,CD151,EFNB1,PPFIA1,NME1,LIMK1,ADAM9,GAST,RAC2,LAMB3,HOXB9,UCHL1,MYH9,BCL2A1,THBS2,ITIH5,WNT5A,CD1E,MAP2K6,CLDN5,NTRK2,CCL21,SERPINA5,AGPAT9,LTB4R2,PEBP4,GAB2,DKK2,MSMB,FAM84A,ZG16B,DPEP1,AGT,CYP17A1,ANKS1B,DCN,ELANE,DIO2,KRT31,POMC,CADM1,PDZD2,ISL1,PLD1,ELN,ASAP3,FUT7,PSCA,PITPNM3,NOV,SLC38A3,MAPK7,CADM4,NDRG2,DLX4,SLIT3,CXCR2,RGS5,TTR,MMRN1,EDNRB,GNRHR,CCR2,TMPRSS2,TIMP4,CTSG,NTRK3,ACPP,DIRAS3,ADAM23,CRAT,CLEC3B,DARC,MACC1,DLEC1,CLU,CEACAM6,JAM2,CX3CR1,PRSS3,OCLN,NFIX,KLK1,CCL15,PTGDS,LYPD3,TGFBR3,WISP2,CCL19,LY6D,SASH1,TSPAN8,AR,KLF4,ECM1,BMP7,TEX101,BARX2,CLDN7,PTN,DKK4,FUT3,CXCL12,IGFBP2,FIGF,FRZB,SELE,DSG1,OGN,GUCY2C,TYR,PMEL,TYRP1,MUC15,KRT13,SPINK7,TGM3

Diagnostic 160 CXCL10,MUCL1,FST,TNC,KIF14,HMGA2,CAV1,ITGA3,LTBP1,IL2RA,TNFAIP3,XAF1,CEP55,LY6K,IRS1,FN1,STC1,FAT1,VEGFC,ICOS,PDGFA,MTAP,FAM83A,S100P,FOXM1,ATP6V1C1,RAD51,MMP9,CDC25B,FBN2,FABP4,STAT1,UBE2C,MCM4,IFIT5,CDKN2A,CLSPN,CALB1,XRCC2,GNLY,ADA,CDC6,DEPDC1,CXCL5,FCGR2A,SLC38A1,PLEC,ABCA1,AURKB,CD14,HELLS,IGF2BP3,CALB2,TFRC,GGH,CTGF,FBLIM1,ERRFI1,EGFR,STIL,CCND2,SCD5,CD70,ANO6,PRIM2,BIRC3,GAST,UCHL1,MCL1,NOP2,PYGB,CAPS,RGS22,WNT5A,OSGIN1,CD1E,CD79B,APLNR,MS4A1,KCNH1,CLDN5,FGF23,CBFA2T3,TYRO3,GPRC5A,TACSTD2,LLGL2,CD34,HTR1B,TF,KRT20,MAPK13,ACSBG1,DBI,BCL11A,TP53,S1PR1,KRT10,DBP,CD79A,KIR3DL2,SOX10,POMC,CADM1,PDZD2,ELN,PSCA,MYLIP,SLC38A3,SNED1,CD1C,CD19,UGT1A6,SLIT3,ABCB1,TTR,FGF12,MAP2,SFRP4,TMPRSS2,KIT,ATP1B2,ACPP,DLEC1,CLU,CEACAM6,TRPM1,SFTPD,PBOV1,KLK1,CD1A,AGSK1,CHRNA3,AR,MAF,TSPAN7,ECM1,TEX101,EHF,KRT15,EPHX2,MAPT,PITX1,KRT19,IVL,CXCL12,IGFBP2,ARG1,SELE,GUCY2C,ALDH3A1,MLANA,KLK13,TYR,ODAM,PMEL,CLCA4,XIST,SERPINB11,MAL

CellProliferation 185 CXCL10,FST,CYP27B1,IL2RA,IFI44,TAP1,EIF2AK2,ITGA5,MKI67,FEZ1,NRG1,MELK,IRS1,AREG,CTLA4,PMEPA1,LYN,IGF2BP2,SOD2,CSF2,TPX2,CCL3,CCNA1,PDGFA,ECT2,NDC80,TNFRSF9,IL7R,TRIO,CCL3L3,STAT2,IFI27,FOXM1,ATAD2,ADM,PML,SULF1,RBP1,TSPAN1,FABP4,NUDCD1,EIF2S1,CCNA2,SOX9,UBE2C,GREM1,IL1B,ODC1,EXT1,ZYX,F2RL1,CLSPN,SLC3A2,CD86,CXCL5,PAK1,MYBL2,SLC6A2,RHOB,GINS1,CENPF,ABCA1,BLM,CDKN3,CD14,BRCA2,SHC1,KDM1A,P2RY6,ITPR3,GLI3,TFRC,SPHK1,CTGF,CCNE1,KIF20A,EGFR,CCND2,CENPE,TK1,CD70,IGF2BP1,ATIC,KIAA0101,RBBP8,PKP2,HPRT1,GAST,CCNB2,TIMELESS,SKIL,CXCL2,CYP24A1,RNASEH2A,ANG,ATOH1,WNT5A,NUPR1,NNAT,HMGCR,MS4A1,KCNH1,SIX3,CBX7,NTRK2,CCL21,ID4,PDIK1L,DLX5,GAB2,CBFA2T3,RPS6KA6,TYRO3,MAGI1,IL17RD,GALR2,TF,SPRY2,MAPK13,AGT,TP53,AMPD1,ANKS1B,CD79A,KAT2B,POMC,TNFRSF17,TEC,GLUL,CCDC6,MYLIP,NOV,BTC,TSPYL5,MAPK7,CD1C,PALM,CD19,GAS1,NUCB2,NOTCH3,RARG,SLIT3,TRPV6,CXCR2,HSPB8,GNRHR,SFRP4,NTRK3,EVPL,DIRAS3,DLEC1,PTK6,CLU,MXI1,NR3C2,P2RY2,PTGDS,CD1A,MEIS1,WISP2,MGLL,ART4,CCL19,APOD,SASH1,LRRC4,KLF4,VIT,SMO,ANGPTL1,RORA,PRLR,EPHX2,IL1RN,PTN,PITX1,IGFBP5,P2RY1,CXCL12,CYP3A4,IGFBP2,ELF5,DCT,TYR

Immortality 6 FOXM1,AATF,TP53,CD19,PTK6,PBX1

Prognostic 295 CXCL10,INHBA,IFIT3,PLAU,MMP13,SERPINE1,SEMA3C,TGFBI,SPP1,LAMC2,MUCL1,PDCD1LG2,CD274,GALNT6,TNFRSF12A,CA9,HMGA2,CAV1,TREM1,LAMA3,MET,NCF2,XAF1,CCL20,PFN2,ITGA1,TPM1,FEZ1,MELK,BATF2,AREG,FSCN1,ETS1,ANGPT2,WISP3,FAIM3,ESM1,LYN,SEMA3A,IGF2BP2,VEGFC,IFITM1,WNT7A,ANLN,OSMR,ADAMTS12,ISG15,OCIAD2,IRF1,TPX2,TRIB1,IL1RL1,PHLDA1,TNS4,MTAP,FAM83A,ANXA3,RGS1,BUB1B,IFI27,FOXM1,ATAD2,RAD51,PLAUR,MMP9,PML,HLA-A,CDC25B,LGALS3BP,CYR61,APOBEC3A,MMP14,FXYD5,CCNB1,MSN,SOX9,UBE2C,HMMR,HLA-F,THBS1,ITGA2,CDK1,AJAP1,VMP1,CDKN2A,CHEK1,PROCR,SLC3A2,HJURP,LAMP3,GNLY,TYMP,SLC22A1,SCRN1,AFAP1L1,CDC6,DEPDC1,CXCL5,PAK1,PICALM,MYBL2,SLC6A2,MYBPH,HAVCR2,CENPF,PLEC,KIF4A,AURKB,KIF2C,CD14,KRT17,ITGA6,GABRP,IGF2BP3,BRCA2,SHC1,CD44,ABL2,CKS2,TAP2,TFRC,APLN,CTGF,FBLIM1,CCNE1,NMI,MLLT11,CD163,UBD,EGFR,PSMB7,SRGN,NUAK1,STIL,APAF1,CCND2,SCD5,SDC3,ASPM,FLNA,CENPE,PSMA7,PSMB8,VSIG1,SEC14L2,TK1,RHOC,MAD2L1,SLC1A5,HOXA1,KIAA0101,ATG5,RBBP8,NME1,SAMSN1,CASP1,PKP2,RARRES1,BAK1,ADAM9,GAST,LAMB3,CAV2,UCHL1,CCNB2,NOP2,SNRPB,IL1RAP,MRPL28,PTGIS,ANG,SLC5A5,ISX,RGS22,ITIH5,WNT5A,OSGIN1,NUPR1,CD1E,HSPB2,NNAT,MS4A1,KCNH1,CBX7,NTRK2,SERPINA5,DLX5,TACSTD2,MAGI1,GALR2,EFNA2,SPRY2,KRT20,CACNA2D3,BCL11A,TP53,NR0B1,ANKS1B,CD79A,CMA1,BAMBI,POMC,CADM1,CYB5A,C2orf40,MALL,CYP26A1,EPHA7,FUT7,PSCA,LIFR,MYLIP,BDKRB1,CRABP1,CD1C,PALM,PLCD1,AMOT,NDRG2,VWA5A,NOTCH3,TEK,SMPD3,DLX4,SLIT3,SELENBP1,TRPV6,ABCB1,PVRL4,HLA-DQA1,FGF12,ANGPT4,NEGR1,EDNRB,CFD,CIDEB,TMPRSS2,KIT,TIMP4,CFTR,NTRK3,ACPP,CBR1,SEMA3G,ADAM23,CNKSR3,MACC1,PTK6,PACRG,CLU,MXI1,CEACAM6,TRPM1,SLC16A7,KLK1,SOST,LYPD3,CD1A,CEACAM1,CHRNA3,UPK1A,APOD,SASH1,TSPAN7,CD24,SDR9C7,KLF4,SLC9A3R1,BMP7,CLIC3,TEX101,SLURP1,CLDN7,SLC5A1,MAPT,PTN,PITX1,IGFBP5,COL14A1,CXCL12,IGFBP2,PLP1,SELE,KLK11,DSG1,PITX2,KLK12,DACT2,KLK13,CHL1,TYR,ODAM,PMEL,EPHX3,TYRP1

ImmunoModulation 10 MIR31HG,HTR7,C3orf52,LOC440354,AOC4,MIR210HG,GNG7,DDX11L2,PGD,TYRP1

Therapeutic 370 CXCL11,CXCL10,NELL2,S100A7A,IFIT1,IDO1,SERPINE1,IL8,SPINK6,PDCD1LG2,CD274,IFIT2,TNFRSF12A,IL24,KIF14,CA9,IL2RA,XAF1,TAP1,CEP55,PLAC1,EIF2AK2,SLC16A1,MELK,CD80,BATF2,AREG,FSCN1,AURKA,CTLA4,PHLDB2,F3,HOXC9,FAT1,ESM1,LYN,VEGFC,WNT7A,ICOS,E2F7,OSMR,IFNAR2,SOD2,IRF1,CSF2,SNAI2,CCNA1,MAGEA4,SERPINE2,GLIPR1,PSMB9,IL32,KIF18A,ITGAV,PLSCR1,NDC80,MTAP,TNFRSF9,CCL4,NRIP1,IFI27,FOXM1,RTP3,TXNRD1,ATAD2,RAD51,PLAUR,ADM,MMP9,GBP1,TTK,IL13RA2,PML,MX2,CDC25B,SULF1,RTKN,TSPAN1,NUDCD1,CCL8,CDC20,DCBLD2,APOBEC3B,PRKDC,MSN,CCNA2,SOX9,UBE2C,HMMR,APP,RRAS,VMP1,CDKN2A,CHEK1,LPAR3,SOCS3,SLC3A2,CEBPB,HJURP,CD86,GNLY,TYMP,SLC22A1,COL4A2,DEPDC1,CXCL5,PAK1,NBN,KIF23,FCGR2A,ACSL5,TINAGL1,HAVCR2,RHOB,GINS1,LOXL2,ZHX2,ASAP1,ABCA1,BLM,AURKB,EPHB2,KIF2C,TNFSF10,CD14,PXN,IGF2BP3,BRCA2,KDM1A,BRMS1,IFNG,TNFSF18,TFRC,GLS,SPHK1,PTAFR,NUP37,APLN,NRP2,STX1A,CTGF,KIF11,CCNE1,BACH1,PTP4A1,NAPB,CDCA3,SMS,XCL1,EGFR,NCAPG2,APAF1,CCND2,ASPM,FLNA,CENPE,SELL,LEPREL1,PSMA7,VSIG1,PTGES,CD70,KIF20B,RHOC,CD151,STK10,MAD2L1,EFNB1,CIT,ANTXR2,ATIC,KIAA0101,SAMSN1,ABCC3,PLEKHA2,RARRES1,BIRC3,PRSS23,TGIF1,ADAM9,GAST,CAV2,C5AR1,UCHL1,MCL1,CDCA8,RECQL,GZMA,IL1RAP,ACLY,PYGB,RNASEH2A,ANG,ATOH1,FGFR3,SLC5A5,ISX,CAPS,RGS22,PPM1D,PRDM14,WNT5A,ZNF135,ISM1,RAB11FIP1,BCL9,CD79B,HMGCR,MS4A1,KCNH1,NTRK2,CCL21,SPATA19,PLA2G1B,ID4,CX3CL1,IL17RB,LTB4R2,SSTR4,PEBP4,PSORS1C2,GAB2,TYRO3,MSMB,PIM1,HTR1B,TF,ABCA7,SPRY2,NCAM2,MAPK13,CACNA2D3,AGT,TP53,S1PR1,CYP17A1,AMPD1,MMAB,NR0B1,SMR3A,CD79A,MPPED2,CMA1,CNTFR,KAT2B,SOX10,ASAH1,KRT31,POMC,MTUS1,KCNIP3,EPS8,CRYM,DACT3,PSCA,C10orf90,MS4A2,MYLIP,CDON,PITPNM3,NOV,BMX,CRABP1,MAPK7,SNED1,CD1C,NELL1,CD19,MKNK2,NUCB2,VWA5A,UGT1A6,NOTCH3,TEK,LGALSL,FGFR2,DLX4,SLIT3,TRPV6,CXCR2,ABCB1,CP,LGR6,HSPB8,MMRN1,EDNRB,DIRAS1,GNRHR,ABCA3,SFRP4,LBP,ARSF,CCR2,CRABP2,TMPRSS2,TIMP4,CTSG,ASPA,CFTR,EVPL,ACPP,CBR1,DLEC1,PTK6,KRT81,CLU,HBA2,CEACAM6,CAMK1D,NR3C2,GDF10,NUAK2,PRSS3,NFIX,KLK1,PTGDS,LYPD3,CD1A,MEIS1,GDA,WISP2,RSPO1,LY6D,APOD,PDGFD,CAPN6,AR,SDR9C7,KLF4,BCL2L10,FZD10,SMO,BMP7,FCER1A,CYSLTR1,AQP1,EHF,RORA,GSTA1,PRLR,IL1RN,SLC5A1,MAPT,PTN,IGFBP5,SOX21,CXCL12,CYP3A4,IGFBP2,ARG1,ROR1,FIGF,CYP4B1,CCL14,SELE,TMPRSS11E,PPL,GUCY2C,ALDH3A1,DCT,CYP2C18,KLK13,TYR,ODAM,PMEL,TYRP1,XIST,SPINK7,TMPRSS11A,MAL

Angiogenesis 96 CXCL11,CXCL10,NELL2,SERPINE1,IL8,MMP7,IL20,WARS,AREG,ETS1,ANGPT2,F3,WISP3,ESM1,VEGFC,E2F7,IL32,ITGAV,FOXM1,ADM,SULF1,CCM2,GREM1,CXCL3,APP,SLC3A2,POSTN,TYMP,COL4A2,AGRN,CXCL5,JHDM1D,KRT17,NRP2,STX1A,CTGF,CALD1,SDC3,LCP2,PPFIA1,HPRT1,GAST,CCL11,THBS2,RNASEH2A,ANG,ISM1,CFP,APLNR,NTRK2,EMCN,PLA2G1B,ID4,CX3CL1,DDAH2,CD34,EFNA2,AGT,TP53,CMA1,NELL1,AMOT,TEK,SMPD3,SLIT3,CXCR2,MMRN1,ANGPT4,MAP2,SFRP4,CCR2,CLEC3B,DLEC1,CX3CR1,PBX1,CYP2C19,CEACAM1,VLDLR,CYP2C9,SOSTDC1,CAPN6,MAF,AQP1,ANGPTL1,KRT15,TFAP2B,TM7SF2,IL1RN,PTN,CXCL12,IGFBP2,CXCL17,FIGF,CCL14,SELE,CYP2C18

Inflammation 37 MMP3,S100A7A,SERPINE1,S100A7,SAA1,DUSP6,TREM1,NCF2,F3,KIF18A,TNS4,CCL13,ADM,PLA2G7,FBN2,OAS1,UBASH3B,PTGS1,SEC14L2,PTGES,CCL11,RNASEH2A,HSPB2,PLA2G1B,SERPINA5,GPRC5A,TST,AGT,CD79A,MYLIP,CXCR2,TTR,FOXE1,SFTPD,IL1RN,CYP3A4,SELE

Apoptosis 238 CXCL10,CD274,IL20,CXCL9,IFI6,IL24,DDX58,DUSP6,SLCO1B3,TLR2,IL2RA,XAF1,EIF2AK2,TPM1,FCGR3B,IRS1,AREG,AURKA,CTLA4,LRP12,F3,PDE7A,FAIM3,LYN,IGF2BP2,TNFRSF10B,SOD2,IRF1,CCNA1,DOCK5,IL32,PHLDA1,ECT2,IL7R,CCL18,STK17A,IFIH1,SDC4,PI3,IFI27,FOXM1,RAD51,CFB,ADM,PML,EXO1,CDC25B,SULF1,CCM2,CDC20,PRKDC,OAS1,MMP11,CCNA2,SOX9,CASP7,APP,RRAS,IFIT5,CHEK1,ITGB1,CEBPB,CALB1,PLXNA1,CD86,GNLY,COL4A2,NFIL3,RARRES3,NBN,MYBL2,ANXA5,RHOB,PLEC,ABCA1,BLM,EPHB2,APOL2,TNFSF10,CD14,BIRC2,BRCA2,SHC1,KDM1A,CKS2,STK3,ELF4,GLS,SPHK1,PTAFR,TGFB1,BIRC5,FBLIM1,AATF,DUSP4,PRKCQ,LMNB1,EGFR,SLC9A1,APAF1,ZAK,CCND2,HENMT1,SPECC1,QSOX2,PAG1,CD70,IFI16,KIF20B,LIMS1,MAD2L1,ANKRD1,ATIC,ATG5,PRIM2,RBBP8,CASP1,ABCC3,RAPGEF1,TGIF1,FADD,GAST,MICU1,RAC2,PLEK,TBP,BCL2L11,C5AR1,UCHL1,MCL1,GZMA,CHMP5,MPHOSPH6,JUN,SKIL,RNASEH2A,PRDM14,PLAGL1,LDHD,OSGIN1,NUPR1,HSPB2,HMGCR,NPBWR2,APBB1,MS4A1,NTRK2,PLA2G1B,ID4,IL17RB,PEBP4,TYRO3,MSMB,PIM1,GALR2,TF,TP53,MMAB,ELANE,ASAH1,CADM1,KCNIP3,CTNNBIP1,CRYM,IRX5,SNCAIP,DACT3,ITPR2,DUOXA1,MYLIP,CMTM5,BMX,MAS1L,MAPK7,CD19,GAS1,NOTCH3,RARG,FGFR2,TRPV6,CXCR2,NLRP2,ABCB1,HSPB8,RHOV,FOXE1,DIRAS1,SFRP4,NRTN,CFTR,NTRK3,EVPL,SMPD2,DIRAS3,DLEC1,PTK6,KLK7,CLU,MXI1,CEACAM6,NOS1,HSPA1A,MAP3K1,GDF10,TPPP3,KLK1,P2RY2,PTGDS,MEIS1,BEX2,CCL19,CYP2J2,SASH1,CAPN6,KLF4,PADI1,BCL2L10,AQP3,SMO,TGM1,CYSLTR1,TRPS1,LDOC1,MAPT,IGFBP5,CXCL12,FETUB,MAP1LC3A,AADAC,SCNN1A,DNASE1L3,RAET1E,PITX2,FAM3B,GPX3,HLF,DCT,TYR

<<<<<<<<<<<<<<<<<<<<<<<<<<<<<<<<<<<<<<<<<<<<<<<<<<<

>>>>>>>>>>>>>>>>>>>>>>>>>>>>>>>>>>>>>>>>>>>>>>>>>>

Genes sharing common annotation or function

###########################################

CommonAnnotation No. of genes Genelist

--------------------------------------------

Diagnostic#Angiogenesis 1 APLNR,CD34,MAP2,MAF,KRT15

Therapeutic#Prognostic#Diagnostic#Angiogenesis#Apoptosis#Metastasis#CellProliferation#Immortality 1 FOXM1

Angiogenesis#CellProliferation 1 GREM1,HPRT1,ANGPTL1

Therapeutic#Diagnostic 1 KIF14,CEP55,ICOS,FCGR2A,BIRC3,PYGB,CAPS,CD79B,HTR1B,S1PR1,SOX10,SNED1,UGT1A6,EHF,ARG1,ALDH3A1,XIST,MAL

Therapeutic#Inflammation 1 S100A7A,KIF18A,PTGES

Prognostic#Angiogenesis#Metastasis 1 ETS1,WISP3,SDC3

Angiogenesis#Immortality 1 PBX1

Therapeutic#Diagnostic#Angiogenesis#Apoptosis#Metastasis#CellProliferation 1 DLEC1

Therapeutic#Prognostic#Diagnostic#Apoptosis#Metastasis#CellProliferation 1 CLU,TYR

Therapeutic#Prognostic#Diagnostic#CellProliferation#Inflammation 1 CD79A

Therapeutic#Prognostic#Metastasis 1 TNFRSF12A,PLAUR,HMMR,VMP1,PSMA7,RHOC,ADAM9,DLX4,EDNRB,TIMP4,LYPD3,BMP7

Therapeutic#Diagnostic#Apoptosis 1 MCL1

Therapeutic#Prognostic#Diagnostic#Angiogenesis#Metastasis 1 VEGFC

Therapeutic#Prognostic#Diagnostic#Apoptosis#Metastasis 1 UCHL1,CEACAM6,KLK1

Therapeutic#Apoptosis#Metastasis 1 NBN,PEBP4,MSMB

Therapeutic#Prognostic#Apoptosis#CellProliferation 1 LYN,IFI27,SOX9,NOTCH3,TRPV6,IGFBP5

Therapeutic#Prognostic#Diagnostic#Angiogenesis#Apoptosis#CellProliferation#Immortality 1 TP53

Therapeutic#Angiogenesis#Metastasis#CellProliferation#Inflammation 1 AGT

Diagnostic#Metastasis#Inflammation 1 TTR

Therapeutic#Prognostic#Metastasis#ImmunoModulation 1 TYRP1

Therapeutic#CellProliferation#Inflammation 1 CYP3A4

Therapeutic#Prognostic#Angiogenesis#Metastasis#CellProliferation 1 SLC3A2,PTN

Therapeutic#Diagnostic#Metastasis 1 FAT1,AR,GUCY2C

Angiogenesis 1 MMP7,WARS,CXCL3,POSTN,AGRN,JHDM1D,LCP2,CFP,EMCN,DDAH2,CYP2C19,VLDLR,CYP2C9,SOSTDC1,TFAP2B,TM7SF2,CXCL17

Therapeutic#Prognostic#Apoptosis#Metastasis 1 CHEK1

Therapeutic#Angiogenesis#CellProliferation#Inflammation 1 IL1RN

Prognostic#Metastasis#Inflammation 1 SERPINA5

Diagnostic#Apoptosis 1 IFIT5,CALB1,PRIM2

Therapeutic#Metastasis 1 SPINK6,SNAI2,DCBLD2,LOXL2,ASAP1,BRMS1,PTP4A1,SELL,CD151,EFNB1,LTB4R2,CYP17A1,KRT31,PITPNM3,CTSG,PRSS3,NFIX,LY6D,SPINK7

Prognostic#Apoptosis#Inflammation 1 HSPB2

Therapeutic#Prognostic#Diagnostic#Angiogenesis#Metastasis#Inflammation 1 SELE

Angiogenesis#Apoptosis 1 IL20,CCM2

Therapeutic#Angiogenesis#Apoptosis#Metastasis#Inflammation 1 F3

ImmunoModulation 1 MIR31HG,HTR7,C3orf52,LOC440354,AOC4,MIR210HG,GNG7,DDX11L2,PGD

Prognostic#Metastasis#CellProliferation 1 ANKS1B

Prognostic#Angiogenesis 1 ANGPT2,KRT17,EFNA2,AMOT,SMPD3,ANGPT4,CEACAM1

Therapeutic#Angiogenesis#Metastasis 1 ITGAV,MMRN1,CCR2,FIGF

Therapeutic#Apoptosis 1 IL24,AURKA,CDC20,PRKDC,RRAS,CEBPB,EPHB2,TNFSF10,GLS,PTAFR,KIF20B,ABCC3,TGIF1,C5AR1,GZMA,PRDM14,IL17RB,PIM1,MMAB,ASAH1,KCNIP3,CRYM,DACT3,BMX,FGFR2,DIRAS1,GDF10,BCL2L10,CYSLTR1

Therapeutic#Prognostic#Diagnostic#Apoptosis 1 XAF1,RAD51,CDC25B,GNLY,ABCB1,MAPT

Apoptosis#Immortality 1 AATF

Prognostic#Diagnostic#CellProliferation 1 PITX1

Therapeutic#Prognostic#Apoptosis#CellProliferation#Immortality 1 PTK6

Angiogenesis#Inflammation 1 CCL11

Prognostic 1 INHBA,IFIT3,MMP13,SEMA3C,TGFBI,SPP1,LAMC2,GALNT6,LAMA3,CCL20,PFN2,ITGA1,SEMA3A,IFITM1,ANLN,ADAMTS12,ISG15,OCIAD2,TRIB1,IL1RL1,ANXA3,RGS1,BUB1B,HLA-A,CYR61,MMP14,FXYD5,CCNB1,HLA-F,THBS1,CDK1,AJAP1,PROCR,SCRN1,AFAP1L1,PICALM,MYBPH,KIF4A,GABRP,ABL2,TAP2,NMI,MLLT11,CD163,UBD,PSMB7,SRGN,PSMB8,SLC1A5,HOXA1,BAK1,SNRPB,MRPL28,PTGIS,BAMBI,CYB5A,C2orf40,MALL,CYP26A1,EPHA7,LIFR,BDKRB1,PLCD1,SELENBP1,PVRL4,HLA-DQA1,NEGR1,CFD,CIDEB,SEMA3G,CNKSR3,PACRG,SLC16A7,SOST,UPK1A,CD24,SLC9A3R1,CLIC3,SLURP1,COL14A1,PLP1,KLK11,KLK12,DACT2,CHL1,EPHX3

Metastasis#Inflammation 1 MMP3,SAA1

Prognostic#Apoptosis#CellProliferation 1 IGF2BP2,MYBL2,RBBP8,NUPR1,GALR2,MXI1

Therapeutic#Prognostic#Angiogenesis#Apoptosis#Metastasis#CellProliferation 1 NTRK2

Therapeutic#Angiogenesis#Apoptosis#CellProliferation#Inflammation 1 ADM,RNASEH2A

Prognostic#Diagnostic#Apoptosis 1 PLEC,FBLIM1,OSGIN1

Apoptosis#CellProliferation 1 IL7R,SKIL,GAS1,RARG,P2RY2

Prognostic#Inflammation 1 TREM1,NCF2,TNS4,SEC14L2

Apoptosis#Metastasis 1 SDC4,CASP7,ITGB1,ZAK,RAC2,ELANE

Prognostic#Apoptosis#Metastasis#CellProliferation 1 SHC1,NTRK3,SASH1

Therapeutic#Diagnostic#Apoptosis#Metastasis#CellProliferation 1 ABCA1

Therapeutic#Diagnostic#Angiogenesis#Apoptosis#CellProliferation 1 SFRP4

Therapeutic#Prognostic#Apoptosis#Metastasis#CellProliferation 1 PML,BRCA2,KLF4

Diagnostic#Inflammation 1 FBN2,GPRC5A,SFTPD

Therapeutic#Prognostic#Diagnostic#Metastasis#CellProliferation 1 WNT5A,POMC

Prognostic#Diagnostic#Apoptosis#Metastasis 1 CADM1

Apoptosis#Inflammation 1 DUSP6,OAS1,FOXE1

Prognostic#Apoptosis#Metastasis 1 CKS2

Therapeutic#Prognostic#Diagnostic#Apoptosis#CellProliferation 1 CD14,EGFR,CCND2,MS4A1

Metastasis 1 MMP1,MMP10,PTHLH,PDPN,CDH3,HOXD10,CTSL1,PANX1,LGALS1,BST2,FMNL2,ETV4,FAP,PTPN12,F2RL2,ADAM19,FNDC3B,IL6,COL7A1,MT1L,ST3GAL5,LIMK1,HOXB9,MYH9,BCL2A1,MAP2K6,AGPAT9,DKK2,FAM84A,ZG16B,DPEP1,DCN,DIO2,ISL1,PLD1,ASAP3,CADM4,RGS5,CRAT,DARC,JAM2,OCLN,CCL15,TGFBR3,TSPAN8,BARX2,DKK4,FUT3,FRZB,OGN,MUC15,KRT13,TGM3

Diagnostic 1 ITGA3,LTBP1,TNFAIP3,STC1,S100P,ATP6V1C1,STAT1,MCM4,XRCC2,ADA,SLC38A1,HELLS,GGH,ERRFI1,ANO6,FGF23,LLGL2,ACSBG1,DBI,KRT10,DBP,KIR3DL2,ATP1B2,PBOV1,AGSK1,KRT19,IVL,MLANA,CLCA4,SERPINB11

Therapeutic#Prognostic 1 PDCD1LG2,CA9,BATF2,FSCN1,WNT7A,OSMR,MSN,HJURP,SLC22A1,HAVCR2,KIF2C,APLN,ASPM,FLNA,VSIG1,SAMSN1,RARRES1,CAV2,IL1RAP,SLC5A5,ISX,CACNA2D3,NR0B1,CRABP1,VWA5A,CBR1,SDR9C7,SLC5A1

Therapeutic#Prognostic#Diagnostic 1 MTAP,DEPDC1,AURKB,RGS22,KLK13,ODAM

Therapeutic#Apoptosis#CellProliferation 1 EIF2AK2,CTLA4,SOD2,CCNA1,CCNA2,CD86,BLM,KDM1A,ATIC,HMGCR,HSPB8,EVPL,MEIS1,SMO,DCT

Prognostic#CellProliferation 1 FEZ1,TPX2,SLC6A2,CENPF,TK1,PKP2,CCNB2,NNAT,CBX7,DLX5,MAGI1,PALM

Inflammation 1 S100A7,CCL13,PLA2G7,UBASH3B,PTGS1,TST

Metastasis#CellProliferation 1 IFI44,ITGA5,TRIO,F2RL1

Therapeutic#Angiogenesis#Apoptosis#Metastasis 1 IL32,APP

Apoptosis#Metastasis#CellProliferation 1 ECT2,DIRAS3,CCL19

Therapeutic#Prognostic#Diagnostic#Apoptosis#CellProliferation#Inflammation 1 MYLIP

Therapeutic#Angiogenesis#Apoptosis#Inflammation 1 PLA2G1B

Therapeutic#Angiogenesis#Apoptosis#Metastasis#CellProliferation#Inflammation 1 CXCR2

Diagnostic#CellProliferation 1 FST,PDGFA,FABP4,CLSPN,CBFA2T3,EPHX2

Prognostic#Diagnostic 1 MUCL1,HMGA2,CAV1,FAM83A,CDC6,STIL,SCD5,NOP2,TACSTD2,KRT20,BCL11A,FGF12,KIT,TRPM1,CHRNA3,TSPAN7

Therapeutic#Diagnostic#Apoptosis#CellProliferation 1 IL2RA,CD70,TYRO3,TF

Diagnostic#Metastasis 1 TNC,LY6K,FN1,CALB2,CLDN5,PDZD2,ELN,SLC38A3,ECM1

Therapeutic#Prognostic#Diagnostic#Angiogenesis#Apoptosis#Metastasis#CellProliferation 1 CXCL10,GAST,CXCL12

Therapeutic#Metastasis#CellProliferation 1 TSPAN1,NUDCD1,CCL21,GAB2,NOV,GNRHR,WISP2

Therapeutic#Angiogenesis 1 CXCL11,NELL2,IL8,E2F7,NRP2,STX1A,ISM1,CX3CL1,NELL1,AQP1,CCL14,CYP2C18

Diagnostic#Apoptosis#CellProliferation 1 IRS1

Therapeutic#Prognostic#Angiogenesis#Metastasis#Inflammation 1 SERPINE1

Therapeutic#Prognostic#Metastasis#CellProliferation 1 PAK1

Therapeutic#Prognostic#Angiogenesis 1 ESM1,TYMP,CMA1,TEK

Prognostic#Metastasis 1 PLAU,MET,LGALS3BP,APOBEC3A,ITGA2,LAMP3,ITGA6,CD44,NUAK1,NME1,LAMB3,ITIH5,FUT7,NDRG2,ADAM23,MACC1,CLDN7,DSG1

Therapeutic#Prognostic#CellProliferation 1 MELK,ATAD2,CCNE1,CENPE,KIAA0101,SPRY2,APOD

Therapeutic#Prognostic#Diagnostic#Angiogenesis#Metastasis#CellProliferation 1 CXCL5,CTGF,SLIT3,IGFBP2

Therapeutic#Prognostic#Diagnostic#Metastasis 1 MMP9,CDKN2A,IGF2BP3,PSCA,TMPRSS2,ACPP,PMEL

Therapeutic#Prognostic#Angiogenesis#CellProliferation 1 ANG

Therapeutic 1 IFIT1,IDO1,IFIT2,PLAC1,SLC16A1,CD80,PHLDB2,HOXC9,IFNAR2,MAGEA4,SERPINE2,GLIPR1,PSMB9,PLSCR1,CCL4,NRIP1,RTP3,TXNRD1,GBP1,TTK,IL13RA2,MX2,RTKN,CCL8,APOBEC3B,LPAR3,SOCS3,KIF23,ACSL5,TINAGL1,ZHX2,PXN,IFNG,TNFSF18,NUP37,KIF11,BACH1,NAPB,CDCA3,SMS,XCL1,NCAPG2,LEPREL1,STK10,CIT,ANTXR2,PLEKHA2,PRSS23,CDCA8,RECQL,ACLY,FGFR3,PPM1D,ZNF135,RAB11FIP1,BCL9,SPATA19,SSTR4,PSORS1C2,ABCA7,NCAM2,SMR3A,MPPED2,CNTFR,MTUS1,EPS8,C10orf90,MS4A2,CDON,MKNK2,LGALSL,CP,LGR6,ABCA3,LBP,ARSF,CRABP2,ASPA,KRT81,HBA2,CAMK1D,NUAK2,GDA,RSPO1,PDGFD,FZD10,FCER1A,GSTA1,SOX21,ROR1,CYP4B1,TMPRSS11E,PPL,TMPRSS11A

Therapeutic#CellProliferation 1 TAP1,CSF2,NDC80,TNFRSF9,GINS1,ATOH1,AMPD1,KAT2B,NUCB2,NR3C2,RORA,PRLR

Therapeutic#Apoptosis#Metastasis#CellProliferation 1 RHOB,SPHK1,MAPK7,PTGDS

Therapeutic#Angiogenesis#Apoptosis#Metastasis#CellProliferation 1 SULF1

Therapeutic#Diagnostic#CellProliferation 1 MAPK13

Therapeutic#Angiogenesis#Apoptosis 1 COL4A2,CAPN6

CellProliferation 1 CYP27B1,MKI67,NRG1,PMEPA1,CCL3,CCL3L3,STAT2,RBP1,EIF2S1,IL1B,ODC1,EXT1,ZYX,CDKN3,P2RY6,ITPR3,GLI3,KIF20A,IGF2BP1,TIMELESS,CXCL2,CYP24A1,SIX3,PDIK1L,RPS6KA6,IL17RD,TNFRSF17,TEC,GLUL,CCDC6,BTC,TSPYL5,MGLL,ART4,LRRC4,VIT,P2RY1,ELF5

Apoptosis 1 CXCL9,IFI6,DDX58,SLCO1B3,TLR2,FCGR3B,LRP12,PDE7A,TNFRSF10B,DOCK5,CCL18,STK17A,IFIH1,PI3,CFB,EXO1,MMP11,PLXNA1,NFIL3,RARRES3,ANXA5,APOL2,BIRC2,STK3,ELF4,TGFB1,BIRC5,DUSP4,PRKCQ,LMNB1,SLC9A1,HENMT1,SPECC1,QSOX2,PAG1,IFI16,LIMS1,ANKRD1,RAPGEF1,FADD,MICU1,PLEK,TBP,BCL2L11,CHMP5,MPHOSPH6,JUN,PLAGL1,LDHD,NPBWR2,APBB1,CTNNBIP1,IRX5,SNCAIP,ITPR2,DUOXA1,CMTM5,MAS1L,NLRP2,RHOV,NRTN,SMPD2,KLK7,NOS1,HSPA1A,MAP3K1,TPPP3,BEX2,CYP2J2,PADI1,AQP3,TGM1,TRPS1,LDOC1,FETUB,MAP1LC3A,AADAC,SCNN1A,DNASE1L3,RAET1E,FAM3B,GPX3,HLF

Therapeutic#Angiogenesis#Apoptosis#CellProliferation 1 ID4

Therapeutic#Prognostic#Apoptosis 1 CD274,IRF1,APAF1,MAD2L1,CFTR

Therapeutic#Prognostic#Angiogenesis#Apoptosis#CellProliferation 1 AREG

Prognostic#Apoptosis 1 TPM1,FAIM3,PHLDA1,ATG5,CASP1,PITX2

Therapeutic#Prognostic#Diagnostic#CellProliferation 1 UBE2C,TFRC,KCNH1,CD1C,CD1A

Angiogenesis#Metastasis 1 CALD1,PPFIA1,THBS2,CLEC3B,CX3CR1

Therapeutic#Diagnostic#Apoptosis#CellProliferation#Immortality 1 CD19

Prognostic#Diagnostic#Metastasis 1 CD1E,TEX101

<<<<<<<<<<<<<<<<<<<<<<<<<<<<<<<<<<<<<<<<<<<<<<<<<<<

Total No. of Genes Differentially expressed :2290

Total No. of Genes Annotated :1014

Total no. of Genes Significantly Annotated: 841
